# Design and Synthesis of New Quinoxaline Derivatives as Potential Histone Deacetylase Inhibitors Targeting Hepatocellular Carcinoma: *In Silico*, *In Vitro*, and SAR Studies

**DOI:** 10.3389/fchem.2021.725135

**Published:** 2021-09-22

**Authors:** Chao Ma, Mohammed S. Taghour, Amany Belal, Ahmed B. M. Mehany, Naglaa Mostafa, Ahmed Nabeeh, Ibrahim H. Eissa, Ahmed A. Al-Karmalawy

**Affiliations:** ^1^ Hepatobiliary and Pancreatic Surgery, Cancer Hospital of Zhengzhou University, Zhengzhou City, China; ^2^ Pharmaceutical Medicinal Chemistry and Drug Design Department, Faculty of Pharmacy (Boys), Al-Azhar University, Cairo, Egypt; ^3^ Department of Pharmaceutical Chemistry, College of Pharmacy, Taif University, Taif, Saudi Arabia; ^4^ Zoology Department, Faculty of Science (Boys), Al-Azhar University, Cairo, Egypt; ^5^ Biophysics Department, Faculty of Women for Arts, Science and Education, Ain Shams University, Cairo, Egypt; ^6^ Department of Pharmaceutical Medicinal Chemistry, Faculty of Pharmacy, Horus University-Egypt, New Damietta, Egypt

**Keywords:** quinoxaline, anti-proliferative, HDAC, apoptosis, molecular modeling, structure-activity relationship

## Abstract

Guided by the structural optimization principle and the promising anticancer effect of the quinoxaline nucleus, a new series of novel HDAC inhibitors were designed and synthesized. The synthesized compounds were designed to bear the reported pharmacophoric features of the HDAC inhibitors in addition to an extra moiety to occupy the non-used vacant deep pocket of the HDAC receptor. The newly prepared compounds were evaluated for their *in vitro* anti-proliferative activities against HepG-2 and HuH-7 liver cancer cell lines. The tested compounds showed promising anti-proliferative activities against both cell lines. The most active ten candidates (**6**_**c**_, **6**_**d**_, **6**_**f**_, **6**_**g**_, **6**_**k**_, **6**_**l**_, **7**_**b**_, **8**, **10**_**h**_, and **12**) were further evaluated for their effect on the gene expression levels of Bax as an apoptotic marker and Bcl-2 as an anti-apoptotic one. Moreover, they were evaluated for their ability to inhibit histone deacetylase (HDAC1, HDAC4, and HDAC6) activities. Compound **6**_**c**_ achieved the best cytotoxic activities on both HepG-2 and HuH-7 cell lines with IC_50_ values of 1.53 and 3.06 µM, respectively, and also it showed the most inhibitory activities on HDAC1, HDAC4, and HDAC6 with IC_50_ values of 1.76, 1.39, and 3.46 µM, respectively, compared to suberoylanilide hydroxamic acid (SAHA) as a reference drug (IC_50_ = 0.86, 0.97, and 0.93 µM, respectively). Furthermore, it achieved a more characteristic arrest in the growth of cell population of HepG-2 at both G0/G1 and S phases with 1.23-, and 1.18-fold, respectively, compared to that of the control, as determined by cell cycle analysis. Also, compound **6**_**c**_ showed a marked elevation in the AnxV-FITC apoptotic HepG-2 cells percentage in both early and late phases increasing the total apoptosis percentage by 9.98-, and 10.81-fold, respectively, compared to the control. Furthermore, docking studies were carried out to identify the proposed binding mode of the synthesized compounds towards the prospective target (HDAC4). *In silico* ADMET and toxicity studies revealed that most of the synthesized compounds have accepted profiles of drug-likeness with low toxicity. Finally, an interesting SAR analysis was concluded to help the future design of more potent HDACIs in the future by medicinal chemists.

**GRAPHICAL ABSTRACT:**
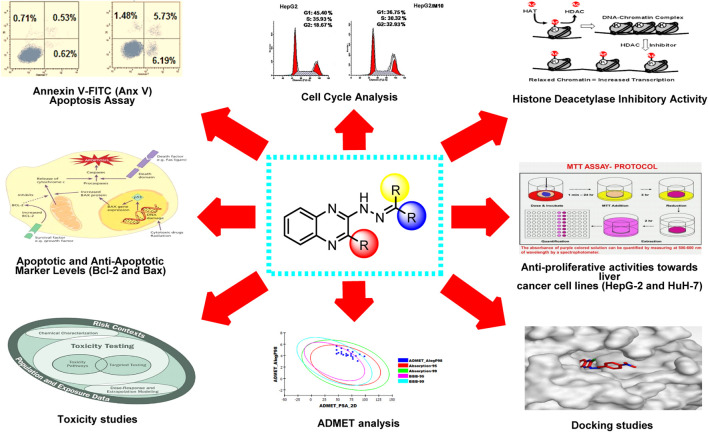


## Highlights

• Twenty-seven quinoxaline derivatives were designed and synthesized.
• Cytotoxic activities were evaluated against two liver cancer cell lines (HepG-2 and HuH-7).
• *In vitro* histone deacetylase-4 inhibitory activities were evaluated.
• The effect on cell cycle analysis was studied.
• The effect on apoptosis was studied.
• Apoptotic and anti-apoptotic marker levels (Bcl-2 and Bax) were evaluated.
• Molecular docking studies were carried out against histone deacetylase-4.
• ADMET analysis was done for the newly synthesized derivatives.
• Computational toxicity studies were done.
• SAR analysis was concluded.

## Introduction

Liver cancer, also known as hepatic cancer, may start in the liver (Yamashita and Wang, 2013) or spread from elsewhere to the liver, known as liver metastasis (McGuire, 2016). Symptoms include pain in the right side below the rib cage, swelling of the abdomen, yellowish skin, weight loss, and weakness (Liu, 2017). Liver cirrhosis from hepatitis B, hepatitis C, or alcohol is considered the main cause of liver cancer (Perz et al., 2006). The most common types of liver cancer are hepatocellular carcinoma (HCC), which contributes up to 80% of the cases, and cholangiocarcinoma (CCA), which are known as primary liver cancers (PLCs) (McGuire, 2016).

One of the most important challenges facing liver cancer types is their high resistance and poor response to chemotherapy. The produced resistance arises from synergistic interactions among diverse mechanisms of chemoresistance (MOC) in which about 100 genes are involved (Marin et al., 2018).

A very important pathway to fight cancer has appeared after the discovery of tumor angiogenesis by Judah Folkman approximately 50 years ago (Maj et al., 2016). Different effective antiangiogenic agents were approved, mostly targeting vascular endothelial growth factor (VEGF) (El-Helby et al., 2019a; El-Helby et al., 2019b). The antiangiogenic agents either increase the effectiveness of standard chemotherapy or even replace it completely. Now, there are novel strategies other than targeting the VEGF pathway, which are aimed at influencing the molecular factors involved in tumor angiogenesis (Maj et al., 2016). Subsequently, there is a great interest in the development of new antiangiogenic agents that could effectively inhibit tumor vascularization (Vasudev and Reynolds, 2014).

Hypoxia-inducible factor-α (HIF-1α), a central regulator of oxygen detection and adaptation at the cellular level, and its transcriptional activity are the key mediator of VEGF activity. Both HIF-1α and VEGF are crucial to angiogenesis and can be regulated by post-translational modifications (PTMs), including acetylation by histone acetyltransferases (HATs) and deacetylation by histone deacetylases (HDACs). Subsequently, many studies indicated HDAC inhibitors (HDACIs) as promising antiangiogenic compounds and recommended them as an effective class of anticancer therapeutics (Ellis et al., 2009; Deng et al., 2020).

The first HDACI approved by the FDA for the treatment of cutaneous T-cell lymphoma is vorinostat, followed by depsipeptide for the same purpose (Ververis et al., 2013). This was followed by the FDA approval of two other drugs, belinostat and panobinostat. Despite the success of HDACIs in the treatment of leukemias, they are still failing in the case of solid tumors (Sangwan et al., 2018). Many structurally diverse HDACIs are in different phases of clinical trials as a monotherapy and/or in combination with other anticancer agents (Marks and Xu, 2009).

**FIGURE 1:**
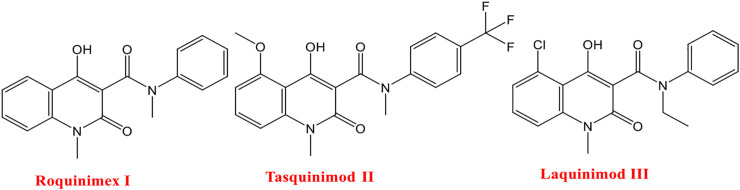
Some reported drugs acting as HDACIs.

Roquinimex **I** (linomide) is a quinoline derivative immunostimulant by increasing natural killer (NK) cell activity and macrophage cytotoxicity. It also inhibits angiogenesis and reduces the secretion of TNF-α. It was indicated for the treatment of some cancer types and autoimmune diseases, such as multiple sclerosis, and prevention of autoimmune diabetes mellitus (Gross et al., 2001; Banu et al., 2017). But several trials have been terminated due to observed cardiovascular toxicity (Tan et al., 2000). Tasquinimod **II** is a second-generation quinoline-3-carboxamide agent that is orally active in HDACI and is currently in phase III clinical trials for the treatment of castration-resistant prostate cancer (Gupta et al., 2014). It counteracts cancer development by inhibiting angiogenesis and metastasis and on the other hand by modulating the immune system (Isaacs et al., 2006; Jennbacken et al., 2012; Isaacs et al., 2013). Meanwhile, the mode of action for tasquinimod **II** is not fully understood, and several studies demonstrated its ability to interfere with tumor angiogenesis, cytokine production, macrophage infiltration, and autoimmune/inflammatory diseases. Furthermore, several studies including preclinical ones are required to investigate the real mechanisms of action for tasquinimod **II** (Gupta et al., 2014). Laquinimod **III** is an experimental immunomodulator and is being tested as an oral treatment for multiple sclerosis (MS). Phase III clinical trials for MS started in December 2007 and showed a slow progression of disability and reduction in the rate of relapse in patients with relapsing-remitting multiple sclerosis (Brück and Zamvil, 2012; Banu et al., 2017) (Figure 1).

The discovery of the histone deacetylases (HDACs) role has promised to be new hope for the treatment of various malignancies (Cappellacci et al., 2020). Thus, the intention for making new potent and safe anticancer therapeutic agents with minimal side effects is still a major concern for researchers nowadays. Besides, quinoxaline moiety is an important *N*-containing heterocycle in organic synthesis and drug discovery (Corona et al., 2009; Ibrahim, 2012; Ibrahim et al., 2013; Ibrahim et al., 2015; Eissa et al., 2018; Ibrahim et al., 2018; Abbass et al., 2020) due to its large scope of biological activity, especially antitumor activities (Corona et al., 2009). So, our goal is to design and synthesize new quinoxaline derivatives as antiangiogenic agents targeting HDAC with promising effects against liver cancer.

### Rational of Molecular Design

**FIGURE 2:**
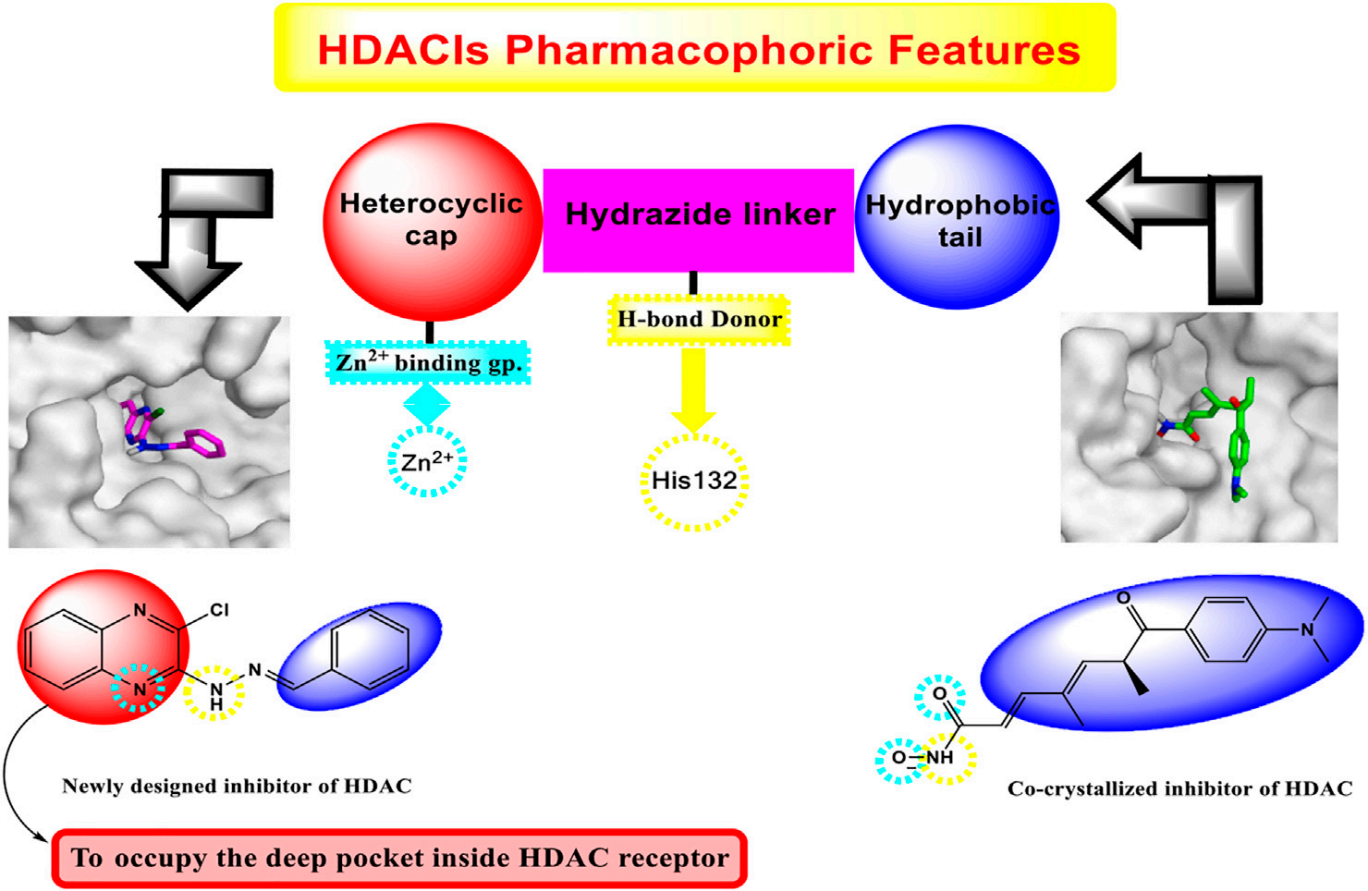
Graphical representation explaining the new idea of our rational.

Investigation of the common pharmacophoric features shared by various HDACIs revealed that most of them have three main features: i) a heterocyclic aromatic cap that occupies the narrow tubular pocket of HDAC and containing at least one H-bond acceptor. This H-bond acceptor constitutes the Zn^2+^-binding group, ii) an amide linker which occupies the linker region between the zinc-binding region and the hydrophobic tail region and contains one H-bond donor that forms an H-bond with the crucial amino acid His132, iii) a terminal hydrophobic group which protrudes to outside, similar to that of the co-crystallized inhibitor, and helps in the stabilization of the remaining part of the compound (Figure 2).

Interestingly, the newly synthesized quinoxaline candidates were found to form nearly the same binding mode compared to the co-crystallized inhibitor (TSA) with an additional extra positioning of the quinoxaline moiety inside the narrow tubular pocket of HDAC, which provides a more promising fitting as the N1 of quinoxaline, and it was found to be the zinc-binding group, as described in Figure 2.

**FIGURE 3:**
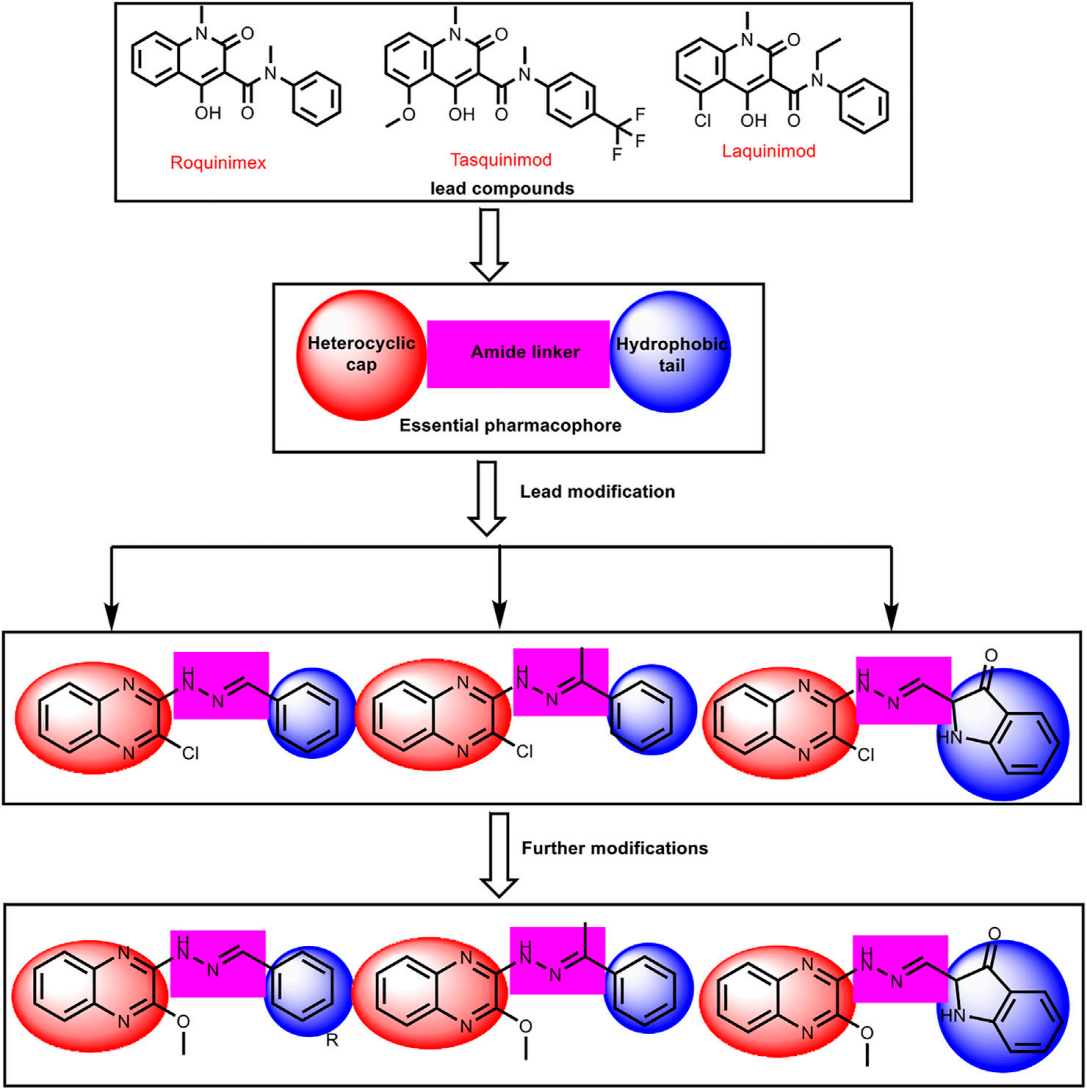
The basic structural requirements for roquinimex, tasquinimod, and laquinimod as reported HDAC inhibitors and their existence in our newly synthesized compounds.

In continuation of our previous works (Eissa et al., 2016; Eldehna et al., 2017; El-Naggar et al., 2017; Eissa et al., 2018; Gaber et al., 2018; Ibrahim et al., 2018; Elmetwally et al., 2019; Metwaly et al., 2019; Alesawy et al., 2020; Al-Karmalawy and Khattab, 2020; Eliaa et al., 2020; El-Zahabi et al., 2020; Ghanem et al., 2020; Al-Karmalawy and Elshal, 2021 and Elshal; El-Shershaby et al., 2021b; Khattab and Al-Karmalawy, 2021; Samra et al., 2021), that proved potential anticancer activities of novel chemical agents, a new series of quinoxaline derivatives were designed and synthesized to have the essential pharmacophoric features of the reported and clinically used HDACIs to get more potent antitumor molecules. The main core of our molecular design rational comprised bio-isosteric modification strategies of the lead compounds (roquinimex, tasquinimod, and laquinimod) at three different positions (Figure 3).

The first position was the heterocyclic aromatic cap. Two different substituted quinoxaline moieties were used as follows: 2-chloroquinoxaline (compounds **6**_**a-l**_ **, 7**_**a,b**_ **, 8,** and **9**) and 2-methoxyquinoxaline (compounds **10**_**a-i**_ **, 11,** and **12**). The choice of quinoxaline was based on some bio-isosteric considerations: i) the bicyclic structure of quinazoline core which occupies the narrow tubular pocket of HDAC superior to the co-crystallized inhibitor (Hou et al., 2003), ii) the nitrogen atoms serve as hydrogen-bond acceptors and one of them is acting as the zinc-binding region conferring excellent HDAC inhibitory activity. The second position was the linker (spacer) region. The amide linker of the lead compounds was modified to be methylenehydrazine (compounds **6**_**a-l**_ **, 8, 9, 10, 11**_**,**_ and **12**) or ethylidenehydrazine (compounds **7**_**a,b**_). The third position was the terminal hydrophobic group. We used many different aromatic moieties to play the role of the terminal hydrophobic group. The wide variety of modifications enabled us to study the SAR of these compounds as effective anticancer agents with potential HDAC inhibitory activities, which is considered a crucial objective of our work. All modification pathways and molecular design rational were illustrated and summarized in Figure 3 and Supplementary Figure SI1.

Also, to explore and emphasize the mechanism of action of the synthesized compounds, molecular docking studies were conducted to understand the expected binding interactions of the target compounds with HDAC active sites. For the same purpose, other studies regarding the ability of the most active compounds to induce apoptosis and arrest cell cycle growth have been done.

## Results and Discussion

### Chemistry

**Scheme 1:**
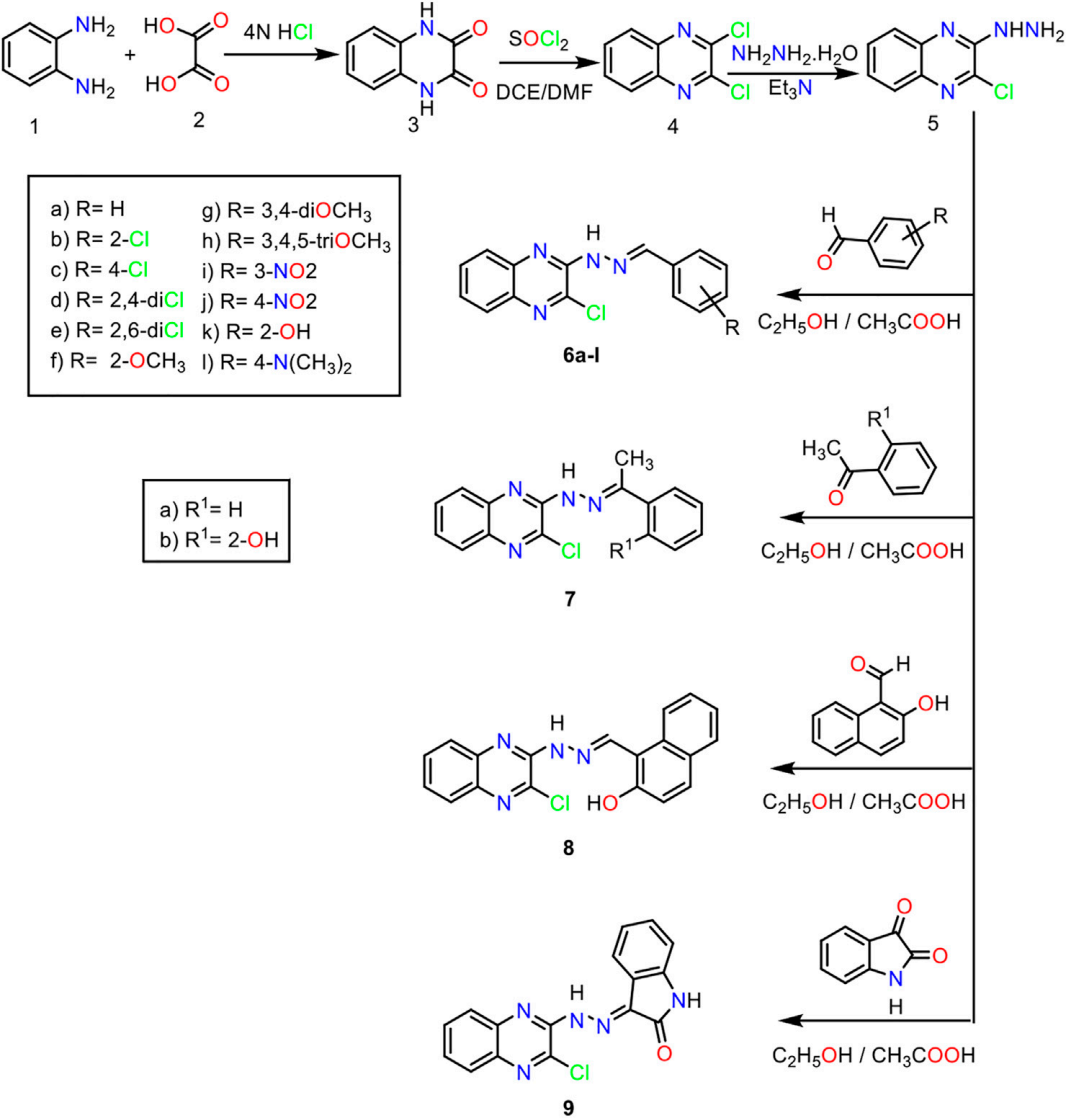
General procedure for the synthesis of compounds **6**_**a-l**_, **7**_**a,b**_, **8**, and **9**.

**Scheme 2:**
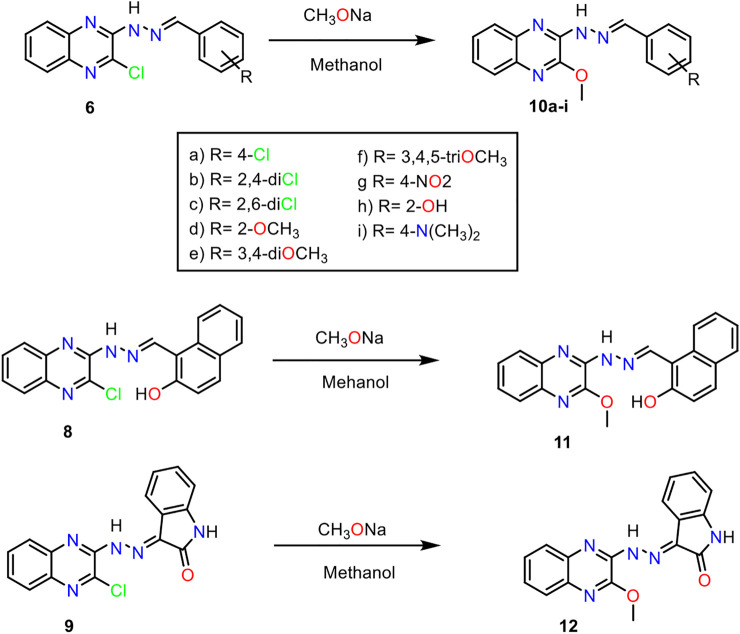
General procedure for the synthesis of compounds **10**_**a-i**_, **11**, and **12**.

For the synthesis of the target compounds, the reaction sequences were illustrated in Schemes 1, 2. At first, *o*-phenylenediamine was treated with oxalic acid in the presence of 4 N HCl to afford 2,3-(1*H*,4*H*)-quinoxalinedione **3**. The latter was treated with thionyl chloride in the presence of a catalytic amount of DMF to produce 2,3- dichloroquinoxaline **4** (Romer, 2009). Stirring of the 2,3 dichloroquinoxaline with hydrazine hydrate in the presence of TEA at room temperature afforded 2-chloro-3-hydrazinylquinoxaline **5**. Reflux of 2-chloro-3-hydrazinylquinoxaline with commercially available aldehydes and ketones, namely, benzaldehyde, 2-chlorobenzaldehyde, 4-chlorobenzaldehyde, 2,4-dichlorobenzaldehyde, 2,6-dichlorobenzaldehyde, 2-methoxybenzaldehyde, 3,4-dimethoxybenzaldehyde, 3,4,5-trimethoxybenzaldehyde, 3-nitrobenzaldehyde, 4-nitrobenzaldehyde, 2-hydroxybenzaldehyde, 4-*N,N*-dimethylaminobenzaldehyde, acetophenone, 2-hydroxyacetophenone, 2-hydroxy-1-naphthaldehyde, and isatin, in the presence of a catalytic amount of acetic acid produces target compounds **6**_**a-l**_, **7**_**a,b**_, **8**, and **9,** respectively (Scheme 1). The formation of benzylidene derivatives **6**_**a-l**_, **7**_**a,b**_, **8**, and **9** was confirmed by ^1^H NMR spectra, which showed the appearance of a singlet signal for benzylidene methine proton in the range of *δ* 7.80–8.00 ppm. This methine proton was detected also in both ^13^C NMR spectra resonating around 127.00 ppm. Besides, ^1^H NMR and ^13^C NMR spectra exhibited increased aromatic signals indicating that the condensation reactions were completed.

The hydrazone derivatives **6**_**c-h**_, **6**_**j-l**_ **, 8**, and **9** were treated with sodium methoxide to obtain final methoxy derivatives **10**_**a-i**_ **, 11,** and **12**, respectively (Scheme 2). The formation of methoxy derivatives **6**_**c-h**_, **6**_**j-l**_ **, 8**, and **9** was confirmed by ^1^H NMR spectra, which showed the appearance of a singlet signal for methoxy around *δ* 4.10 ppm. This methoxy group was detected also in both ^13^C NMR spectra resonating around 54.30 ppm.

### Biological Evaluation

#### Anti-Proliferative Activities Towards Liver Cancer Cell Lines (HepG-2 and HuH-7)

Anti-proliferative of the synthesized compounds was examined towards liver cancer HepG-2 and HuH-7 cell lines, using MTT assay (Skehan et al., 1990). The IC_50_ values for the synthesized derivatives were compared with doxorubicin as a positive control (Table 1).

**TABLE 1:** Anti-proliferative activities for **6**_**a-l**_, **7**_**a,b**_, **8**, **9**, **10**_**a-i**_, **11**, and **12** compounds against HepG-2 and HuH-7 liver cancer cell lines.
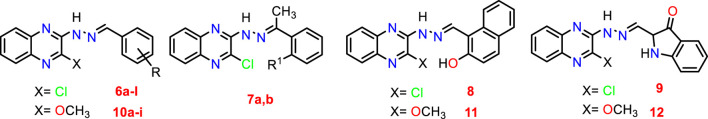

Compound	Quinoxaline derivative	R	R^1^	IC_50_ (µM)^a^ ^,^ ^b^	
				HepG-2	HuH-7
**6**_**a**_	2-Cl	H	—	14.29	18.17
**6**_**b**_	2-Cl	2-Cl	—	15.81	18.7
**6**_**c**_	2-Cl	4-Cl	—	1.53	3.06
**6**_**d**_	2-Cl	2,4-diCl	—	6.16	9.47
**6**_**e**_	2-Cl	2,6-diCl	—	13.51	17.5
**6**_**f**_	2-Cl	2-OCH_3_	—	6.63	8.52
**6**_**g**_	2-Cl	3,4-diOCH_3_	—	2.16	3.05
**6**_**h**_	2-Cl	3,4,5-triOCH_3_	—	11.79	14.68
**6**_**i**_	2-Cl	3-NO_2_	—	63.87	65.72
**6**_**j**_	2-Cl	4-NO_2_	—	15.94	25.82
**6**_**k**_	2-Cl	2-OH	—	7.71	6.52
**6**_**l**_	2-Cl	4-N(CH_3_)_2_	—	5.39	9.27
**7**_**a**_	2-Cl	—	H	28.52	37.11
**7**_**b**_	2-Cl	—	2-OH	4.03	6.19
**8**	2-Cl	—	—	3.88	6.14
**9**	2-Cl	—	—	11.58	16.43
**10**_**a**_	2-OCH_3_	4-Cl	—	53.87	57.84
**10**_**b**_	2-OCH_3_	2,4-diCl	—	32.06	35.24
**10**_**c**_	2-OCH_3_	2,6-diCl	—	60.46	65.35
**10**_**d**_	2-OCH_3_	2-OCH_3_	—	28.6	27.31
**10**_**e**_	2-OCH_3_	3,4-diOCH_3_	—	55.74	50.62
**10**_**f**_	2-OCH_3_	3,4,5-triOCH_3_	—	56.92	48.81
**10**_**g**_	2-OCH_3_	4-NO_2_	—	12.97	19.12
**10**_**h**_	2-OCH_3_	2-OH	—	5.56	6.72
**10**_**i**_	2-OCH_3_	4-N(CH_3_)_2_	—	23.01	22.68
**11**	2-OCH_3_	—	—	18.47	20.31
**12**	2-OCH_3_	—	—	1.99	3.08
**13**	Doxorubicin			8.25	9.2 ± 0.65

a IC_50_ values are the mean ± S.D. of three separate experiments.

b IC_50_ (µM): 1–10 (very strong), 11–20 (strong), 21–30 (moderate), 31–50 (weak).

To conclude a reasonable structure-activity relationship (SAR) of quinoxaline-based HDACIs, five series: **6**, **7**, **8**, **9**, and **10,** was designed through hybridization between quinoxaline and different aldehydes, acetophenones, 2-hydroxy-1-naphthaldehyde, or isatin, and at the same time, substitution of 2-chloro group with 2-methoxy one to study their different effects.

Analyzing the IC_50_ values of the newly synthesized compounds on both HepG-2 and HuH-7 liver cancer cell lines revealed the following interesting results. Compounds of **6** series showed the best promising cytotoxic activities with IC_50_ ranging from 1.53 to 18.70 µM against the two cell lines except for compound **6**_**i**_ with a 3-NO_2_ side chain (IC_50_ = 63.87 and 65.72 µM, respectively). Compound **6**_**c**_ with a 4-Cl side chain showed the best cytotoxic activities against both cell lines among all the synthesized series (IC_50_ = 1.53 and 3.06 µM, respectively).

On the other hand, the derivatives of **10** series incorporating 2-OCH_3_ group at 2-position of quinoxaline nucleus showed a decrease in the cytotoxic activities ranging from 5.56 to 65.46 µM against the two cell lines, except for compound **10**_**h**_ incorporating 2-OH group in the side chain which elevated greatly its cytotoxic activity (IC_50_ = 5.56 and 6.72 µM) against both HepG-2 and HuH-7 cell lines, respectively.

The two compounds of the **7** series showed variable anticancer activities. On the one hand, compound **7**_**b**_ incorporates 2-OH (IC_50_ = 4.03 and 6.19 µM against HepG-2 and HuH-7, respectively). On the other hand, the unsubstituted member **7a** showed decreased activities against the two cell lines (IC_50_ = 28.52 and 37.11 µM against HepG-2 and HuH-7, respectively).

The presence of 2-hydroxy-1-naphthyl moiety in the side chain of compound **8** made it superior to compound **9** incorporating isatin moiety against the two cell lines. Compound **8** (IC_50_ = 3.88 and 6.14 µM against HepG-2 and HuH-7, respectively) was 2.98-fold more active than compound 9 (IC_50_ = 11.58 and 16.43 µM against HepG-2 and HuH-7, respectively).

Insertion of the 2-Cl group in quinoxaline moiety of compound **8** (IC_50_ = 3.88 and 6.14 µM against HepG-2 and HuH-7, respectively) makes it more active than the corresponding member **11** incorporating 2-OCH_3_ (IC_50_ = 18.47 and 20.31 µM against HepG-2 and HuH-7, respectively). Compound **8** showed increase activities by 4.76-fold against the HepG-2 cell line and 3.30-fold against the HuH-7 cell line. Contrary to the above, the insertion of the 2-OCH_3_ group in quinoxaline moiety of compound **12** (IC_50_ = 1.99 and 3.08 µM against HepG-2 and HuH-7, respectively) make it more active than the corresponding member **9** incorporating 2-Cl (IC_50_ = 11.58 and 16.43 µM against HepG-2 and HuH-7, respectively) Compound **12** showed increase activities by 5.81-fold against the HepG-2 cell line and 5.33-fold against the HuH-7 cell line.

#### Histone Deacetylase Inhibitory Activities

The most active compounds (**6**_**c**_, **6**_**d**_, **6**_**f**_, **6**_**g**_, **6**_**k**_, **6**_**l**_, **7**_**b**_, **8**, **10**_**h**_, **12**) were further evaluated for their ability to inhibit histone deacetylase activities (HDAC1, HDAC4, and HDAC6) (Table 2). The HDAC4 enzyme was chosen since it is widely seen in early tumorigenesis through deacetylation and demethylation of the residues (especially the lysine residues) of the histone H4 as the most important feature in cancer prognosis (Montgomery et al., 2007). Moreover, both HDAC1 and HDAC6 enzymes were evaluated to further confirm the HDAC inhibitory activities of the newly designed and synthesized compounds. The tested compounds showed promising HDAC inhibitory activities compared to the reference drug, suberoylanilide hydroxamic acid (SAHA). Especially compounds **6**_**c**_, **6**_**g**_, and **12** achieved the highest inhibitory activities against HDAC1, HDAC4, and HDAC6 as well. Again, compound **6**_**c**_ showed better HDAC inhibitory activities compared to the reference standard that clarifies greatly its promising targeting as an HDAC inhibitor after further preclinical and clinical studies.

**TABLE 2:** Effect of conjugates (**6**_**c**_, **6**_**d**_, **6**_**f**_, **6**_**g**_, **6**_**k**_, **6**_**l**_, **7**_**b**_, **8**, **10**_**h**_, and **12**) as HDAC inhibitors.

Compound	HDAC1 IC_50_ (µM)^a^	HDAC4 IC_50_ (µM)^a^	HDAC6 IC_50_ (µM)^a^
**6**_**c**_	1.76	1.39	3.46
**6**_**d**_	3.52	3.25	4.28
**6**_**f**_	3.29	3.44	5.32
**6**_**g**_	2.17	1.64	3.81
**6**_**k**_	4.18	3.81	6.29
**6**_**l**_	3.29	2.79	4.09
**7**_**b**_	2.67	2.51	5.24
**8**	1.72	2.09	4.37
**10**_**h**_	3.54	3.15	7.21
**12**	2.01	1.72	3.62
**SAHA**	0.86	0.97	0.93z

a Each experiment was repeated twice. SAHA (suberoylanilide hydroxamic acid).

#### Cell Cycle Analysis

**FIGURE 4:**
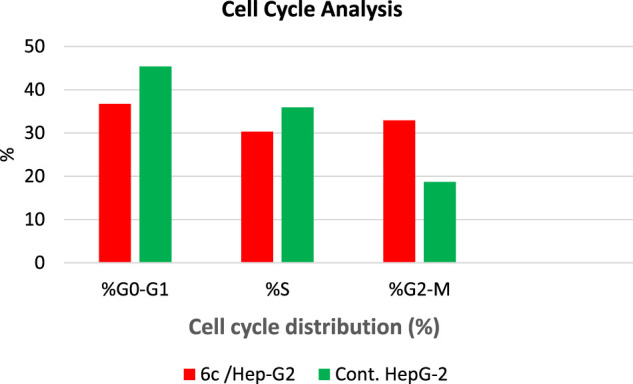
Cell cycle analysis of HepG-2 cells treated with compound **6**_**c**_ at 1.53 µM concentration.

The most compound **6**_**c**_ incorporating 4-Cl benzylidene side chain was further evaluated through cell cycle analysis in HepG-2 cell line. Such a test was carried out to detect the exact phase at which cell cycle arrest takes place. The HepG-2 cells were treated with compound **6**_**c**_ at a concentration of 1.53 µM equal to its IC_50_, and its impact on the different phases of cell growth was recorded. Treatment of HepG-2 cells with **6**_**c**_ showed a significant decline in the cell population at both G0/G1 and S phases with 36.75% (1.23-fold) and 30.32% (1.18-fold), respectively, compared to that of the control which was 45.40 and 35.95% (Table 3 and Figure 4). Besides, there was an increase in the population of cells at the G2/M phase by 1.76-fold compared to the control. This indicates that the compound halted the cell cycle progression of HepG-2 cells in the G0/G1 phase.

**TABLE 3:** Effect of compound **6c** on the cell cycle phases of HepG-2 cells.

Compound	%G0-G1	%S	%G2/M
**6**_**c**_ **/Hep-G2**	36.75	30.32	32.93
**Cont. HepG-2**	45.40	35.93	18.67
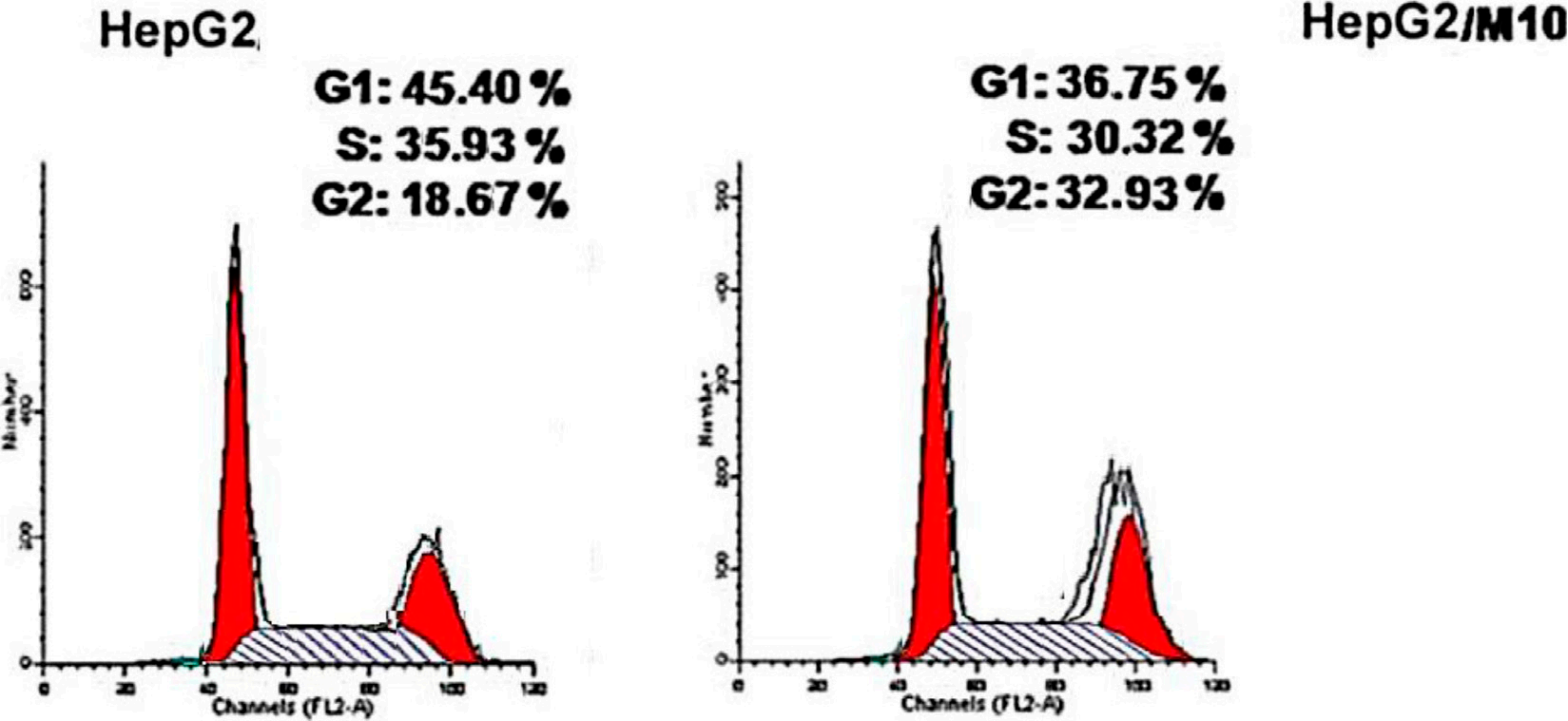

#### Annexin V-FITC Apoptosis Assay

**FIGURE 5:**
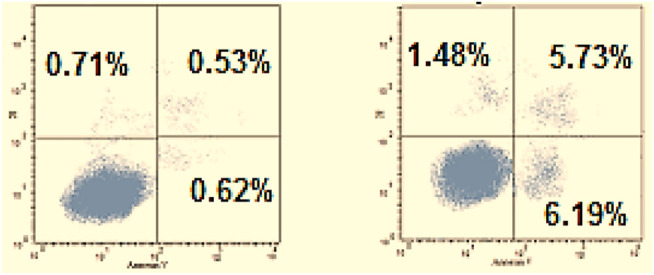
Influence of **6c** on the percentage of annexin V-FITC-positive staining in HepG-2 cells.

It is well-known that cell death may be due to programmed apoptosis or uncontrolled necrosis. Annexin V-based flow cytometry assay as a helpful strategy to determine the exact cause of death was carried out. Since compound **6**_**c**_ showed the highest anticancer activity toward the HepG-2 cell line, it was tested to investigate its apoptotic effect. The results revealed that the treatment of HepG-2 cells with **6**_**c**_ with a concentration of 1.53 µM showed a marked elevation in the AnxV-FITC apoptotic cells percentage in both early (from 0.62 to 6.19%, respectively) and late apoptosis (from 0.53 to 5.73%, respectively) phases (Figure 5). This refers to an increase in the total apoptosis percentage by 9.98-, and 10.81-fold, respectively, compared to the control. This confirms that the cytotoxic activity of compound **6**_**c**_ is due to programmed apoptosis, and not to nonspecific necrosis.

#### Apoptotic and Anti-Apoptotic Marker Levels (Bcl-2 and Bax)

To prove that the synthesized compounds exert their cytotoxic effects through driving cells to apoptosis, the effect of most active compounds against the level of Bax (as an apoptotic marker) and Bcl-2 (as an anti-apoptotic marker) was evaluated. Compounds **6**_**c**_, **6**_**d**_, **6**_**f**_, **6**_**g**_, **6**_**k**_, **6**_**l**_, **7**_**b**_, **8**, **10**_**h**_, and **12** were utilized in this test.

Auspiciously, the tested members greatly elevated the level of proapoptotic Bax protein by a range from 164.3 to 274.3 pg/ml (from 6.74 to 11.25-fold increase), compared to the control (24.37 pg/ml). On the other hand, all the tested compounds were able to decrease the level of the anti-apoptotic Bcl-2 protein by a range from 2.91 to 1.27 pg/ml (from 1.73 to 3.96-fold decrease), compared to the control (5.04 pg/ml). Again, compound **6**_**c**_ among all of the tested derivatives achieved the highest increase in the level of Bax protein (274.3 pg/ml) and the lowest decrease in the level of Bcl-2 protein (1.27 pg/ml), compared to the control (24.37 and 5.04 pg/ml), respectively (Table 4).

**TABLE 4:** Effect of compounds **6**_**c**_, **6**_**d**_, **6**_**f**_, **6**_**g**_, **6**_**k**_, **6**_**l**_, **7**_**b**_, **8**, **10**_**h**_, and **12** on the expression levels of Bcl-2 and Bax in HepG-2 cells.

Compound	Bax (pg/ml)	Bcl-2 (pg/ml)
**6**_**c**_	274.3	1.278
**6**_**d**_	179.2	2.711
**6**_**f**_	185.7	2.705
**6**_**g**_	247.5	1.687
**6**_**k**_	164.3	2.914
**6**_**l**_	219.7	2.519
**7**_**b**_	217.9	2.371
**8**	233.6	1.839
**10**_**h**_	205.4	2.673
**12**	257.9	1.571
**Control**	24.37	5.048

### *In silico* Studies Results

#### Docking Studies

Docking studies of the synthesized compounds were carried out to rationalize the obtained biological results and to understand the proposed binding mode of such compounds with the prospective target (HDAC). Trichostatin A (TSA) as an HDAC inhibitor was used as a reference drug in the docking studies.

**FIGURE 6:**
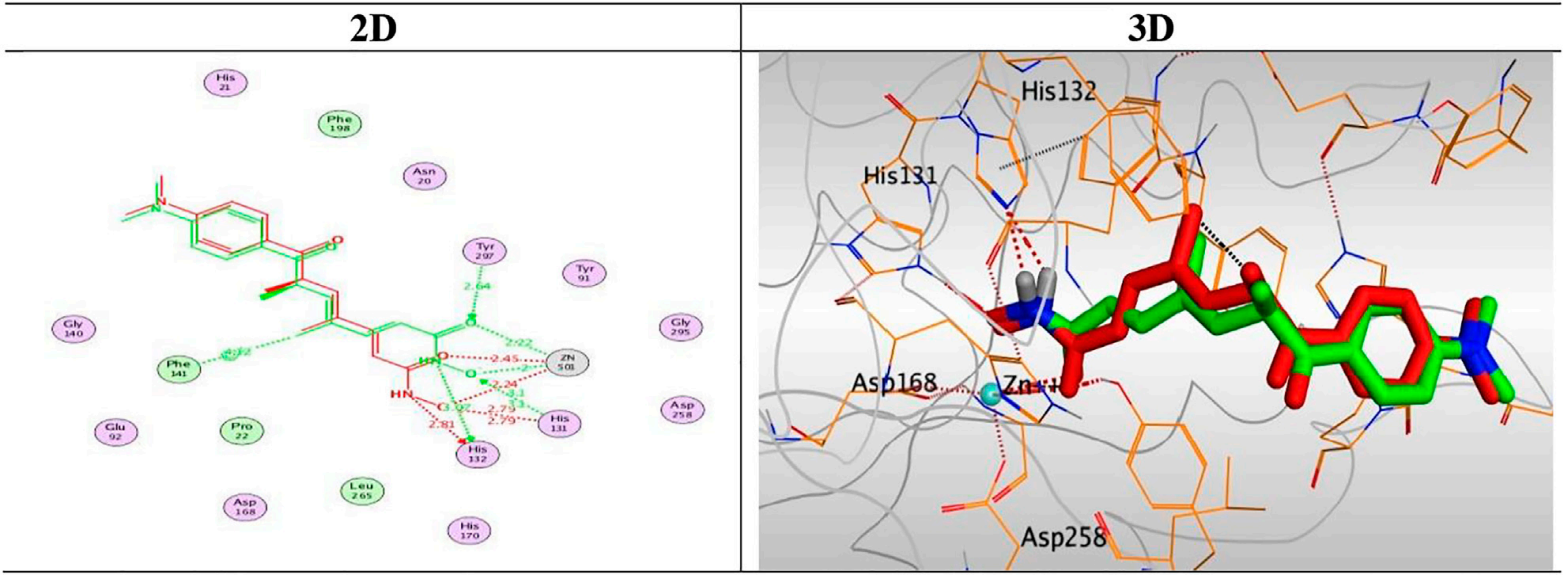
2D and 3D representations for the redocked co-crystallized TSA antagonist inside the HDAC receptor pocket.

At first, a validation process was performed for the target receptor by running a redocking process for only the co-crystallized inhibitor, and a low RMSD value indicated the valid performance (RMSD = 0.57) (Figure 6; Davis and Baker, 2009; Alnajjar et al., 2020; Abo Elmaaty et al., 2021).

**FIGURE 7:**
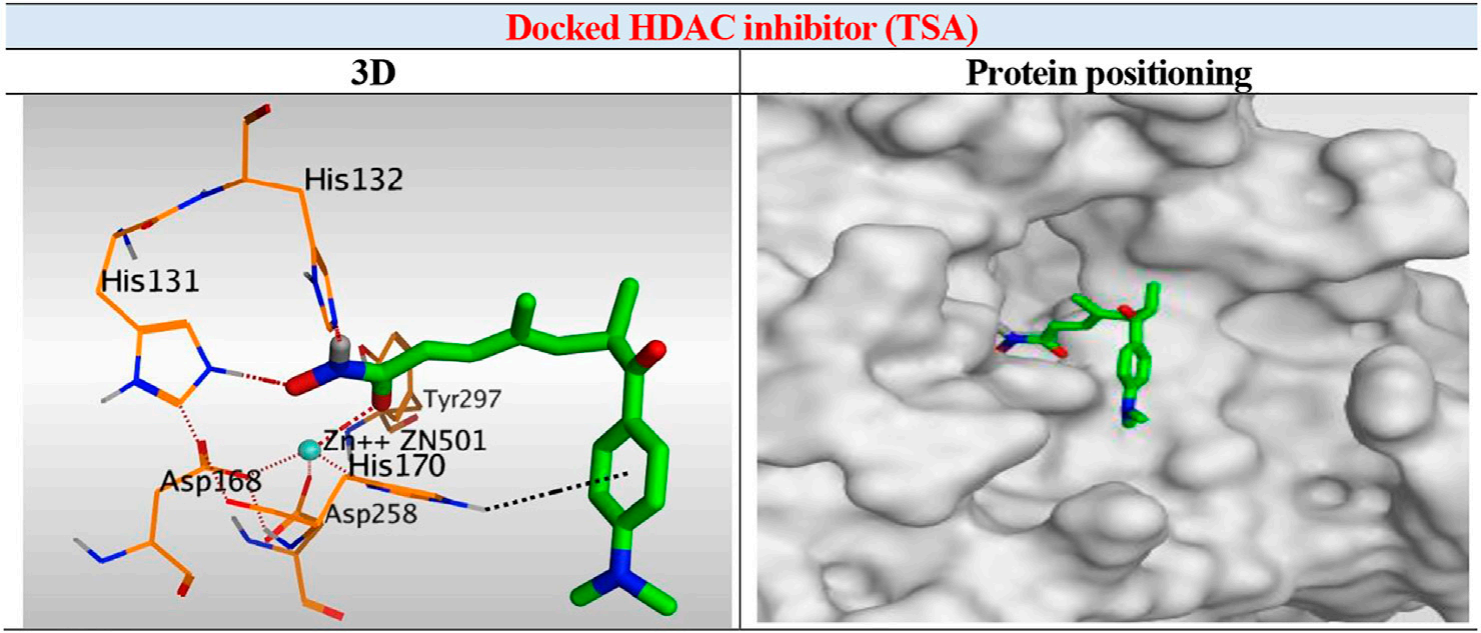
3D representation and positioning for the docked TSA inside the tubular pocket of the HDAC receptor.

The results of docking studies of TSA revealed that it occupied the tube-shaped pocket of the receptor with nearly a fingerprint binding mode compared to the co-crystallized one. It was found that the phenyl ring of TSA is located outside the receptor pocket and fits in the narrow portion of the pocket through its five-carbon-long branched aliphatic chain making multiple van der Waal interactions with the surrounding hydrophobic moieties lining the pocket. The hydroxamic acid group at the end of its aliphatic chain reaches the polar bottom part of the pocket, where it binds the zinc in a bidentate fashion and also contacts the crucial active site residues (His131 and His132). It recorded a binding score of −11.95 kcal/mol and an RMSD of 1.23 Å. It bound Zn^2+^ metal in a similar bidentate manner through its terminal charged and carbonyl oxygen atoms with 1.93 and 2.51 Å, respectively. Moreover, it formed one ionic bond through its charged oxygen atom with His131 with 3.17 Å, and two hydrogen bonds with Tyr297 and His132 with 2.67 and 3 Å through the carbonyl oxygen and amidic NH groups, respectively. Finally, it formed a pi-H interaction through its phenyl group with His170 with 4.74 Å. It was noted that the hydroxamic acid group at the end of the aliphatic chain of TSA fitted the polar bottom part of the pocket, leaving its hydrophobic moiety outside (Figure 7).

Interestingly, the synthesized derivatives were found to form nearly the same binding mode compared to the co-crystallized inhibitor (TSA) with an additional extra positioning of the quinoxaline moiety inside the narrow tubular pocket of HDAC, which provides a more promising fitting as the N1 of quinoxaline was found to be the zinc-binding group.

**FIGURE 8:**
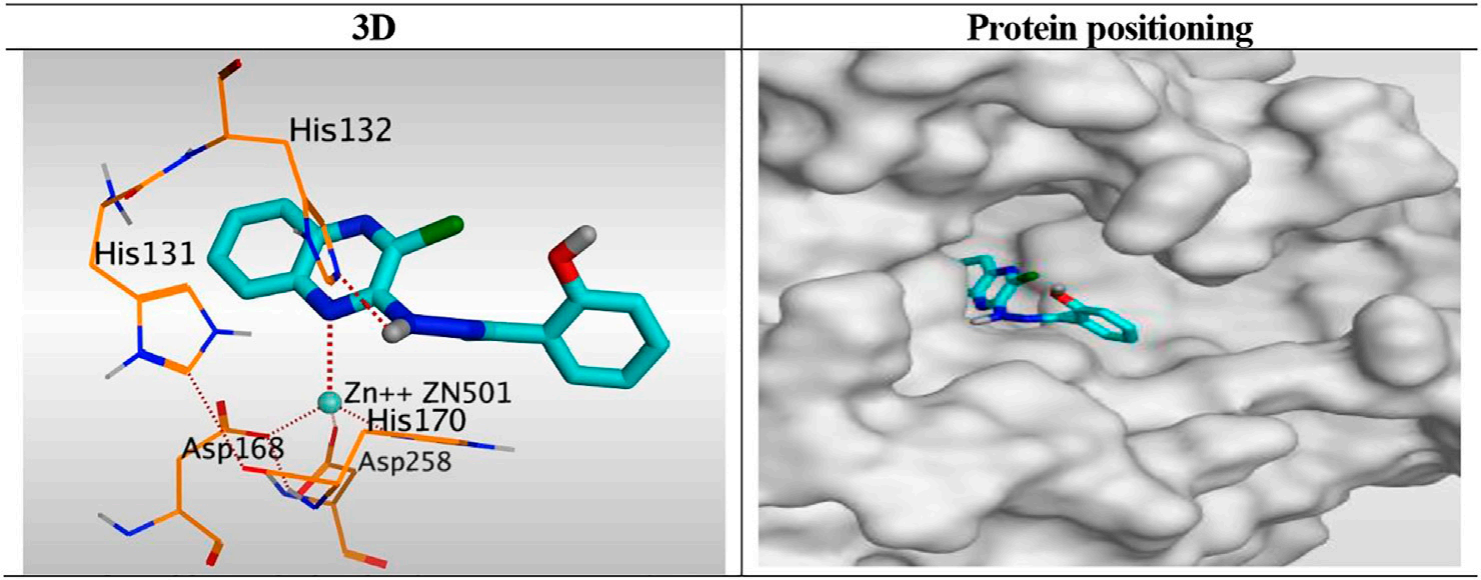
3D representation and positioning for the docked **6**_**k**_ inside the tubular pocket of the HDAC receptor.

Compound **6**_**K,**_ as a representative example, was fitted inside the deep pocket through its quinoxaline ring with a binding score of − 6.46 kcal/mol and RMSD of 0.77 Å. It bound Zn^2+^ metal in a unidentate manner through its N1 atom of quinoxaline moiety with 2.63 Å. Furthermore, it formed a hydrogen bond with His132 at 2.96 Å through its linker NH group of the hydrazide moiety (Figure 8).

**FIGURE 9:**
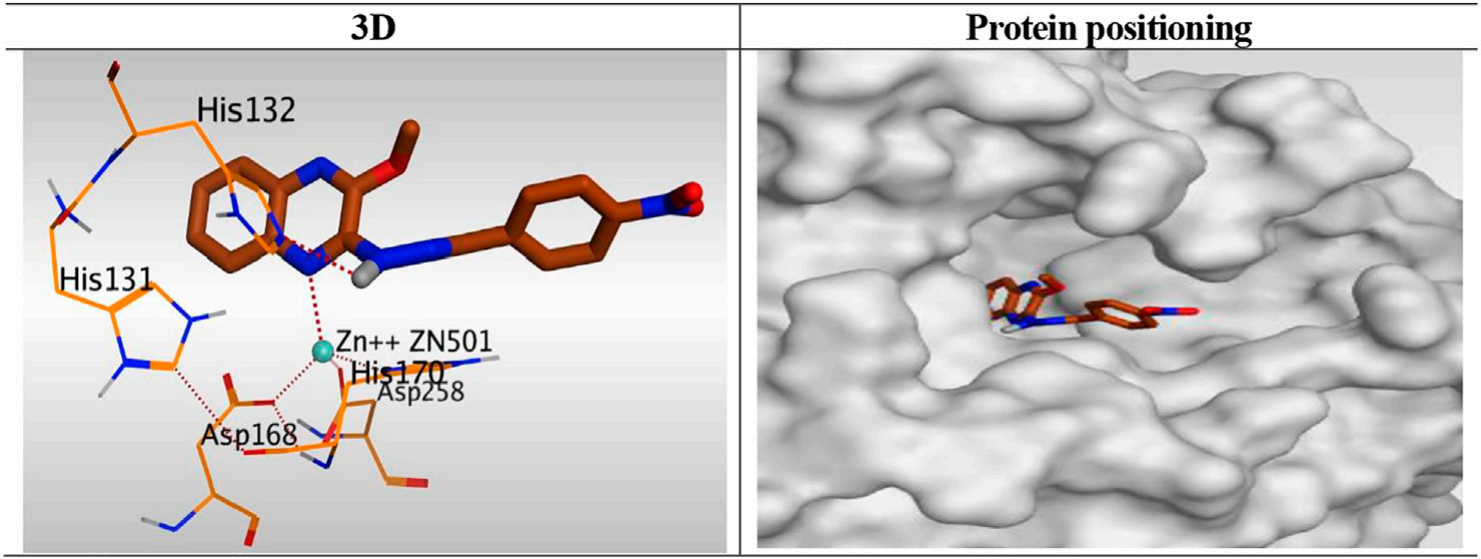
3D representation and positioning for the docked **10**_**h**_ inside the tubular pocket of the HDAC receptor.

Besides, the quinoxaline ring of compound **10**_**h**_ was positioned inside the narrow tube-like pocket of HDAC forming a binding score of 6.45 kcal/mol and RMSD of 1.54 Å. As mentioned before as a general binding mode of our quinoxaline derivatives, it was stabilized in HDAC pocket through the formation of a unidentate bond with Zn^2+^ metal by N1 of quinoxaline ring with 2.58 Å. Moreover, it formed an H-bond with His132 through its NH group of hydrazide linker with 3.12 Å (Figure 9).

#### ADMET Analysis

ADMET studies were carried out for the synthesized compounds, including some descriptors. The predicted descriptors are listed in the Supplementary Table SI1.

ADMET-BBB penetration results revealed that compounds **6**_**i**_ **, 6**_**j**_ **, 10**_**g**_ **,** and **12** have low or very low levels; so that, these compounds were expected to be safe to CNS. The other compounds were predicted to have a very high, high, or medium level of BBB penetration. All the tested compounds showed low to very low range levels of ADMET aqueous solubility.

Intestinal absorption is defined as the percentage absorbed of a compound from the gut wall (Mannhold et al., 2012; Zaki et al., 2021). A well-absorbed compound can penetrate the bloodstream in humans by at least 90% (Klopman et al., 2002). According to ADMET studies, the absorption levels of all compounds appeared in the good range.

**FIGURE 10:**
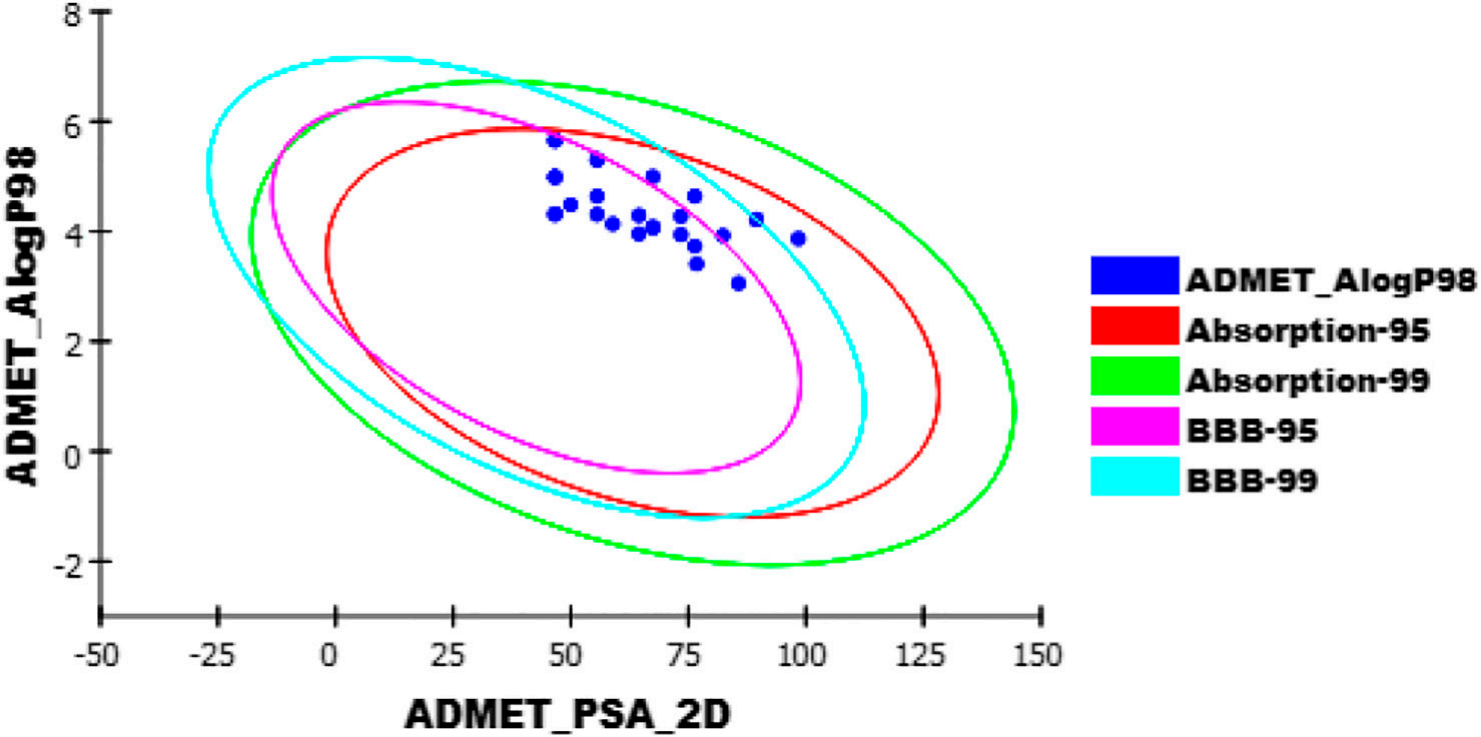
The expected ADMET study for the newly synthesized quinoxaline derivatives.

The cytochrome P450 2D6 (CYP2D6) model predicts CYP2D6 enzyme inhibition using a 2D chemical structure as input. CYP2D6 inhibition experiment is required as part of the regulatory procedures in the drug discovery and development process (Roy and Roy, 2009). All the tested compounds were predicted to be non-inhibitors of CYP2D6 except compounds **6**_**b**_ **, 6**_**c**_ **, 6**_**d**_ **, 6e,** and **10**_**b**_. Consequently, a liver dysfunction side effect is not expected upon administration of these compounds. The plasma protein-binding model predicts whether a compound is likely to be highly bound (≥90% bound) to carrier proteins in the blood (Ghafourian and Amin, 2013). All compounds were expected to bind plasma protein over 90% (Figure 10).

#### Toxicity Studies

Toxicity prediction was carried out for the synthesized compounds based on the validated and constructed models in Discovery Studio software (Xia et al., 2004; Agoni et al., 2020).

It is worth mentioning that most compounds showed *in silico* low adverse effects and toxicity against the tested models. Concerning FDA rodent carcinogenicity, all the tested compounds were predicted to be non-carcinogenic except compounds **8**, **10**_**f**_, and **12** which were single carcinogens. For the carcinogenic potency TD_50_ rat model, the tested compounds showed TD_50_ values ranging from 1.797 to 52.581 mg/kg body weight/day. Regarding the rat maximum tolerated dose model, the compounds showed maximum tolerated dose with a range of 0.082–0.760 g/kg body weight. Additionally, all compounds were non-toxic against the developmental toxicity potential model except compound **6**_**h**_. For the rat oral LD_50_ model, all compounds showed low oral LD_50_ values (from 0.102 to 1.109 mg/kg body weight/day). For the rat chronic LOAEL model, the compounds showed LOAEL values ranging from 0.055 to 0.413 g/kg body weight. Moreover, all compounds were predicted to be mild and non-irritant against ocular irritancy and skin irritancy models, respectively, as represented in Supplementary Table SI2.

### Structure-Activity Relationship Studies

**FIGURE 11:**
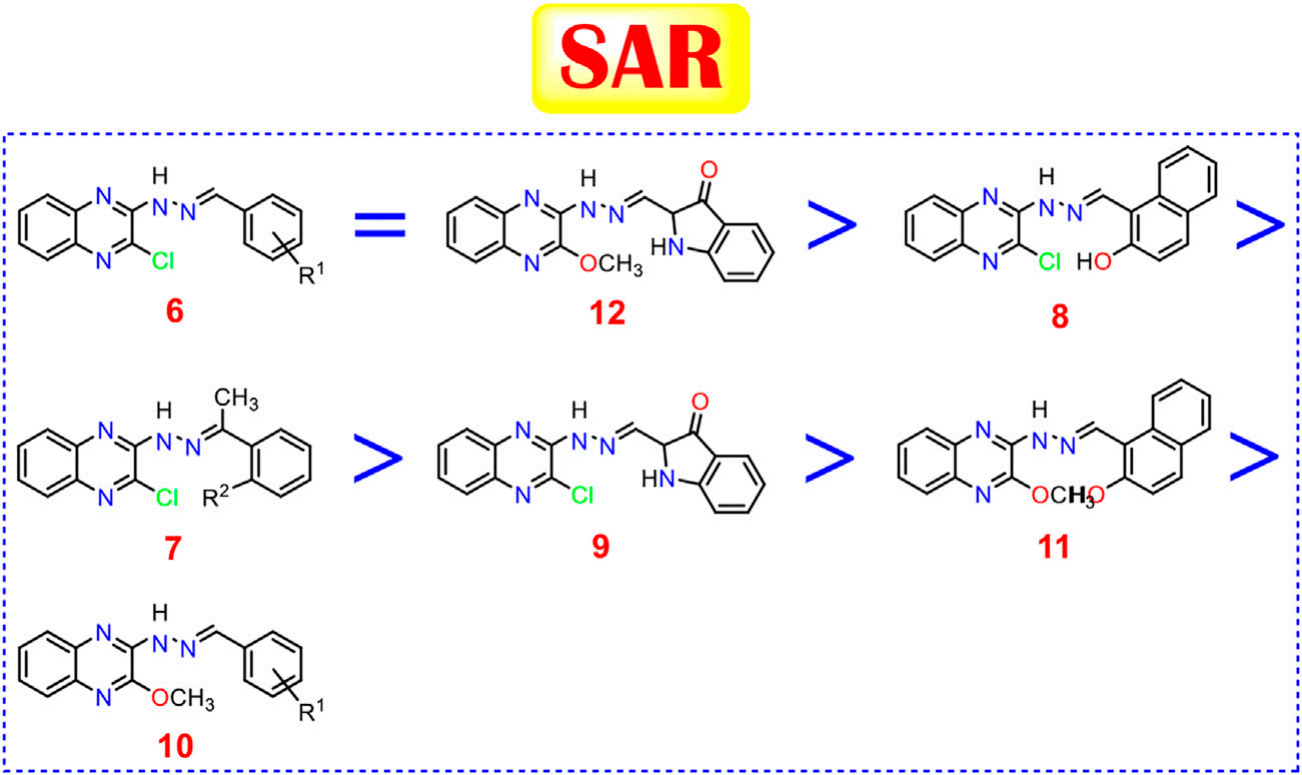
Structure-activity relationships of the newly synthesized quinoxaline candidates as HDACIs.

Studying the structure-activity relationship of our newly synthesized tested quinoxaline candidates according to their IC_50_ values towards liver cancer cell lines (HepG-2 and HuH-7) showed the following interesting results:

Generally, 2-chloro quinoxaline derivatives (**6**) with different substituted benzylidene side chains were found to exert the most promising cytotoxic activityranging from very strong to strong (except for 3-NO_2_ one). Besides, 2-methoxy quinoxaline compound (**12**) with isatin side chain maintains a very strong cytotoxic activity as well. Moreover, 2-chloro quinoxaline derivative with a 2-hydroxy-1-naphthaldehyde side chain (**8**) showed a very strong anticancer activity on both cell lines which exceeds that of some derivatives of (**6**). Furthermore, the introduction of a 2-hydroxy acetophenone side chain (**7**_**b**_) maintains the very strong cytotoxic activity of 2-chloro quinoxaline moiety in contrast to the plain acetophenone derivative (**7**_**a**_) which decreased the cytotoxic effect greatly. Also, the presence of an isatin side chain on 2-chloro quinoxaline (**9**) achieved a strong anti-proliferative effect. On the other hand, the analog of the previously mentioned compound (**8**) with a very strong cytotoxic activity**,** 2-methoxy quinoxaline derivative (**11**), showed only a strong cytotoxic activity. Finally, most of the 2-methoxy quinoxaline derivatives (**10**) compared to their 2-chloro analogs showed weak anti-proliferative activities except those with 2-OH, 4-NO_2_ and 2-OCH_3_ benzylidene side chains which showed very strong, strong, and moderate cytotoxic activities, respectively (Figure 11).

## Conclusion

Twenty-seven new quinoxaline derivatives were synthesized with different substitutions to study their SAR as promising anticancer candidates targeting the HDAC enzymes (HDAC1, HDAC4, and HDAC6 subtypes). Twenty compounds showed cytotoxic effects ranging from very strong to moderate against two liver cancer cell lines (HepG-2 and HuH-7). Then, the most active ten compounds (**6**_**c**_, **6**_**d**_, **6**_**f**_, **6**_**g**_, **6**_**k**_, **6**_**l**_, **7**_**b**_, **8**, **10**_**h**_, and **12**) were further evaluated as HDAC1, HDAC4, and HDAC6 inhibitors and revealed IC_50_ values ranging from 1.39 to 7.21 µM, compared to the reference drug, suberoylanilide hydroxamic acid (SAHA). Moreover, the most active compound **6**_**c**_ with a 4-Cl benzylidene side chain was further evaluated through cell cycle analysis in HepG-2 cell line and showed a significant decline in the cell population at both G0/G1 and S phases with 36.75% (1.23-fold) and 30.32% (1.18-fold), respectively, compared to that of the control which was 45.40 and 35.95%. Furthermore, it was subjected to annexin V-based flow cytometry assay and achieved an increase in the total apoptosis percentage by 9.98-, and 10.81-fold, respectively, compared to the control. Finally, the aforementioned most active ten compounds greatly elevated the level of proapoptotic Bax protein by a range from 6.74- to 11.25-fold increase, compared to the control. On the other hand, they were able to decrease the level of the anti-apoptotic Bcl-2 protein by a range from 1.73- to 3.96-fold decrease, compared to the control. Again, compound **6**_**c**_ among all of the tested derivatives achieved the highest increase in the level of Bax protein (274.3 pg/ml) and the lowest decrease in the level of Bcl-2 protein (1.27 pg/ml), compared to the control (24.37 and 5.04 pg/ml), respectively. Interestingly, docking studies revealed nearly the same binding mode compared to the co-crystallized inhibitor (TSA) with an additional extra positioning of the quinoxaline moiety inside the narrow tubular pocket of HDAC, which may explain the reason behind the previously discussed promising biological results, and confirm our proposed mechanism of action for them as HDACIs. Also, the SAR studies of our diverse synthesized derivatives based on their biological results may give a clear spot on the essential pharmacophoric features required for targeting HDAC as well in the future by medicinal chemists.

## Materials and Methods

### Chemistry

#### General

All melting points were carried out by the open capillary method on a Gallen Kamp apparatus. The infrared spectra were recorded on a Pye Unicam SP 1000 IR spectrophotometer using the potassium bromide disk technique. Proton and carbon magnetic resonance NMR spectra were recorded on a Bruker AVANCE-III 400 MHz-NMR spectrometer. TMS was used as an internal standard, and chemical shifts were measured on a d scale (ppm). The mass spectra were recorded on Varian MAT 311-A (70 e.v.) and Direct Inlet unit (DI-50) of SHIMADZU GC/MSeQP5050A. The reactions were monitored by thin-layer chromatography (TLC) using TLC sheets precoated with UV fluorescent silica gel Merck 60 F254 plates and were visualized using a UV lamp and different solvents as mobile phases. 2,3-(1*H*,4*H*)-Quinoxalinedione **3**, 2,3- dichloroquinoxaline **4**, 2-chloro-3-hydrazinylquinoxaline **5** were obtained according to the reported procedures(Romer, 2009).

#### General Procedure for the Synthesis of Compounds 6_a-l_, 7_a,b_, 8, and 9

Equimolar amounts of compound **5** (0.7 g, 0.002 mol) and appropriate carbonyl derivatives (0.002 mol), namely, benzaldehyde, 2-chlorobenzaldehyde, 4-chlorobenzaldehyde, 2,4-dichlorobenzaldehyde, 2,6-dichlorobenzaldehyde, 2-methoxybbenzaldehyde, 3,4-dimethoxybenzaldehyde, 3,4,5-trimethoxybenzaldehyde, 3-nitrobenzaldehyde, 4-nitrobenzaldehyde, 2-hydroxybenzaldehyde, acetophenone, 2-hydroxyacetophenone, 2-hydroxy-1-naphthaldehyde, and isatin were refluxed in absolute ethanol (25 ml) in the presence of a catalytic amount of glacial acetic acid for the appropriate time, and the reaction was followed up by TLC. The mixture was cooled, and the formed solid product was filtered, dried, and crystallized from ethanol to afford the corresponding compounds **6**_**a-l**_, **7**_**a,b**_, **8**, and **9**, respectively.

##### 2-(2-Benzylidenehydrazinyl)-3-chloroquinoxaline **6**_**a**_

Canary yellow crystal **(**yield, 89%); m. p. = 228–230°C; IR (KBr, cm^1^): 3,265 (NH), 3,056 (CH aromatic), 1,607 (C=N); ^1^H NMR (400 MHz, DMSO-*d*_6_) *δ* 10.99 (s, 1H, NH), 8.62 (s, 1H, NCH), 8.32–7.63 (m, 3H, Ar-H, H2 and H6 of benzene, H8 of quinoxalne), 7.51 (dd, *J* = 14.9, 7.8 Hz, 5H, Ar-H, H5, H6 and H7 of quinoxalne, H3 and H5 of benzene), 7.16 (s, 1H, Ar-H, H4, of benzene); ^13^C NMR (101 MHz, DMSO-*d*_6_) *δ* 156.44 (Ar-C, C3 of quinoxaline), 146.82 (NCH), 140.42 (Ar-C2 of quinoxaline), 135.44 (Ar-C), 133.53 (Ar-C), 130.85(Ar-C), 129.13(Ar-C), 128.78 (Ar-C), 127.37 (Ar-C), 126.02 (Ar-C), 125.31(Ar-C), 123.05 (Ar-C), 115.36 (Ar-C); C_15_H_11_ClN_4_ (282.73).

##### 2-Chloro-3-(2-(2-chlorobenzylidene)hydrazinyl)quinoxaline **6**_**b**_

Bright yellow crystal **(**yield, 83%); m. p. = 224–226°C; IR (KBr, cm^1^): 3,361 (NH), 3,006 (CH aromatic), 1,610 (C=N); ^1^H NMR (400 MHz, DMSO-*d*_6_) *δ* 11.36 (s, 1H, NH), 9.07 (s, 1H, NCH), 8.61 (d, *J* = 5.2 Hz, 1H, Ar-H, H5 of quinoxaline), 8.11 (d, *J* = 7.7 Hz, 1H, Ar-H, H8 of quinoxaline), 7.58 (t, *J* = 7.0 Hz, 1H, Ar-H, H6 of quinoxaline), 7.56–7.49 (m, 2H, Ar-H, H7 of quinoxaline and H6 of benzene), 7.46 (d, *J* = 7.8 Hz, 2H, Ar-H, H3 and H5 of benzene), 7.24–7.12 (m, 1H, H4 of benzene); C_15_H_10_C_l2_N_4_ (317.17). ^13^C NMR (101 MHz, DMSO-*d*_6_) δ 152.16, 145.05, 142.73, 140.94, 134.08, 133.63, 132.84, 131.34, 130.38, 129.27, 127.83, 127.34, 125.60, 123.30, 115.48.

##### 2-Chloro-3-(2-(4-chlorobenzylidene)hydrazinyl)quinoxaline **6**_**c**_

Orang powder **(**yield, 84%); m. p. = 184–186°C; ^1^H NMR (400 MHz, DMSO-*d*_6_) *δ* 10.82 (s, 1H, NH), 8.61 (s, 1H, NCH), 7.87 (d, *J* = 8.2 Hz, 2H, Ar-H, H5 and H8 of quinoxaline), 7.75 (t, *J* = 9.1 Hz, 2H, Ar-H, H6 and H7of quinoxaline), 7.61 (d, *J* = 7.8 Hz, 1H, Ar-H, H2 of benzene), 7.52 (d, *J* = 8.1 Hz, 2H, 2H, Ar-H, H4 and H5 of benzene), 7.40 (d, *J* = 7.7 Hz, 1H, Ar-H, H6 of benzene); ^13^C NMR (101 MHz, DMSO-*d*_6_) ^13^C NMR (101 MHz, DMSO-*d*_6_) *δ* 145.05 (Ar-C, C3 of quinoxaline), 144.83, (NCH), 138.41 (Ar-C, C2 of quinoxaline), 136.33 (Ar-C), 134.96 (Ar-C), 134.08 (Ar-C), 131.06 (Ar-C), 129.55 (Ar-C), 129.29 (Ar-C), 128.06 (Ar-C), 125.40 (Ar-C).; C_15_H_10_C_l2_N_4_ (317.17).

##### 2-Chloro-3-(2-(2,4-dichlorobenzylidene)hydrazinyl)quinoxaline **6**_**d**_

Yellow crystal (yield, 80%); m. p. = 182–184°C; ^1^H NMR (400 MHz, DMSO-*d*_6_) *δ* 11.50 (s, 1H, NH), 9.03 (s, 1H, NCH), 8.12 (d, *J* = 8.5 Hz, 1H, Ar-H, H6 of benzene), 7.89–7.83 (m, 1H, Ar-H, H3 of benzene), 7.75 (d, *J* = 7.3 Hz, 2H, Ar-H, H5 and H8 of quinoxaline), 7.58 (m, *J* = 8.3 Hz, 3H, Ar-H, H6 and H7 of quinoxaline, H5 of benzene); ^13^C NMR (101 MHz, DMSO-*d*_6_) *δ* 145.27 (Ar-C, C3 of quinoxaline), 142.25 (Ar-C, C2 of quinoxaline), 140.93 (NCH), 137.54 (Ar-C), 136.71 (Ar-C), 135.09 (Ar-C), 134.02 (Ar-C), 131.62 (Ar-C), 131.20 (Ar-C), 129.86 (Ar-C), 128.47 (Ar-C), 128.03 (Ar-C), 126.94 (Ar-C).; C_15_H_9_Cl_3_N_4_ (351.62).

##### 2-Chloro-3-(2-(2,6-dichlorobenzylidene)hydrazinyl)quinoxaline **6**_**e**_

Orange crystal (yield, 81%); m. p. = 220–222°C; ^1^H NMR (400 MHz, DMSO-*d*_6_) *δ* 11.50 (s, 1H, NH), 8.83 (s, 1H, NCH), 7.87 (d, *J* = 8.2 Hz, 1H, Ar-H, H5 of quinoxaline), 7.72 (t, *J* = 7.6 Hz, 1H, Ar-H, H8 of quinoxaline), 7.59 (t, *J* = 8.2 Hz, 3H, Ar-H, H6 of quinoxaline, H3 and H5 of benzene), 7.50–7.42 (m, 1H, Ar-H, H4 of benzene); ^13^C NMR (101 MHz, DMSO-*d*_6_) *δ* 13C NMR (101 MHz, DMSO-d6) *δ* 145.37 (Ar-C, C3 of quinoxaline), 142.69 (Ar-C, C3 of quinoxaline), 140.88 (Ar-C, C2 of quinoxaline), 137.58 (NCH), 136.59 (Ar-C), 134.44 (Ar-C), 131.60 (Ar-C), 131.15 (Ar-C), 129.52 (Ar-C), 128.01 (Ar-C), 127.02 (Ar-C); C_15_H_9_C_l3_N_4_ (351.62).

##### 2-Chloro-3-(2-(2-methoxybenzylidene)hydrazinyl)quinoxaline **6**_**f**_

Canary yellow crystal (yield, 88%); m. p. = 210–212°C; IR (KBr, cm^1^): 3,369 (NH), 3,015 (CH aromatic), 2,952 (CH aliphatic), 1,627 (C=N); ^1^H NMR (400 MHz, DMSO-*d*_6_) *δ* 10.95 (s, 1H, NH), 8.82 (s, 1H, NCH), 8.39 (d, *J* = 7.6 Hz, 1H, Ar-H, H5 of quinoxaline), 8.24 (s, 1H, Ar-H, H6 of benzene), 7.51 (d, *J* = 7.4 Hz, 1H, Ar-H, H8 of quinoxaline), 7.46 (t, *J* = 7.7 Hz, 1H, Ar-H, H6 of quinoxaline), 7.32 (d, *J* = 7.4 Hz, 1H, Ar-H, H3 of benzene), 7.13 (t, *J* = 8.5 Hz, 1H, Ar-H, H7 of quinoxaline), 7.07–7.03 (m, 2H, Ar-H, H4 and H5 of benzene), 3.89 (s, 3H, OCH_3_); ^13^C NMR (101 MHz, DMSO-*d*_6_) *δ* 158.51 (Ar-C, C3 of quinoxaline), 151.40 (Ar-C, C2 of benzene), 149.37 , 140.31 (NCH), 133.88 (Ar-C, C2 of quinoxaline), 132.30 (Ar-C), 128.58 (Ar-C), 127.73 (Ar-C), 124.77 (Ar-C), 123.75 (Ar-C), 123.36 (Ar-C), 122.73 (Ar-C), 120.93 (Ar-C), 115.21 (Ar-C), 112.18 (Ar-C), 56.17 (OCH_3_).; MS (*m/z*): 313 (M^+^ + 1, 8.18% %), 308.42 (100%, base beak); C_16_H_13_ClN_4_O (312.76).

##### 2-Chloro-3-(2-(3,4-dimethoxybenzylidene)hydrazinyl)quinoxaline **6**_**g**_

Orange powder (yield, 79%); m. p. = 164–166°C; IR (KBr, cm^1^): 3,292 (NH), 3,053 (CH aromatic), 2,967 (CH aliphatic); ^1^H NMR (400 MHz, DMSO-*d*_6_) *δ* 10.89 (s, 1H, NH), 8.56 (s, 1H, NCH), 7.86–7.66 (m, 2H, Ar-H, H5 and H8 of quinoxaline), 7.66–7.45 (m, 2H, Ar-H, H6 and H7 of quinoxaline), 7.39 (d, *J* = 11.9 Hz, 2H, Ar-H, H5 and H6 of benzene), 7.08 (d, *J* = 8.2 Hz, 1H, Ar-H, H2 of benzene), 3.89 (s, 3H, OCH_3_), 3.84 (s, 3H, OCH_3_); ^13^C NMR (101 MHz, DMSO-*d*_6_) *δ* 151.42 (Ar-C, C3 of quinoxaline), 149.66 (Ar-C, C4 and C3 of benzene), 145.24 (NCH), 144.73 (Ar-C, C2 of quinoxaline), 131.04 (Ar-C), 128.08 (Ar-C), 127.87 (Ar-C), 125.98 (Ar-C), 124.58 (Ar-C), 122.96 (Ar-C), 116.42 (Ar-C), 111.74 (Ar-C), 109.84 (Ar-C), 56.20 (OCH_3_), 56.07 (OCH_3_).; C_16_H1_3_ClN_4_O (312.76).

##### 2-Chloro-3-(2-(3,4,5-trimethoxybenzylidene)hydrazinyl)quinoxaline **6**_**h**_

Orange powder (yield, 75%); m. p. = 210–212°C; IR (KBr, cm^1^): 3,275 (NH), 3,065 (CH aromatic), 2,998 (CH aliphatic), 1,607 (C=N); ^1^H NMR (400 MHz, DMSO-*d*_6_) *δ* 11.2 (s, 1H, NH), 8.60 (s, 1H, NCH), 8.00–7.51 (m, 3H, Ar-H, H5, H8 and H6 of quinoxaline ), 7.52–6.94 (m, 3H, Ar-H, H7of quinoxaline, H2 and H6 of benzene), 3.89 (s, 6H, 2OCH_3_), 3.74 (s, 3H, OCH_3_); MS (*m/z*): 372 (M^+^, 100%); C_18_H_17_ClN_4_O_3_ (372.81); ^13^C NMR (101 MHz, DMSO-*d*_6_) *δ* 153.65 (Ar-C, C3 of quinoxaline), 145.03 (Ar-C, C3 and C5 of benzene), 144.82 (Ar-C, C2 of quinoxaline), 131.05 (Ar-C), 130.66 (Ar-C), 128.10 (Ar-C), 106.03 (Ar-C), 105.46 (Ar-C), 60.65 (OCH_3_), 56.50 (OCH_3_).

##### 2-Chloro-3-(2-(3-nitrobenzylidene)hydrazinyl)quinoxaline **6**_**i**_

Orange crystal (yield, 81%); m. p. = 214–216°C; IR (KBr, cm^1^): 3,260 (NH), 3,066 (CH aromatic), 1,615 (C=N); ^1^H NMR (400 MHz, DMSO-*d*_6_) *δ* 11.47 (s, 1H, NH), 8.75 (s, 1H, Ar-H, H2 of benzene), 8.34 (d, *J* = 7.7 Hz, 3H, NCH, Ar-H, H5 and H6 of benzene) , 8.02 (d, *J* = 8.3 Hz, 1H, Ar-H, H5 of quinoxaline), 7.89 (d, *J* = 8.1 Hz, 1H, Ar-H, H8 of quinoxaline), 7.75 (t, *J* = 7.3 Hz, 1H, Ar-H, H5 of benzene), 7.68–7.20 (m, 2H, Ar-H, H6 and H7 of quinoxaline); C_15_H_10_ClN_5_O_2_ (327.73); ^13^C NMR (101 MHz, DMSO-*d*_6_) *δ* 155.63 (Ar-C, C3 of quinoxaline), 147.95 (Ar-C C3 of benzene), 145.20 (NCH), 141.46 (Ar-C, C2 of quinoxaline), 140.88 (Ar-C), 137.63 (Ar-C) , 136.73 (Ar-C), 131.22 (Ar-C), 129.70 (Ar-C), 128.15 (Ar-C), 127.06 (Ar-C), 124.60 (Ar-C), 115.95 (Ar-C).

##### 2-Chloro-3-(2-(4-nitrobenzylidene)hydrazinyl)quinoxaline **6**_**j**_

Yellow crystal (yield, 80%); m. p. = 231–232°C; IR (KBr, cm^1^): 3,308 )NH), 3,100 (CH aromatic), 1,615 (C=N^1^H NMR (400 MHz, DMSO-*d*_6_) *δ* 11.40 (s, 1H, NH), 8.77 (s, 1H, NCH), 8.58 (m, 1H, Ar-H, H4 of benzene), 8.37–8.26 (m, 1H, Ar-H, H5 of benzene), 8.19 (m, 1H, Ar-H, H2 of benzene), 7.96–7.85 (m, 1H, Ar-H, H6 of benzene), 7.84–7.70 (m, 2H, Ar-H, H5 and H8 of quinoxaline), 7.68–7.14 (m, 2H, Ar-H, H6 and H7 of quinoxaline); ^13^C NMR (101 MHz, DMSO-*d*_6_) *δ* 155.83 (Ar-C, C3 of quinoxaline), 148.78 (Ar-C C4 of benzene), 145.34 (NCH), 140.92 (Ar-C, C2 of quinoxaline), 137.54 (Ar-C), 136.96 (Ar-C), 134.95 (Ar-C), 133.67 (Ar-C), 131.22 (Ar-C), 128.03 (Ar-C), 127.05 (Ar-C), 124.41 (Ar-C), 121.17 (Ar-C).; C_15_H_10_ClN_5_O_2_ (327.73).

##### 2-((2-(3-Chloroquinoxalin-2-yl)hydrazono)methyl)phenol **6**_**k**_

Yellow crystal (yield, 79%); m. p. = 225–227°C; IR (KBr, cm^1^): 3,337 (NH), 3,055 (CH aromatic), 1,618 (C=N); ^1^H NMR (400 MHz, DMSO-*d*_6_) *δ* 11.74 (s, 1H, OH), 11.51 (s, 1H, NH), 8.82 (s, 1H, NCH), 8.06–7.38 (m, 5H, Ar-H, H5, H6, H7 and H8 of quinoxaline, H6 of benzene), 7.33 (t, *J* = 7.7 Hz, 1H, H4 of benzene), 7.04–6.89 (m, 2H, H3 and H5 of benzene); ^13^C NMR (101 MHz, DMSO-*d*_6_) *δ* 157.92 (Ar-C, C3 of quinoxaline), 144.88 (Ar-C, C2 of benzene), 131.66 (Ar-C), 131.23 (Ar-C), 130.28 (Ar-C), 128.07 (Ar-C), 126.39 (Ar-C), 126.12 (Ar-C), 119.79 (Ar-C), 119.44 (Ar-C), 116.96 (Ar-C); C_15_H_11_ClN_4_O (298.73).

##### 4-((2-(3-Chloroquinoxalin-2-yl)hydrazono)methyl)-N,N-dimethylaniline **6**_**l**_

Yellow crystal (yield, 82%); m. p. = 202–204°C; IR (KBr, cm^1^): 3,386 (OH), 3,239 (NH), 3,050 (CH aromatic), 2,892 (CH aliphatic), 1,610 (C=N); ^1^H NMR (400 MHz, DMSO-*d*_6_) *δ* 11.00 (s, 1H, NH), 8.51 (s, 1H, NCH), 7.87 (d, *J* = 8.6 Hz, 1H, Ar-H, H5 of quinoxaline), 7.81 (dd, *J* = 7.7, 5.1 Hz, 1H, Ar-H, H8 of quinoxaline), 7.60 (d, *J* = 8.7 Hz, 2H, Ar-H, H3 and H5 of benzene), 7.55–7.47 (m, 1H, Ar-H, H6 of quinoxaline), 7.14 (t, *J* = 7.6 Hz, 1H, Ar-H, H7 of quinoxaline), 6.81 (d, *J* = 3.0 Hz, 1H, Ar-H, H2 of benzene), 6.79 (d, *J* = 3.1 Hz, 1H, Ar-H, H6 of benzene), 3.03 (s, 3H, NCH_3_), 3.01 (s, 3H, NCH_3_).; C_17_H_16_ClN_5_ (325.80); ^13^C NMR (101 MHz, DMSO-*d*_6_) *δ* 158.56 (Ar-C, C3 of quinoxaline), 152.44 (Ar-C, C4 of benzene), 148.99 (NCH), 145.46 (Ar-C, C2 of quinoxaline), 141.38 (Ar-C), 136.73 (Ar-C), 133.09 (Ar-C), 130.66 (Ar-C), 128.87 (Ar-C), 128.08 (Ar-C), 126.61 (Ar-C), 122.37 (Ar-C), 115.44 (Ar-C), 111.97 (Ar-C), 41.30 (NCH_3_).

##### 2-Chloro-3-(2-(1-phenylethylidene)hydrazinyl)quinoxaline **7**_**a**_

Dark red crystal (yield, 74%); m. p. = 272–274°C; IR (KBr, cm^1^): 3,424 (NH), 3,020 (CH aromatic), 1,603 (C=N); ^1^H NMR (400 MHz, DMSO-*d*_6_) *δ* 11.10 (s, 1H, NH), 8.29 (d, *J* = 29.0 Hz, 3H, Ar-H, H5 and H8 of quinoxaline, H2 of benzene), 8.02 (d, *J* = 8.2 Hz, 1H, Ar-H, H6 of benzene), 7.76–7.61 (m, 1H, Ar-H, H6 of quinoxaline), 7.56–7.42 (m, 1H, Ar-H, H7 of quinoxaline), 7.40–6.98 (m, 3H, Ar-H, H3 , H4 and H5 of benzene), 2.67 (s, 3H, CCH_3_); *MS* (*m/z*): 298 (M^+^ + 1, 28.08%), 297 (87.40%), 296 (100%, base beak); C_16_H_13_ClN_4_ (296.76); ^13^C NMR (101 MHz, DMSO-*d*_6_) *δ* 139.10 (Ar-C, C3 of quinoxaline), 132.64 (NHC), 131.50 (Ar-C, C2 of quinoxaline), 130.87 (Ar-C), 130.00 (Ar-C), 129.30 (Ar-C), 126.25 (Ar-C), 126.00 (Ar-C), 119.92 (Ar-C), 115.60 (Ar-C), 26.94 (CCH_3_).

##### 2-(1-(2-(3-Chloroquinoxalin-2-yl)hydrazono)ethyl)phenol **7**_**b**_

Yellow crystal (yield, 80%); m. p. = 248–250°C; ^1^H NMR (400 MHz, DMSO-*d*_6_) *δ* 13.33 (s, 1H, OH), 9.97 (s, 1H, NH), 7.69 (t, *J* = 7.0 Hz, 1H, Ar-H, H4 of benzene), 7.48 (d, *J* = 7.2 Hz, 1H, Ar-H, H5 of quinoxaline), 7.41 (d, *J* = 7.0 Hz, 1H, Ar-H, H8 of quinoxaline), 7.32 (dd, *J* = 14.3, 7.1 Hz, Ar-H, 1H, H4 of benzene), 7.23–7.12 (m, 1H, Ar-H, H6 of quinoxaline), 7.06–6.79 (m, 3H, Ar-H, H7 of quinoxaline, H6 of benzene), 2.69 (s, 3H, CCH_3_); ^13^C NMR (101 MHz, DMSO-*d*_6_) *δ* 166.14 (Ar-C, C3 of quinoxaline), 159.12 (Ar-C, C2 of benzene), 144.94 (NCH), 137.10 (Ar-C, C2 of quinoxaline), 133.04(Ar-C), 131.35(Ar-C), 129.52(Ar-C), 128.64 (Ar-C), 126.28 (Ar-C), 125.89 (Ar-C), 123.32 (Ar-C), 120.09 (Ar-C), 119.09 (Ar-C), 117.78 (Ar-C), 115.76 (Ar-C), 15.60 (CCH_3_). MS (*m/z*): 313 (M^+^ + 1, 10.2%), 312 (M^+^, 21.90%), 205 (100%, base beak); C_16_H_13_ClN_4_O (312.76).

##### 1-((2-(3-Chloroquinoxalin-2-yl)hydrazono)methyl)naphthalen-2-ol **8**

Yellow crystal (yield, 72%); m. p. = 218–220°C; IR (KBr, cm^1^): 3,342 (NH), 3,058 (CH aromatic), 1,656 (C=N); ^1^H NMR (400 MHz, DMSO-*d*_6_) *δ* 13.18 (s, 1H, OH), 11.58 (s, 1H, NH), 9.76 (s, 1H, NCH), 8.20 (d, *J* = 8.6 Hz, 1H, Ar-H, H8 of naphthalene), 8.01–7.86 (m, 3H, Ar-H, H5 and H8 of quinoxaline, H4 of naphthalene), 7.78 (dt, *J* = 15.2, 8.2 Hz, 2H, Ar-H, H6 and H7 of quinoxaline), 7.65 (t, *J* = 7.7 Hz, 1H, Ar-H, H5 of naphthalene), 7.59 (t, *J* = 7.5 Hz, 1H, Ar-H, H7 of naphthalene), 7.44 (t, *J* = 7.5 Hz, 1H, Ar-H, H6 of naphthalene), 7.29 (d, *J* = 8.9 Hz, 1H, Ar-H, H3 of naphthalene); C_19_H_13_ClN_4_O (348.79); ^13^C NMR (101 MHz, DMSO-d6) *δ* 158.19 (Ar-C, C3 of quinoxaline), 146.46 (Ar-C, C2 of naphthalene), 144.82 (Ar-C, C2 of quinoxaline), 140.94 (NCH), 137.49 (Ar-C), 136.52 (Ar-C), 132.80 (Ar-C), 132.09 (Ar-C), 131.33(Ar-C), 129.47(Ar-C), 128.16 (Ar-C), 126.88 (Ar-C), 124.01 (Ar-C), 120.87 (Ar-C), 119.56 (Ar-C), 109.33 (Ar-C).

##### 2-(2-(3-Chloroquinoxalin-2-yl)hydrazono)indolein-3-one **9**

Red powder (yield, 89%); m. p. > 300°C; IR (KBr, cm^1^): 3,171 )NH), 3,062 (CH aromatic), 1715 (C=O), 1,616 (C=N); ^1^H NMR (400 MHz, DMSO-*d*_6_) *δ* 12.35 (s, 1H, NH indole), 10.72 (s, 1H, NH), 8.52 (d, *J* = 7.6 Hz, 1H, Ar-H, H4 of indole), 7.86 (d, *J* = 8.2 Hz, 1H, Ar-H, H5 of quinoxaline), 7.71 (d, *J* = 7.9 Hz, 1H, Ar-H, H8 of quinoxaline), 7.58 (t, *J* = 7.7 Hz, 1H, Ar-H, H6 of quinoxaline), 7.37 (dq, *J* = 17.4, 8.0 Hz, 2H, Ar-H, H5 and H6 of indole), 7.07 (t, *J* = 7.5 Hz, 1H, Ar-H, H7 of quinoxaline), 6.91 (d, *J* = 7.7 Hz, 1H, Ar-H, H7 of indole); ^13^C NMR (101 MHz, DMSO-d6) *δ* 165.85 (Ar-C, C3 of indole), 148.07 (Ar-C, C3 of quinoxaline), 146.52 (Ar-C, C2 of indole), 144.26 (Ar-C, C2 of quinoxaline), 133.23 (Ar-C), 131.99 (Ar-C), 131.55 (Ar-C), 128.34 (Ar-C), 124.58 (Ar-C), 122.52 (Ar-C), 118.02 (Ar-C), 117.08 (Ar-C), 110.69 (Ar-C). C_16_H_10_ClN_5_O (323.74).

#### General Procedure for the Synthesis of Compounds **10**_**a-i**_, **11**, and **12**

An equimolar of compounds **6**_**c-h**_, **6**_**j-l**_ **, 8**, and **9** (0.01 mol) and sodium methoxide (0.02 mol) were refluxed in methanol (25 ml) for the appropriate time, and the reaction was followed up by TLC. The mixture was cooled then poured into water (50 ml). The formed precipitated was filtered, dried, and crystallized in ethanol to afford the corresponding compounds **10**, **11**, and **12**, respectively.

##### 2-(2-(4-Chlorobenzylidene)hydrazinyl)-3-methoxyquinoxaline **10**_**a**_

Red orange powder (yield, 73%); m. p. = 184–186°C; ^1^H NMR (400 MHz, DMSO-*d*_6_) *δ* 11.16 (s, 1H, NH), 8.54 (s, 1H, NCH), 7.94–7.74 (m, 2H, Ar-H, H2 and H6 of benzene), 7.68 (m, Hz, 2H, 2H, Ar-H, H5 and H8 of quinoxaline), 7.53 (d, *J* = 8.1 Hz, 2H, 2H, Ar-H, H3 and H5 of benzene), 7.49–7.20 (m, 2H, 2H, Ar-H, H6 and H7 of quinoxaline), 4.11 (s, 3H, OCH_3_); ^13^C NMR (101 MHz, DMSO-d6) *δ* 134.33 (Ar-C), 129.34 (Ar-C), 127.19 (Ar-C), 126.49 (Ar-C), 54.44 (OCH_3_); C_16_H_13_ClN_4_O (312.76).

##### 2-(2-(2,4-Dichlorobenzylidene)hydrazinyl)-3-methoxyquinoxaline **10**_**b**_

Yellow crystal (yield, 85%); m. p. = 232–234°C ^1^H NMR (400 MHz, DMSO-*d*_6_) *δ* 11.42 (s, 1H, NH), 8.85 (s, 1H, NCH), 8.16 (d, *J* = 8.4 Hz, 1H, Ar-H, H5 of quinoxline), 7.71 (s, 1H, Ar-H, H3 of benzene), 7.64 (dd, *J* = 12.7, 8.0 Hz, 2H, Ar-H, H5 and H6 of benzene), 7.54 (d, *J* = 8.6 Hz, 1H, Ar-H, H8 of qunoxaline), 7.43 (t, *J* = 7.4 Hz, 1H, Ar-H, H6 of quinoxline), 7.34 (t, *J* = 6.9 Hz, 1H, Ar-H, H7 of quinoxline), 4.10 (s, 3H, OCH_3_); ^13^C NMR (101 MHz, DMSO-d6) *δ* 129.73 (Ar-C), 128.33 (Ar-C), 126.46 (Ar-C), 54.40 (OCH_3_); MS (*m/z*): 346 (M^+^ - 1, 5.21%), 331 (100%, base beak); C_16_H_12_C_l2_N_4_O (347.20).

##### 2-(2-(2,6-Dichlorobenzylidene)hydrazinyl)-3-methoxyquinoxaline **10**_**c**_

Faint yellow crystal (yield, 88%); m. p. = 240–242°C; ^1^H NMR (400 MHz, DMSO-*d*_6_) *δ* 11.39 (s, 1H, NH), 8.70 (s, 1H, NCH), 7.65 (t, *J* = 7.9 Hz, 2H, Ar-H, H6, H7 of quinoxline), 7.59 (d, *J* = 8.0 Hz, 2H, Ar-H, H5 and H8 of quinoxline), 7.45 (t, *J* = 8.3 Hz, 2H, Ar-H, H3 and H5 of benzene), 7.39 (t, *J* = 7.3 Hz, 1H, Ar-H, H4 of benzene), 4.13 (s, 3H, OCH_3_); ^13^C NMR (101 MHz, DMSO-*d*_6_) *δ* 141.35 (Ar-C, C2 of quinoxline), 136.41 (NCH), 134.41 (Ar-C, C3 of quinoxline), 131.86 (Ar-C), 131.36 (Ar-C), 129.45 (Ar-C), 128.06 (Ar-C), 127.20 (Ar-C), 126.50 (Ar-C), 125.27 (Ar-C), 54.49 (OCH_3_); C_16_H_12_C_l2_N_4_O (347.20).

##### 2-Methoxy-3-(2-(2-methoxybenzylidene)hydrazinyl)quinoxaline **10**_**d**_

Orange yellow powder (yield, 89%); m. p. = 225–227°C; IR (KBr, cm^1^): 3,449 (NH), 3,021 (CH aromatic), 2,935 (CH aliphatic), 1,656 (C=N); ^1^H NMR (400 MHz, DMSO-*d*_6_) *δ* 9.04 (s, 1H, NH), 8.66 (s, 1H, NCH), 8.12 (d, *J* = 7.1 Hz, 1H, Ar-H, H5 of quinoxline), 7.95 (d, *J* = 8.1 Hz, 1H, Ar-H, H8 of quinoxline), 7.84 (d, *J* = 7.3 Hz, 1H, Ar-H, H3 of benzene), 7.73 (t, *J* = 7.5 Hz, 1H, Ar-H, H6 of quinoxline), 7.55 (d, *J* = 7.3 Hz, 1H, Ar-H, H6 of benzene), 7.45 (t, *J* = 7.6 Hz, 1H, Ar-H, H7 of quinoxline), 7.14 (d, *J* = 8.3 Hz, 1H, Ar-H, H5 of benzene), 7.08 (t, *J* = 7.4 Hz, 1H, Ar-H, H4 of benzene), 3.93 (s, 3H, OCH_3_ of quinoxaline), 3.79 (s, 3H, OCH_3_ of benzene); MS (*m/z*): 308 (M^+^, 26.23%), 91 (100%, base beak); C_17_H_16_N_4_O_2_ (308.34); ^13^C NMR (101 MHz, DMSO-*d*_6_) *δ* 158.05 (Ar-C, C2 of quinoxline), 137.69 (NCH), 131.39 (Ar-C, C3 of quinoxline), 129.43 (Ar-C), 126.32 (Ar-C), 125.97 (Ar-C), 123.03 (Ar-C), 122.62 (Ar-C), 121.24 (Ar-C), 112.32 (Ar-C), 56.22 (OCH_3_).

##### 2-(2-(3,4-Dimethoxybenzylidene)hydrazinyl)-3-methoxyquinoxaline **10**_**e**_

Faint yellow crystal (yield, 81%); m. p. = 235–237°C; IR (KBr, cm^1^): 3,419 (NH), 3,060 (CH aromatic), 2,941 (CH aliphatic), 1,661 (C=N); ^1^H NMR (400 MHz, DMSO-*d*_6_) *δ* 12.95 (s, 1H, NH), 8.65 (s, 1H, NCH), 8.01 (t, *J* = 7.6 Hz, 1H, Ar-H, H5 of quinoxaline), 7.85–7.69 (m, 2H, Ar-H, H6 and H8 of quinoxaline), 7.54 (t, *J* = 7.5 Hz, 1H, Ar-H, H7 of quinoxaline), 7.46 (t, *J* = 8.0 Hz, 1H, Ar-H, H6 and H2 of benzene), 7.38 (d, *J* = 7.7 Hz, 1H, Ar-H, H6 of benzene), 7.10 (t, *J* = 8.8 Hz, 1H, Ar-H, H5 of benzene), 4.17 (s, 3H, OCH_3_), 3.92 (s, 3H, OCH_3_ of benzene), 3.85 (s, 3H, OCH_3_ of benzene); ^13^C NMR (101 MHz, DMSO-d6) *δ* 149.69 (Ar-C, C2 of quinoxline), 149.60 (NCH), 145.19 (Ar-C, C3 of quinoxline), 128.02 (Ar-C), 127.59 (Ar-C), 127.25 (Ar-C), 126.48 (Ar-C), 126.03 (Ar-C), 124.52 (Ar-C), 123.92 (Ar-C), 123.75 (Ar-C), 111.88 (Ar-C), 110.33 (Ar-C), 109.96 (Ar-C), 56.35 (OCH_3_), 56.26 (OCH_3_), 56.13 (OCH_3_); C_18_H_18_N_4_O_3_ (338.37).

##### 2-Methoxy-3-(2-(3,4,5-trimethoxybenzylidene)hydrazinyl)quinoxaline 10_f_

Faint yellow crystal (yield, 88%); m. p. = 256–258°C; IR (KBr, cm^1^): 3,421 (NH), 3,025 (CH aromatic), 2,940(CH aliphatic), 1,657 (C=N); ^1^H NMR (400 MHz, DMSO-*d*_6_) *δ* 10.96 (s, 1H, NH), 8.43 (s, 1H, NCH), 7.67–7.50 (m, 2H, Ar-H, H5 and H8 of quinoxaline), 7.38 (t, *J* = 6.4 Hz, 1H, Ar-H, H6 of quinoxaline), 7.31–7.20 (m, 1H, Ar-H, H7 of quinoxaline), 7.09 (s, 2H, Ar-H, H2 and H6 of benzene), 4.07 (s, 3H, OCH_3_ of quinoxaline), 3.88 (s, 6H, OCH_3_ of benzene), 3.72 (s, 3H. OCH_3_); ^13^C NMR (101 MHz, DMSO-d6) *δ* 153.62 (Ar-C, C2 of quinoxline), 126.7 (Ar-C), 126.33 (Ar-C), 106.45 (Ar-C), 104.72 (Ar-C), 60.62 (OCH_3_), 56.45 (OCH_3_). 54.14 (OCH_3_); C_19_H_20_N_4_O_4_ (368.39).

##### 2-Methoxy-3-(2-(4-nitrobenzylidene)hydrazinyl)quinoxaline **10**_**g**_

Yellow crystal **(**yield, 84%); m. p. = 228–230°C; IR (KBr, cm^1^): 3,310 )NH), 3,079 (CH aromatic), 2,991 (CH aliphatic), 1,623 (C=N); ^1^H NMR (400 MHz, DMSO-*d*_6_) *δ* 11.38 (s, 1H, NH), 8.68 (s, 1H, NCH), 8.58 (d, *J* = 8.0 Hz, 2H, Ar-H, H3 and H5 of benzene), 8.27 (d, *J* = 8.0 Hz, 2H, Ar-H, H2 and H6 of benzene), 7.88–7.73 (m, 2H, Ar-H, H5 and H8 of quinoxaline), 7.45 (d, *J* = 24.2 Hz, 2H, Ar-H, H6 and H7 of quinoxaline), 4.14 (s, 3H, OCH_3_); ^13^C NMR (101 MHz, DMSO-d6) *δ* 148.77 (Ar-C, C4 of benzene), 143.70 (Ar-C, C2 of quinoxline), 137.24 (NCH), 133.47 (Ar-C), 130.92 (Ar-C), 127.28 (Ar-C), 126.54 (Ar-C), 124.66 (Ar-C), 123.63 (Ar-C), 123.33 (Ar-C), 54.53 (OCH_3_). C_16_H_13_N_5_O_3_ (323.31).

##### 2-((2-(3-Methoxyquinoxalin-2-yl)hydrazono)methyl)phenol 10_h_

Yellow crystal (yield, 81%); m. p. = 247–249°C; ^1^H NMR (400 MHz, DMSO-*d*_6_) *δ* 12.91 (s, 1H, OH), 11.43 (s, 1H, NH), 8.89 (s, 1H, NCH), 7.83 (d, *J* = 7.8 Hz, 1H, Ar-H, H5 of quinoxaline), 7.76 (d, *J* = 6.7 Hz, 1H, Ar-H, H8 of quinoxaline), 7.71 (d, *J* = 7.8 Hz, 1H, Ar-H, H3 of benzene), 7.53 (t, *J* = 7.4 Hz, 1H, Ar-H, H6 of quinoxaline), 7.45 (t, *J* = 7.3 Hz, 1H, Ar-H, H7 of quinoxaline), 7.34 (q, *J* = 8.3, 7.2 Hz, 1H, Ar-H, H6 of benzene), 7.03 (t, *J* = 7.8 Hz, 1H, Ar-H, H4 of benzene), 6.94 (t, *J* = 7.0 Hz, 1H, Ar-H, H5 of benzene), 4.15 (s, 3H, OCH_3_); C_16_H_14_N_4_O_2_ (294.31).

##### 4-((2-(3-Methoxyquinoxalin-2-yl)hydrazono)methyl)-N,N-dimethylaniline *10*_*i*_

Yellow crystal (yield, 86%); m. p. = 214–216°C; IR (KBr, cm^1^): 3,185 (NH), 3,042 (CH aromatic), 2,941 (CH aliphatic), 1,616 (C=N); ^1^H NMR (400 MHz, DMSO-*d*_6_) *δ* 10.77 (s, 1H, NH), 8.41 (s, 1H, NCH), 7.70–7.52 (m, 4H, Ar-H, H5 and H8 of quinoxaline, H2 and H6 of benzene), 7.40 (s, 1H. Ar-H, H6 of quinoxaline), 7.28 (s, 1H, Ar-H, H7 of quinoxaline), 6.79 (d, *J* = 8.4 Hz, 2H, H3 and H5 of benzene), 4.08 (s, 3H, OCH_3_), 3.00 (s, 6H, NCH_3_); ^13^C NMR (101 MHz, DMSO-*d*_6_) *δ* 152.14 (Ar-C, C2 of quinoxaline), 141.00 (Ar-C, C4 of benzene), 133.22 (Ar-C), 129.49 (Ar-C), 128.06 (Ar-C), 127.03 (Ar-C), 126.39 (Ar-C), 122.77 (Ar-C), 112.27 (Ar-C), 54.35 (OCH_3_), 41.57 (NH_3_); C_18_H_19_N_5_O (321.38).

##### 1-((2-(3-Methoxyquinoxalin-2-yl)hydrazono)methyl)naphthalen-2-ol **11**

Orange powder (yield, 89%); m. p. = 153–155°C; IR (KBr, cm^1^): 3,423 (NH), 3,053 (CH aromatic), 2,941 (CH aliphatic), 1,663 (C=N); ^1^H NMR (400 MHz, DMSO-*d*_6_) *δ* 12.71 (s, 1H, OH), 9.67 (s, 1H, NH), 8.29 (s, 1H, NCH), 8.05–7.77 (m, 3H, Ar-H, H5 and H8 of quinoxaline, H3 of naphthalene), 7.73 (d, *J* = 7.6 Hz, 1H, Ar-H, H3 of naphthalene), 7.64 (t, *J* = 7.1 Hz, 1H, Ar-H, H6 of quinoxaline), 7.53 (t, *J* = 7.4 Hz, 1H, Ar-H, H7 of quinoxaline), 7.45 (q, *J* = 7.6 Hz, 2H, Ar-H, H5 and H8 of quinoxaline), 7.29 (d, *J* = 11.3 Hz, 2H, Ar-H, H6 and H7 of quinoxaline), 4.19 (s, 3H, OCH_3_); ^13^C NMR (101 MHz, DMSO-*d*_6_) *δ* 158.18 (Ar-C, C3 of naphthalene), 140.39 (Ar-C, C2 of qunoxaline) , 132.03 (Ar-C, C3 of qunoxaline), 129.44, 128.44 (Ar-C), 128.19 (Ar-C), 127.54 (Ar-C), 126.68 (Ar-C), 125.87 (Ar-C), 124.34 (Ar-C), 124.00 (Ar-C), 121.24 (Ar-C), 119.47 (Ar-C), 116.00 (Ar-C), 109.52 (Ar-C), 54.74 (OCH_3_).; MS (*m/z*): 345 (M^+^ +1, 15.61%), 344 (M^+^, 21.63%), 332 (100%, base beak); C_20_H_16_N_4_O_2_ (344.37).

##### 2-(2-(3-Methoxyquinoxalin-2-yl)hydrazono)indolein-3-one **12**

Yellow crystal (yield, 87%); m. p. = 242–244°C; ^1^H NMR (400 MHz, DMSO-*d*_6_) *δ* 12.17 (s, 1H, NH of indole), 10.75 (s, 1H, NH of quinoxaline), 8.49 (d, *J* = 7.2 Hz, 1H, Ar-H, H5 of quinoxaline), 7.85 (d, *J* = 8.1 Hz, 1H, Ar-H, H8 of quinoxaline), 7.70 (d, *J* = 7.9 Hz, 1H, Ar-H, H4 of indole), 7.58 (t, *J* = 7.3 Hz, 1H, Ar-H, H6 of quinoxaline), 7.35 (q, *J* = 7.6 Hz, 2H, Ar-H, H7 of quinoxaline, H7 of indole), 7.06 (d, *J* = 7.4 Hz, 1H, Ar-H, H5 of indole), 6.91 (d, *J* = 7.7 Hz, 1H, Ar-H, H6 of indole), 4.13 (s, 3H, OCH_3_); C_17_H_13_N_5_O_2_ (319.32).

### Biological Evaluation

Experimental protocols applied for our newly synthesized compounds in the different biological assays were provided in detail in the Supplementary Material.

#### Anti-proliferative Activities Against Human Liver Cancer Cell Lines

The new quinoxaline derivatives of the five series: **6**, **7**, **8**, **9**, and **10** were evaluated for their potential anti-proliferative activity against two liver cancer cell lines (HepG-2 and HuH-7) obtained from the American Type Culture Collection. Cytotoxicity was assessed following the SRB colorimetric assay protocol (Skehan et al., 1990), as reported earlier (Eldehna et al., 2016; Sabt et al., 2018; Ghanem et al., 2020).

#### Histone Deacetylase Inhibitory Activities

The new quinoxaline analogs (**6**_**c**_, **6**_**d**_, **6**_**f**_, **6**_**g**_, **6**_**k**_, **6**_**l**_, **7**_**b**_, **8**, **10**_**h**_, and **12**) were further assayed for their deacetylase enzymes inhibitory activities (HDAC1, HDAC4, and HDAC6 subtypes) based on a homogeneous fluorescence release assay, as discussed before (Fournel et al., 2008).

#### Cell Cycle Analysis

The aforementioned most active compound **6**_**c**_ incorporating 4-Cl side chain was further evaluated through cell cycle analysis in HepG-2 cell line at IC_50_ = 1.53 µM, using BD FACS Calibur flow cytometer, as described previously (Eliaa et al., 2020; Sabt et al., 2020).

#### Annexin V-FITC Apoptosis Assay

Furthermore, our most active candidate **6**_**c**_ was assayed for apoptosis induction using the FITC Annexin-V/PI kit (Becton Dickenson, Franklin Lakes, NJ) following the manufacture’s protocol. The previous compound was analyzed by FACS as we previously described (Al-Rashood et al., 2020; Eliaa et al., 2020; Eldehna et al., 2021).

#### Apoptotic and Anti-Apoptotic Marker Levels (Bcl-2 and Bax)

Quantitative real-time PCR to evaluate the effects of compounds **6**_**c**_, **6**_**d**_, **6**_**f**_, **6**_**g**_, **6**_**k**_, **6**_**l**_, **7**_**b**_, **8**, **10**_**h**_, and **12** on two important target genes (Bcl-2 and Bax) and the housekeeping gene (GAPDH) in HepG-2 cells was performed as well. The method was performed in detail as previously explained (Eliaa et al., 2020).

### *In silico* Studies

#### Docking Studies

A molecular docking study of the newly synthesized quinoxaline derivatives at the histone deacetylase (HDAC) receptor was performed, and the co-crystallized inhibitor, trichostatin A (TSA), was used as a reference standard. Using MOE 2019.0102 drug design software (Inc, 2016), the binding mode of the compound against histone deacetylase (ID: 1C3R) was predicted (Finnin et al., 1999). The crystal structure of the target receptor (HDAC) was downloaded from Protein Data Bank (http://www.rcsb.org/, PDB code: 1C3R, resolution of 2.00 Å) (Finnin et al., 1999). The protein structure was prepared for docking studies by the default method (Alnajjar et al., 2020; Soltan et al., 2021; Soltane et al., 2021; Zaki et al., 2020). The deacetylase and deacetylase-TSA structures show an active site consisting of a tubular pocket, a zinc-binding site (which is the metal cofactor required for HDAC activity), and two Asp-His charge-relay systems, and explain the mechanism of HDAC inhibition (Finnin et al., 1999). Validation of the docking procedure was carried out by applying the docking process for the co-crystallized ligand (Elmaaty et al., 2021a; Elmaaty et al., 2021b; Kandeil et al., 2021). All of the newly synthesized quinoxaline derivatives were prepared and imported in the same database together with the co-crystallized inhibitor (TSA) and generally docked. After completion of the docking process, the obtained poses for each were carefully studied, and the ones having the best scores and binding modes with the protein pocket residues were selected.

#### ADMET Studies

ADMET descriptors (absorption, distribution, metabolism, excretion, and toxicity) of the synthesized compounds were determined using Discovery studio 4.0. i) Blood-brain barrier penetration predicts blood-brain barrier penetration of a molecule. ii) Intestinal absorption predicts human intestinal absorption (HIA) after oral administration. iii) Aqueous solubility predicts the solubility of each compound in the water at 25°C. iv) CYP2D6 binding predicts cytochrome P450 2D6 enzyme inhibition. v) Plasma protein binding predicts the fraction of drug bound to plasma proteins in the blood (Van De Waterbeemd and Gifford, 2003). Discovery studio 4.0 was used to predict ADMET descriptors for all compounds. At first, the CHARMM force field was applied, and then, the compounds were prepared and minimized according to the preparation of small molecule protocol (Al-Karmalawy et al., 2021; Al-Karmalawy and Eissa, 2021). Then, ADMET descriptors protocol was applied to carry out these studies (Ibrahim et al., 2017; El-Gamal et al., 2018; El-Zahabi et al., 2019; El-Shershaby et al., 2021a).

#### Toxicity Studies

The toxicity parameters of the synthesized compounds were calculated using Discovery studio 4.0. At first, the CHARMM force field was applied, and then, the compounds were prepared and minimized according to the preparation of small molecule protocol. Then, different parameters were calculated from the toxicity prediction (extensible) protocol as follows: i) FDA rodent carcinogenicity which computes the probability of a submitted chemical structure being a carcinogen, ii) carcinogenic potency TD_50_ which predicts the tumorigenic dose rate 50 (TD_50_) of a chemical in a rodent chronic exposure toxicity test of carcinogenic potency (Venkatapathy et al., 2009), iii) rat maximum tolerated dose which predicts the rat maximum tolerated dose (MTD) of a chemical (4,5) (Goodrnan and Wilson, 1992), iv) developmental toxicity potential which predicts whether a particular compound is likely to be toxic in a developmental toxicity potential assessment (Agency, 1991; Louisse et al., 2015), v) rat oral LD_50_ which predicts the rat oral acute median lethal dose (LD_50_) in the toxicity test of a chemical (Gonella Diaza et al., 2015), vi) rat chronic LOAEL which predicts the rat chronic lowest observed adverse effect level (LOAEL) value of a chemical (Venkatapathy et al., 2004; Benfenati, 2016), vii) ocular irritancy which predicts whether a particular compound is likely to be an ocular irritant and how severe the irritation is in the Draize test (Macfarlane et al., 2009), viii) skin irritancy predicts whether a particular compound is likely to be a skin irritant and how severe it is in a rabbit skin irritancy test (Macfarlane et al., 2009).

## Data Availability

The original contributions presented in the study are included in the article/Supplementary Material, and further inquiries can be directed to the corresponding authors.
