# Supplementary material for: Design and Synthesis of New Quinoxaline Derivatives as Potential Histone Deacetylase Inhibitors Targeting Hepatocellular Carcinoma: In Silico, In Vitro, and SAR Studies
Source: Front Chem. 2021 Sep 22;9:725135. doi: 10.3389/fchem.2021.725135 (PMC8493129; doi:10.3389/fchem.2021.725135)
Supplement: Supplementary file 1 [file DataSheet1.PDF]

Design and Synthesis of New Quinoxaline Derivatives as Potential Histone Deacetylase Inhibitors Targeting Hepatocellular Carcinoma: *In Silico, In Vitro,* and SAR Studies

Chao Ma[a], Mohammed S. Taghour[b]*, Amany Belal [c], Ahmed B.M. Mehany[d]*, Naglaa Mostafa[e], Ahmed Nabeeh [d], Ibrahim. H. Eissa[b], Ahmed A. Al-Karmalawy[f]*

[a] Hepatobiliary and Pancreatic Surgery, Cancer Hospital of Zhengzhou University, Zhengzhou City, Henan Province, 450000, China.

[b] Pharmaceutical Medicinal Chemistry & Drug Design Department, Faculty of Pharmacy (Boys), Al-Azhar University, Cairo11884, Egypt.

[c] Department of Pharmaceutical Chemistry, College of Pharmacy, Taif University, P.O. Box 11099, Taif 21944, Saudi Arabia.

[d] Zoology Department, Faculty of Science (Boys), Al-Azhar University, Cairo11884, Egypt.

[e] Biophysics Department, Faculty of Women for Arts, Science and Education, Ain Shams University.

[f] Department of Pharmaceutical Medicinal Chemistry, Faculty of Pharmacy, Horus University-Egypt, New Damietta 34518, Egypt.

***Corresponding authors:**

Mohammed S. Taghour: Pharmaceutical Medicinal Chemistry & Drug Design Department, Faculty of Pharmacy (Boys), Al-Azhar University, Cairo 11884, Egypt. **Email:** Mohammad1533.el@azhar.edu.eg

Ahmed B.M. Mehany: Zoology Department, Faculty of Science (Boy) Al-Azhar University, Cairo 11884, Egypt. **Email:** abelal_81@azhar.edu.eg

Ahmed A. Al-Karmalawy: Department of Pharmaceutical Medicinal Chemistry, Faculty of Pharmacy, Horus University-Egypt, New Damietta 34518, Egypt. **Email:** Akarmalawy@horus.edu.eg

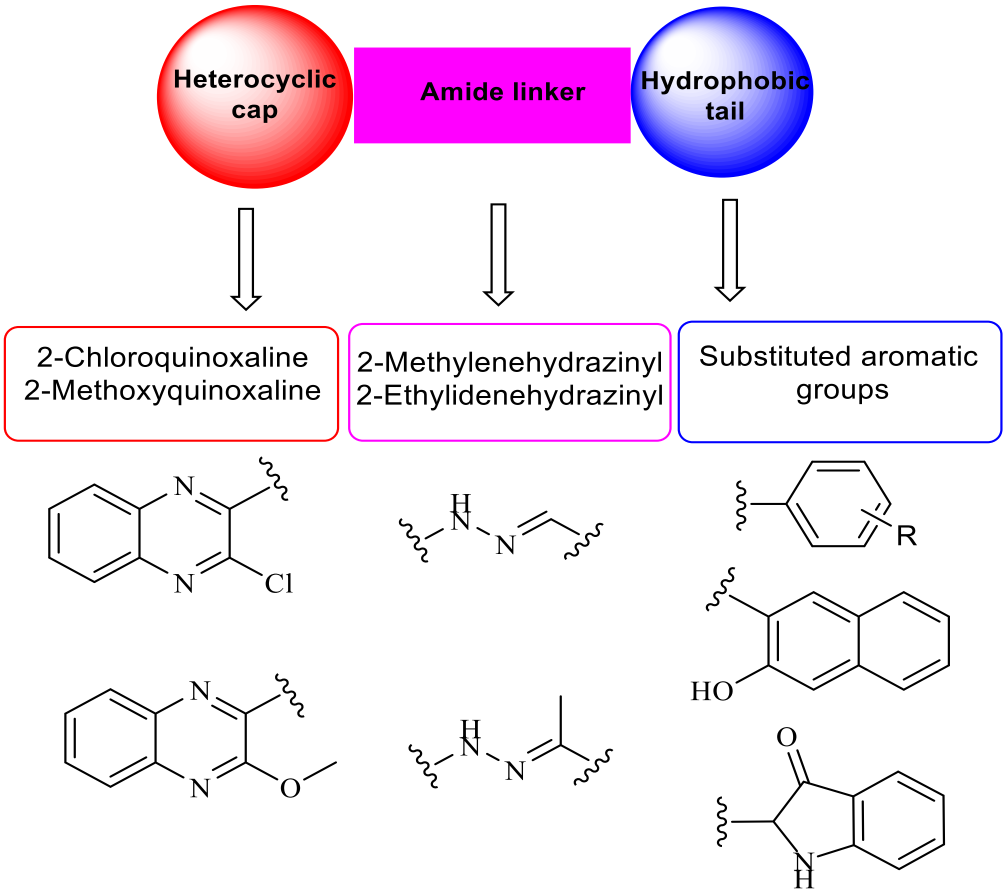

Fig. SI 1: Summary for the possible modifications of HDACIs.

Table SI 1: Predicted ADMET for the synthesized compounds.

**Comp.**	**BBB level [a]**	**Solubility level [b]**	**Absorption level [c]**	**CYP2D6 prediction[d]**	**PPB prediction[e]**
**6_a**	1	2	0	FALSE	TRUE
**6_b**	1	1	0	TRUE	TRUE
**6_c**	1	1	0	TRUE	TRUE
**6_d**	0	1	0	TRUE	TRUE
**6_e**	0	1	0	TRUE	TRUE
**6_f**	1	2	0	FALSE	TRUE
**6_g**	1	2	0	FALSE	TRUE
**6_h**	1	2	0	FALSE	TRUE
**6_i**	4	2	0	FALSE	TRUE
**6_j**	4	2	0	FALSE	TRUE
**6_k**	1	2	0	FALSE	TRUE
**6_l**	1	2	0	FALSE	TRUE
**7_a**	1	2	0	FALSE	TRUE
**7_b**	1	2	0	FALSE	TRUE
**8**	1	1	0	FALSE	TRUE
**9**	2	2	0	FALSE	TRUE
**10_a**	1	2	0	FALSE	TRUE
**10_b**	1	1	0	TRUE	TRUE
**10_c**	1	1	0	FALSE	TRUE
**10_d**	1	2	0	FALSE	TRUE
**10_e**	2	2	0	FALSE	TRUE
**10_f**	2	2	0	FALSE	TRUE
**10_g**	4	2	0	FALSE	TRUE
**10_h**	2	2	0	FALSE	TRUE
**10_i**	1	2	0	FALSE	TRUE

**11**	1	1	0	FALSE	TRUE
**12**	3	2	0	FALSE	TRUE

[a] BBB level, blood brain barrier level, 0 = very high, 1 = high, 2 = medium, 3 = low, 4 = very low.

[b] Solubility level, 1 = very low, 2 = low, 3 = good, 4 = optimal.

[c] Absorption level, 0 = good, 1 = moderate, 2 = poor, 3 = very poor.

[d] CYP2D6, cytochrome P2D6, TRUE = inhibitor, FALSE = non inhibitor. The classification of whether a compound is a CYP2D6 inhibitor using the cutoff Bayesian score of 0.161.

[e] PBB, plasma protein binding, FALSE means less than 90%, TRUE means more than 90%. The classification of whether a compound is highly bounded (>= 90% bound) to plasma proteins using the cutoff Bayesian score of -2.209.

Table SI 2: *In silico* toxicity properties of the newly synthesized quinoxaline derivatives.

Comp.	FDA Rodent Carcinogenicity (Male, mouse)	Carcinogenic Potency TD_{50} (Rat) [a]	Rat Maximum Tolerated Dose (Feed) [b]	Developmental Toxicity Potential	Rat Oral LD_{50} [b]	Rat Chronic LOAEL [b]	Ocular Irritancy (Rat)	Skin Irritancy (Rat)
**6_a**	Non-Carcinogen	23.461	0.226	Non-Toxic	0.102	0.105	Mild	None
**6_b**	Non-Carcinogen	9.251	0.189	Non-Toxic	0.146	0.105	Mild	None
**6_c**	Non-Carcinogen	8.900	0.189	Non-Toxic	0.091	0.082	Moderate	None
**6_d**	Non-Carcinogen	8.317	0.156	Non-Toxic	0.090	0.080	Moderate	None
**6_e**	Non-Carcinogen	6.546	0.156	Non-Toxic	0.109	0.055	Mild	None
**6_f**	Non-Carcinogen	2.395	0.110	Non-Toxic	0.090	0.057	Mild	None
**6_g**	Non-Carcinogen	5.615	0.108	Non-Toxic	0.381	0.107	Mild	Mild
**6_h**	Non-Carcinogen	2.541	0.106	Toxic	0.114	0.058	Mild	Mild
**6_i**	Non-Carcinogen	4.976	0.174	Non-Toxic	0.191	0.076	Moderate	Mild
**6_j**	Non-Carcinogen	4.976	0.174	Non-Toxic	0.204	0.081	Moderate	Mild
**6_k**	Non-Carcinogen	52.581	0.760	Non-Toxic	0.121	0.161	Moderate	None
**6_l**	Non-Carcinogen	5.233	0.127	Non-Toxic	0.116	0.082	Moderate	Mild
**7_a**	Non-Carcinogen	20.699	0.210	Non-Toxic	0.126	0.188	Mild	None
**7_b**	Non-Carcinogen	46.272	0.706	Non-Toxic	0.157	0.413	Mild	None
**8**	Single-Carcinogen	12.431	0.744	Non-Toxic	0.119	0.070	Mild	None
**9**	Single-Carcinogen	11.559	0.353	Non-Toxic	0.314	0.200	Mild	None
**10_a**	Non-Carcinogen	2.440	0.134	Non-Toxic	0.166	0.081	Moderate	None
**10_b**	Non-Carcinogen	2.284	0.111	Non-Toxic	0.164	0.080	Moderate	None
**10_c**	Non-Carcinogen	1.797	0.111	Toxic	0.199	0.054	Mild	None
**10_d**	Non-Carcinogen	3.391	0.092	Non-Toxic	0.189	0.172	Mild	None
**10_e**	Non-Carcinogen	7.958	0.090	Toxic	1.109	0.158	Mild	Mild

**10_f**	Single-Carcinogen	3.606	0.088	Toxic	0.303	0.102	Mild	Mild
**10_g**	Non-Carcinogen	1.948	0.082	Non-Toxic	0.423	0.072	Mild	Mild
**10_h**	Non-Carcinogen	20.559	0.358	Toxic	0.298	0.271	Mild	None
**10_i**	Non-Carcinogen	10.040	0.089	Non-Toxic	0.206	0.131	Moderate	Mild
**11**	Non-Carcinogen	4.871	0.351	Toxic	0.294	0.118	Mild	None
**12**	Single-Carcinogen	4.525	0.166	Non-Toxic	0.422	0.301	Mild	None

[a] Unit: mg/kg body weight/day.

[b] Unit: g/kg body weight.

IR of comp. 6a

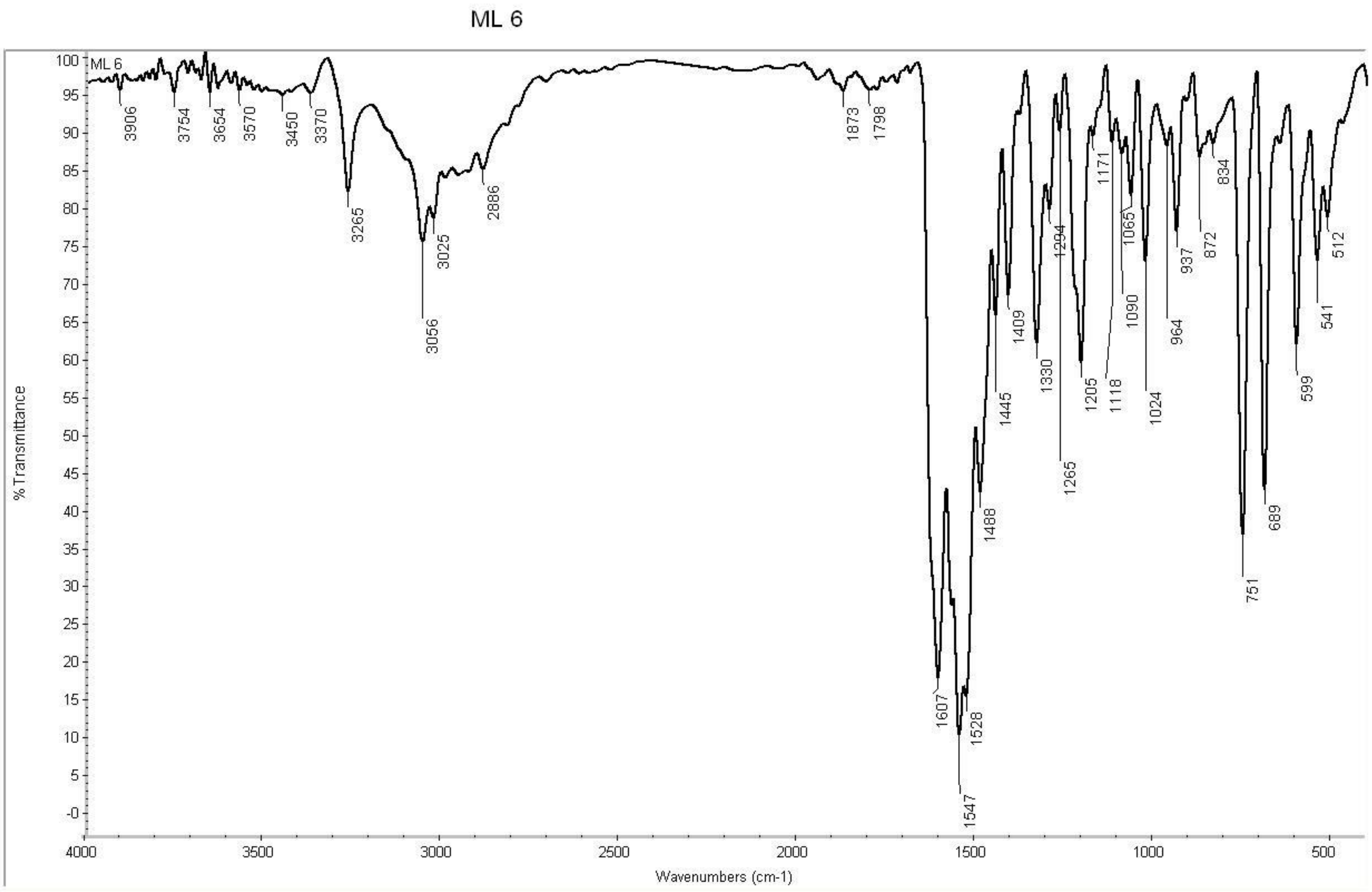
ML 6
ML 6
% Transmittance
Wavenumbers (cm-1)
4000
3500
3000
2500
2000
1500
1000
500
100
95
90
85
80
75
70
65
60
55
50
45
40
35
30
25
20
15
10
5
-0
3906
3754
3654
3570
3450
3370
3265
3056
3025
2886
1873
1798
1607
1547
1528
1488
1445
1409
1330
1294
1265
1205
1171
1118
1090
1065
1024
964
937
872
834
751
689
599
541
512

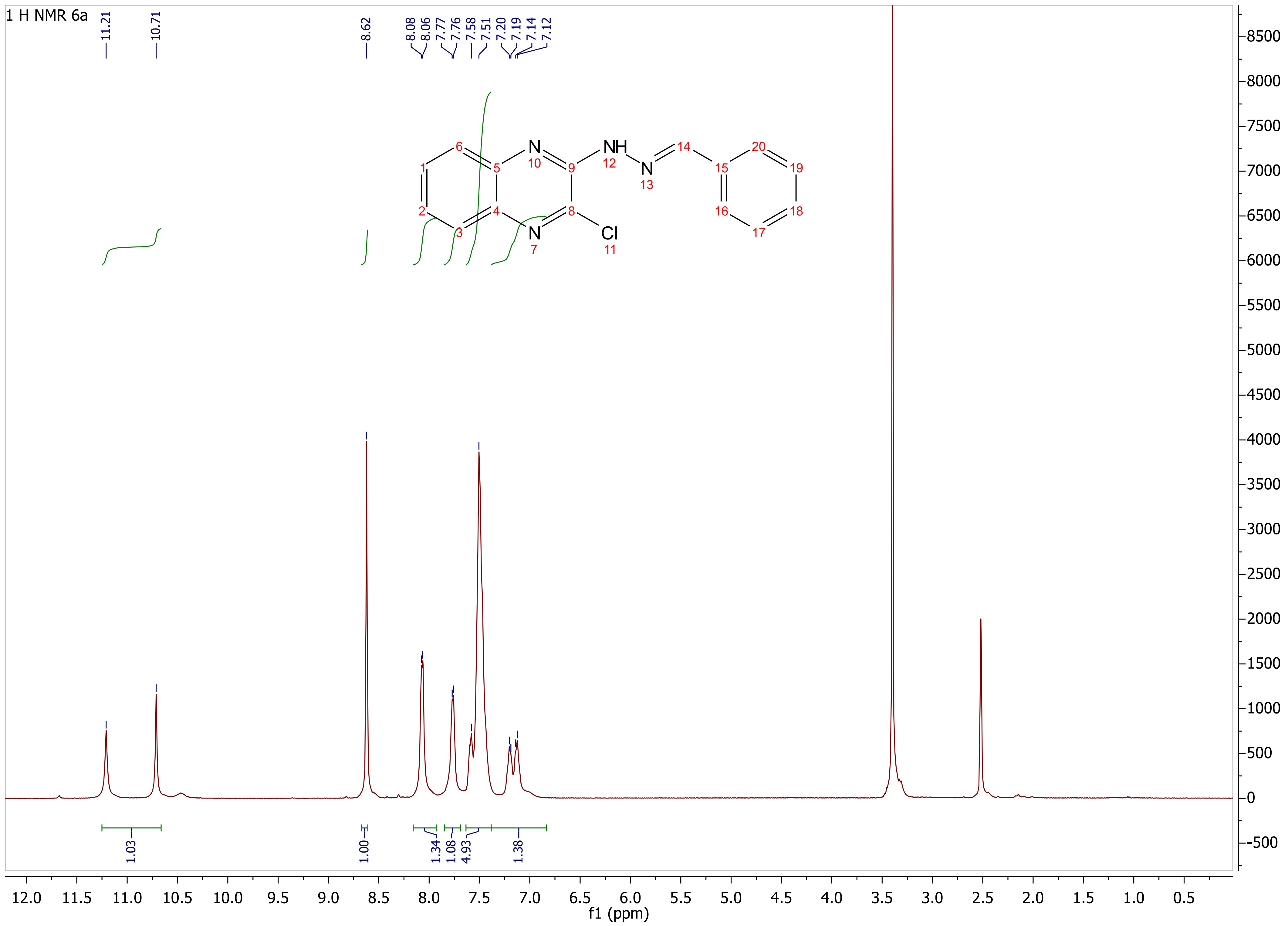
1 H NMR 6a
11.21
10.71
8.62
8.08
8.06
7.77
7.76
7.58
7.51
7.20
7.19
7.14
7.12
N
NH
N
N
Cl
1.03
1.00
1.34
1.08
4.93
1.38
f1 (ppm)

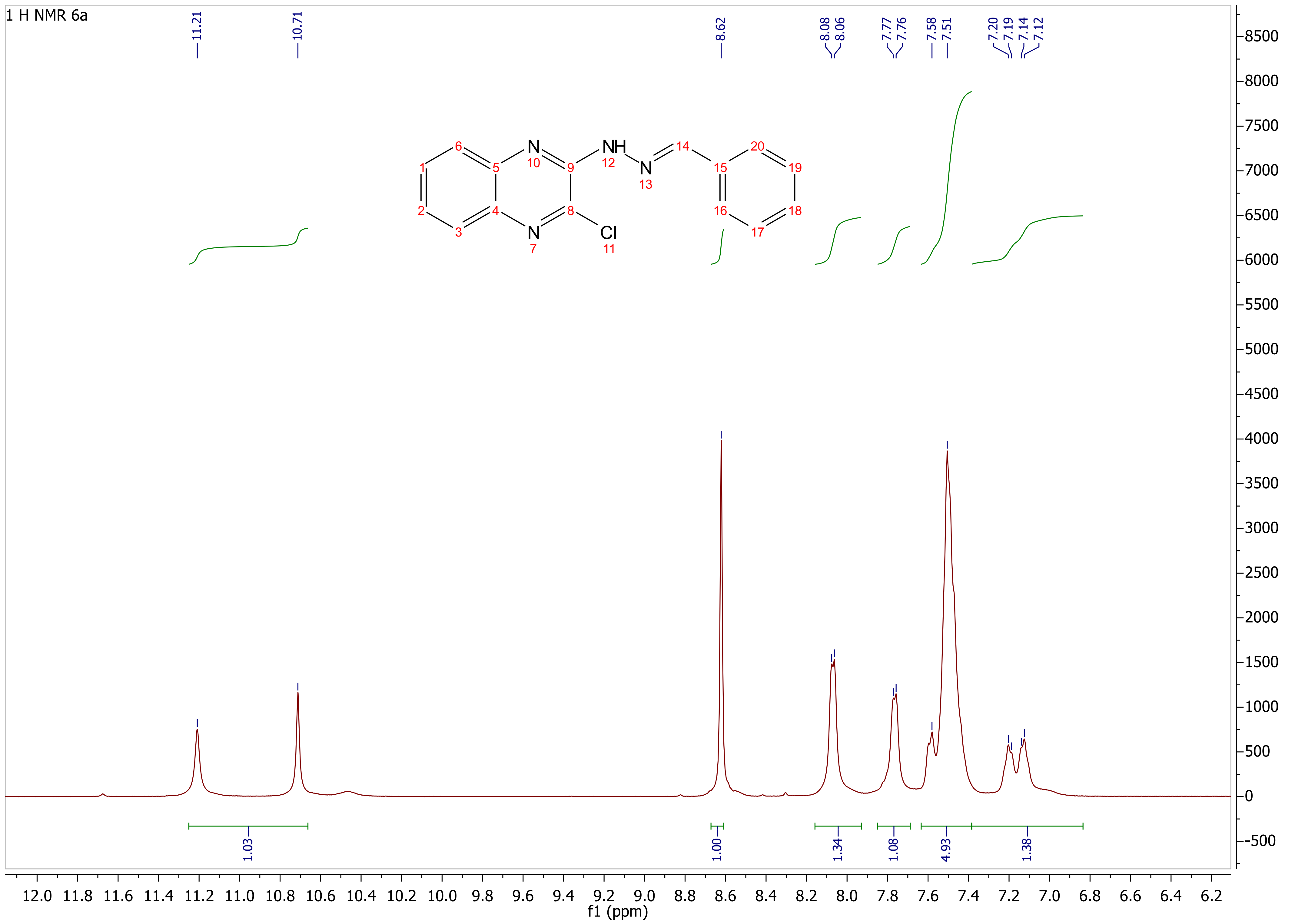
1 H NMR 6a
11.21
10.71
8.62
8.08
8.06
7.77
7.76
7.58
7.51
7.20
7.19
7.14
7.12
N
NH
N
N
Cl
1.03
1.00
1.34
1.08
4.93
1.38
f1 (ppm)

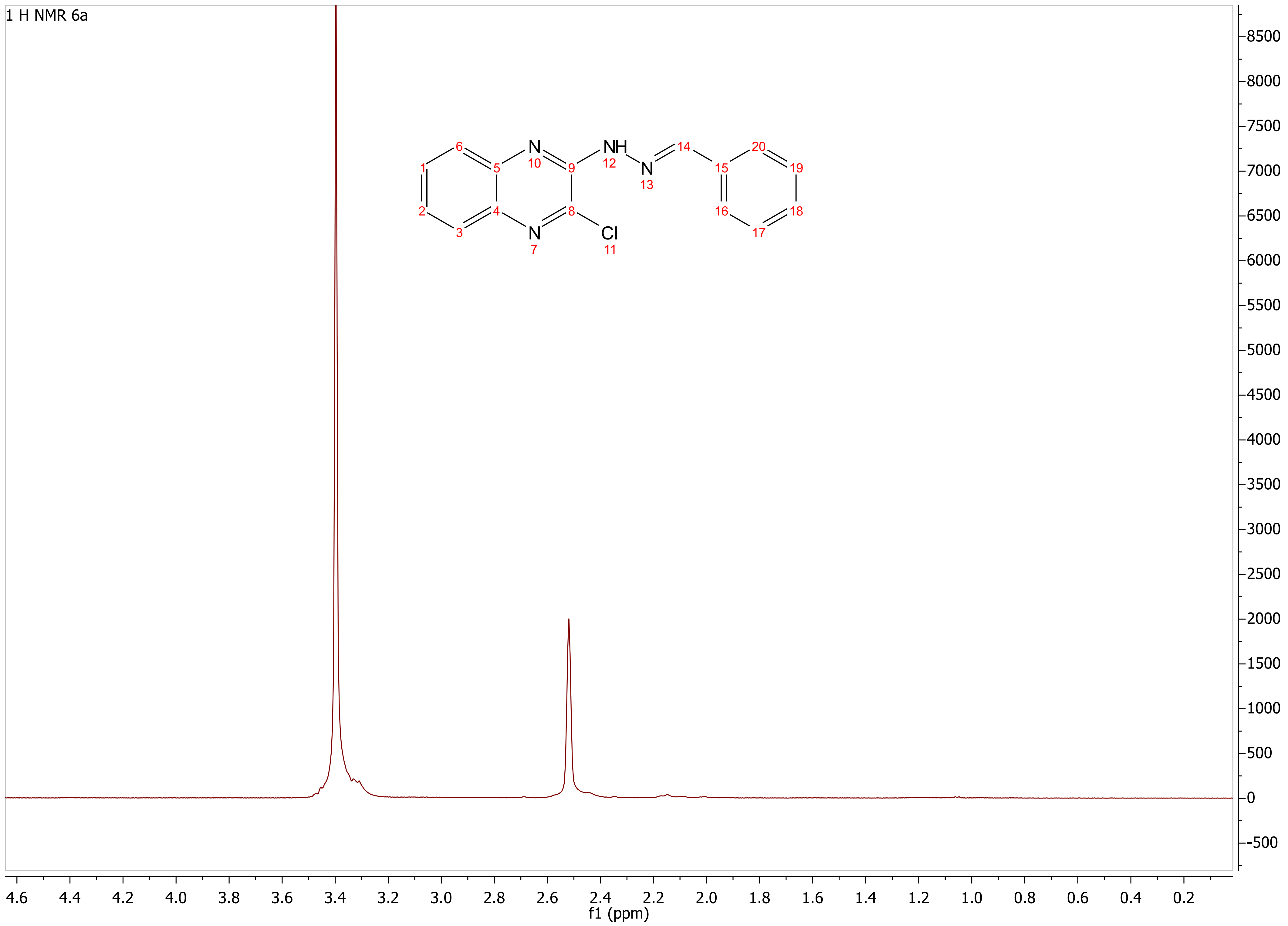
1 H NMR 6a
N
NH
N
N
Cl
1 2 3 4 5 6 7 8 9 10 11 12 13 14 15 16 17 18 19 20
f1 (ppm)
4.6 4.4 4.2 4.0 3.8 3.6 3.4 3.2 3.0 2.8 2.6 2.4 2.2 2.0 1.8 1.6 1.4 1.2 1.0 0.8 0.6 0.4 0.2
8500 8000 7500 7000 6500 6000 5500 5000 4500 4000 3500 3000 2500 2000 1500 1000 500 0 -500

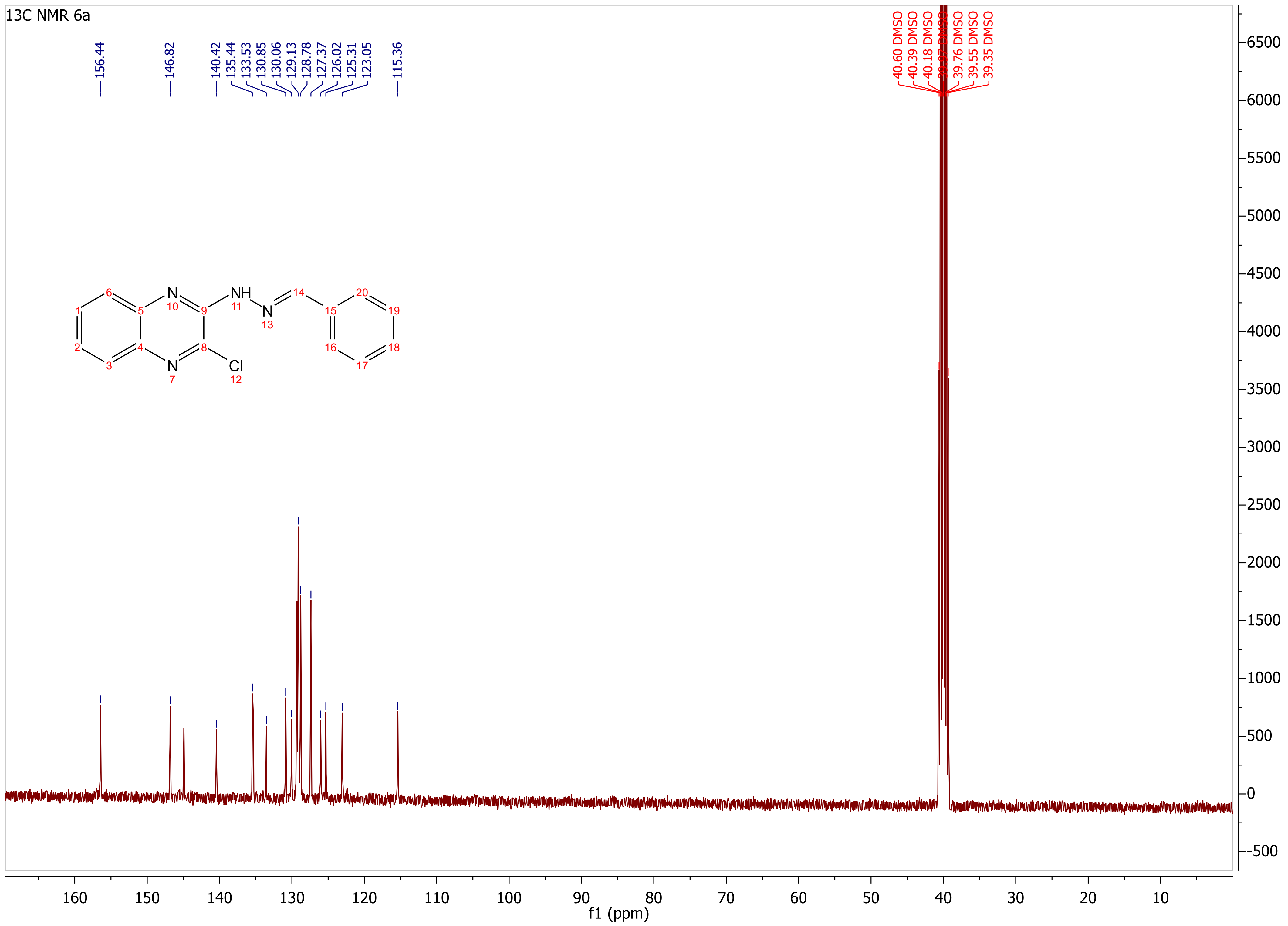
13C NMR 6a
156.44
146.82
140.42
135.44
133.53
130.85
130.06
129.13
128.78
127.37
126.02
125.31
123.05
115.36
40.60 DMSO
40.39 DMSO
40.18 DMSO
39.76 DMSO
39.55 DMSO
39.35 DMSO
NH
N
Cl
f1 (ppm)

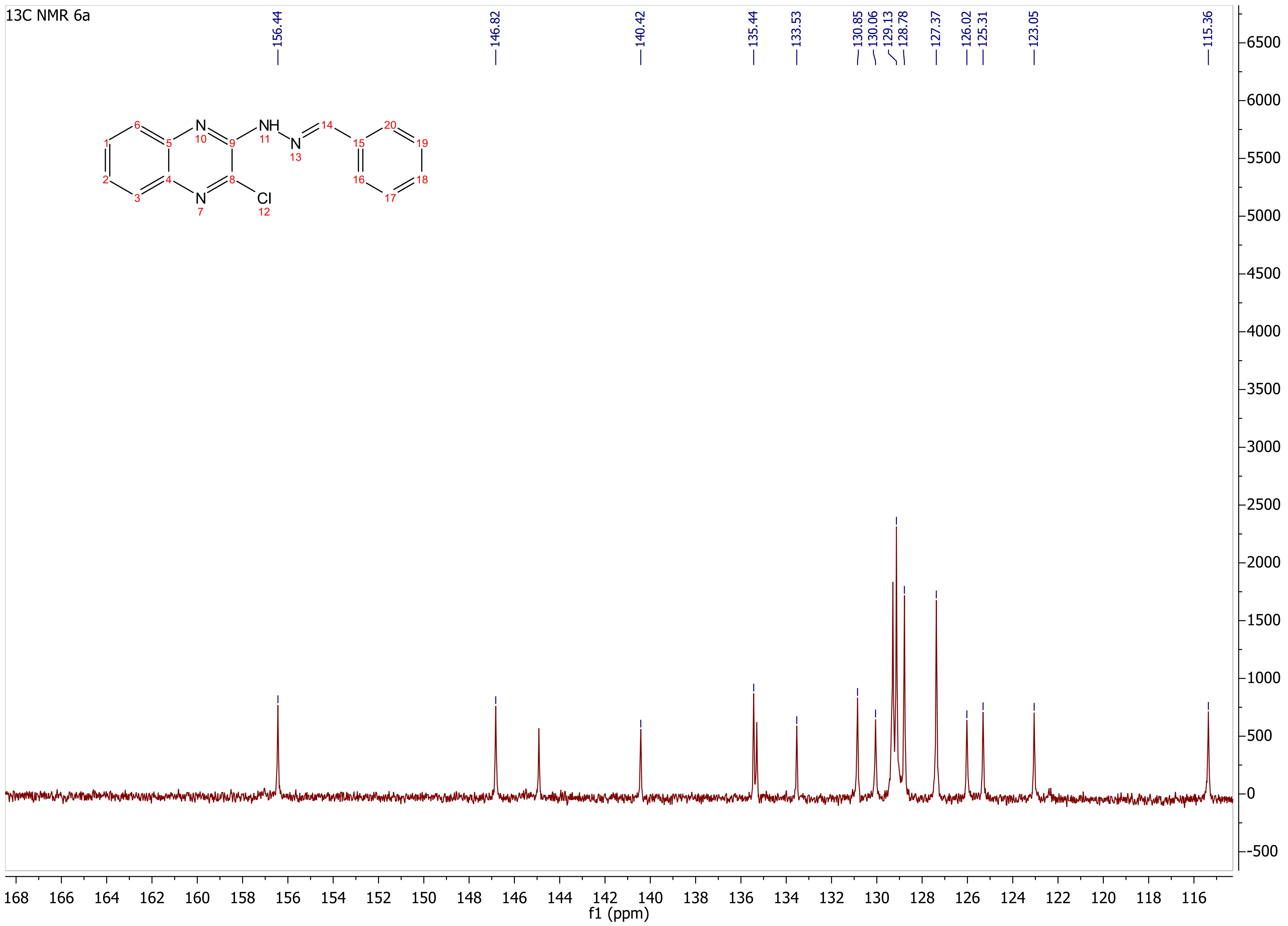
13C NMR 6a
156.44
146.82
140.42
135.44
133.53
130.85
130.06
129.13
128.78
127.37
126.02
125.31
123.05
115.36
f1 (ppm)

13C NMR 6a

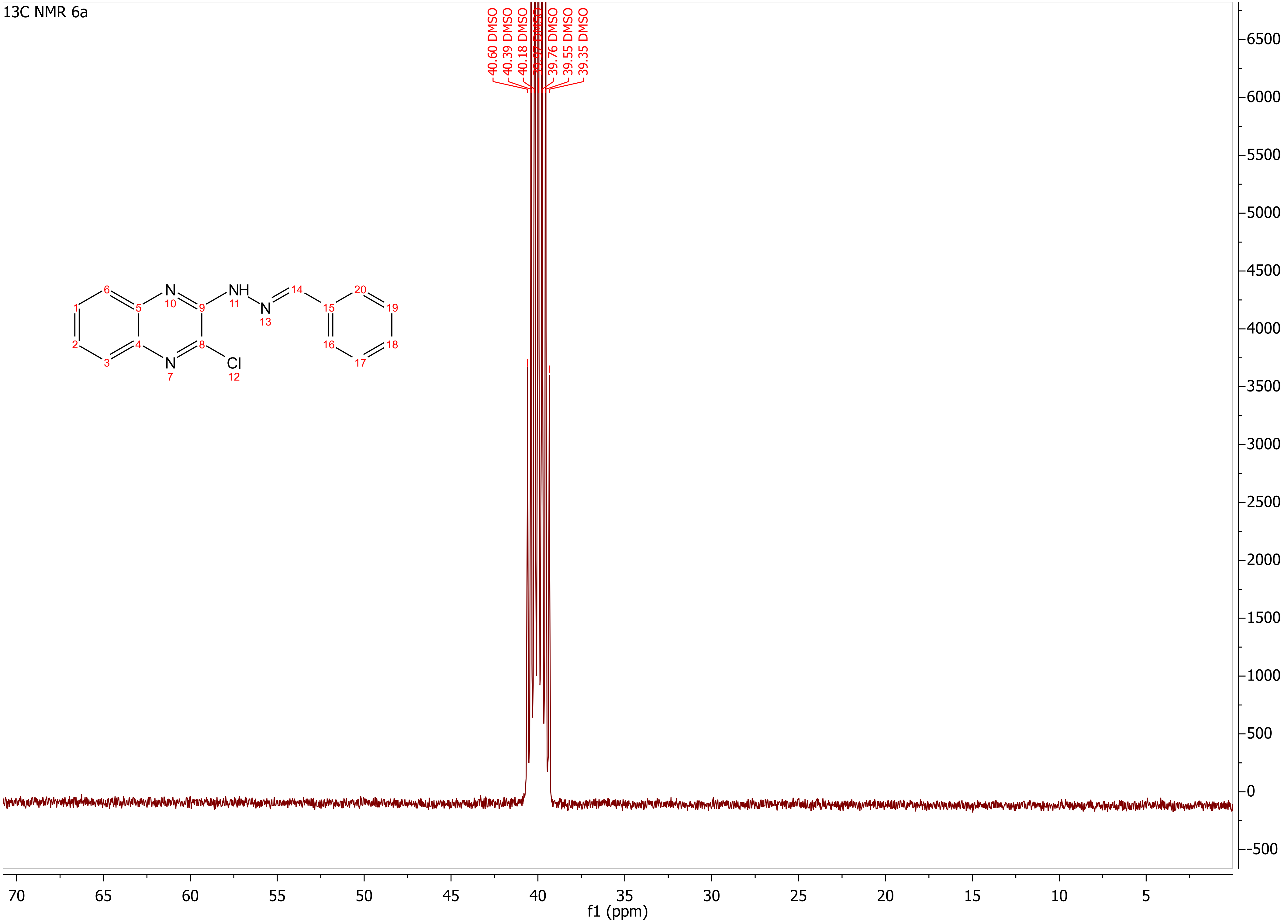

40.60 DMSO
40.39 DMSO
40.18 DMSO
39.97 DMSO
39.76 DMSO
39.55 DMSO
39.35 DMSO
N
NH
N
Cl
f1 (ppm)

IR of comp. 6b

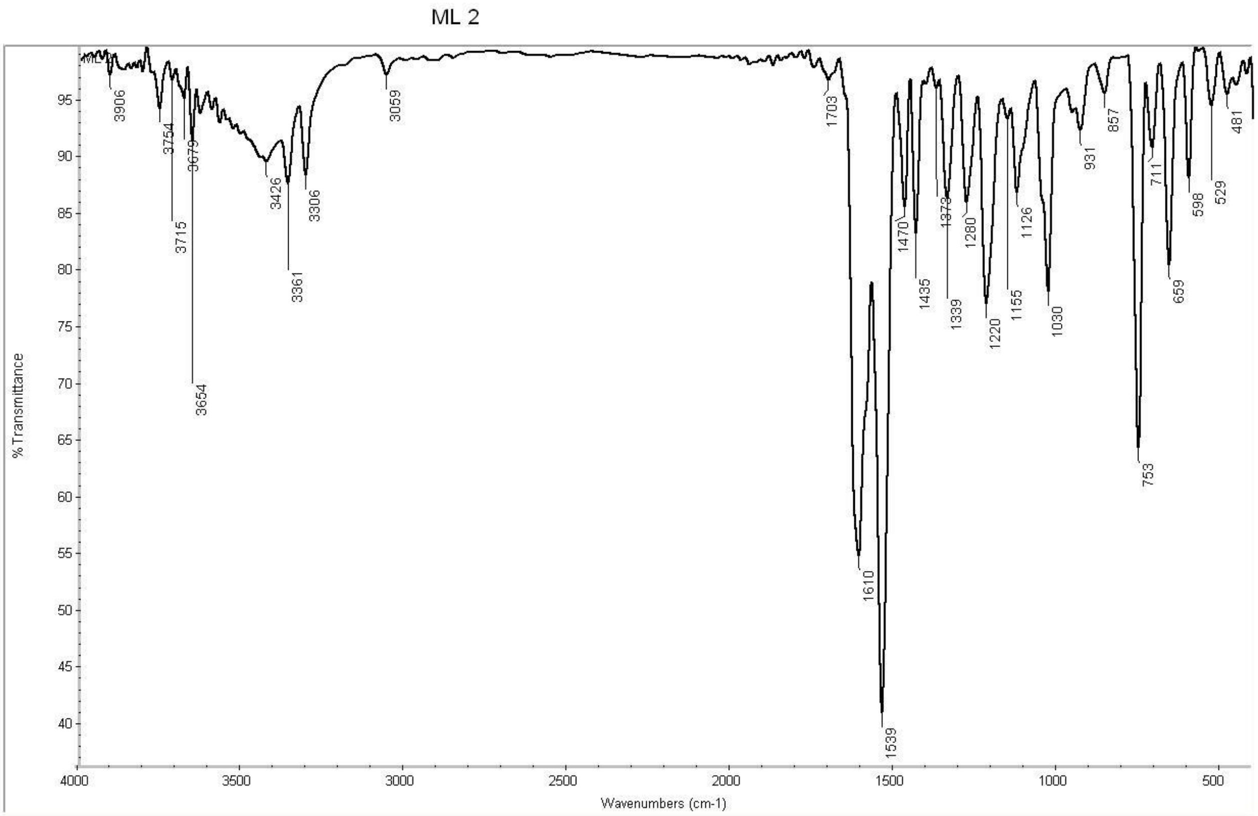
ML 2
% Transmittance
Wavenumbers (cm-1)
4000
3500
3000
2500
2000
1500
1000
500
95
90
85
80
75
70
65
60
55
50
45
40
3906
3754
3715
3679
3654
3426
3361
3306
3059
1703
1610
1539
1470
1435
1373
1339
1280
1220
1155
1126
1030
931
857
753
711
659
598
529
481

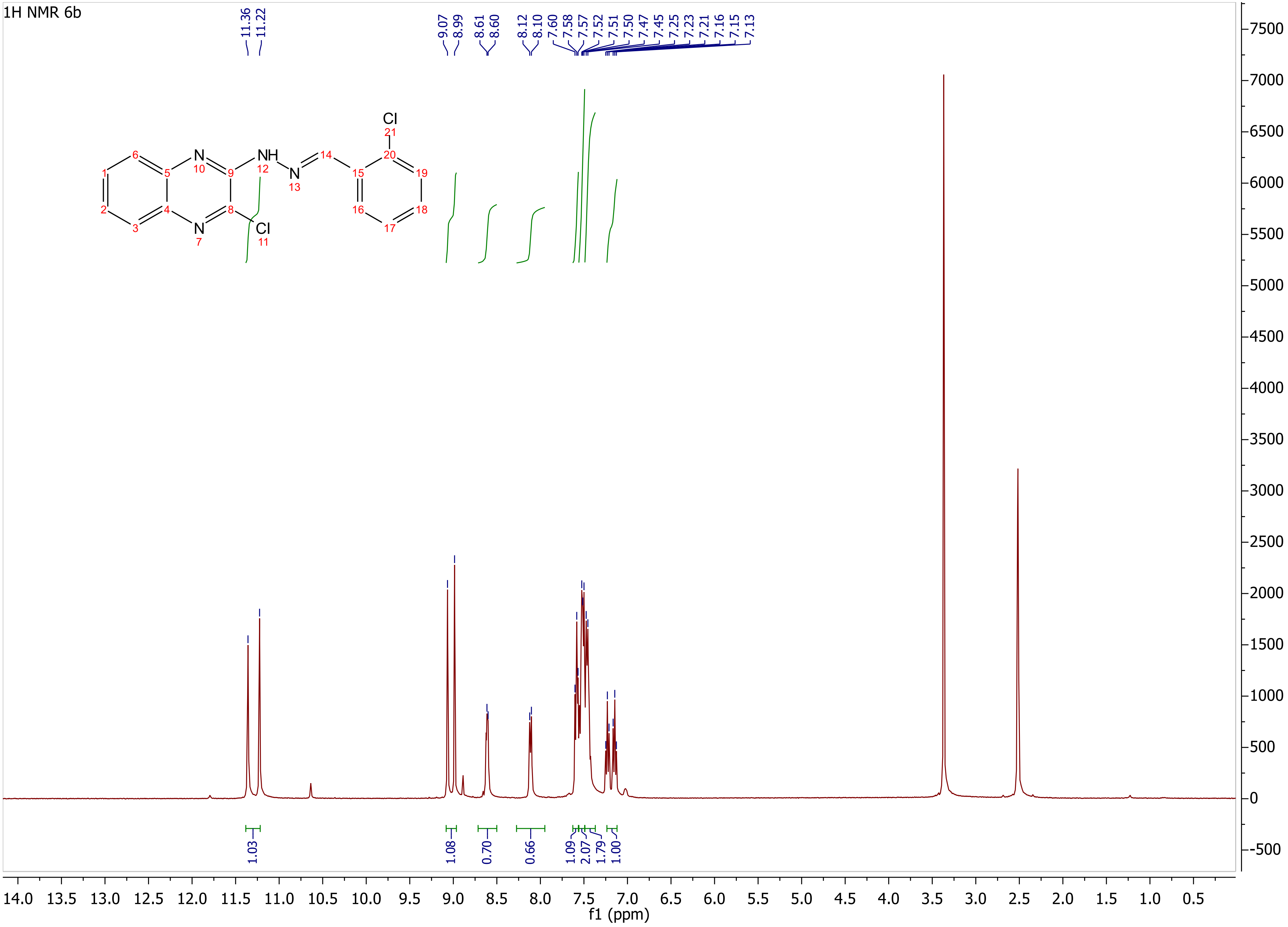
1H NMR 6b
11.36
11.22
9.07
8.99
8.61
8.60
8.12
8.10
7.60
7.58
7.57
7.52
7.51
7.50
7.47
7.45
7.25
7.23
7.21
7.16
7.15
7.13
Cl
21
20
19
18
17
16
15
14
N
13
NH
12
9
N
10
5
6
1
2
3
4
N
7
8
Cl
11
1.03
1.08
0.70
0.66
1.09
2.07
1.79
1.00
f1 (ppm)

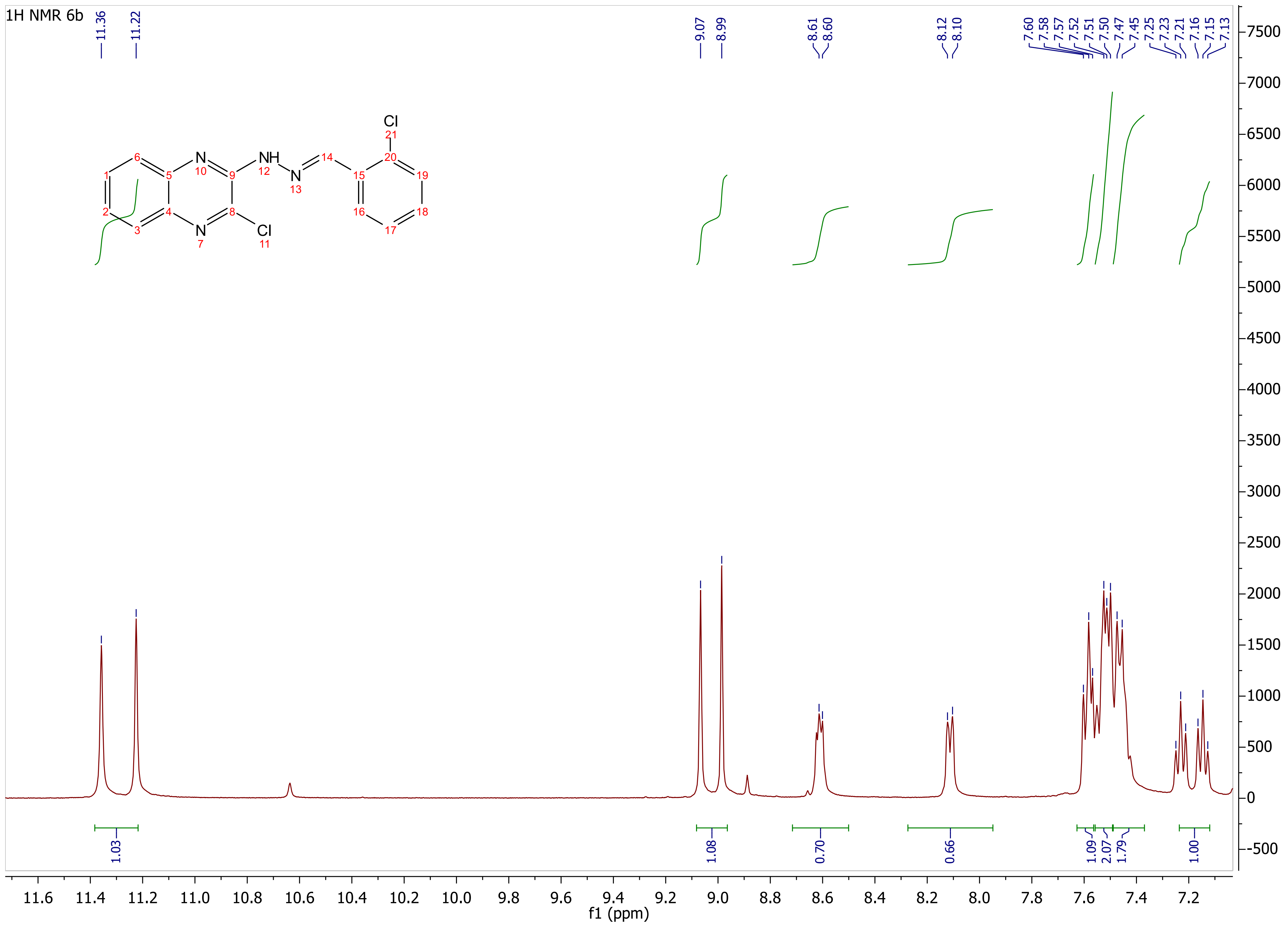
1H NMR 6b
11.36
11.22
9.07
8.99
8.61
8.60
8.12
8.10
7.60
7.58
7.57
7.52
7.51
7.50
7.47
7.45
7.25
7.23
7.21
7.16
7.15
7.13
1.03
1.08
0.70
0.66
1.09
2.07
1.79
1.00
f1 (ppm)

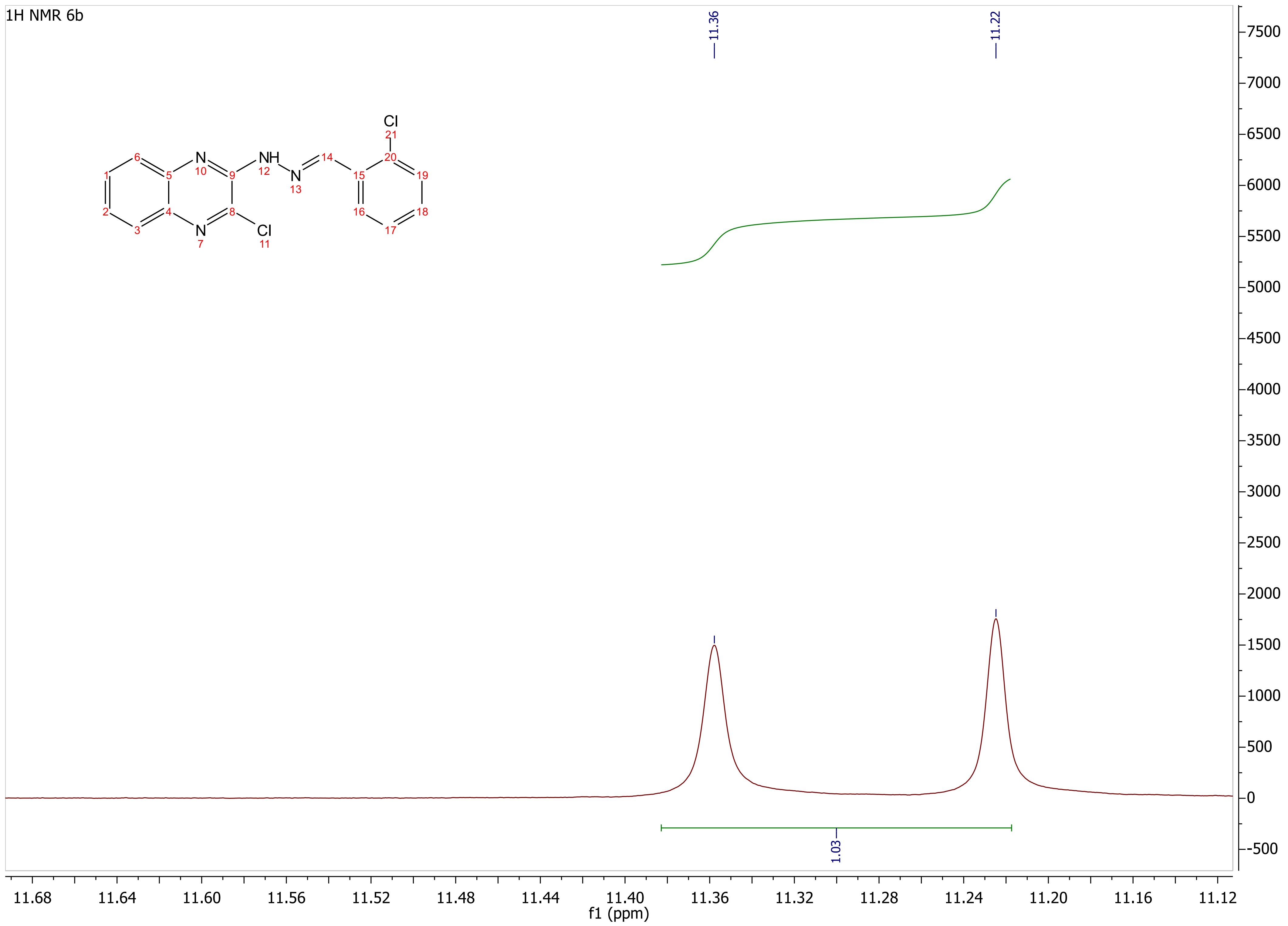
1H NMR 6b
11.36
11.22
1.03
f1 (ppm)
Cl
NH
N
Cl

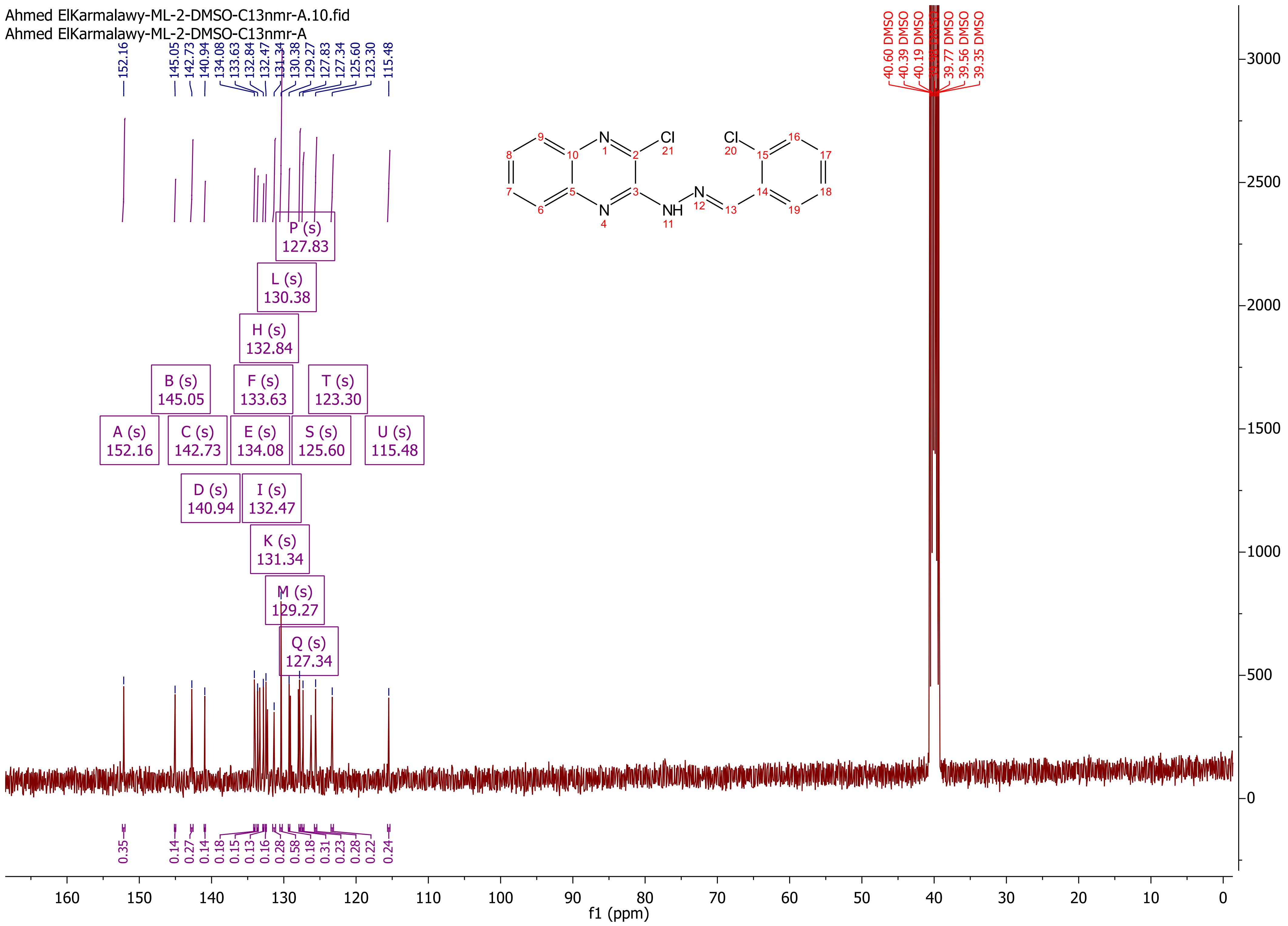
Ahmed ElKarmalawy-ML-2-DMSO-C13nmr-A.10.fid
Ahmed ElKarmalawy-ML-2-DMSO-C13nmr-A
152.16
145.05
142.73
140.94
134.08
133.63
132.84
132.47
131.34
130.38
129.27
127.83
127.34
125.60
123.30
115.48
40.60 DMSO
40.39 DMSO
40.19 DMSO
39.77 DMSO
39.56 DMSO
39.35 DMSO
N
Cl
Cl
NH
N
1
2
3
4
5
6
7
8
9
10
11
12
13
14
15
16
17
18
19
20
21
P (s) 127.83
L (s) 130.38
H (s) 132.84
B (s) 145.05
F (s) 133.63
T (s) 123.30
A (s) 152.16
C (s) 142.73
E (s) 134.08
S (s) 125.60
U (s) 115.48
D (s) 140.94
I (s) 132.47
K (s) 131.34
M (s) 129.27
Q (s) 127.34
0.35
0.14
0.27
0.14
0.18
0.15
0.13
0.16
0.28
0.58
0.18
0.31
0.23
0.28
0.22
0.24
f1 (ppm)
160
150
140
130
120
110
100
90
80
70
60
50
40
30
20
10
0
3000
2500
2000
1500
1000
500
0

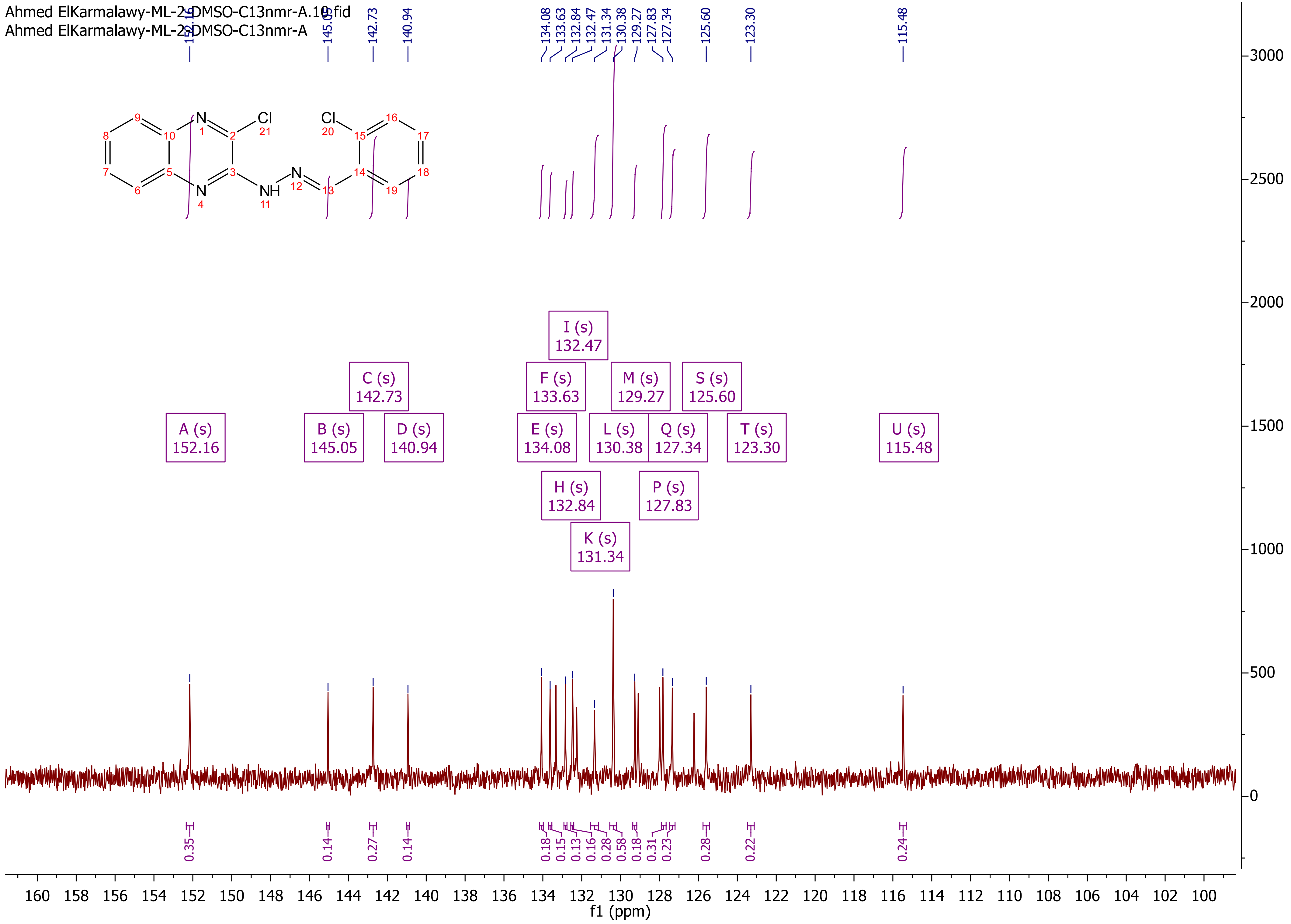
Ahmed ElKarmalawy-ML-2-DMSO-C13nmr-A.10.fid
Ahmed ElKarmalawy-ML-2-DMSO-C13nmr-A
152.16
145.05
142.73
140.94
134.08
133.63
132.84
132.47
131.34
130.38
129.27
127.83
127.34
125.60
123.30
115.48
A (s) 152.16
B (s) 145.05
C (s) 142.73
D (s) 140.94
E (s) 134.08
F (s) 133.63
H (s) 132.84
I (s) 132.47
K (s) 131.34
L (s) 130.38
M (s) 129.27
P (s) 127.83
Q (s) 127.34
S (s) 125.60
T (s) 123.30
U (s) 115.48
0.35
0.14
0.27
0.14
0.18
0.15
0.13
0.16
0.28
0.58
0.18
0.31
0.23
0.28
0.22
0.24
f1 (ppm)

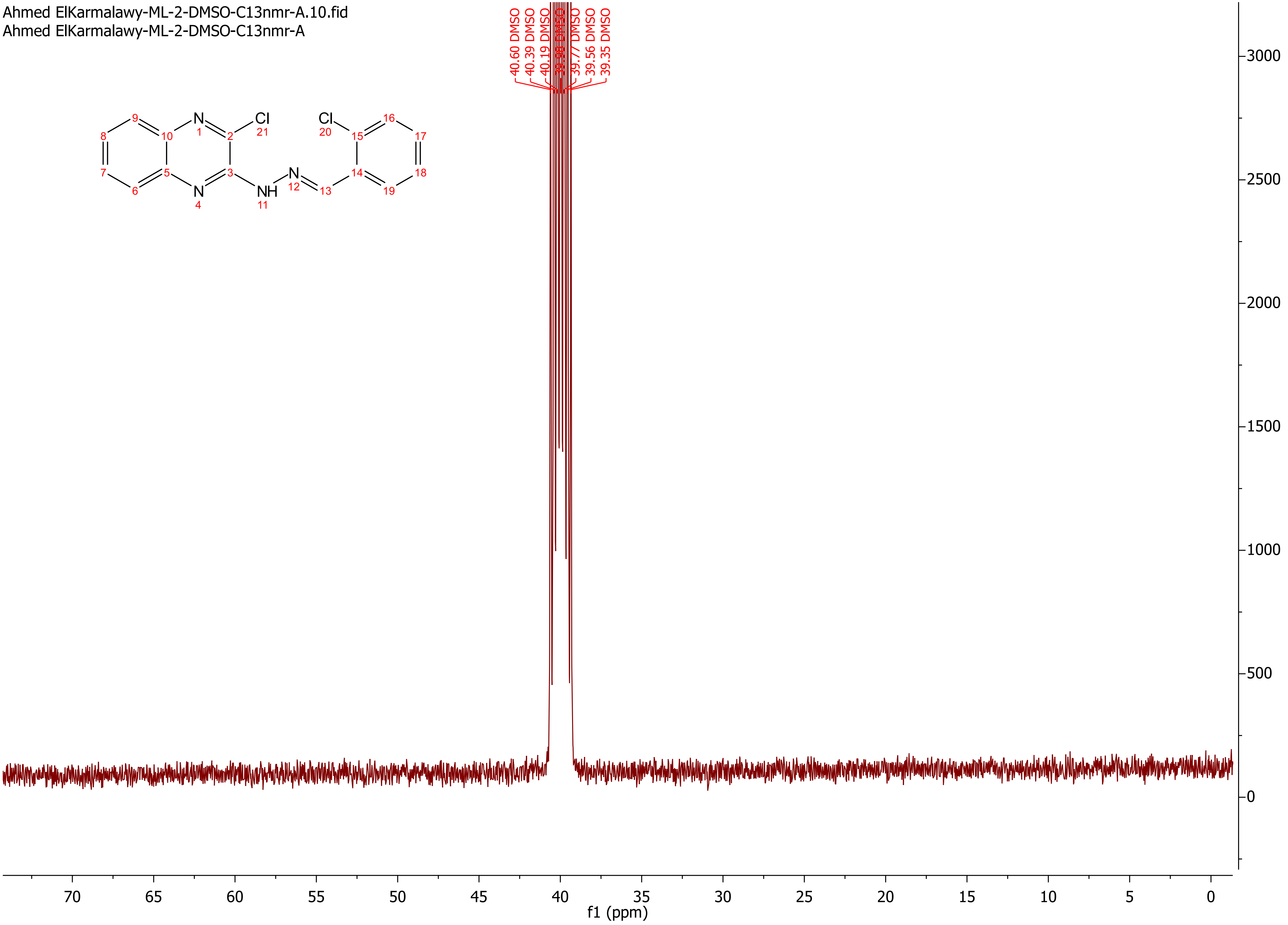

Ahmed ElKarmalawy-ML-2-DMSO-C13nmr-A.10.fid
Ahmed ElKarmalawy-ML-2-DMSO-C13nmr-A
40.60 DMSO
40.39 DMSO
40.19 DMSO
39.98 DMSO
39.77 DMSO
39.56 DMSO
39.35 DMSO
N
Cl
NH
f1 (ppm)

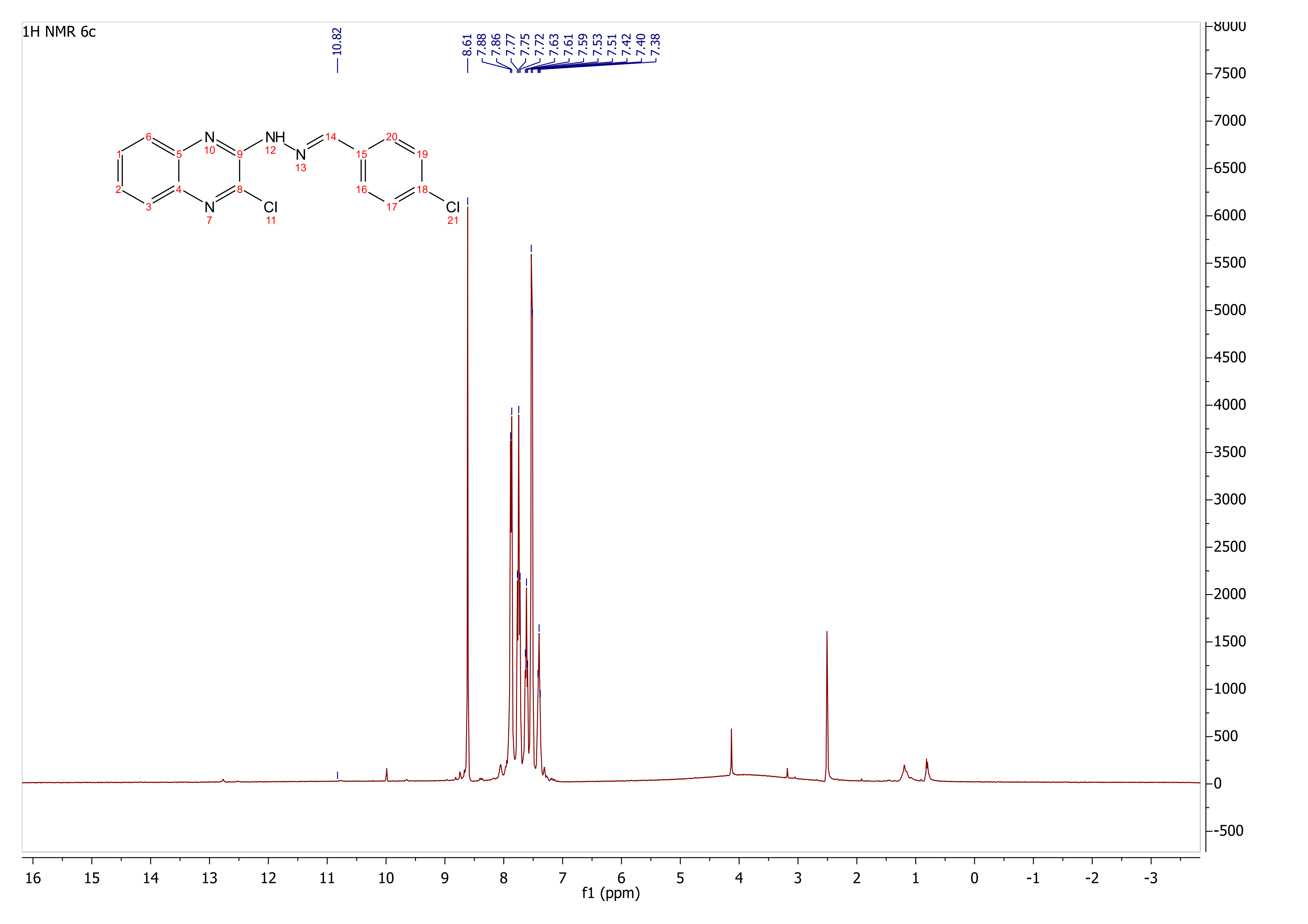

1H NMR 6c
10.82
8.61
7.88
7.86
7.77
7.75
7.72
7.63
7.61
7.59
7.53
7.51
7.42
7.40
7.38
f1 (ppm)

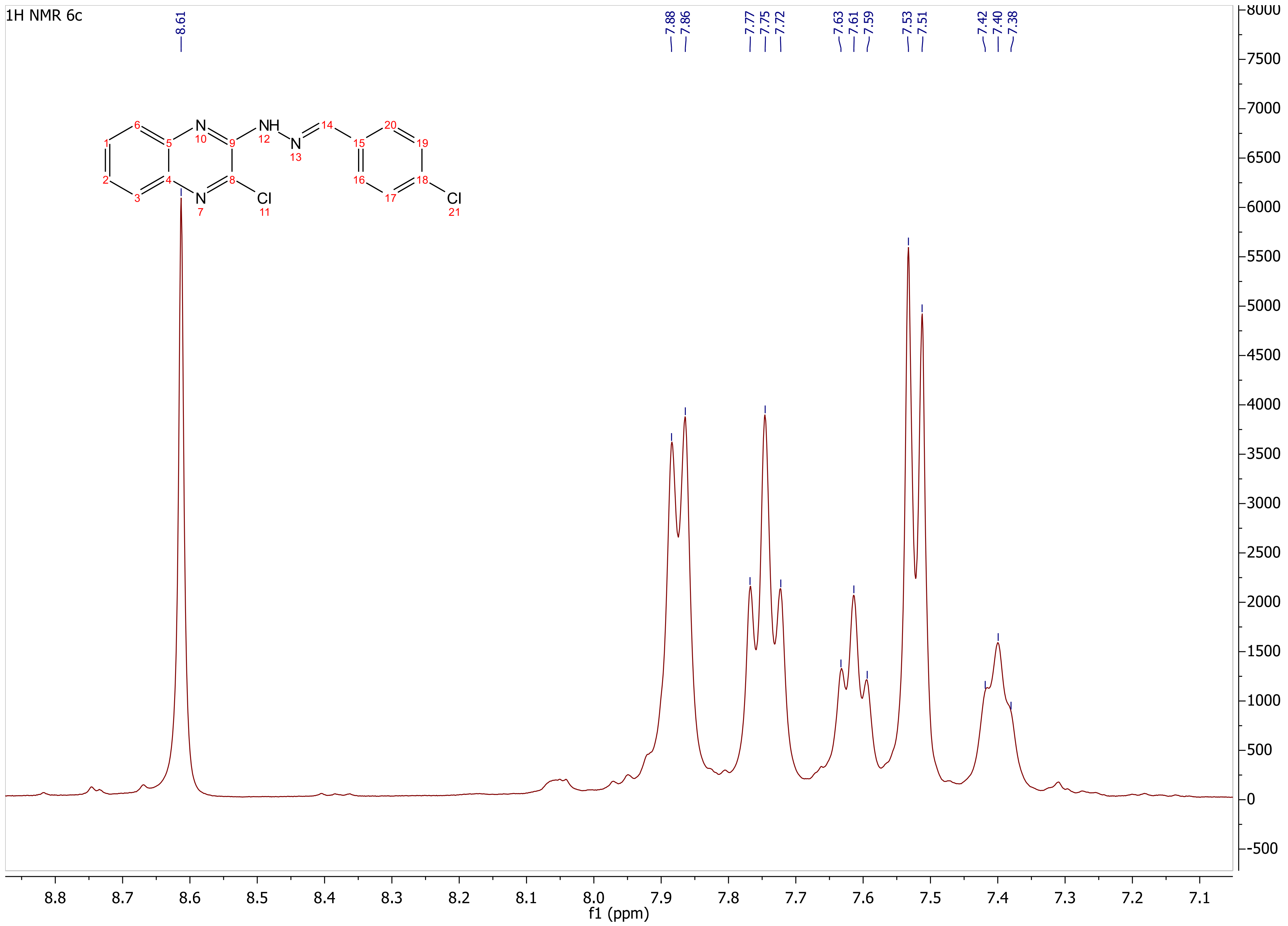

1H NMR 6c
8.61
7.88
7.86
7.77
7.75
7.72
7.63
7.61
7.59
7.53
7.51
7.42
7.40
7.38
N
NH
N
Cl
Cl
f1 (ppm)

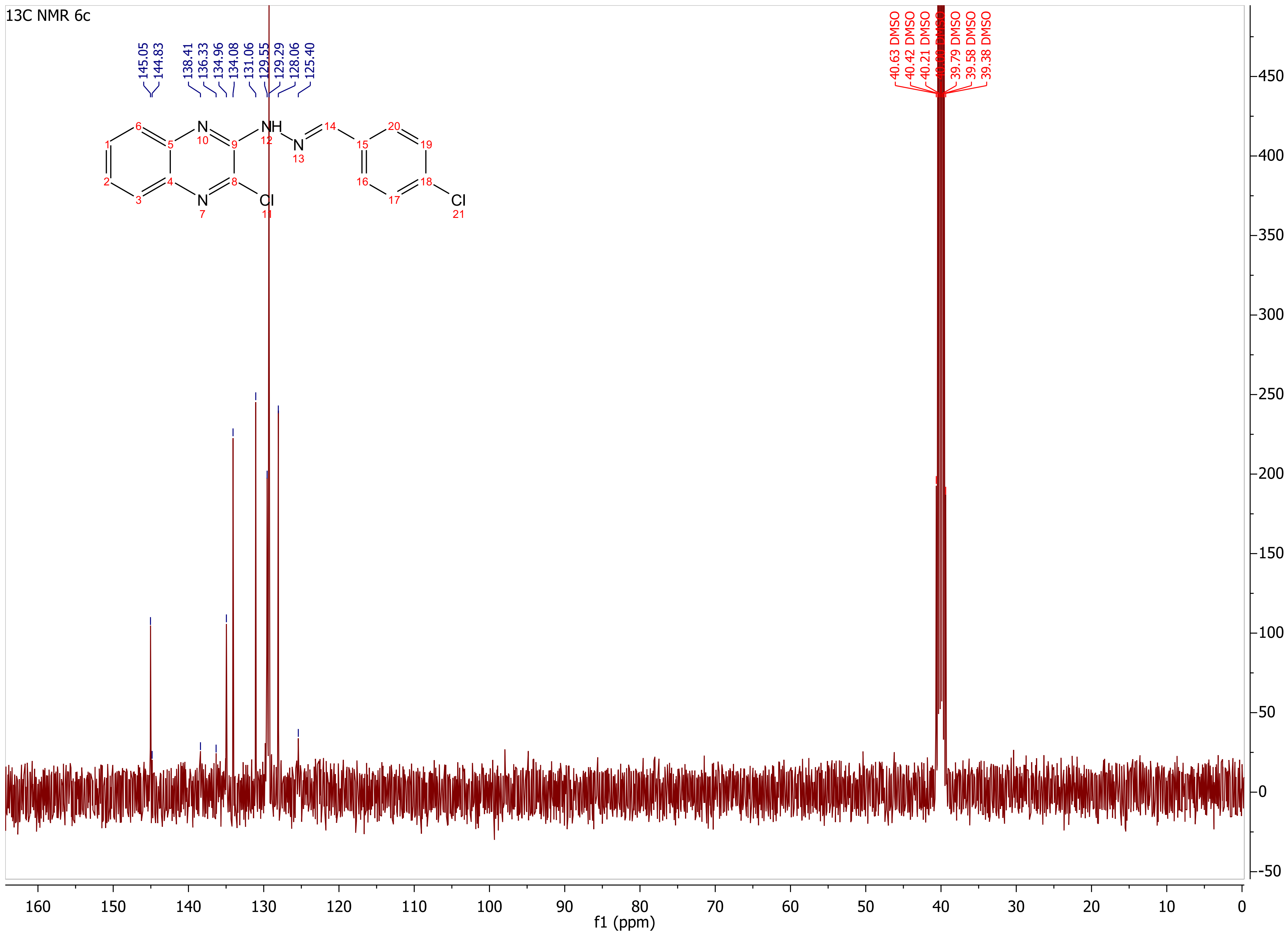
13C NMR 6c
145.05
144.83
138.41
136.33
134.96
134.08
131.06
129.55
129.29
128.06
125.40
40.63 DMSO
40.42 DMSO
40.21 DMSO
39.79 DMSO
39.58 DMSO
39.38 DMSO
NH
N
N
N
Cl
Cl
f1 (ppm)

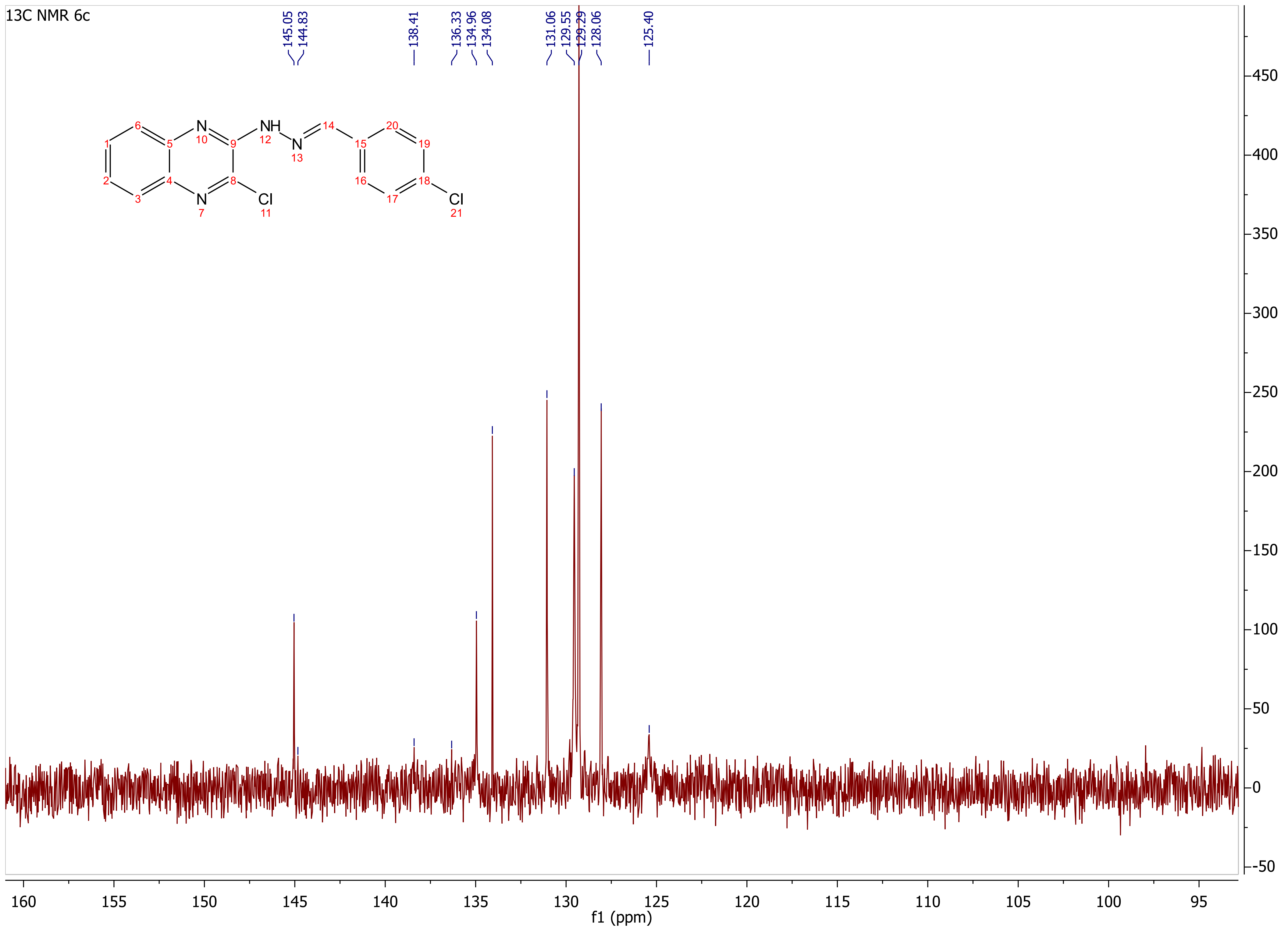

13C NMR 6c
145.05
144.83
138.41
136.33
134.96
134.08
131.06
129.55
129.29
128.06
125.40
f1 (ppm)

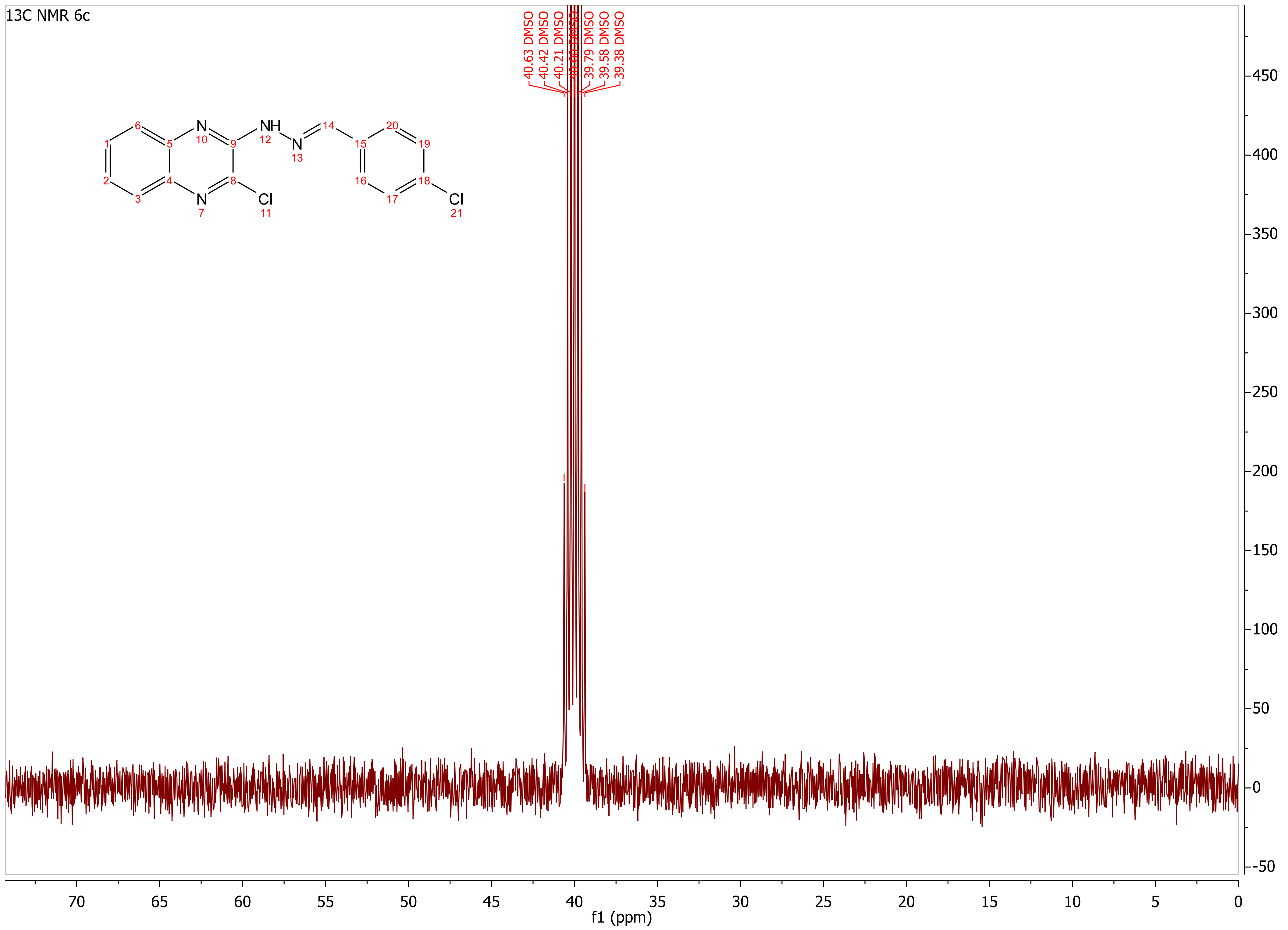
13C NMR 6c
40.63 DMSO
40.42 DMSO
40.21 DMSO
40.00 DMSO
39.79 DMSO
39.58 DMSO
39.38 DMSO
N
NH
N
Cl
N
Cl
f1 (ppm)
70
65
60
55
50
45
40
35
30
25
20
15
10
5
0
450
400
350
300
250
200
150
100
50
0
-50

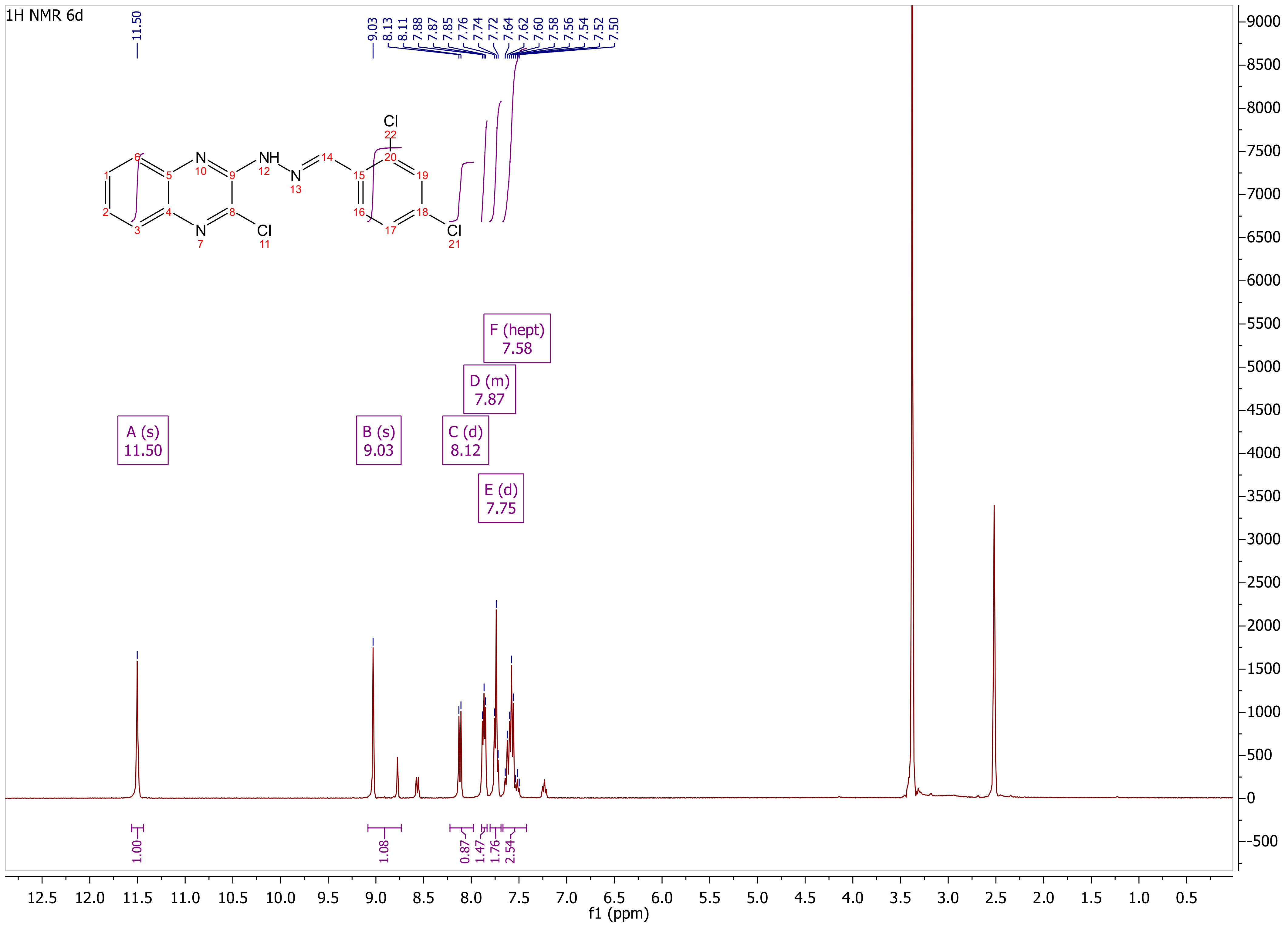
1H NMR 6d
11.50
9.03 8.13 8.11 7.88 7.87 7.85 7.76 7.74 7.72 7.64 7.62 7.60 7.58 7.56 7.54 7.52 7.50
A (s) 11.50
B (s) 9.03
C (d) 8.12
D (m) 7.87
E (d) 7.75
F (hept) 7.58
1.00
1.08
0.87
1.47
1.76
2.54
f1 (ppm)

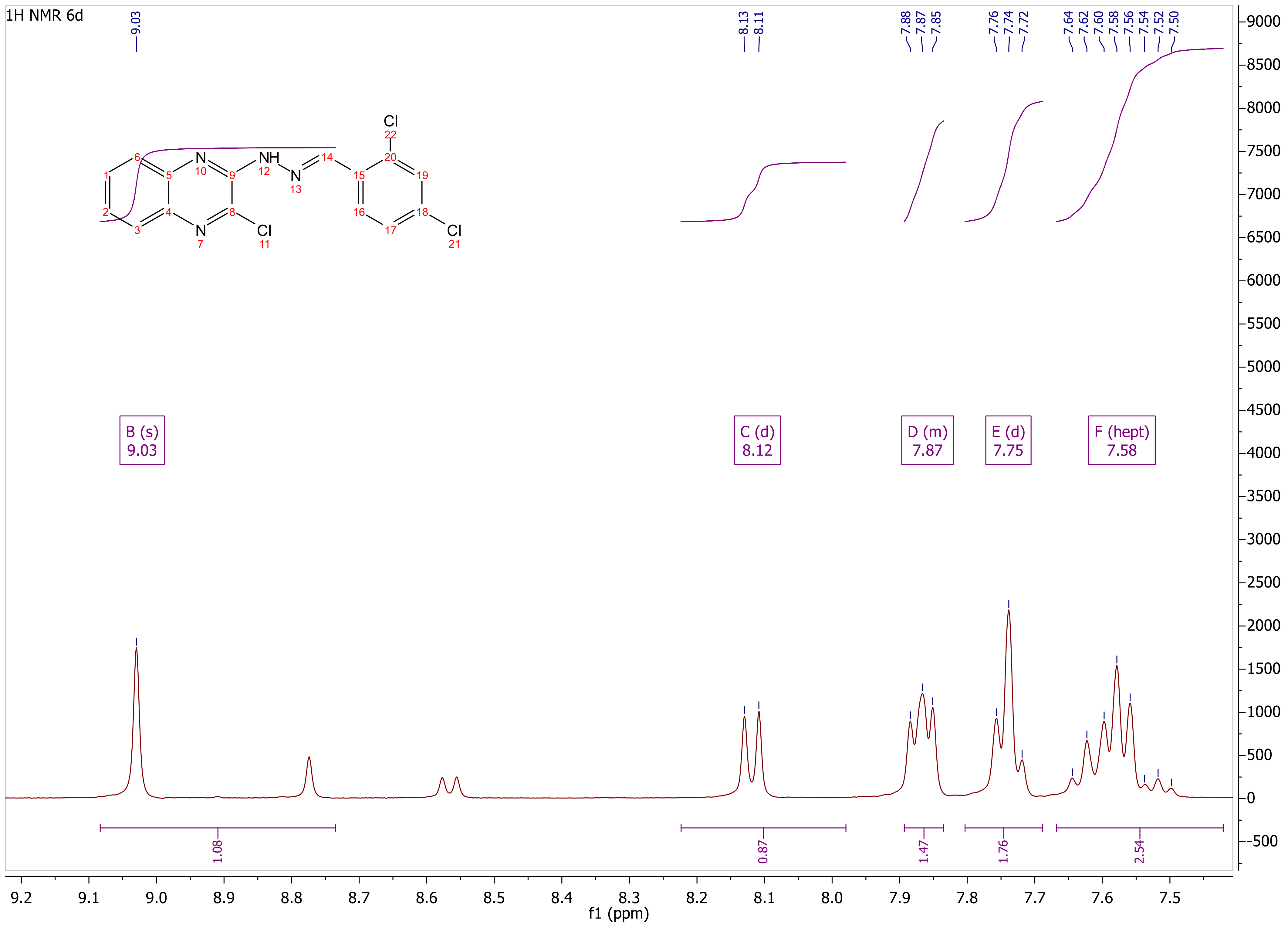
1H NMR 6d
9.03
8.13
8.11
7.88
7.87
7.85
7.76
7.74
7.72
7.64
7.62
7.60
7.58
7.56
7.54
7.52
7.50
B (s) 9.03
C (d) 8.12
D (m) 7.87
E (d) 7.75
F (hept) 7.58
1.08
0.87
1.47
1.76
2.54
f1 (ppm)

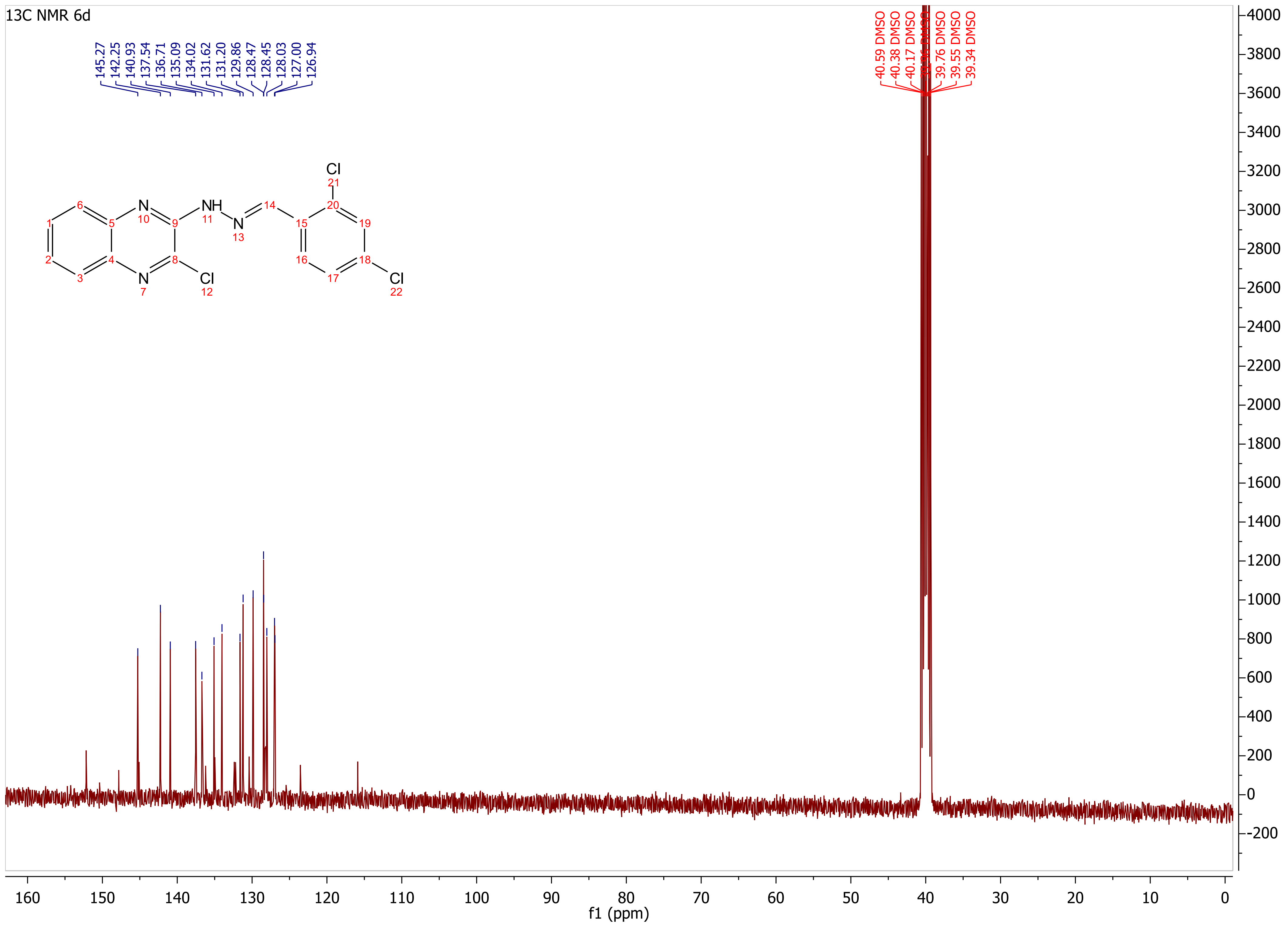

13C NMR 6d
145.27
142.25
140.93
137.54
136.71
135.09
134.02
131.62
131.20
129.86
128.47
128.45
128.03
127.00
126.94
40.59 DMSO
40.38 DMSO
40.17 DMSO
39.76 DMSO
39.55 DMSO
39.34 DMSO
Cl
N
NH
N
Cl
Cl
f1 (ppm)

13C NMR 6d

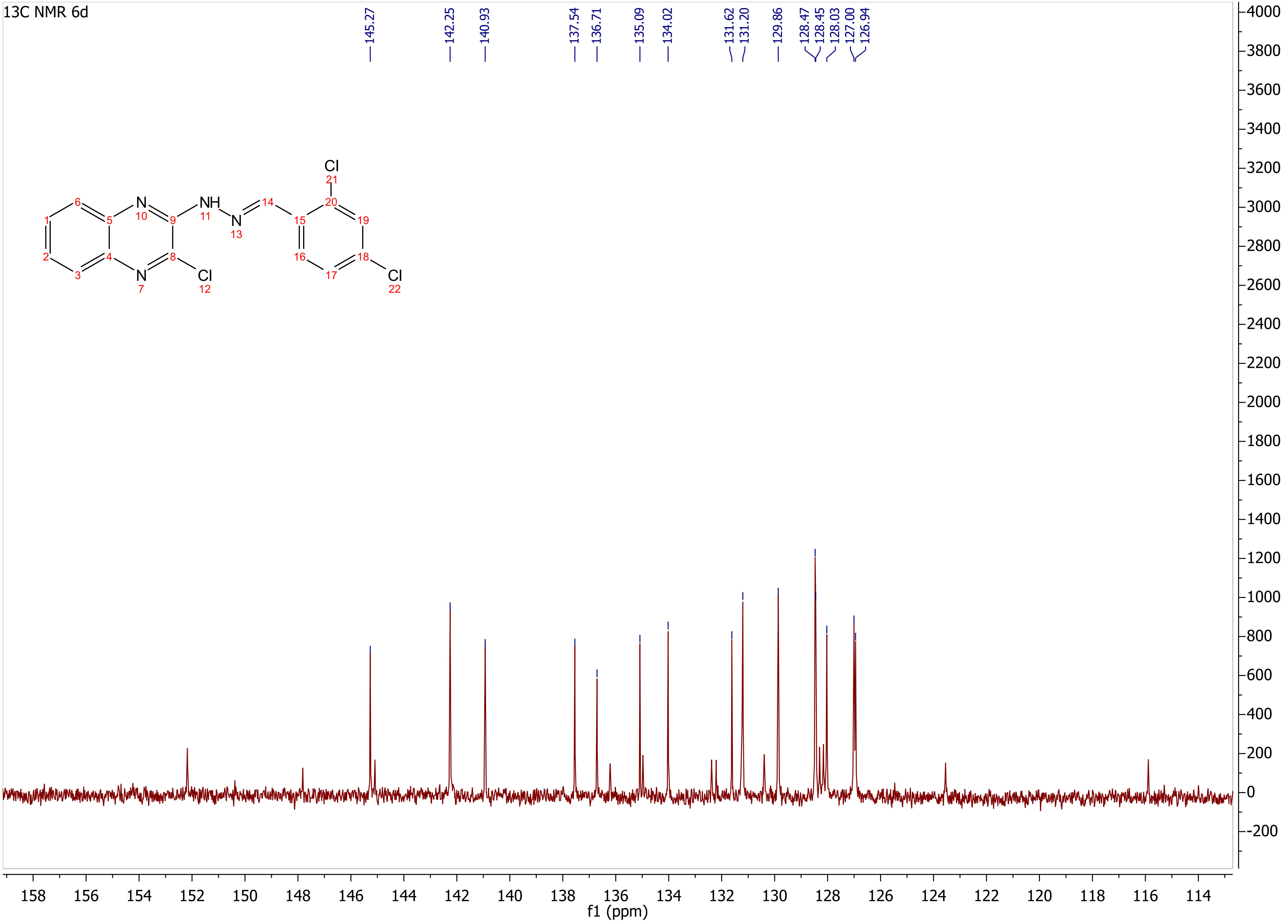

145.27
142.25
140.93
137.54
136.71
135.09
134.02
131.62
131.20
129.86
128.47
128.45
128.03
127.00
126.94
Cl
21
N
NH
6
10
9
11
14
20
19
1
5
N
13
15
2
4
8
16
18
Cl
3
N
Cl
17
7
12
22
4000
3800
3600
3400
3200
3000
2800
2600
2400
2200
2000
1800
1600
1400
1200
1000
800
600
400
200
0
-200
158
156
154
152
150
148
146
144
142
140
138
136
134
132
130
128
126
124
122
120
118
116
114
f1 (ppm)

13C NMR 6d

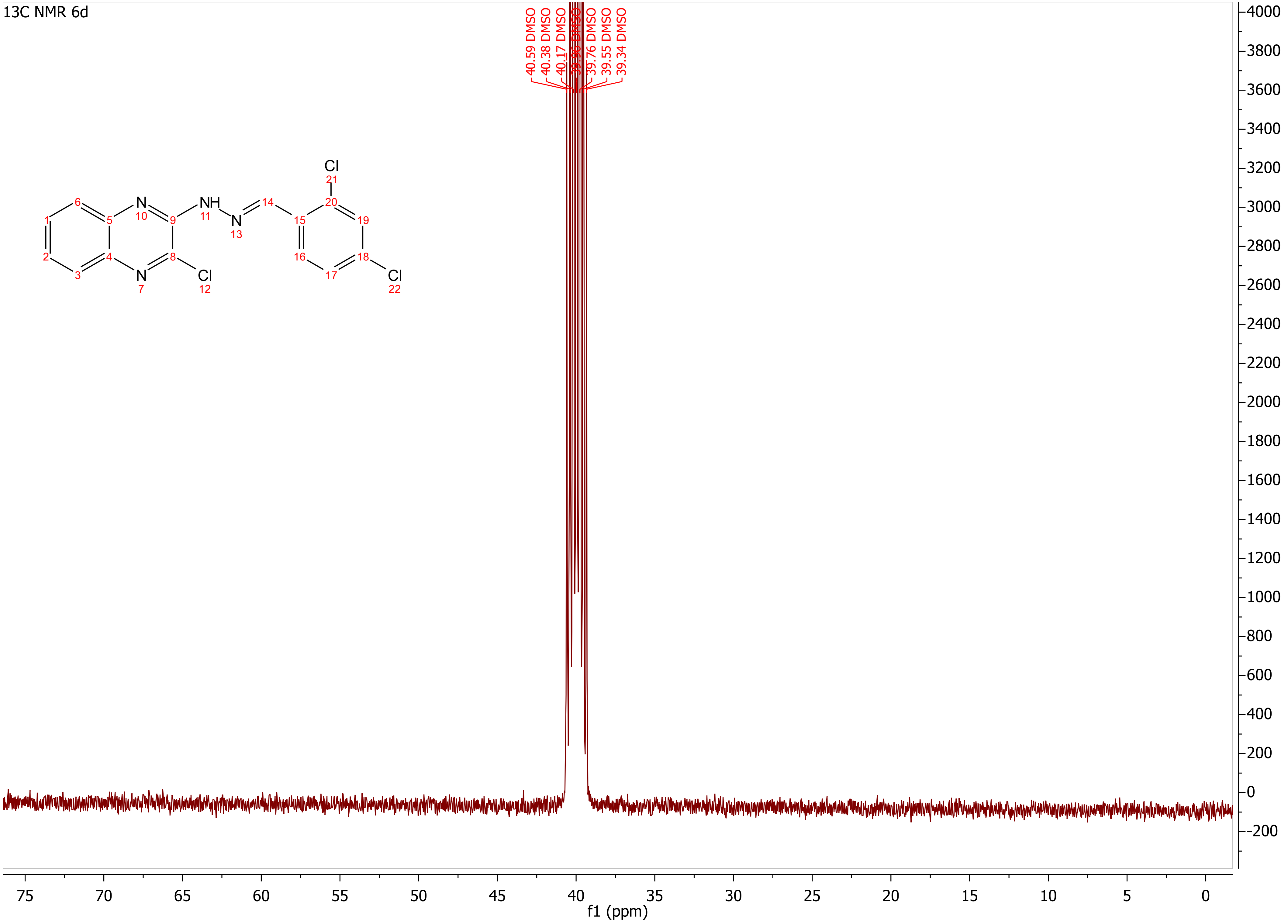

40.59 DMSO
40.38 DMSO
40.17 DMSO
39.96 DMSO
39.76 DMSO
39.55 DMSO
39.34 DMSO
f1 (ppm)

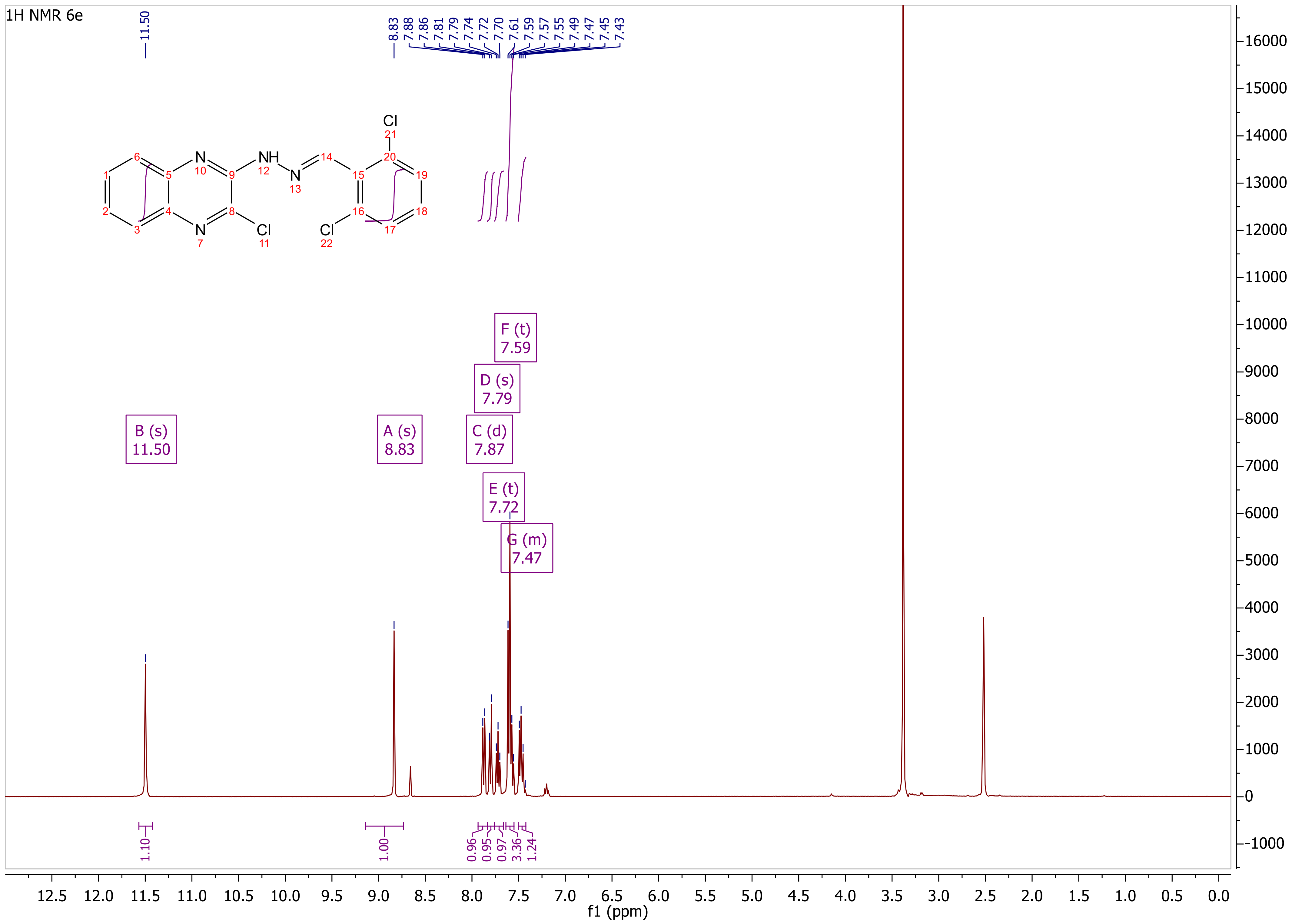
1H NMR 6e
11.50
8.83 7.88 7.86 7.81 7.79 7.74 7.72 7.70 7.61 7.59 7.57 7.55 7.49 7.47 7.45 7.43
B (s) 11.50
A (s) 8.83
C (d) 7.87
D (s) 7.79
E (t) 7.72
F (t) 7.59
G (m) 7.47
1.10
1.00
0.96 0.95 0.97 3.36 1.24
f1 (ppm)

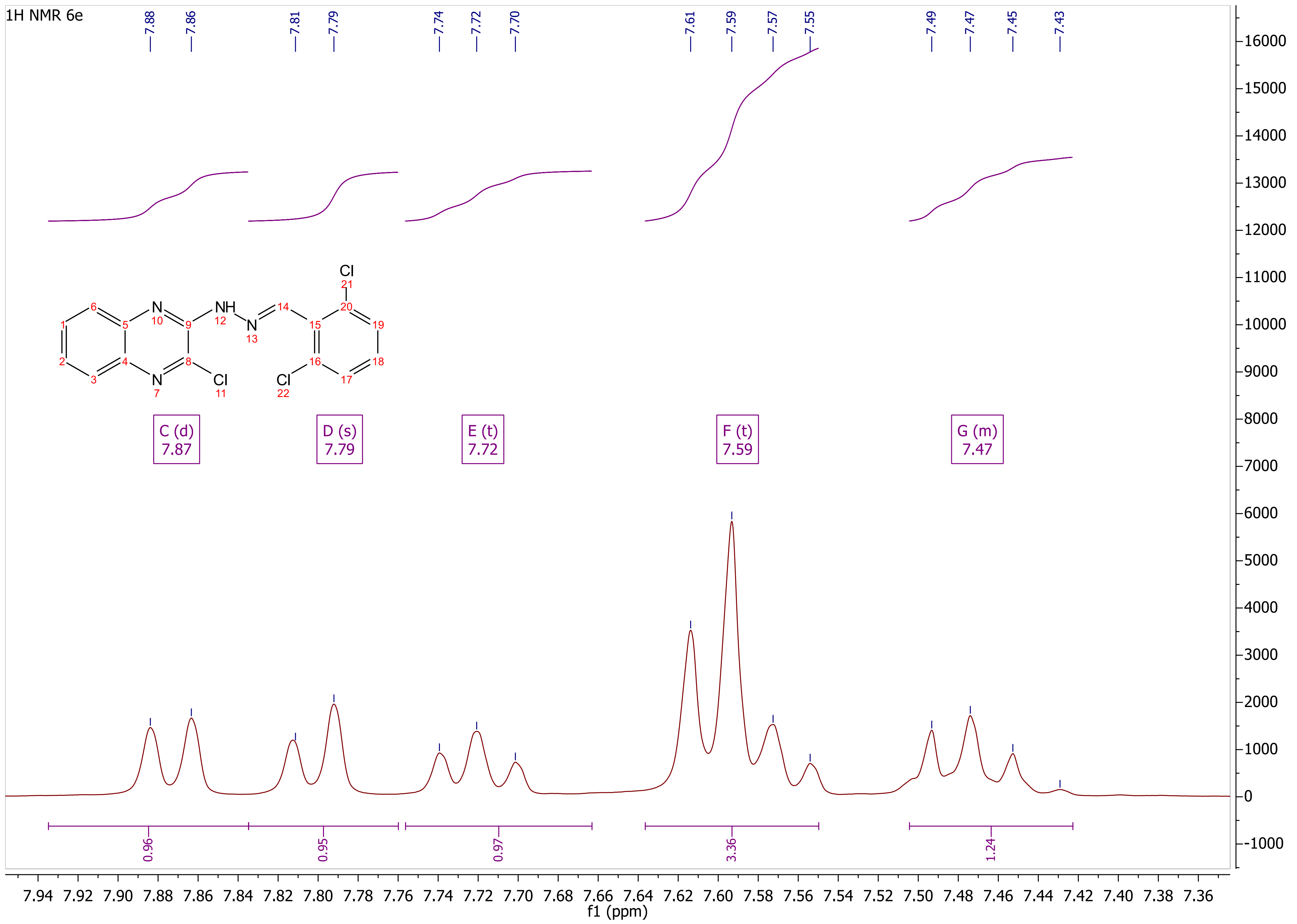

1H NMR 6e
7.88
7.86
7.81
7.79
7.74
7.72
7.70
7.61
7.59
7.57
7.55
7.49
7.47
7.45
7.43
C (d) 7.87
D (s) 7.79
E (t) 7.72
F (t) 7.59
G (m) 7.47
0.96
0.95
0.97
3.36
1.24
f1 (ppm)

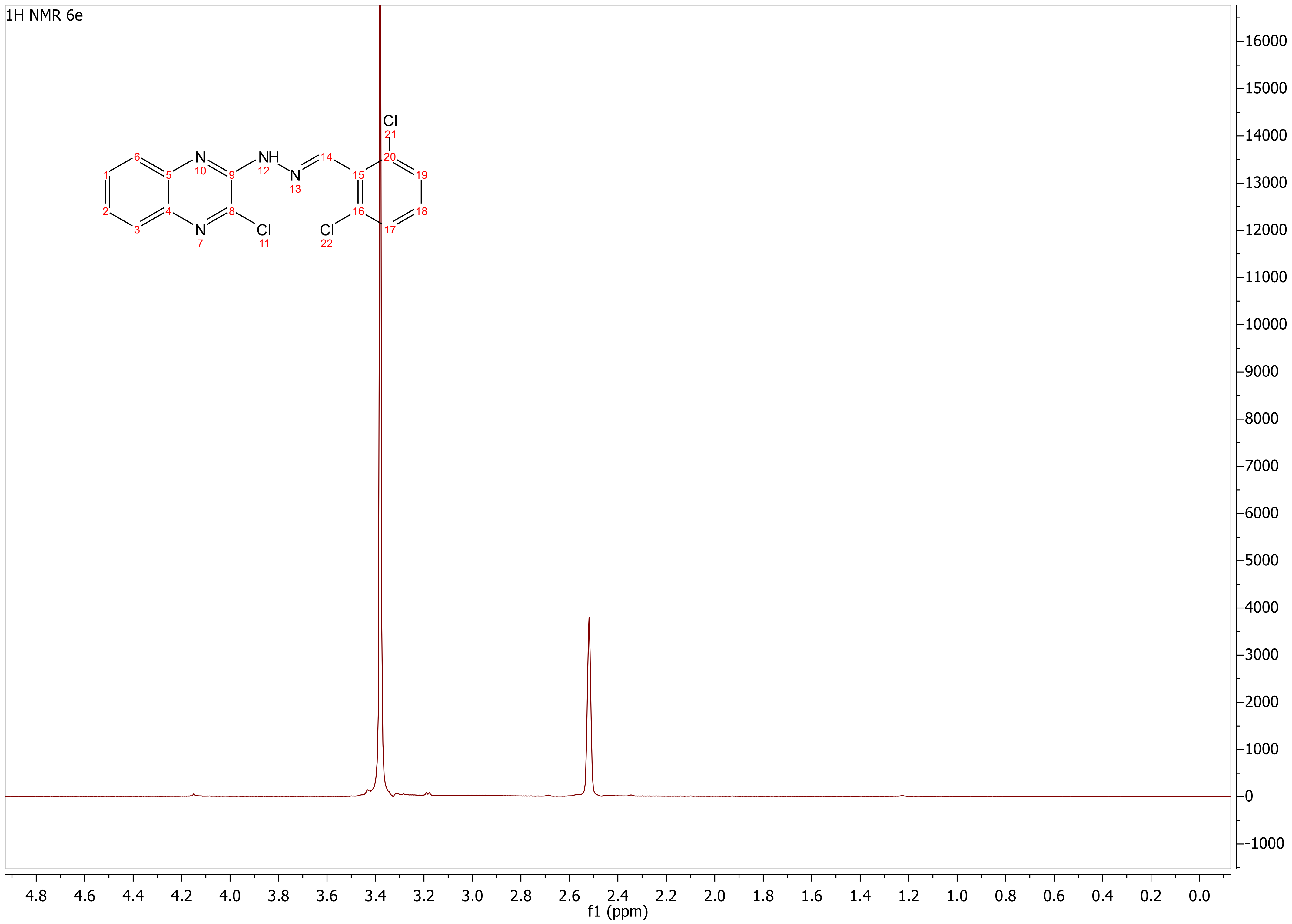
1H NMR 6e
NH
N
Cl
Cl
Cl
f1 (ppm)

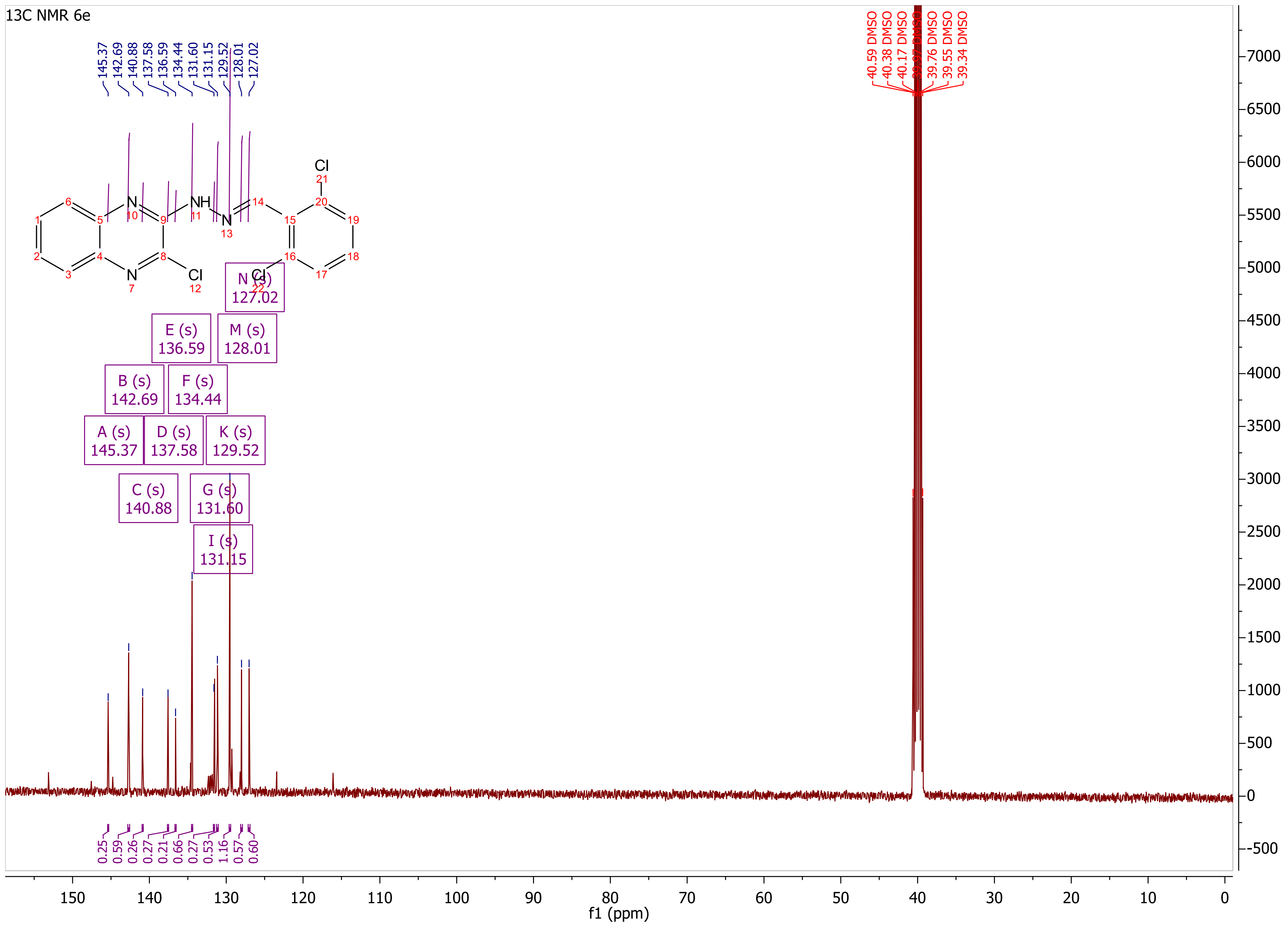
13C NMR 6e
145.37
142.69
140.88
137.58
136.59
134.44
131.60
131.15
129.52
128.01
127.02
40.59 DMSO
40.38 DMSO
40.17 DMSO
39.97 DMSO
39.76 DMSO
39.55 DMSO
39.34 DMSO
A (s) 145.37
B (s) 142.69
C (s) 140.88
D (s) 137.58
E (s) 136.59
F (s) 134.44
G (s) 131.60
I (s) 131.15
K (s) 129.52
M (s) 128.01
N (s) 127.02
0.25
0.59
0.26
0.27
0.21
0.66
0.27
0.53
1.16
0.57
0.60
f1 (ppm)

13C NMR 6e

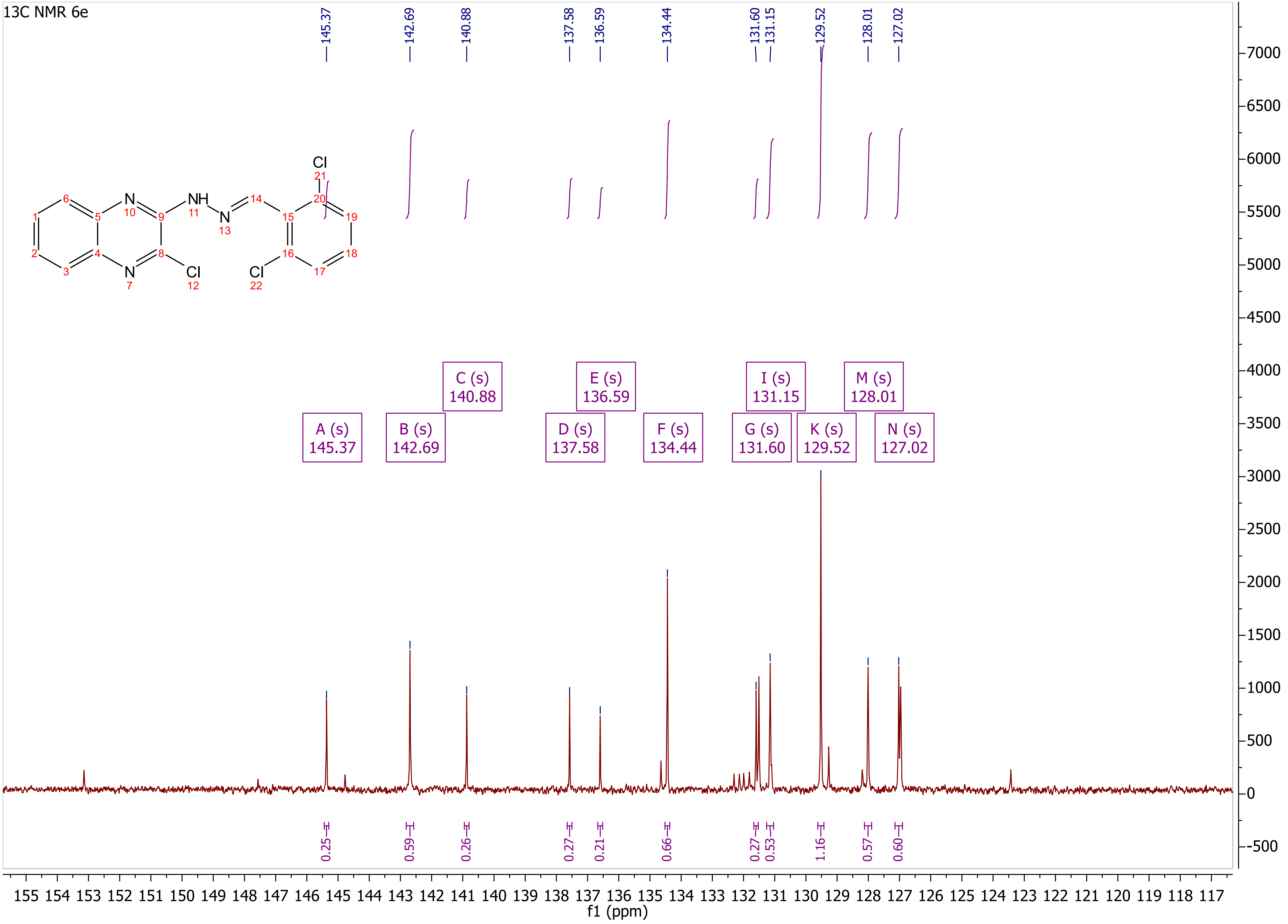

145.37
142.69
140.88
137.58
136.59
134.44
131.60
131.15
129.52
128.01
127.02
A (s) 145.37
B (s) 142.69
C (s) 140.88
D (s) 137.58
E (s) 136.59
F (s) 134.44
G (s) 131.60
I (s) 131.15
K (s) 129.52
M (s) 128.01
N (s) 127.02
0.25
0.59
0.26
0.27
0.21
0.66
0.27
0.53
1.16
0.57
0.60
NH
N
N
N
Cl
Cl
Cl
f1 (ppm)

IR of comp. 6f

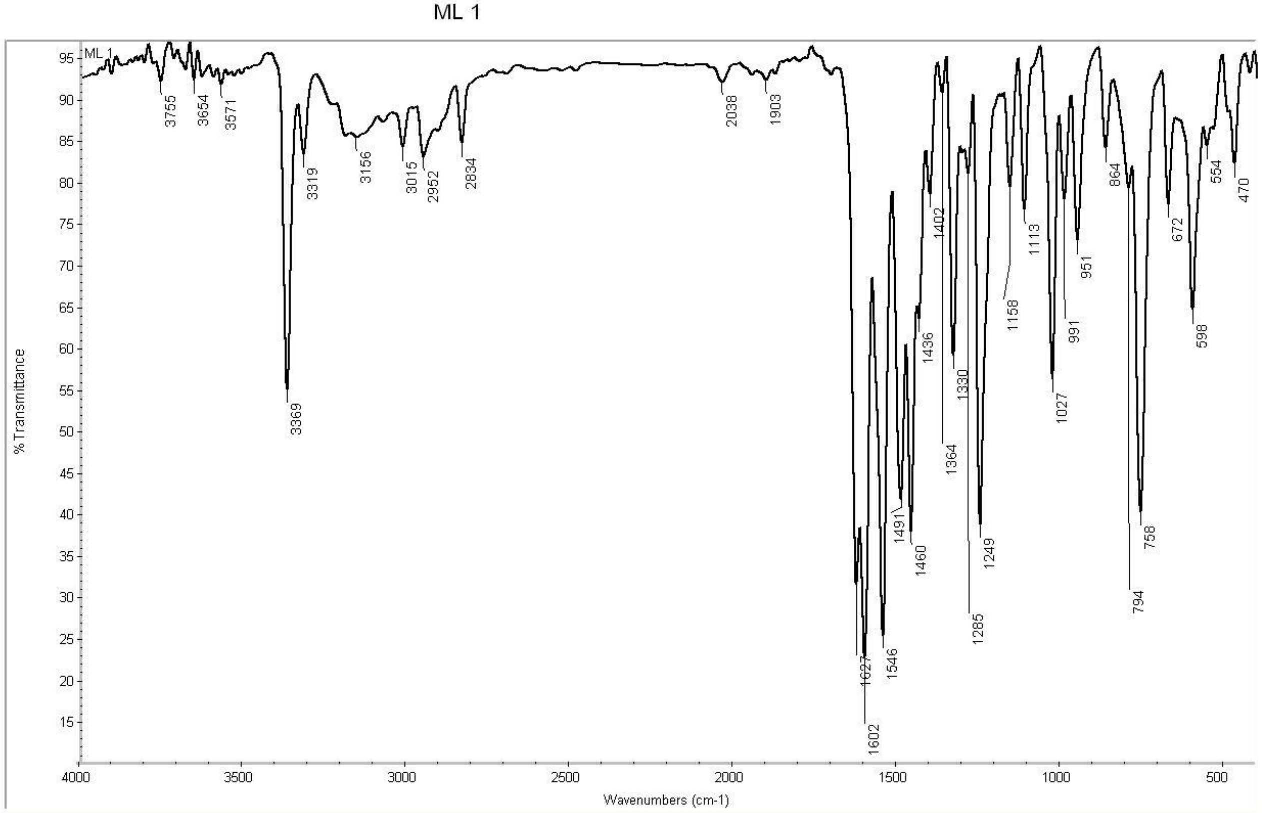
ML 1
ML 1
% Transmittance
Wavenumbers (cm-1)
3755
3654
3571
3369
3319
3156
3015
2952
2834
2038
1903
1627
1602
1546
1491
1460
1436
1402
1364
1330
1285
1249
1158
1113
1027
991
951
864
794
758
672
598
554
470

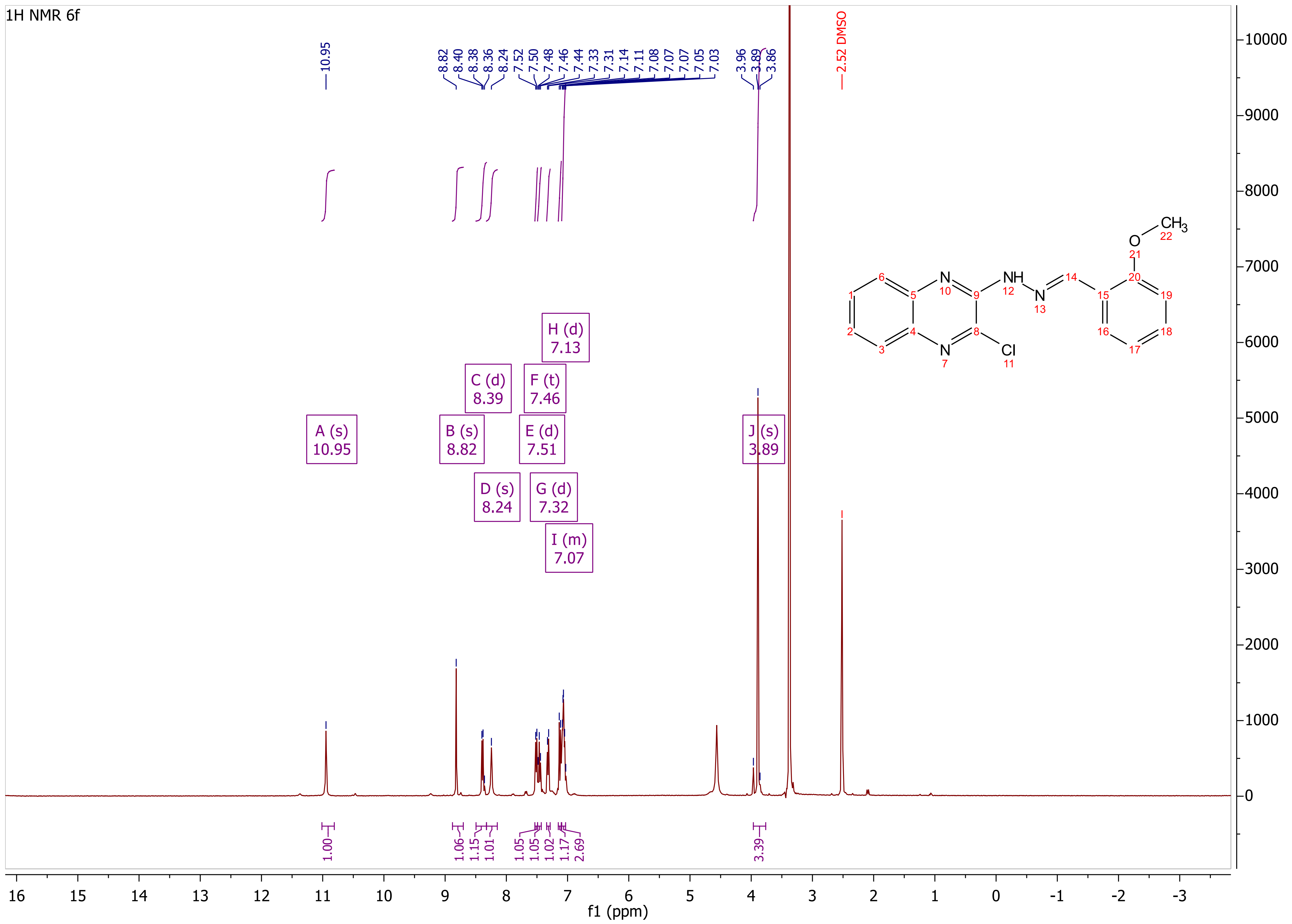
1H NMR 6f
10.95
8.82
8.40
8.38
8.36
8.24
7.52
7.50
7.48
7.46
7.44
7.33
7.31
7.14
7.11
7.08
7.07
7.07
7.05
7.03
3.96
3.89
3.86
2.52 DMSO
A (s) 10.95
B (s) 8.82
C (d) 8.39
D (s) 8.24
E (d) 7.51
F (t) 7.46
G (d) 7.32
H (d) 7.13
I (m) 7.07
J (s) 3.89
1.00
1.06
1.15
1.01
1.05
1.05
1.02
1.17
2.69
3.39
f1 (ppm)
NH
N
N
Cl
O
CH3

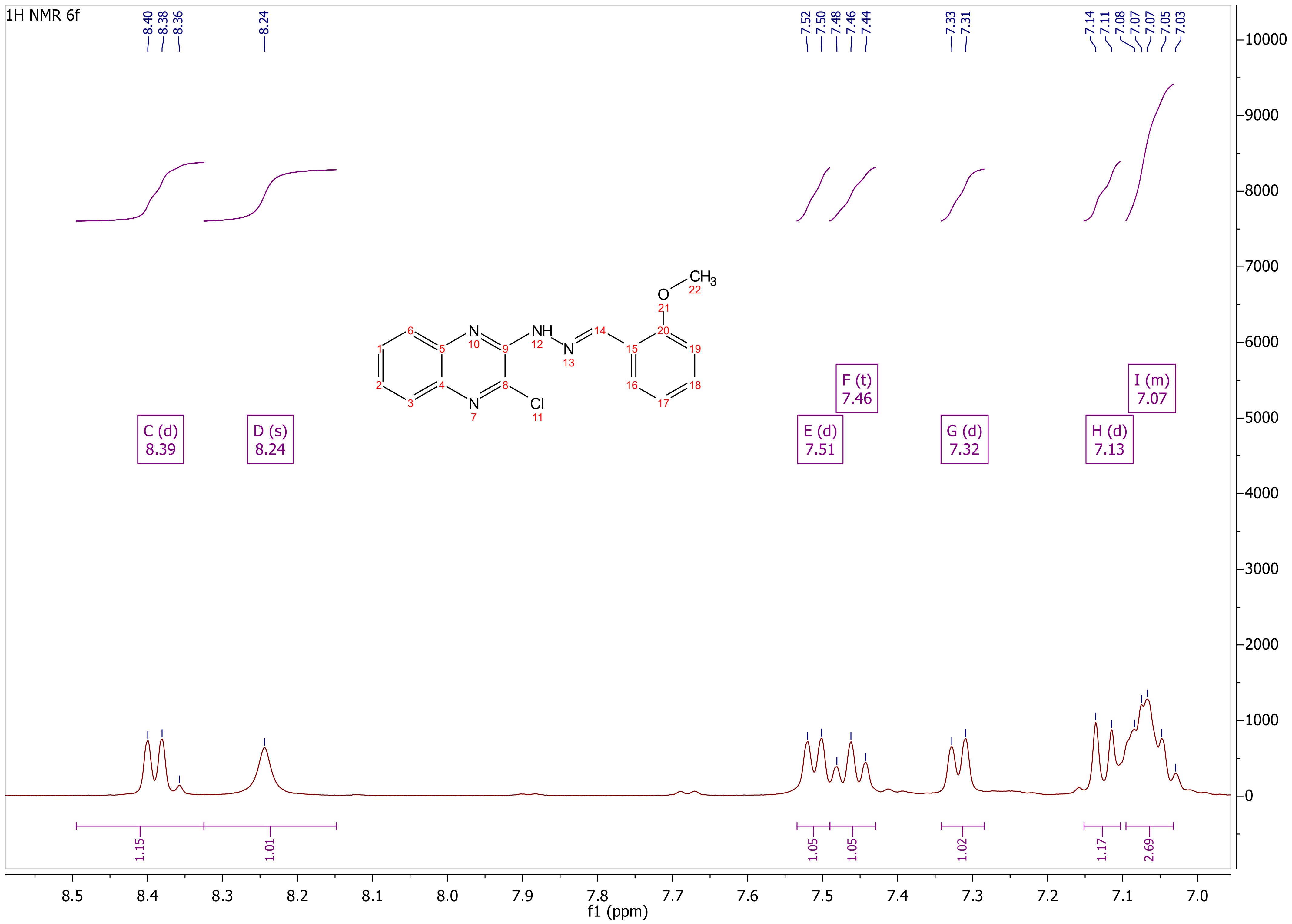

1H NMR 6f
8.40
8.38
8.36
8.24
7.52
7.50
7.48
7.46
7.44
7.33
7.31
7.14
7.11
7.08
7.07
7.07
7.05
7.03
C (d)
8.39
D (s)
8.24
E (d)
7.51
F (t)
7.46
G (d)
7.32
H (d)
7.13
I (m)
7.07
1.15
1.01
1.05
1.05
1.02
1.17
2.69
f1 (ppm)

IBRAHIM-HASSAN-6F #129 RT: 2.18 AV: 1 SB: 2 4.45 , 4.45 NL: 1.31E3
T: {0,0} + c EI Full ms [40.00-1000.00]

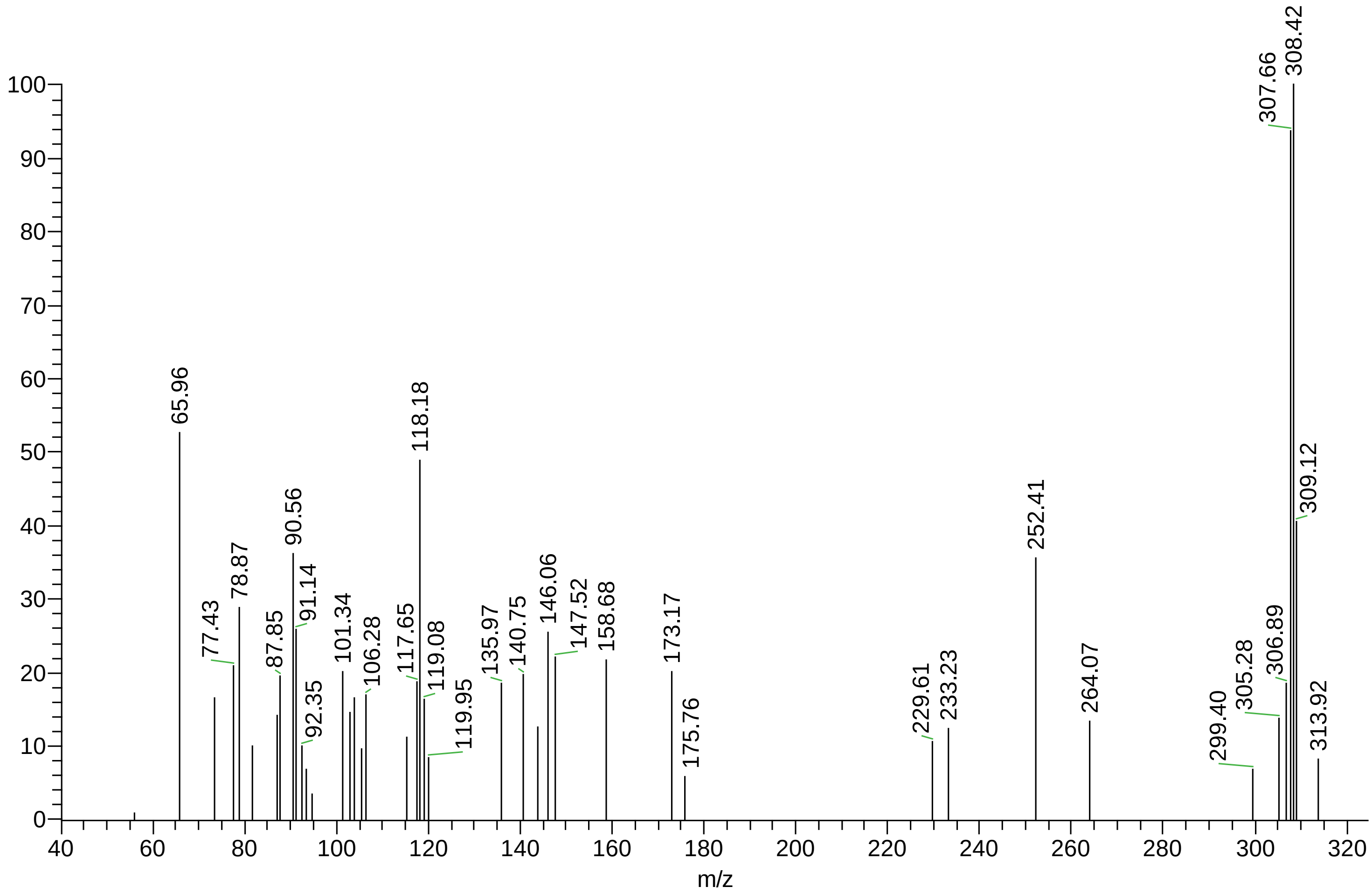

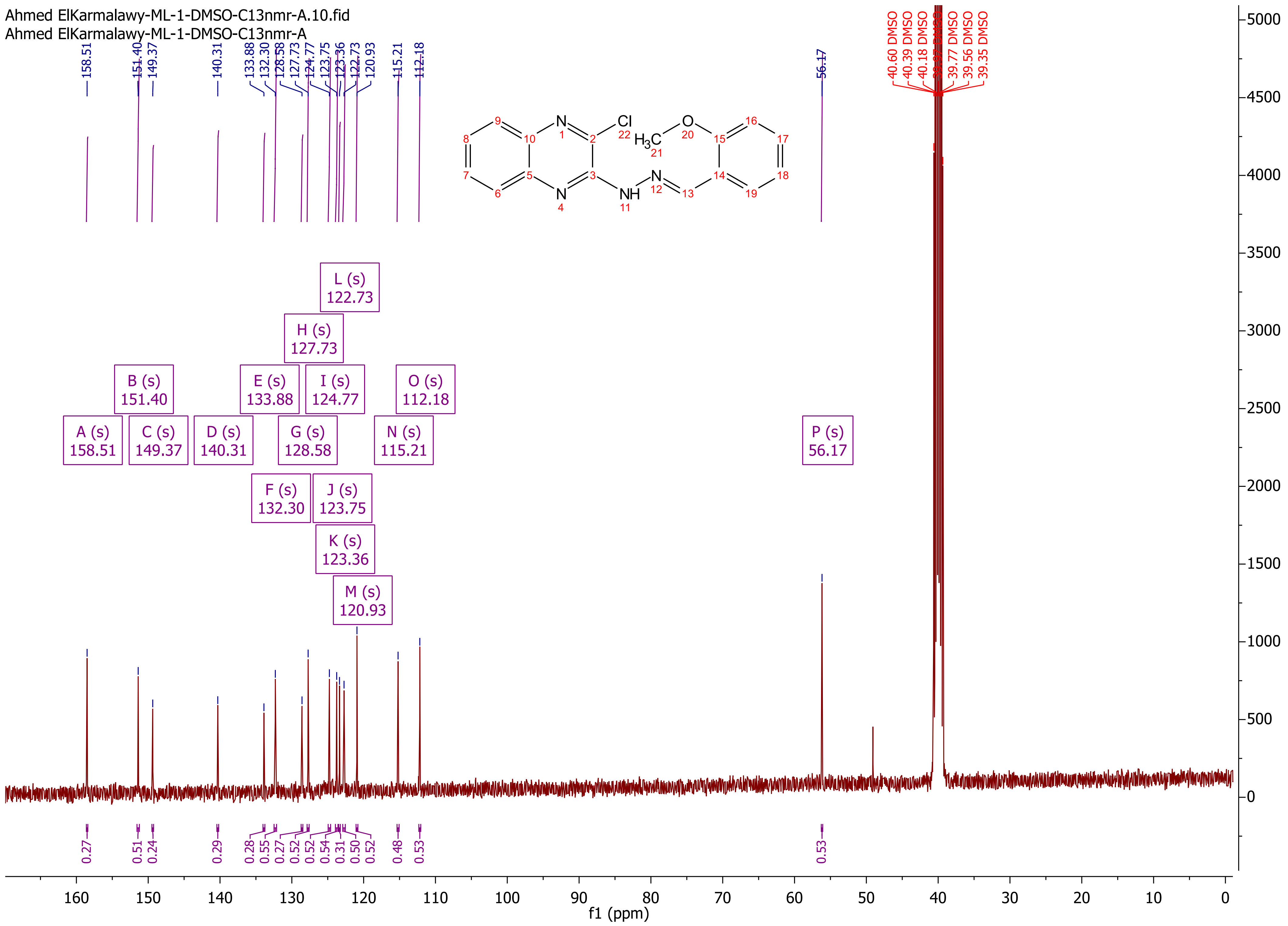
Ahmed ElKarmalawy-ML-1-DMSO-C13nmr-A.10.fid
Ahmed ElKarmalawy-ML-1-DMSO-C13nmr-A
158.51
151.40
149.37
140.31
133.88
132.30
128.58
127.73
124.77
123.75
123.36
122.73
120.93
115.21
112.18
56.17
40.60 DMSO
40.39 DMSO
40.18 DMSO
39.77 DMSO
39.56 DMSO
39.35 DMSO
N
Cl
H3C
O
NH
N
A (s) 158.51
B (s) 151.40
C (s) 149.37
D (s) 140.31
E (s) 133.88
F (s) 132.30
G (s) 128.58
H (s) 127.73
I (s) 124.77
J (s) 123.75
K (s) 123.36
L (s) 122.73
M (s) 120.93
N (s) 115.21
O (s) 112.18
P (s) 56.17
0.27
0.51
0.24
0.29
0.28
0.55
0.27
0.52
0.52
0.54
0.31
0.50
0.52
0.48
0.53
0.53
f1 (ppm)

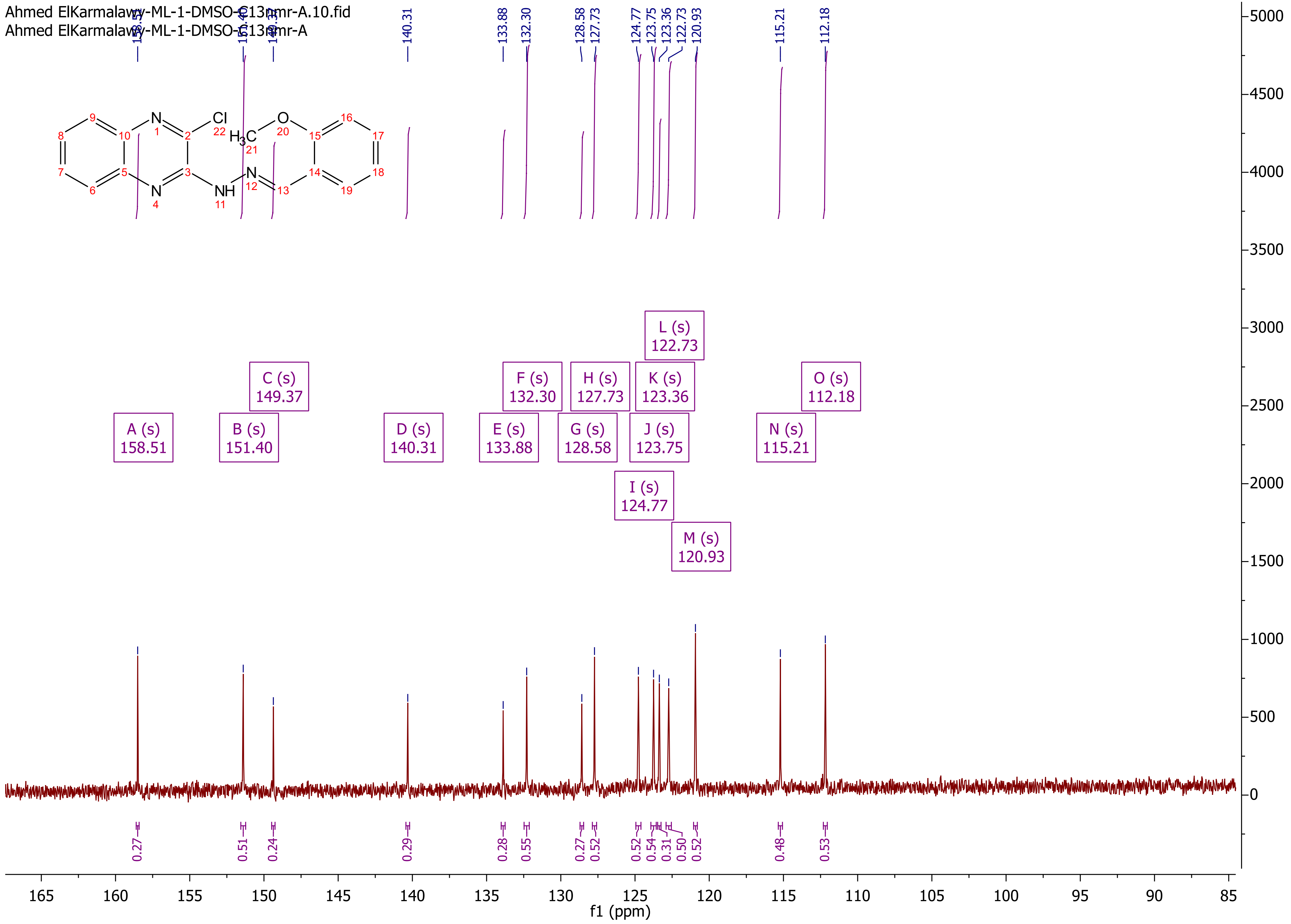
Ahmed ElKarmalawy-ML-1-DMSO-C13nmr-A.10.fid
Ahmed ElKarmalawy-ML-1-DMSO-C13nmr-A
140.31
133.88
132.30
128.58
127.73
124.77
123.75
123.36
122.73
120.93
115.21
112.18
A (s) 158.51
B (s) 151.40
C (s) 149.37
D (s) 140.31
E (s) 133.88
F (s) 132.30
G (s) 128.58
H (s) 127.73
I (s) 124.77
J (s) 123.75
K (s) 123.36
L (s) 122.73
M (s) 120.93
N (s) 115.21
O (s) 112.18
f1 (ppm)

Ahmed ElKarmalawy-ML-1-DMSO-C13nmr-A.10.fid
Ahmed ElKarmalawy-ML-1-DMSO-C13nmr-A

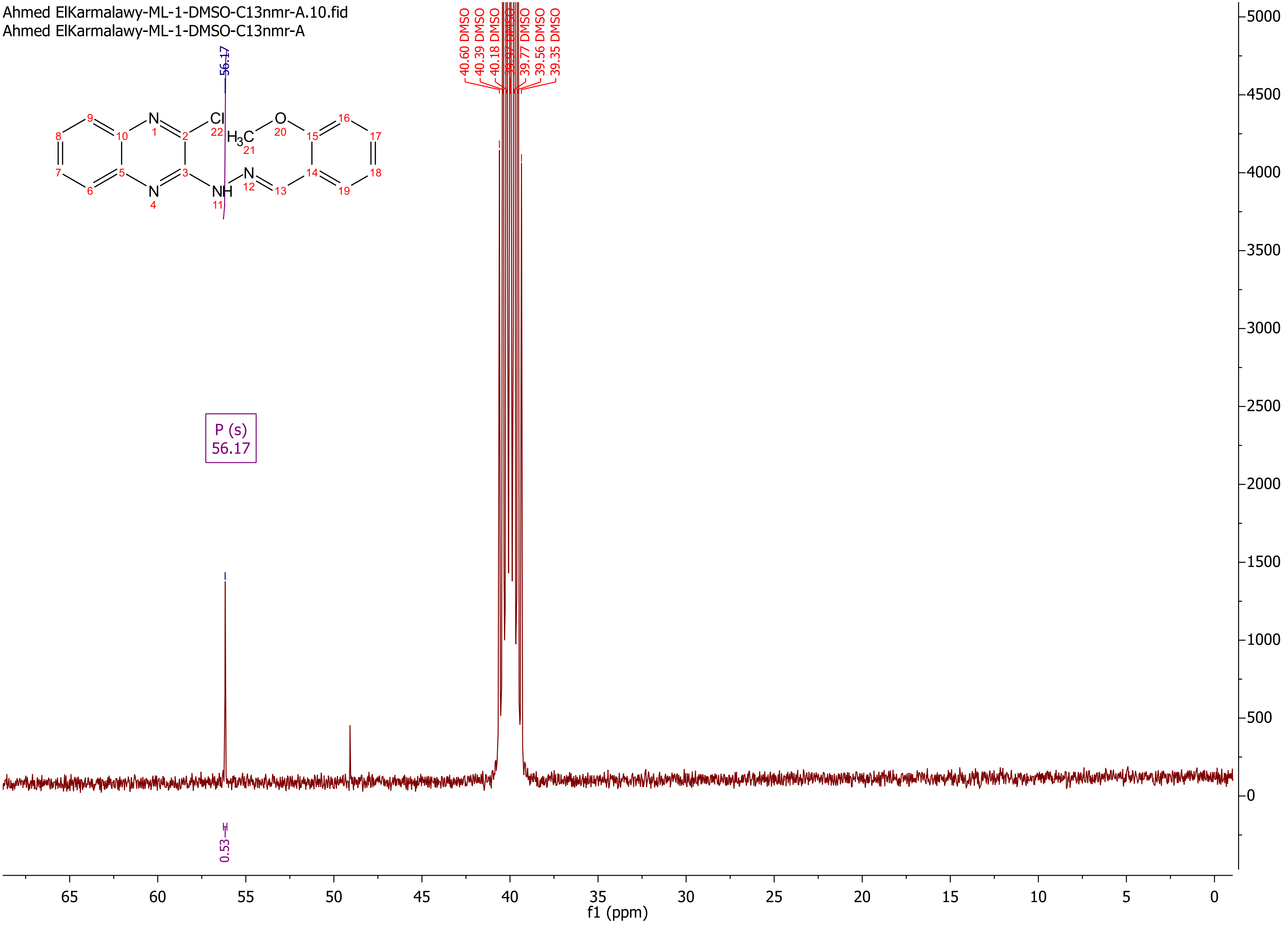

IR of compound 6g

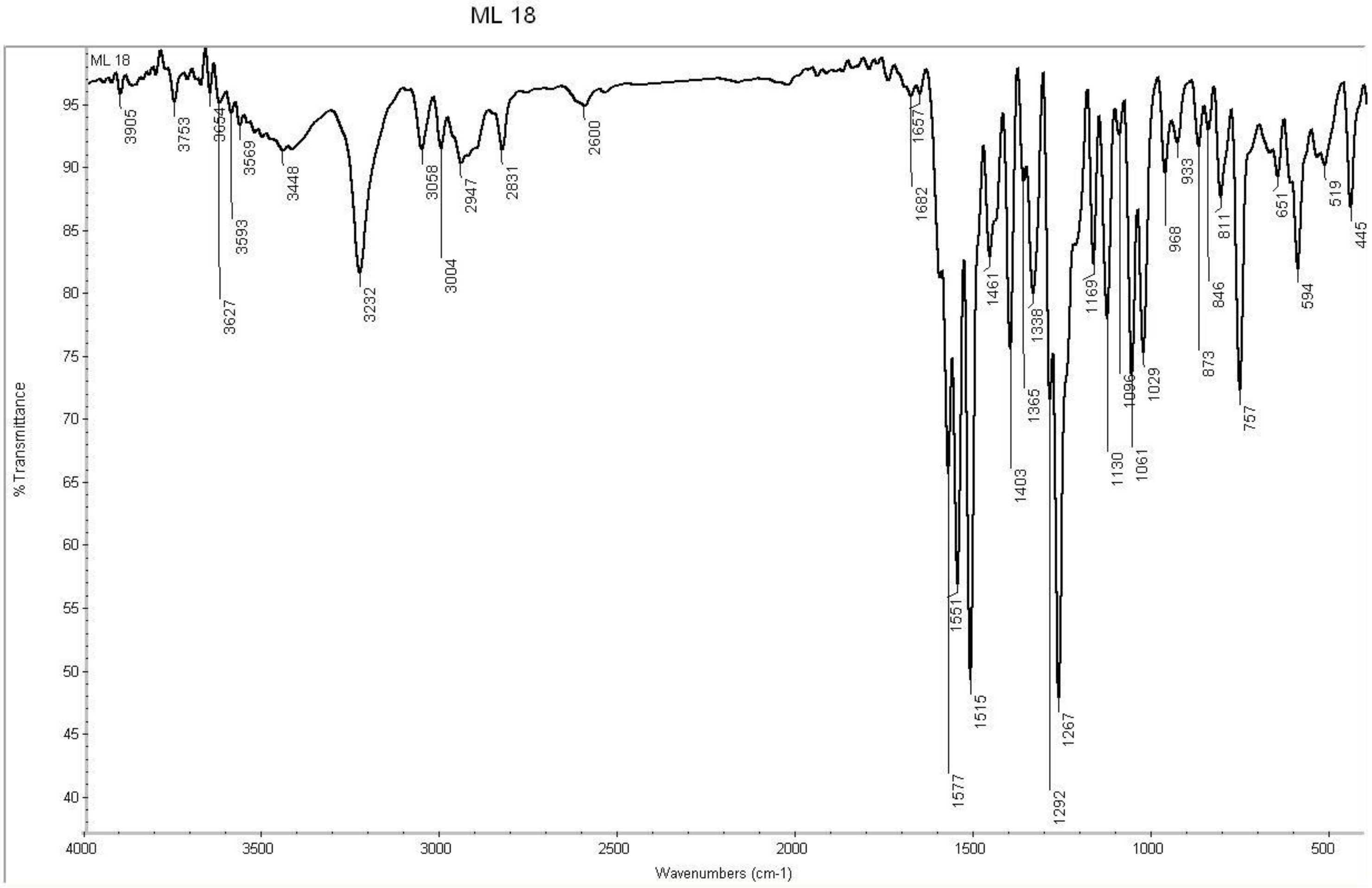

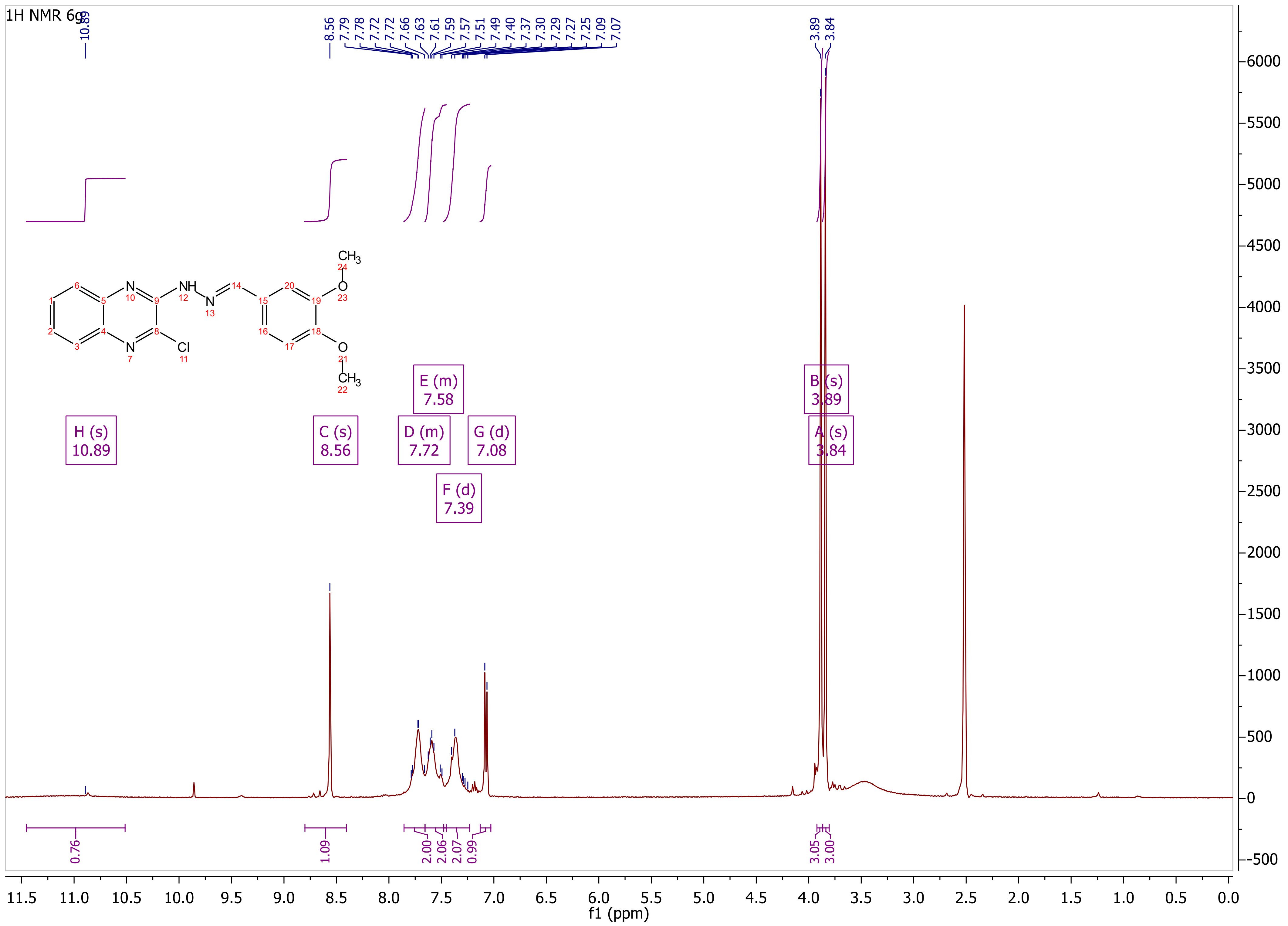
1H NMR 6g
10.89
8.56
7.79
7.78
7.72
7.72
7.66
7.63
7.61
7.59
7.57
7.51
7.49
7.40
7.37
7.30
7.29
7.27
7.25
7.09
7.07
3.89
3.84
H (s)
10.89
C (s)
8.56
D (m)
7.72
E (m)
7.58
F (d)
7.39
G (d)
7.08
B (s)
3.89
A (s)
3.84
0.76
1.09
2.00
2.06
2.07
0.99
3.05
3.00
f1 (ppm)

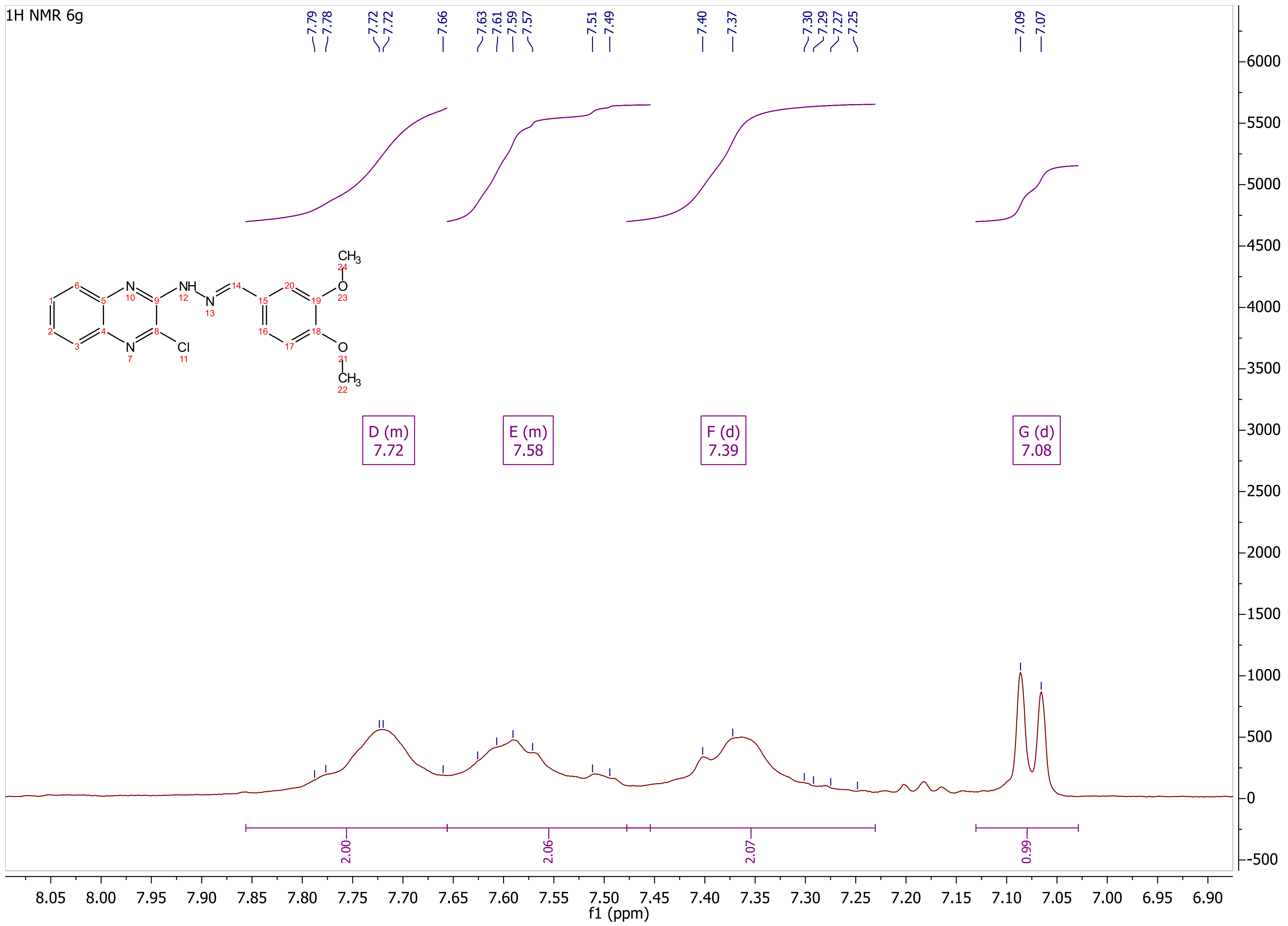

1H NMR 6g
7.79
7.78
7.72
7.72
7.66
7.63
7.61
7.59
7.57
7.51
7.49
7.40
7.37
7.30
7.29
7.27
7.25
7.09
7.07
D (m)
7.72
E (m)
7.58
F (d)
7.39
G (d)
7.08
2.00
2.06
2.07
0.99
f1 (ppm)

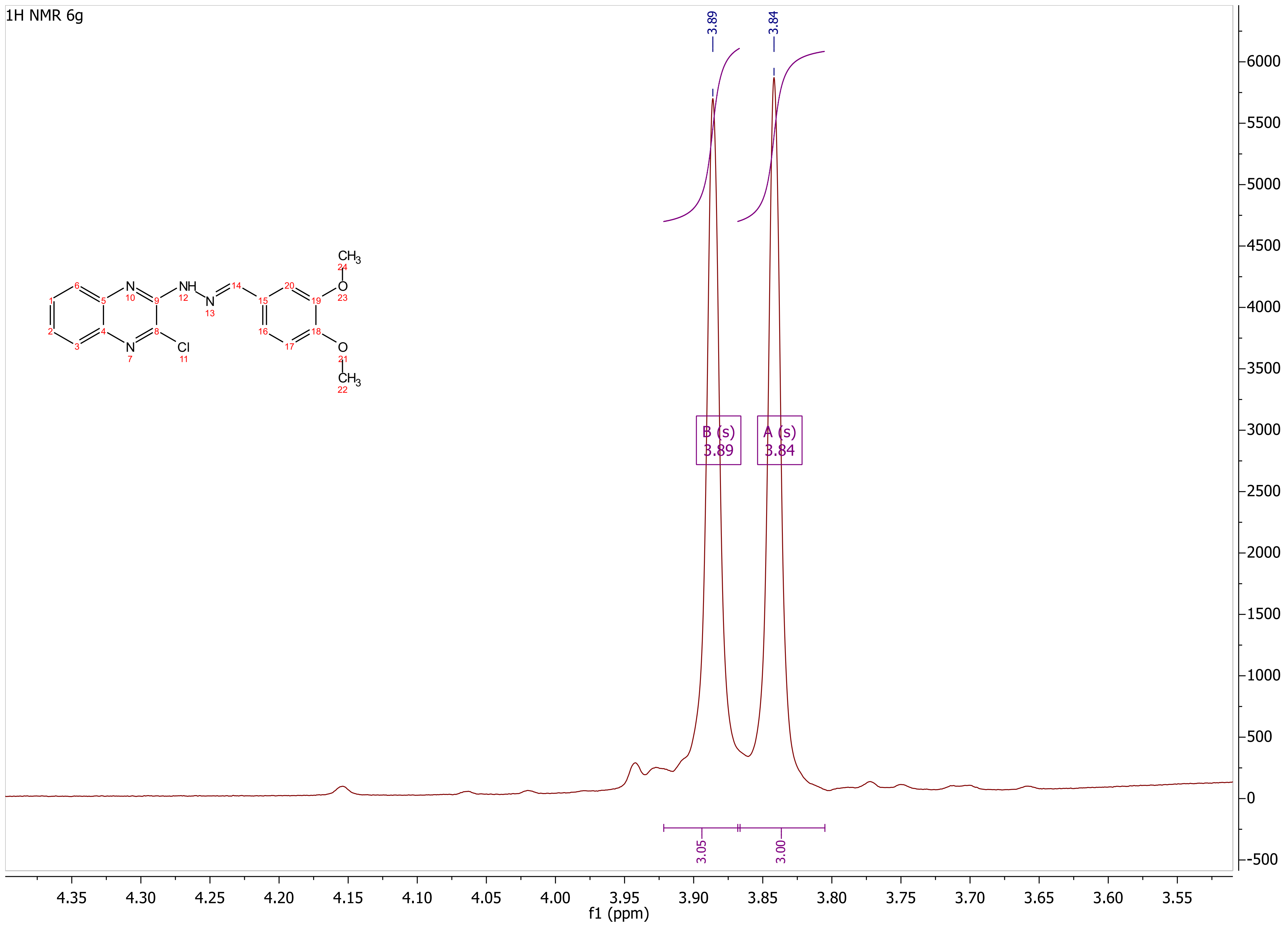

1H NMR 6g
3.89
3.84
CH3
O
O
CH3
NH
N
N
N
N
Cl
B (s)
3.89
A (s)
3.84
3.05
3.00
f1 (ppm)

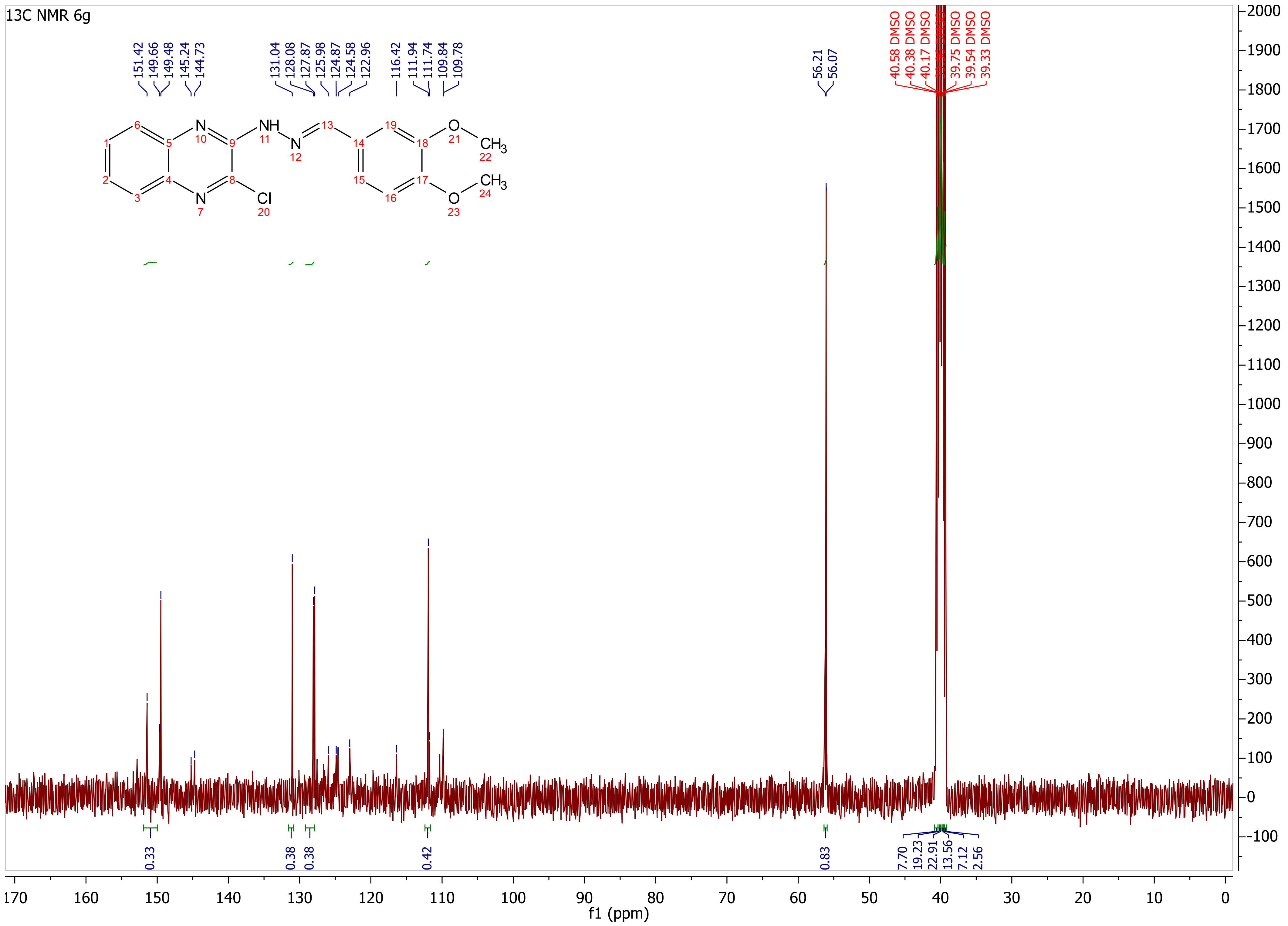

13C NMR 6g
151.42
149.66
149.48
145.24
144.73
131.04
128.08
127.87
125.98
124.87
124.58
122.96
116.42
111.94
111.74
109.84
109.78
56.21
56.07
40.58 DMSO
40.38 DMSO
40.17 DMSO
39.75 DMSO
39.54 DMSO
39.33 DMSO
0.33
0.38
0.38
0.42
0.83
7.70
19.23
22.91
13.56
7.12
2.56
f1 (ppm)

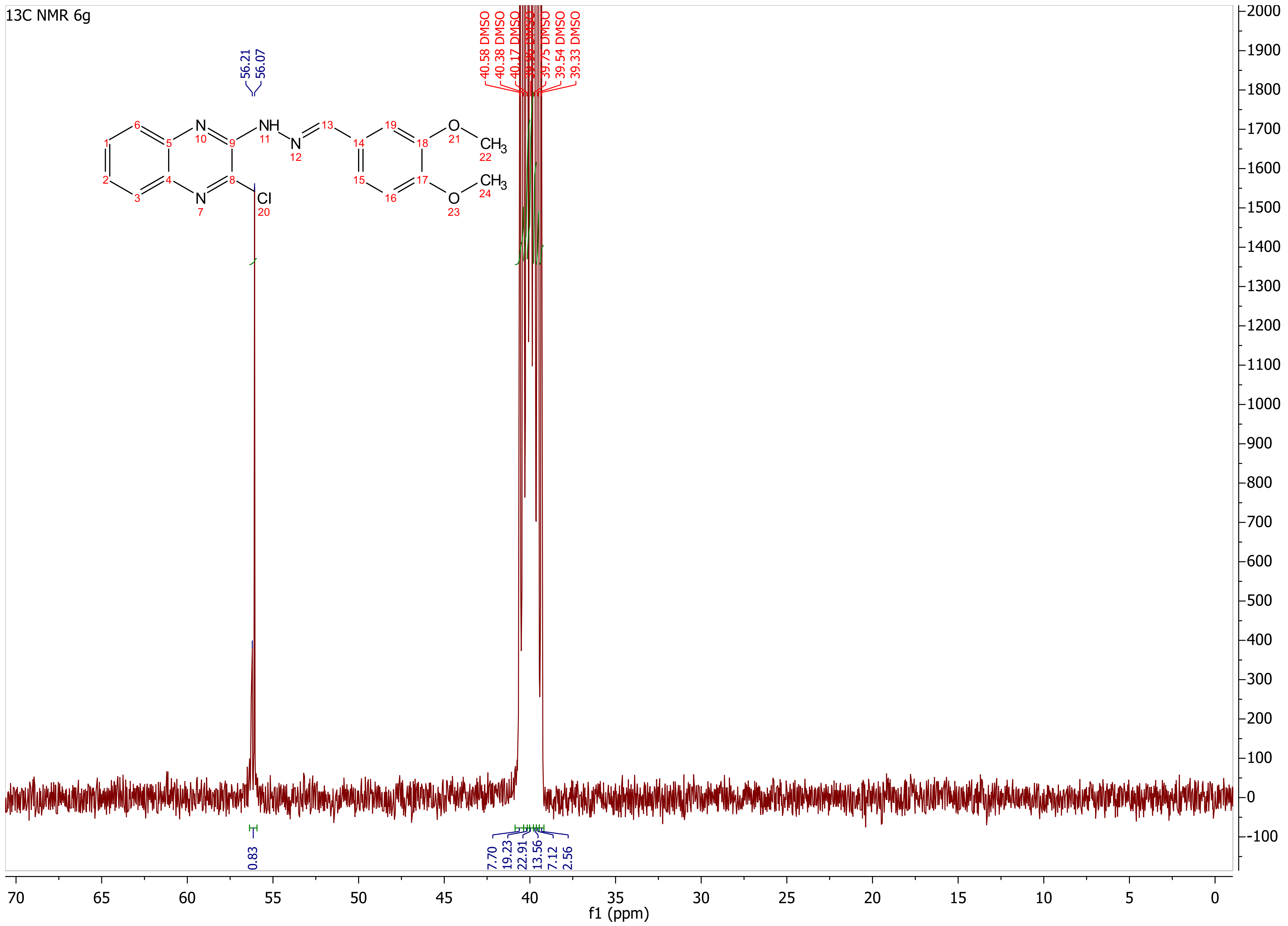

13C NMR 6g
56.21
56.07
40.58 DMSO
40.38 DMSO
40.17 DMSO
39.96 DMSO
39.75 DMSO
39.54 DMSO
39.33 DMSO
N
NH
N
O
CH3
CH3
O
N
Cl
0.83
7.70
19.23
22.91
13.56
7.12
2.56
f1 (ppm)

IR of compound 6h

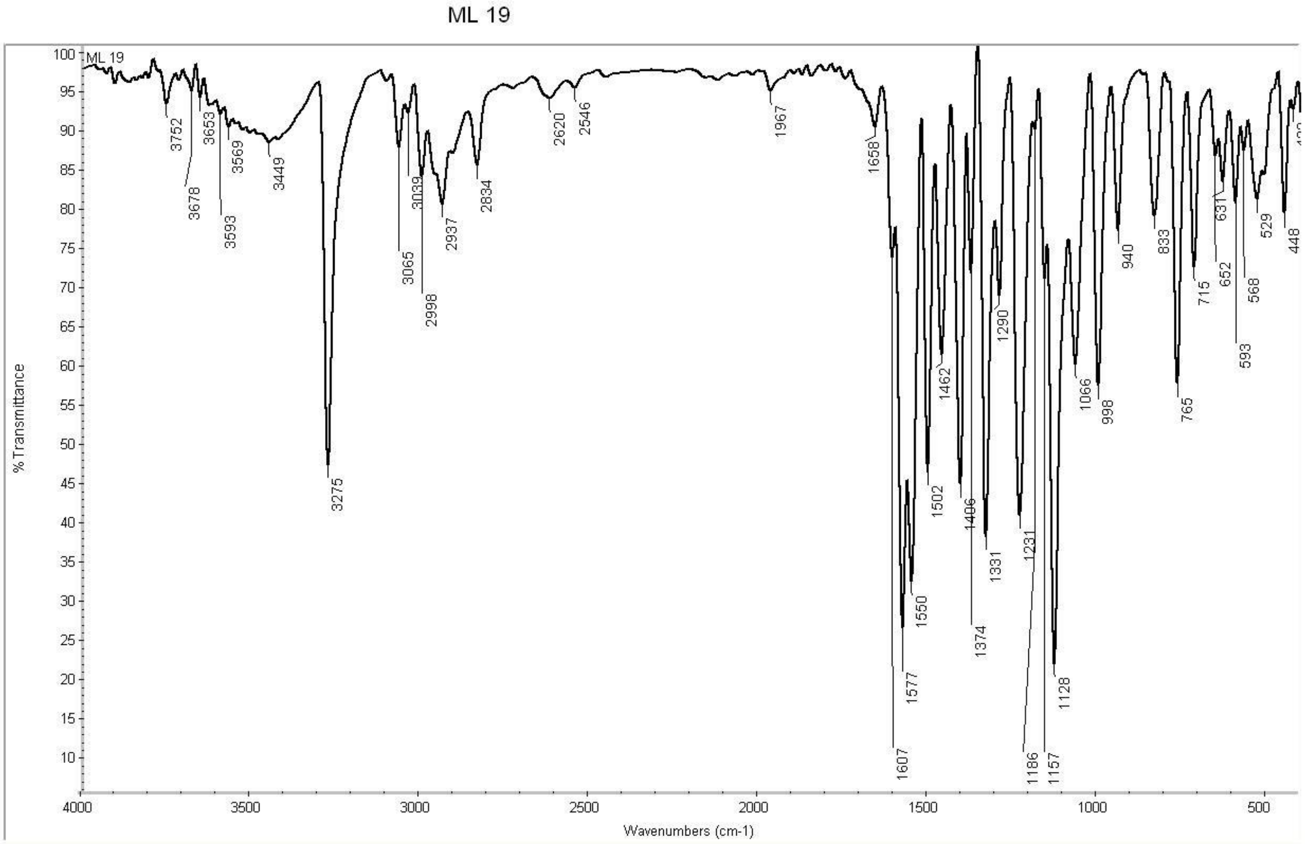

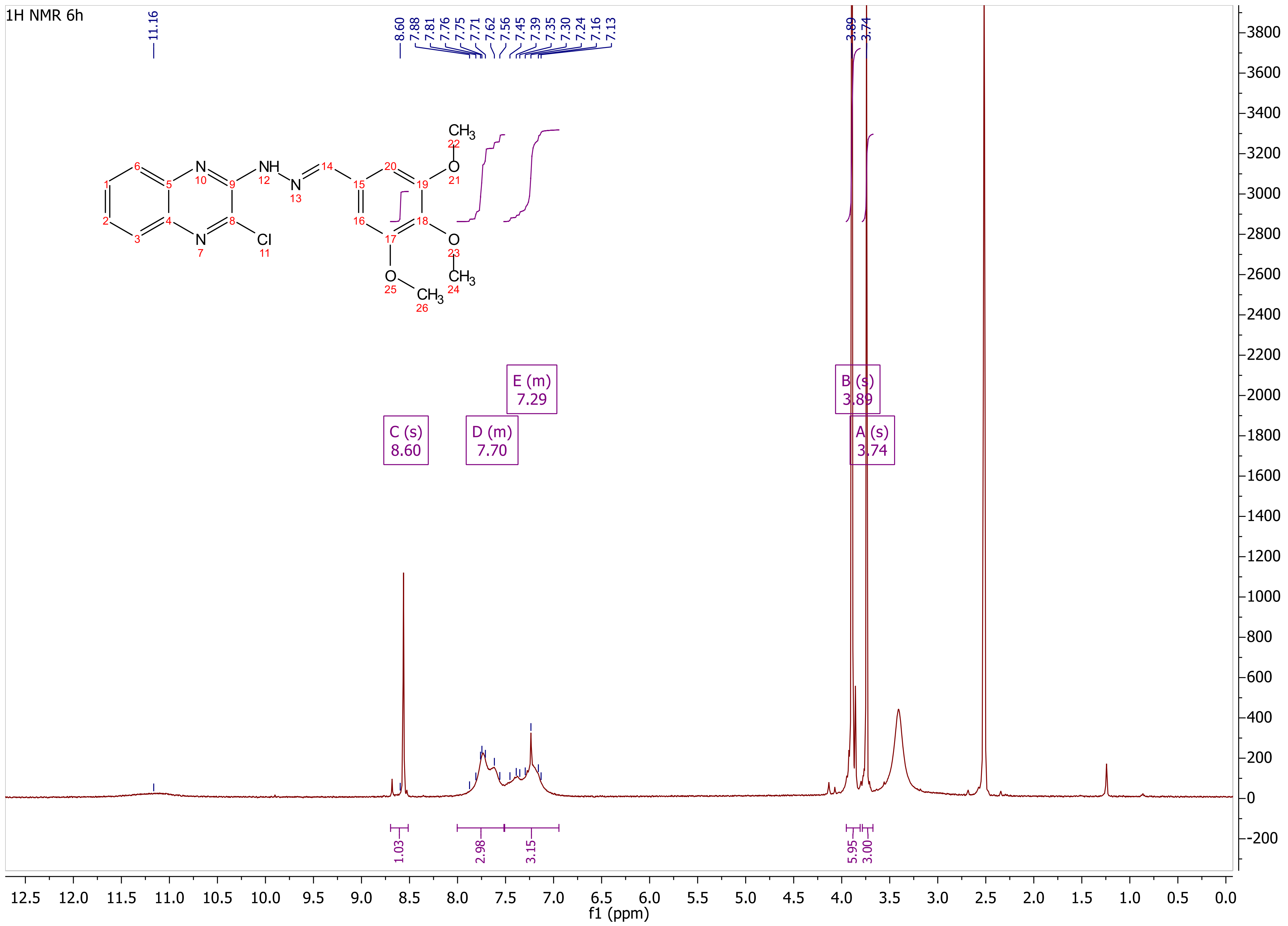
1H NMR 6h
11.16
8.60
7.88
7.81
7.76
7.75
7.71
7.62
7.56
7.45
7.39
7.35
7.30
7.24
7.16
7.13
3.89
3.74
C (s) 8.60
D (m) 7.70
E (m) 7.29
B (s) 3.89
A (s) 3.74
1.03
2.98
3.15
5.95
3.00
f1 (ppm)

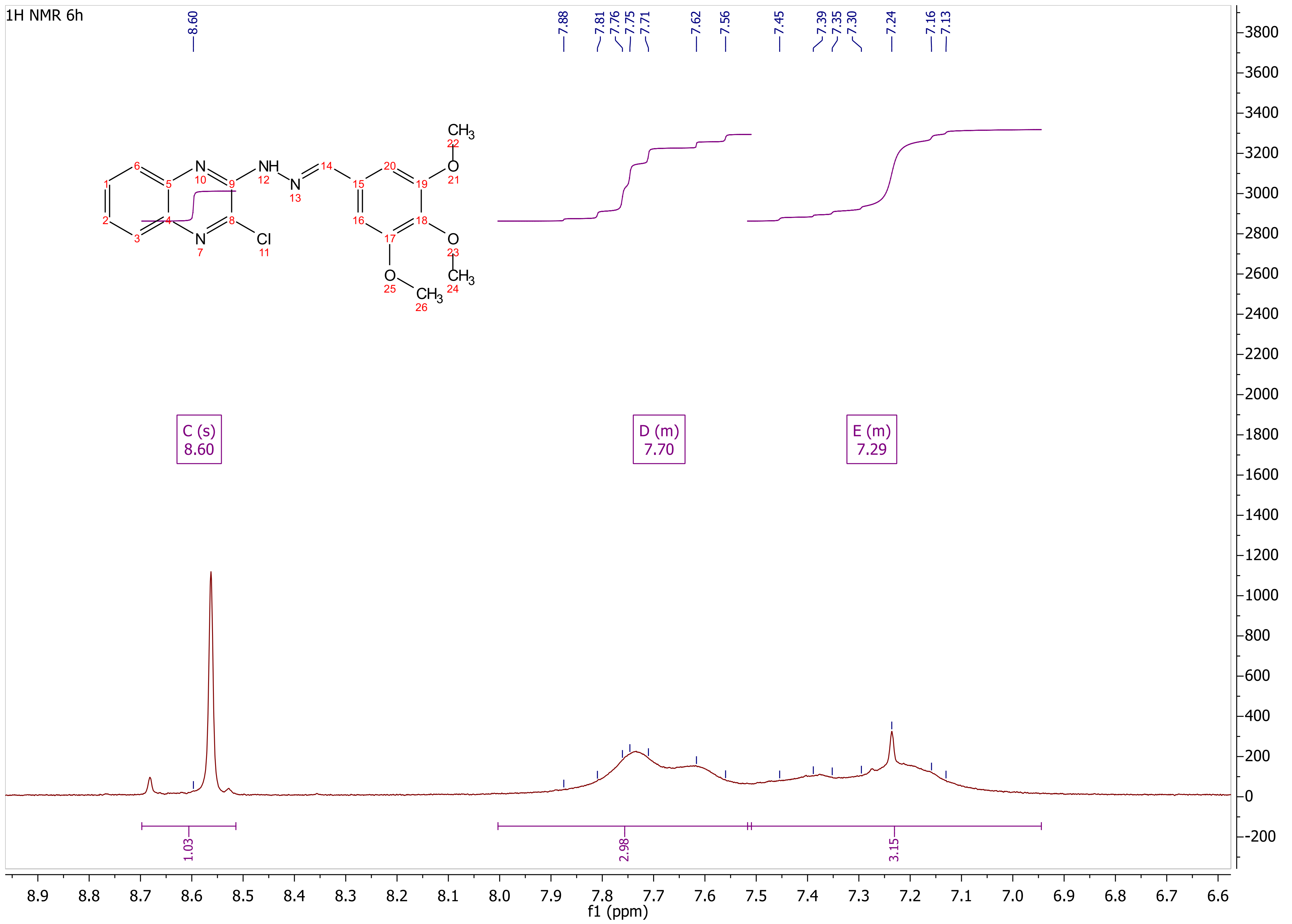
1H NMR 6h
8.60
7.88
7.81
7.76
7.75
7.71
7.62
7.56
7.45
7.39
7.35
7.30
7.24
7.16
7.13
C (s) 8.60
D (m) 7.70
E (m) 7.29
1.03
2.98
3.15
f1 (ppm)

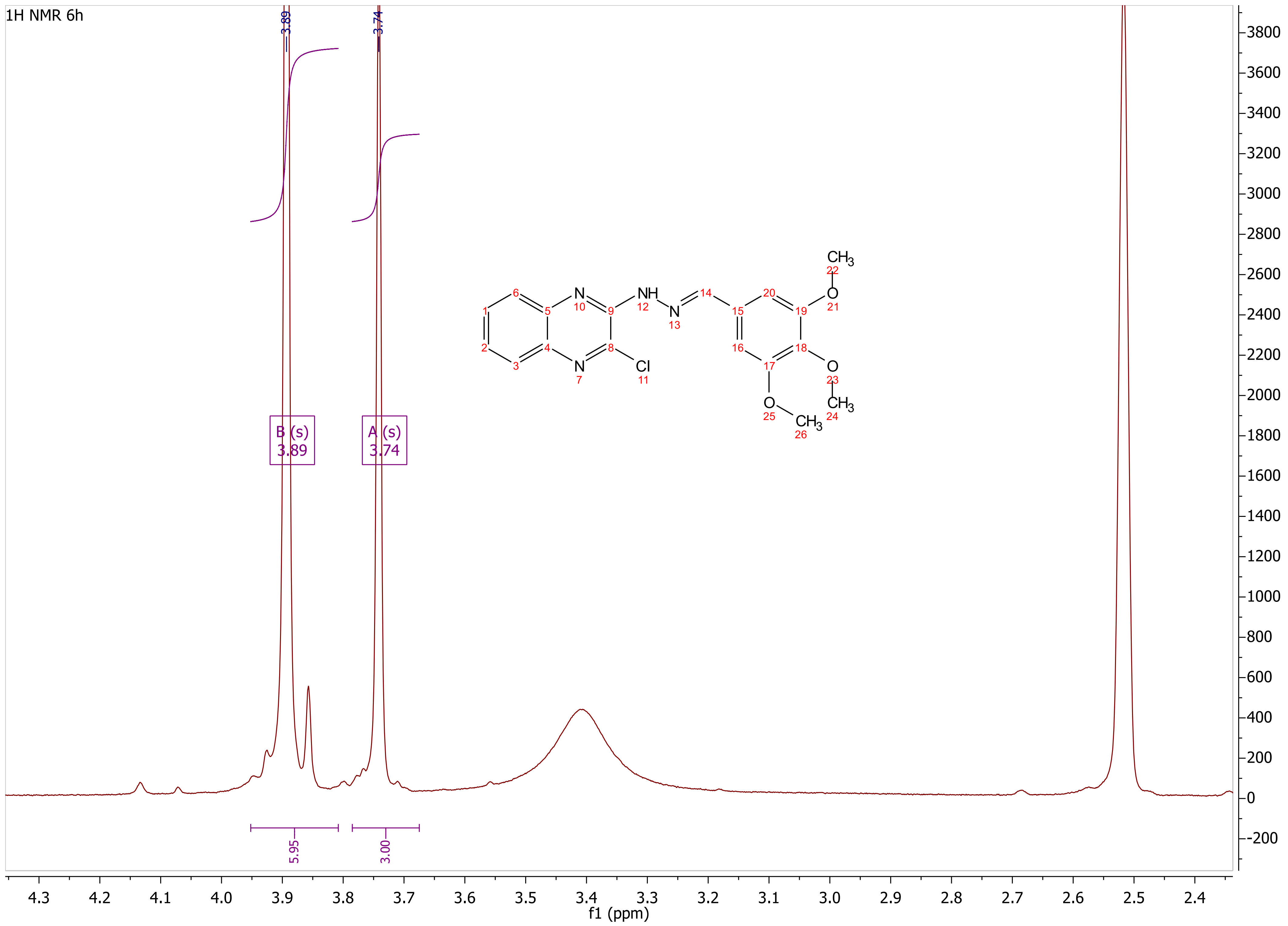

1H NMR 6h
3.89
3.74
B (s)
3.89
A (s)
3.74
5.95
3.00
f1 (ppm)
CH3
O
N
NH
N
Cl

IBRAHIM-HASSAN-6h #153 RT: 2.58 AV: 1 SB: 2 4.45 , 4.45 NL: 5.75E4
T: {0,0} + c EI Full ms [40.00-1000.00]

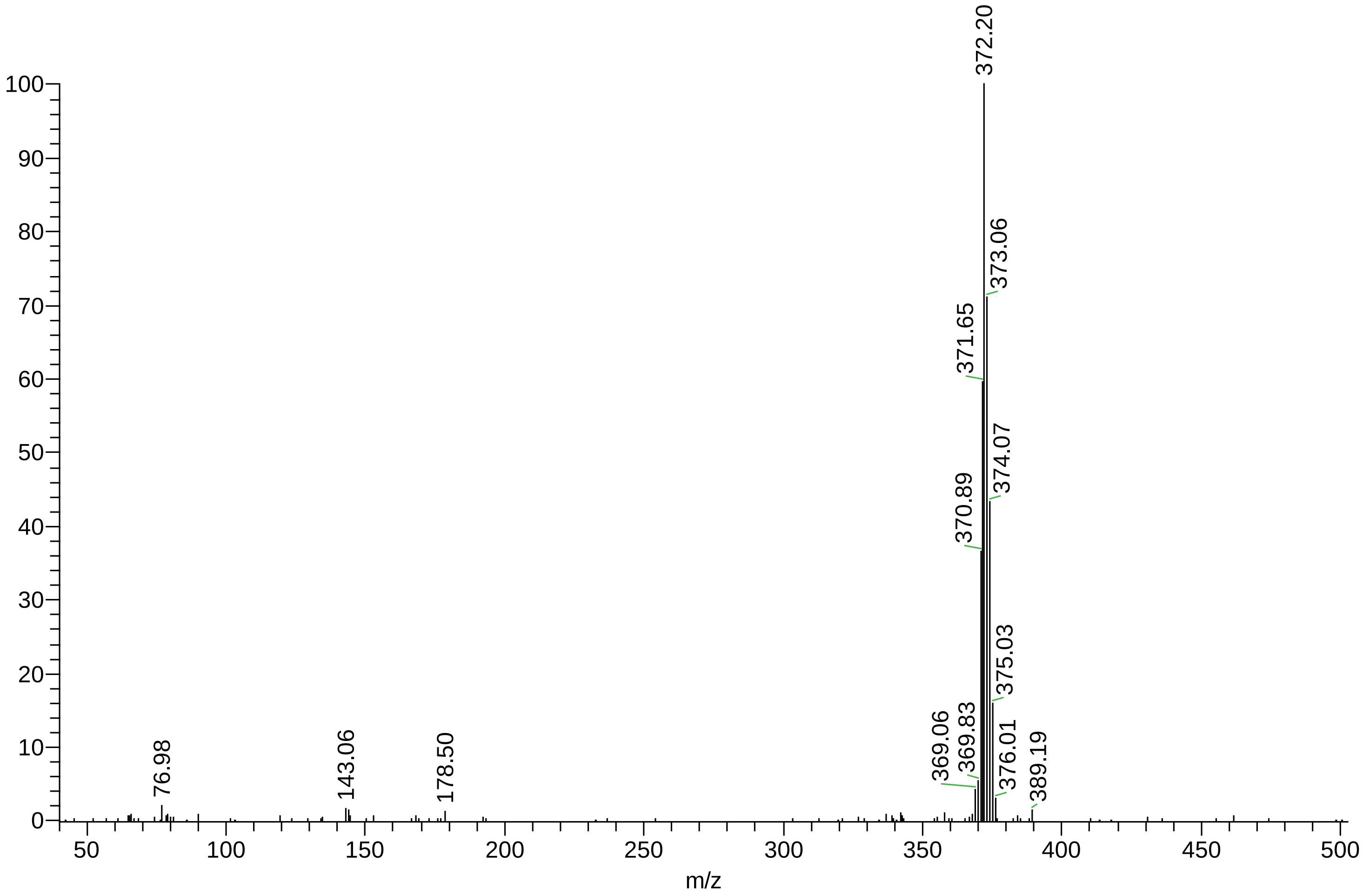

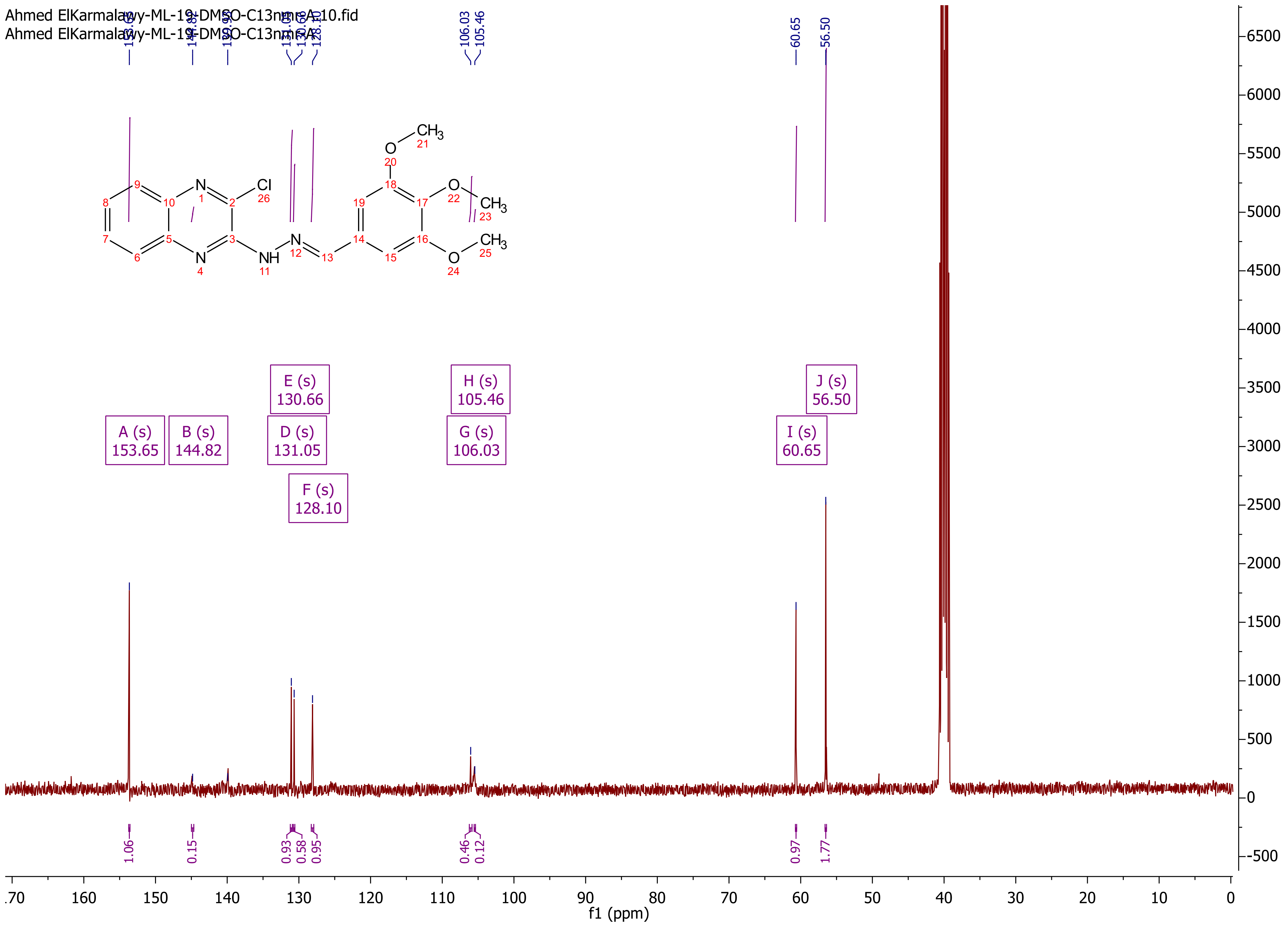

Ahmed ElKarmalawy-ML-19-DMSO-C13nmrA.10.fid
Ahmed ElKarmalawy-ML-19-DMSO-C13nmrA
106.03
105.46
60.65
56.50
A (s)
153.65
B (s)
144.82
D (s)
131.05
E (s)
130.66
F (s)
128.10
G (s)
106.03
H (s)
105.46
I (s)
60.65
J (s)
56.50
1.06
0.15
0.93
0.58
0.95
0.46
0.12
0.97
1.77
f1 (ppm)

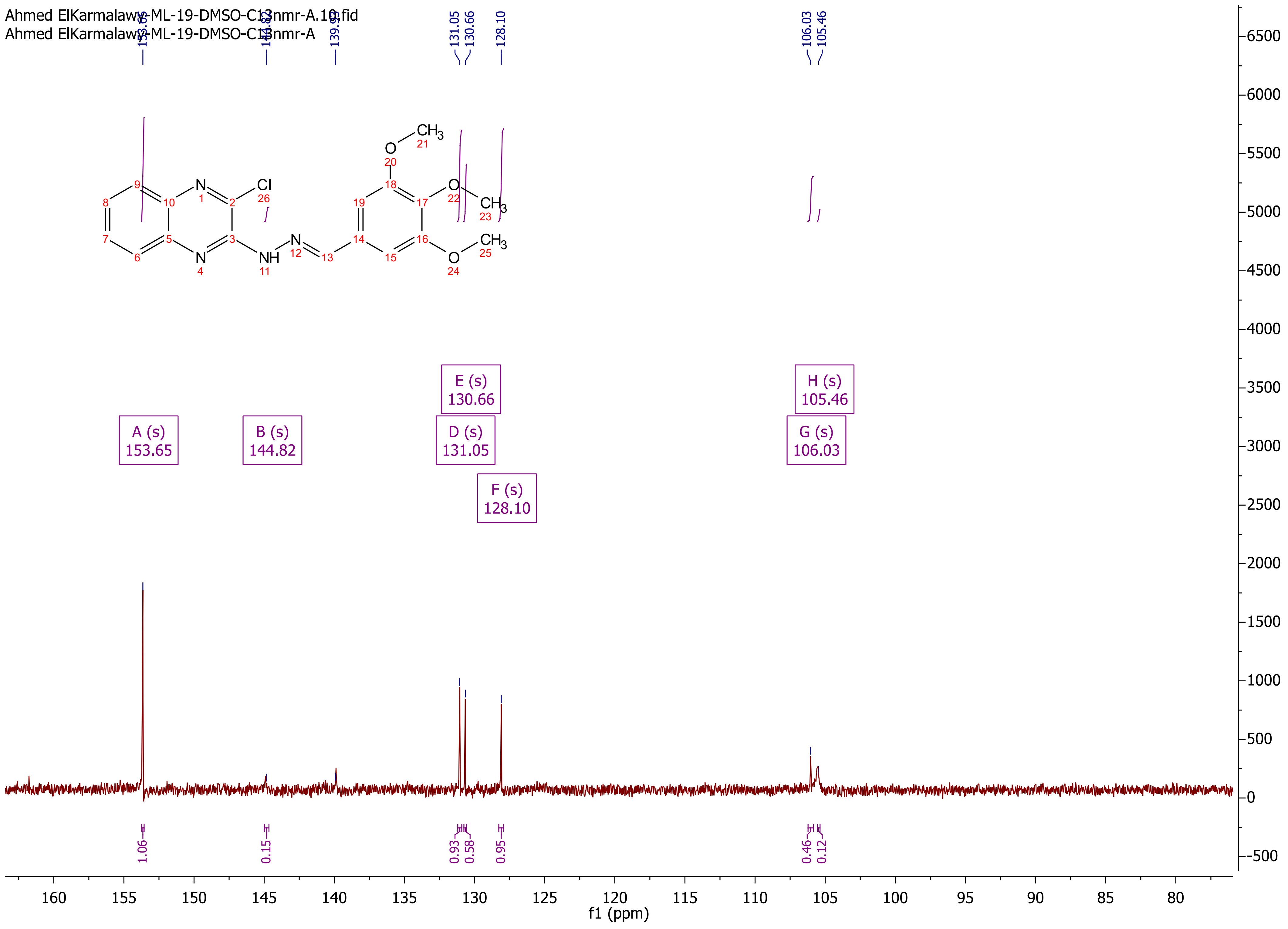

Ahmed ElKarmalawy-ML-19-DMSO-C13nmr-A.10.fid
Ahmed ElKarmalawy-ML-19-DMSO-C13nmr-A
139.92
131.05
130.66
128.10
106.03
105.46
CH3
O
Cl
N
NH
A (s) 153.65
B (s) 144.82
D (s) 131.05
E (s) 130.66
F (s) 128.10
G (s) 106.03
H (s) 105.46
1.06
0.15
0.93
0.58
0.95
0.46
0.12
f1 (ppm)

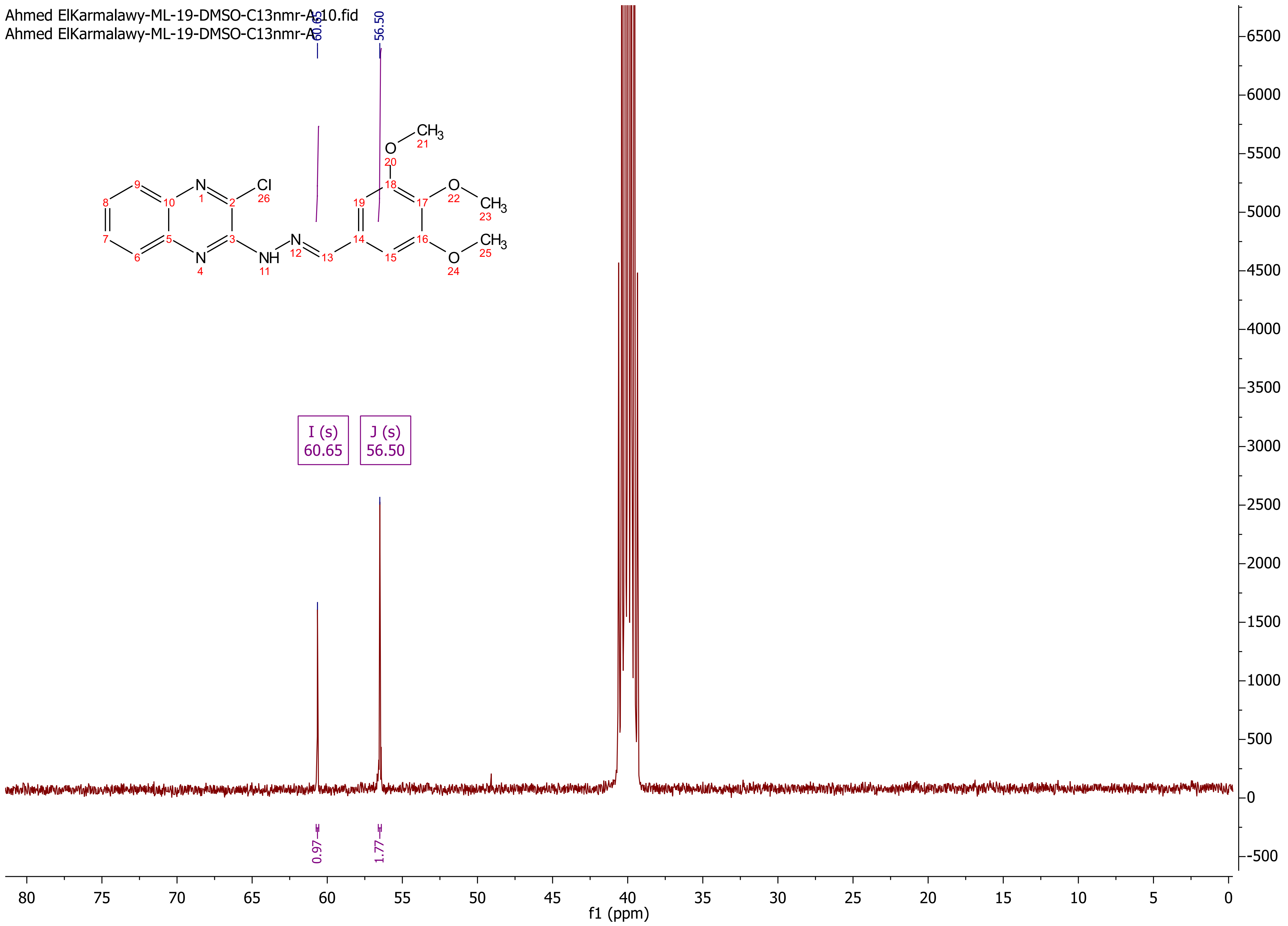
Ahmed ElKarmalawy-ML-19-DMSO-C13nmr-A.10.fid
Ahmed ElKarmalawy-ML-19-DMSO-C13nmr-A
60.65
56.50
I (s)
60.65
J (s)
56.50
0.97
1.77
f1 (ppm)

IR of compound 6i

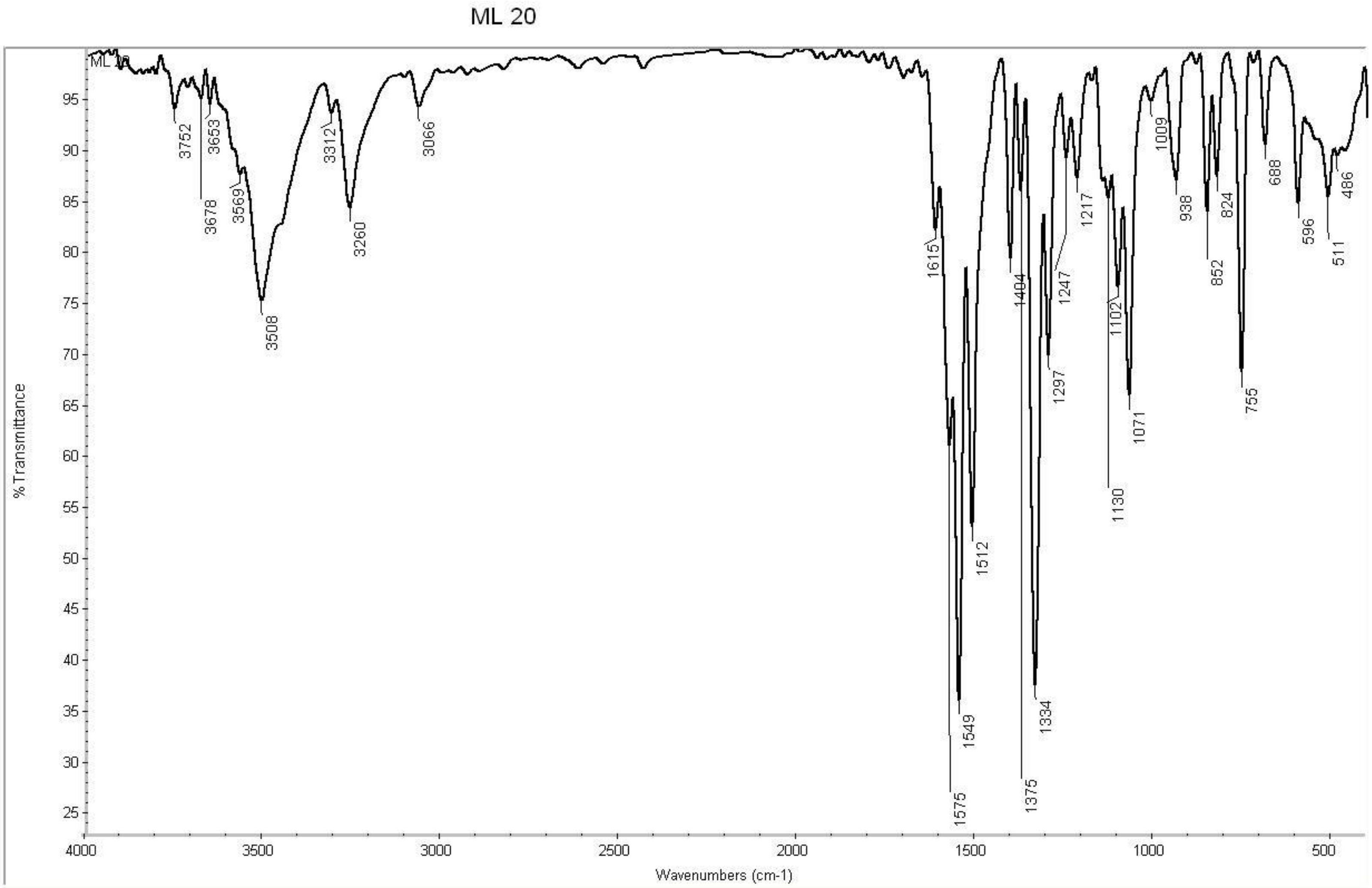

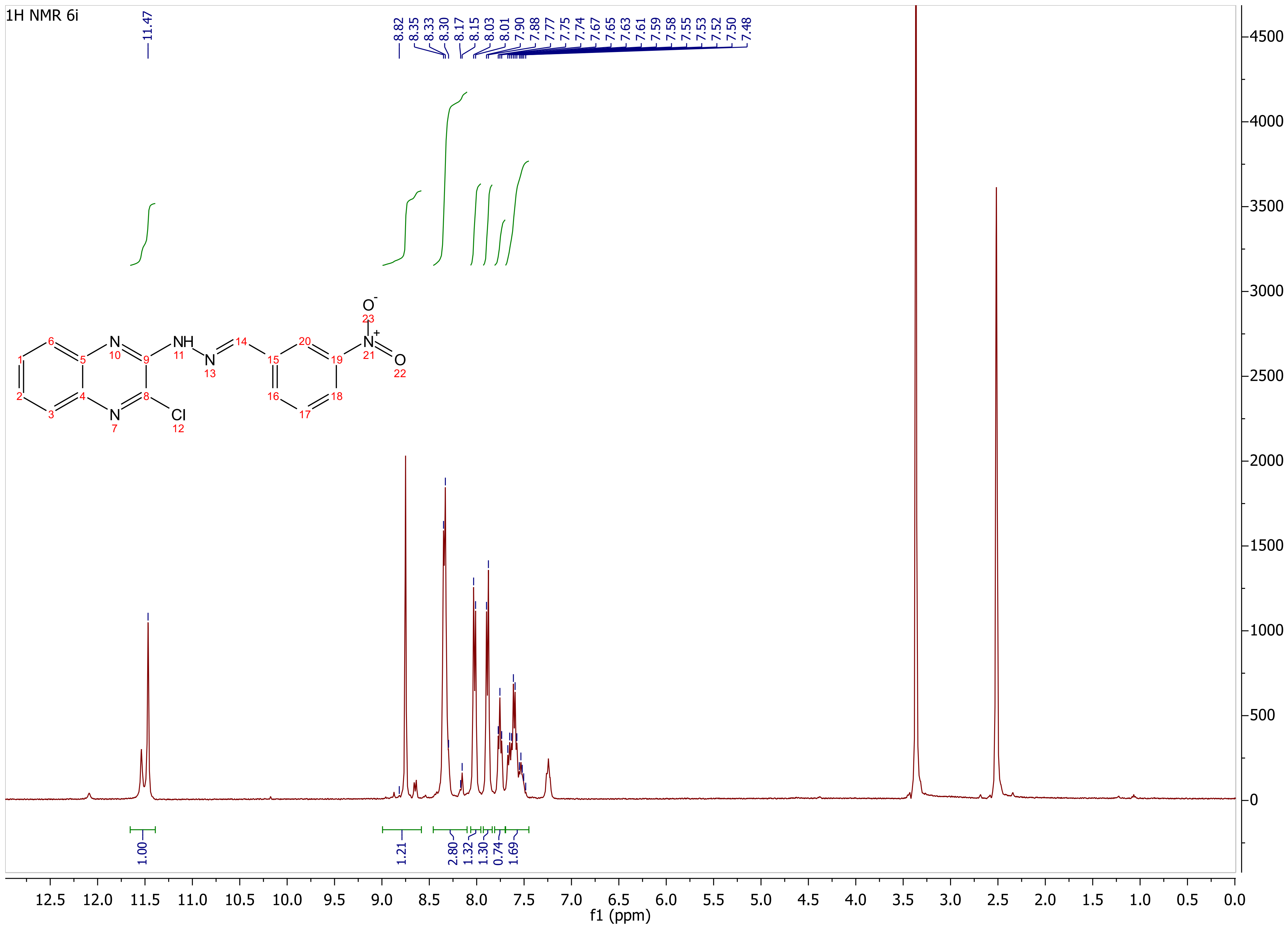
1H NMR 6i
11.47
8.82
8.35
8.33
8.30
8.17
8.15
8.03
8.01
7.90
7.88
7.77
7.75
7.74
7.67
7.65
7.63
7.61
7.59
7.58
7.55
7.53
7.52
7.50
7.48
1.00
1.21
2.80
1.32
1.30
0.74
1.69
f1 (ppm)

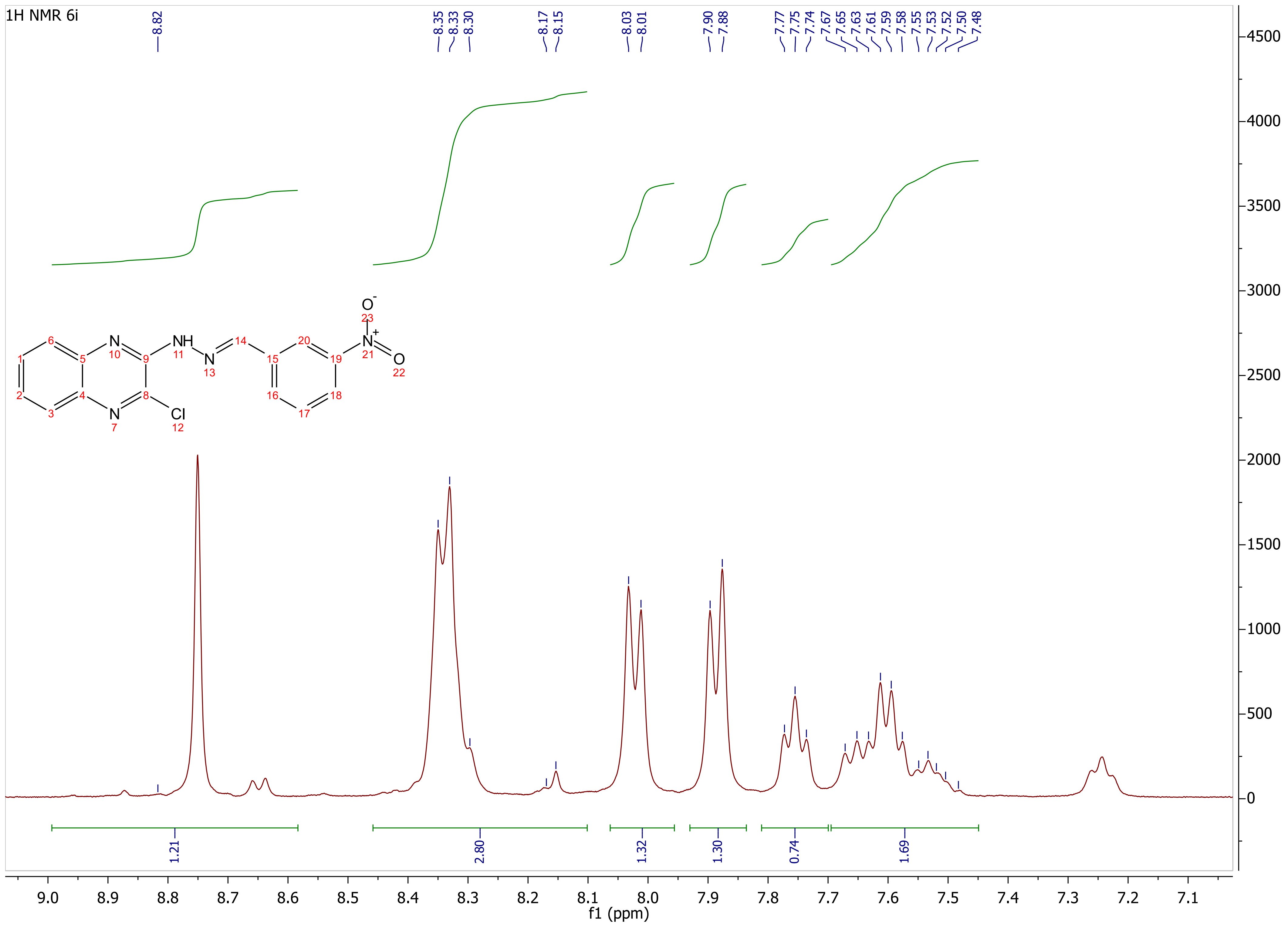
1H NMR 6i
8.82
8.35
8.33
8.30
8.17
8.15
8.03
8.01
7.90
7.88
7.77
7.75
7.74
7.67
7.65
7.63
7.61
7.59
7.58
7.55
7.53
7.52
7.50
7.48
1.21
2.80
1.32
1.30
0.74
1.69
f1 (ppm)

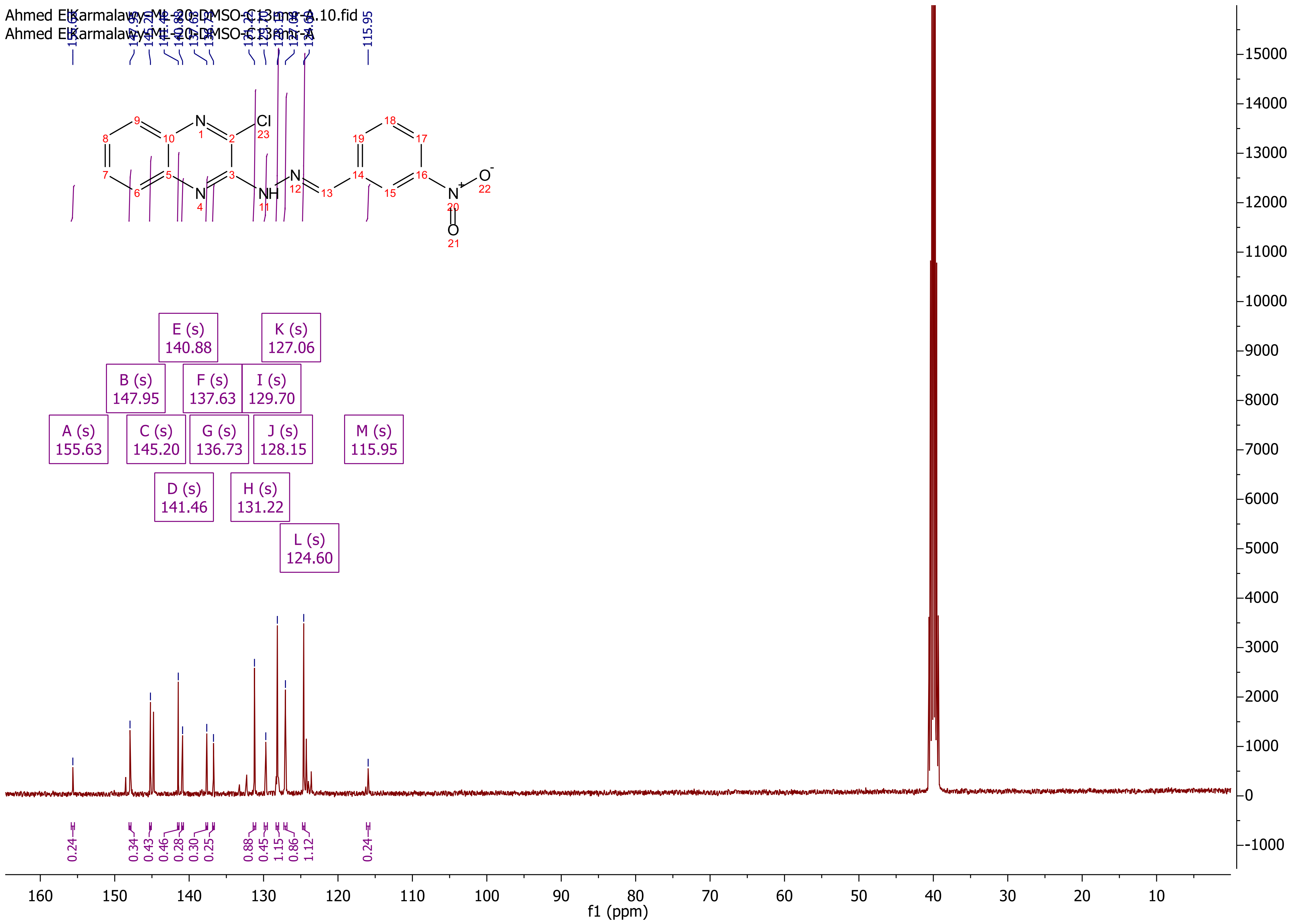
Ahmed ElKarmalawy-ML-20-DMSO-C13nmr-A.10.fid
Ahmed ElKarmalawy-ML-20-DMSO-C13nmr-A
115.95
A (s) 155.63
B (s) 147.95
C (s) 145.20
D (s) 141.46
E (s) 140.88
F (s) 137.63
G (s) 136.73
H (s) 131.22
I (s) 129.70
J (s) 128.15
K (s) 127.06
L (s) 124.60
M (s) 115.95
Cl
NH
N
O
0.24
0.34
0.43
0.46
0.28
0.30
0.25
0.88
0.45
1.15
0.86
1.12
0.24
f1 (ppm)

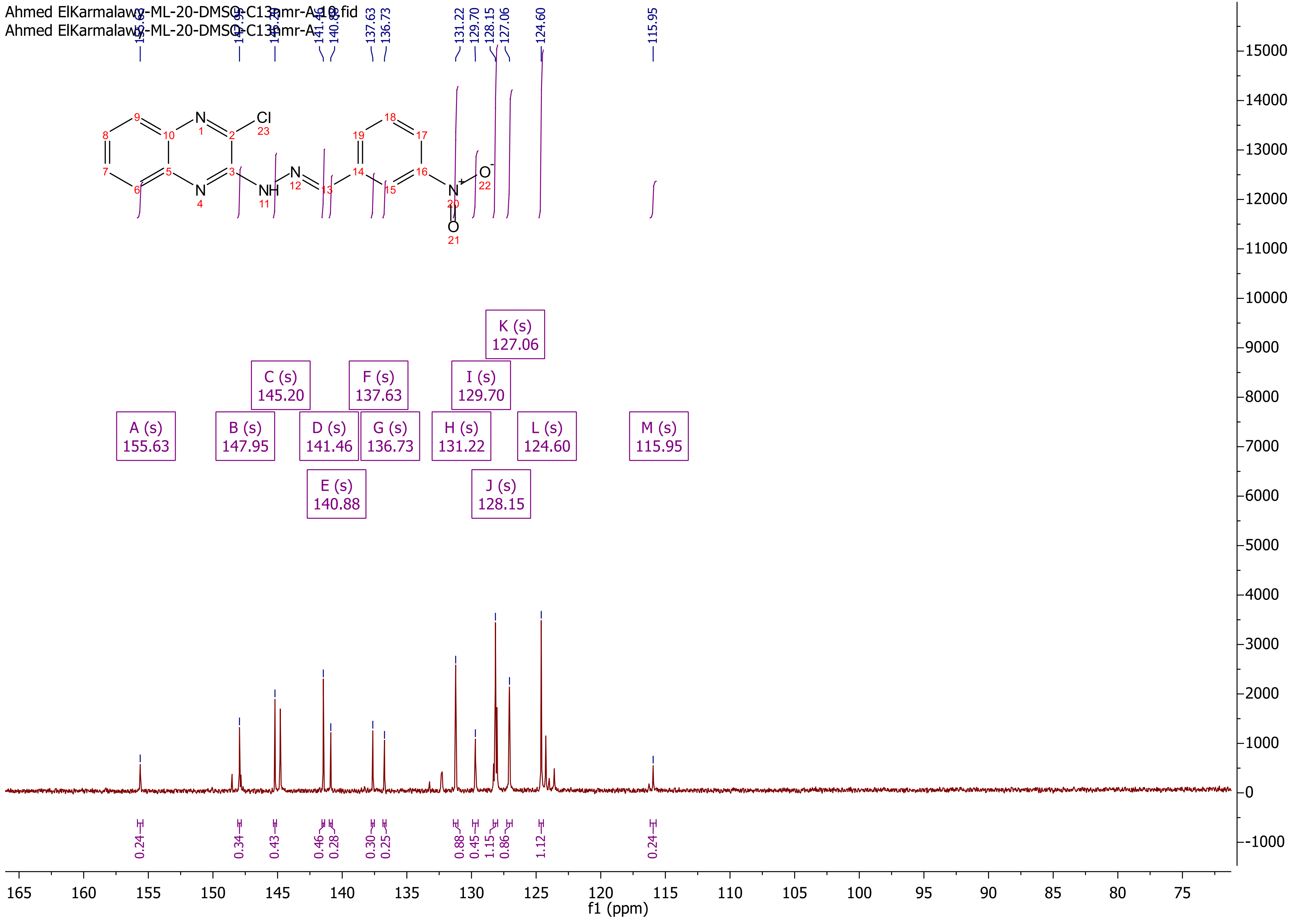

141.46
137.63
136.73
131.22
129.70
128.15
127.06
124.60
115.95
A (s) 155.63
B (s) 147.95
C (s) 145.20
D (s) 141.46
E (s) 140.88
F (s) 137.63
G (s) 136.73
H (s) 131.22
I (s) 129.70
J (s) 128.15
K (s) 127.06
L (s) 124.60
M (s) 115.95
0.24
0.34
0.43
0.46
0.28
0.30
0.25
0.88
0.45
1.15
0.86
1.12
0.24
f1 (ppm)

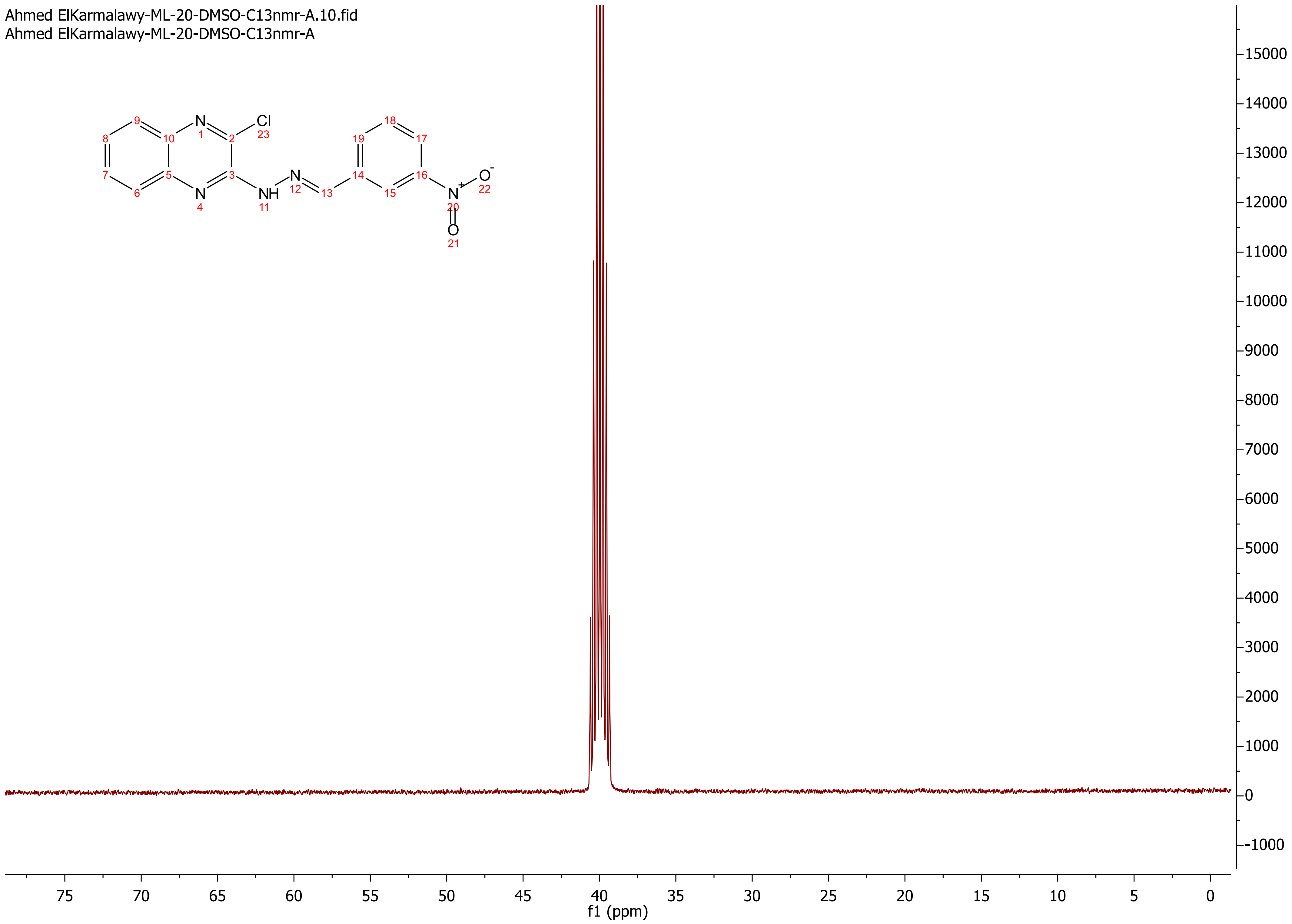
Ahmed ElKarmalawy-ML-20-DMSO-C13nmr-A.10.fid
Ahmed ElKarmalawy-ML-20-DMSO-C13nmr-A
N
Cl
NH
N
N
N
O
O
f1 (ppm)
75
70
65
60
55
50
45
40
35
30
25
20
15
10
5
0
15000
14000
13000
12000
11000
10000
9000
8000
7000
6000
5000
4000
3000
2000
1000
0
-1000

IR of compound 6j

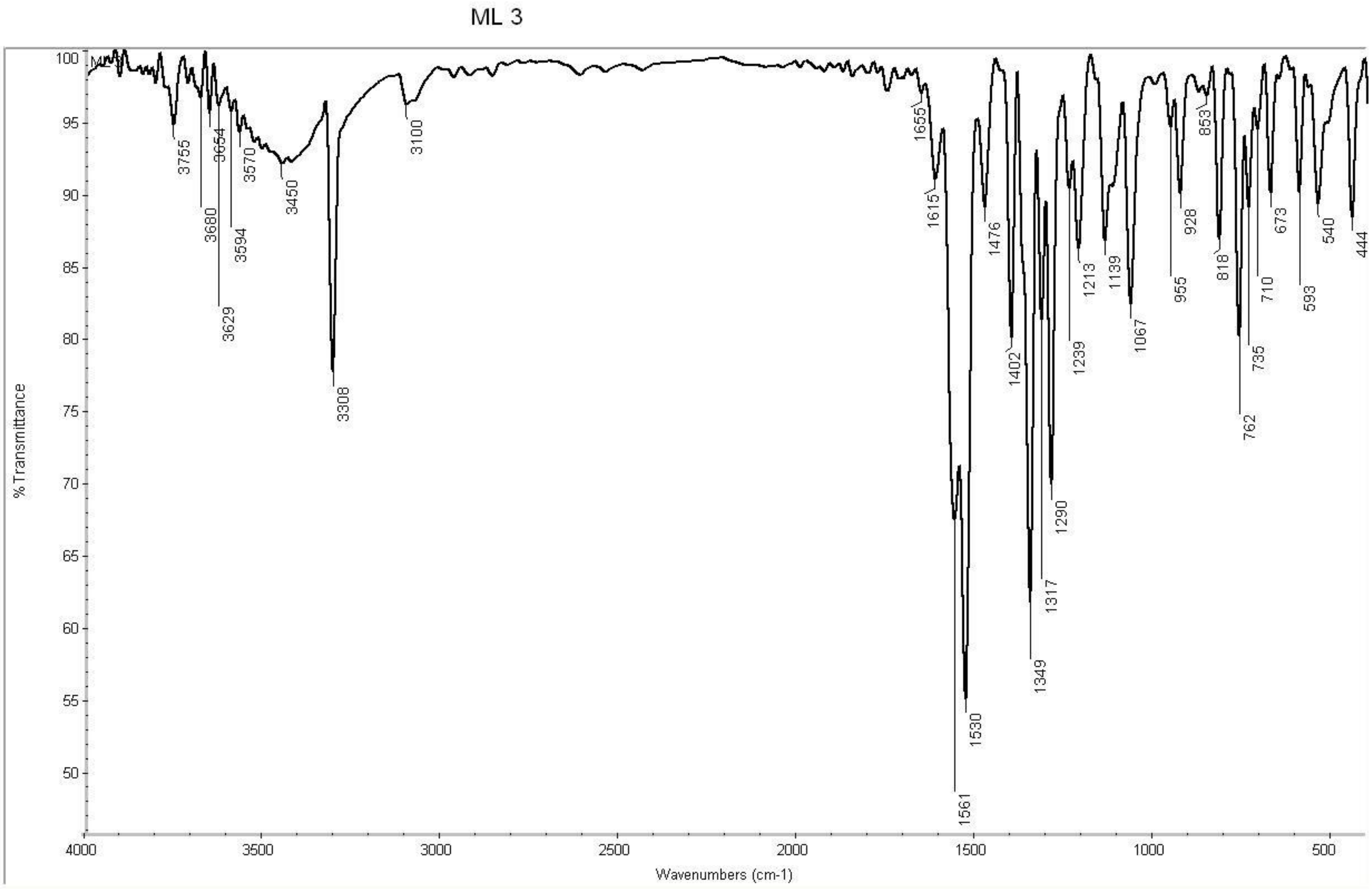

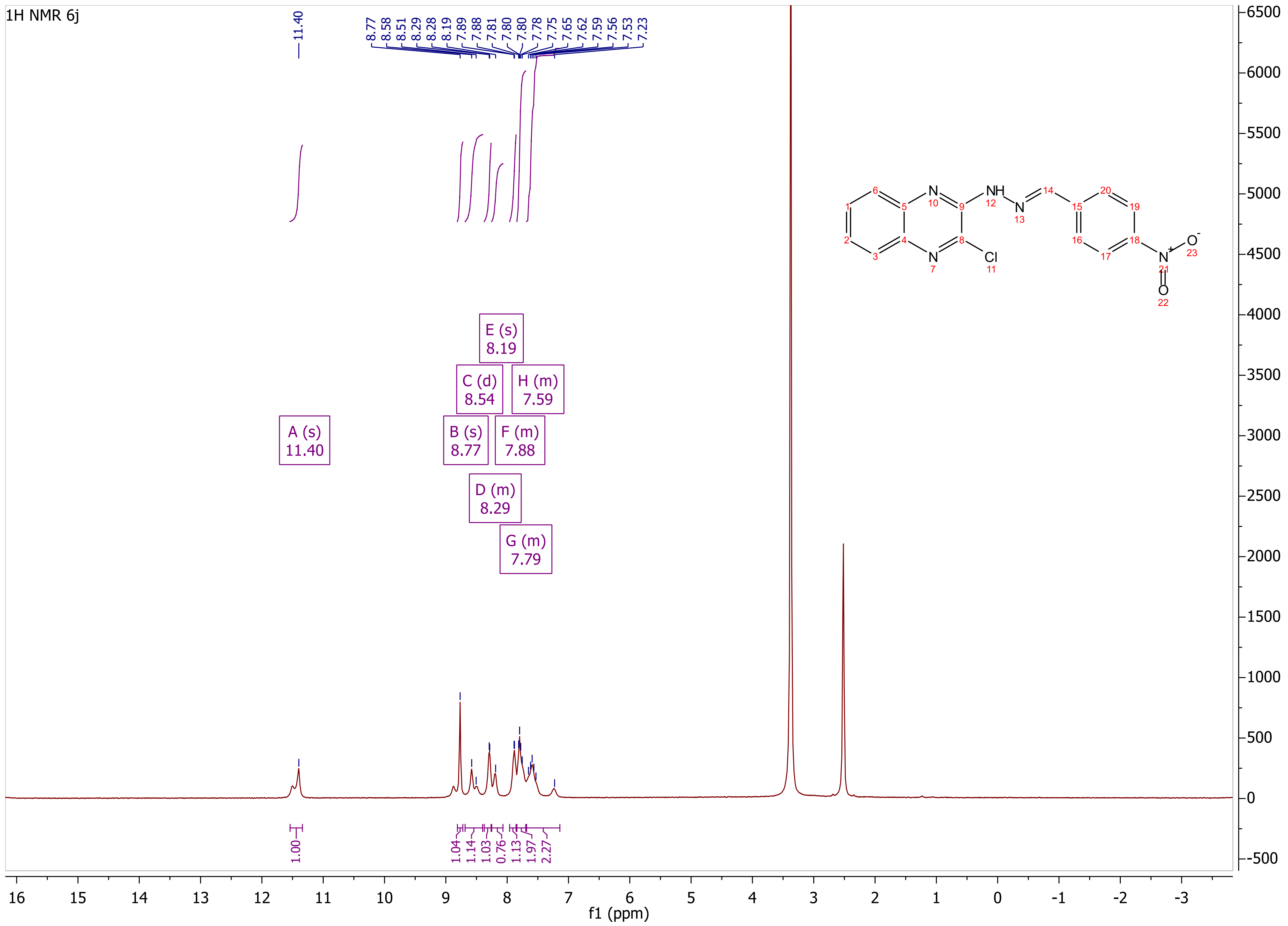
1H NMR 6j
11.40
8.77 8.58 8.51 8.29 8.28 8.19 7.89 7.88 7.81 7.80 7.80 7.78 7.75 7.65 7.62 7.59 7.56 7.53 7.23
E (s) 8.19
C (d) 8.54
H (m) 7.59
A (s) 11.40
B (s) 8.77
F (m) 7.88
D (m) 8.29
G (m) 7.79
1.00 1.04 1.14 1.03 0.76 1.13 1.97 2.27
f1 (ppm)
NH
N
Cl
O
O-
N+

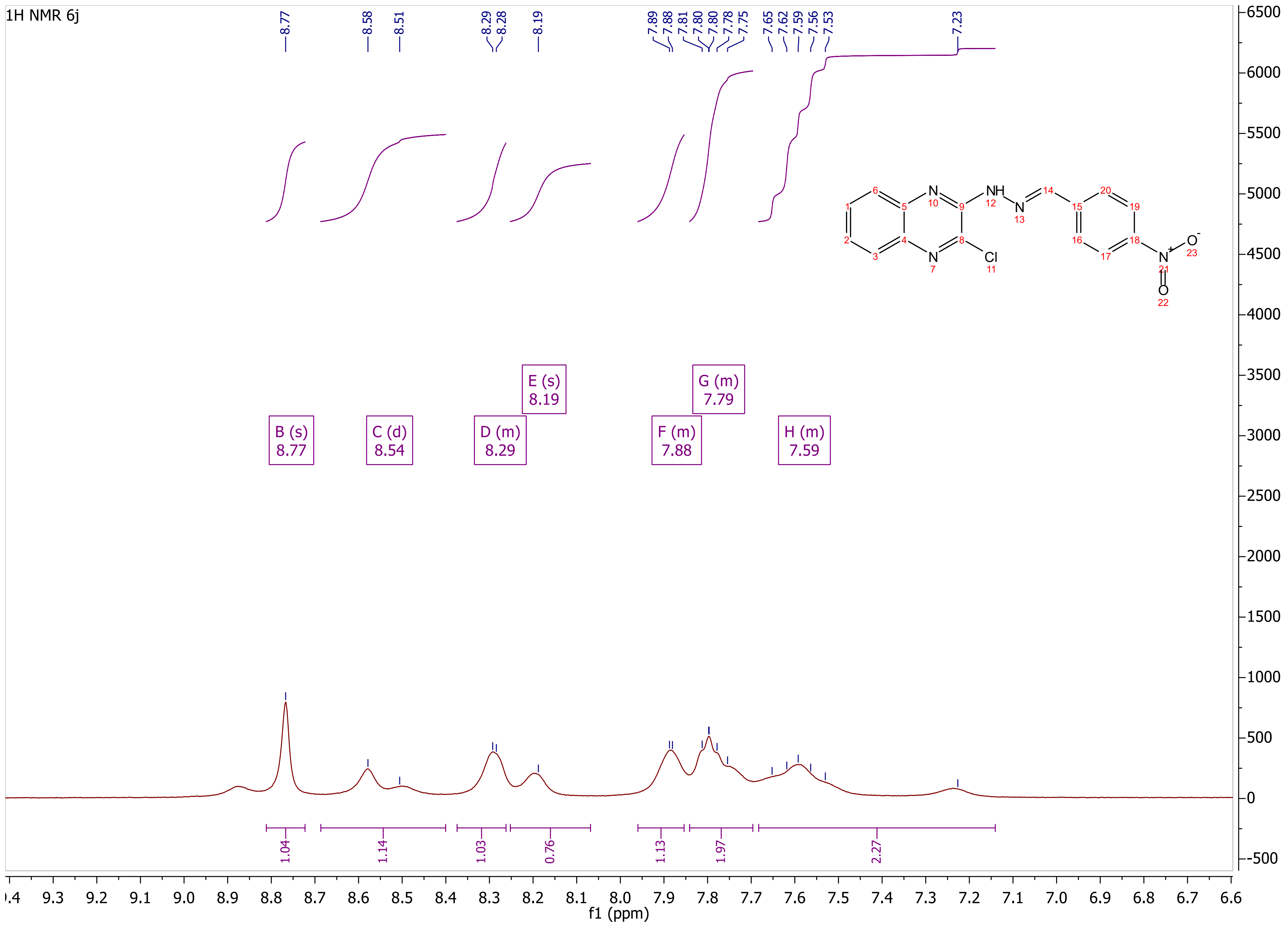
1H NMR 6j
8.77
8.58
8.51
8.29
8.28
8.19
7.89
7.88
7.81
7.80
7.80
7.78
7.75
7.65
7.62
7.59
7.56
7.53
7.23
B (s) 8.77
C (d) 8.54
D (m) 8.29
E (s) 8.19
F (m) 7.88
G (m) 7.79
H (m) 7.59
1.04
1.14
1.03
0.76
1.13
1.97
2.27
NH
N
N
N
Cl
O
O
f1 (ppm)

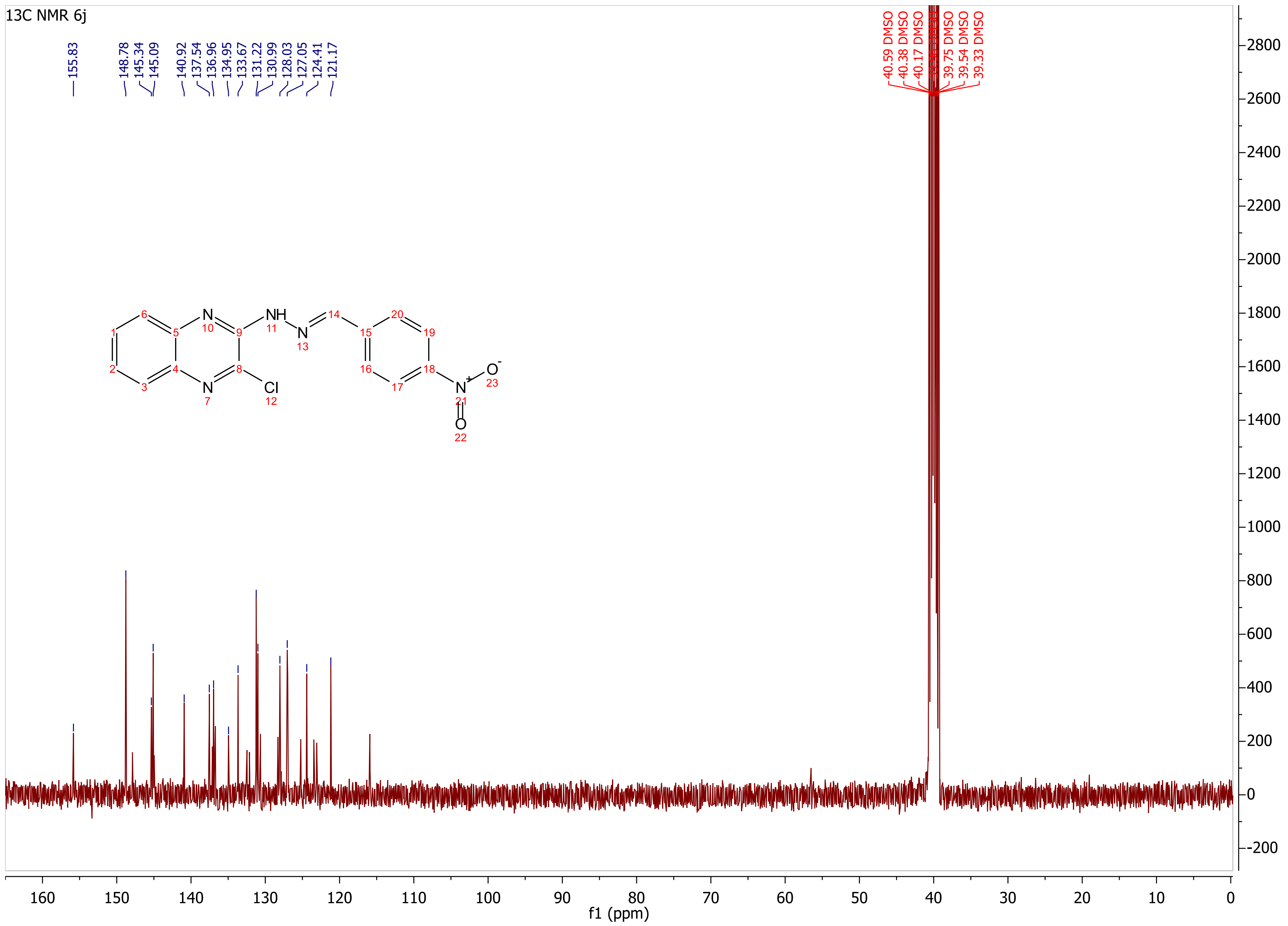

13C NMR 6j
155.83
148.78
145.34
145.09
140.92
137.54
136.96
134.95
133.67
131.22
130.99
128.03
127.05
124.41
121.17
40.59 DMSO
40.38 DMSO
40.17 DMSO
39.75 DMSO
39.54 DMSO
39.33 DMSO
NH
N
Cl
O
f1 (ppm)

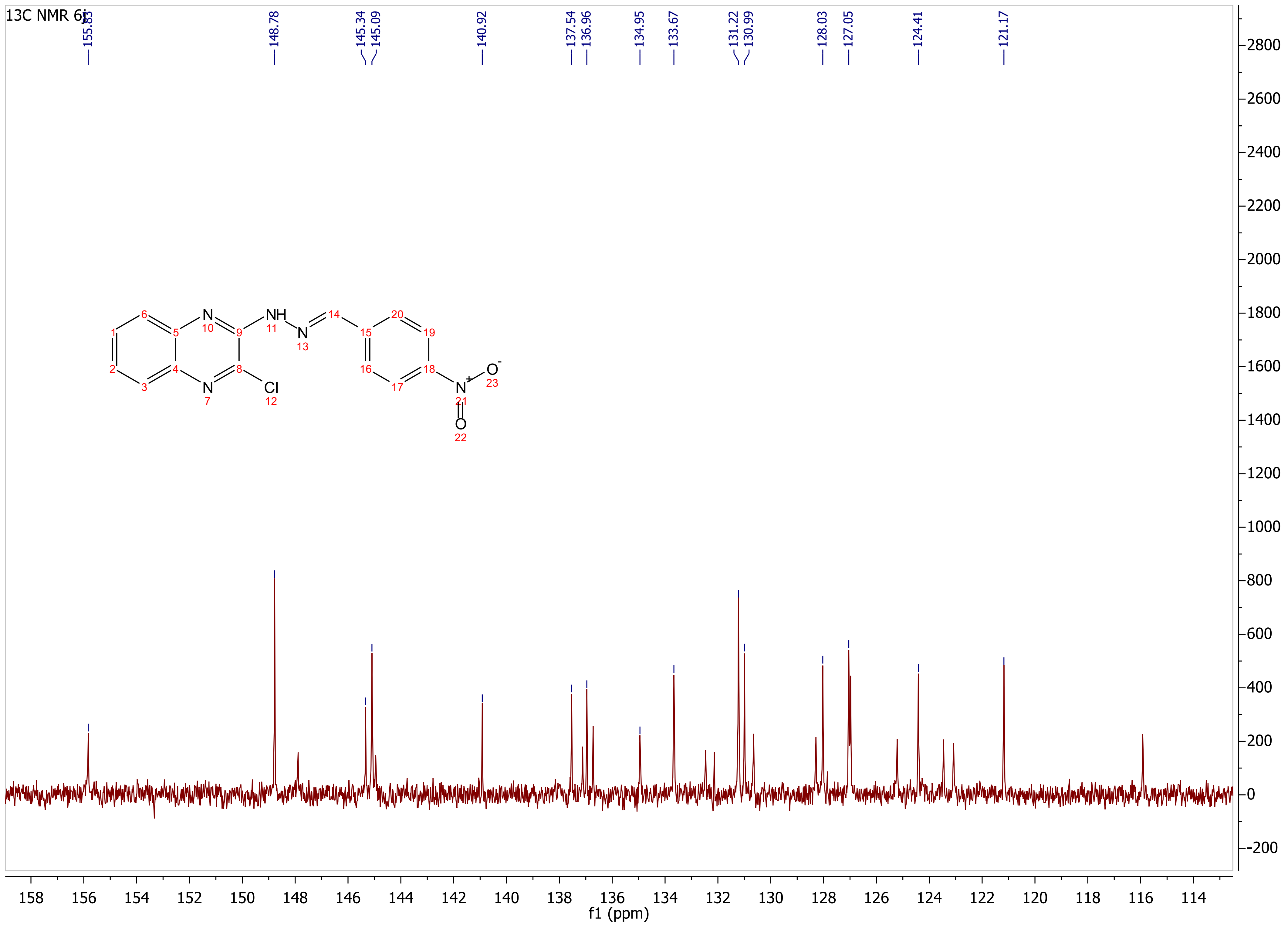

13C NMR 6j
155.83
148.78
145.34
145.09
140.92
137.54
136.96
134.95
133.67
131.22
130.99
128.03
127.05
124.41
121.17
NH
N
Cl
O
f1 (ppm)

IR of compound 6k

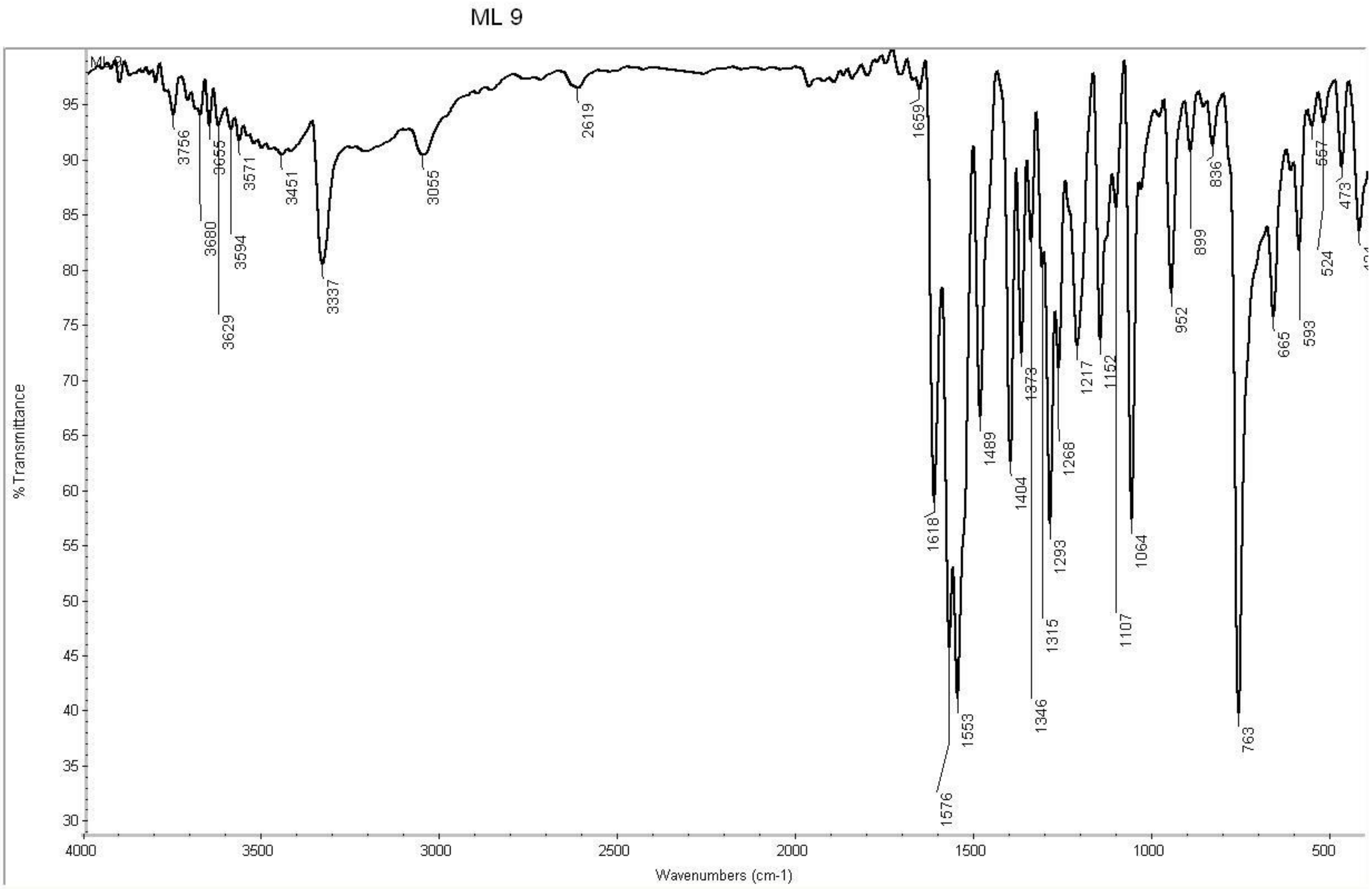

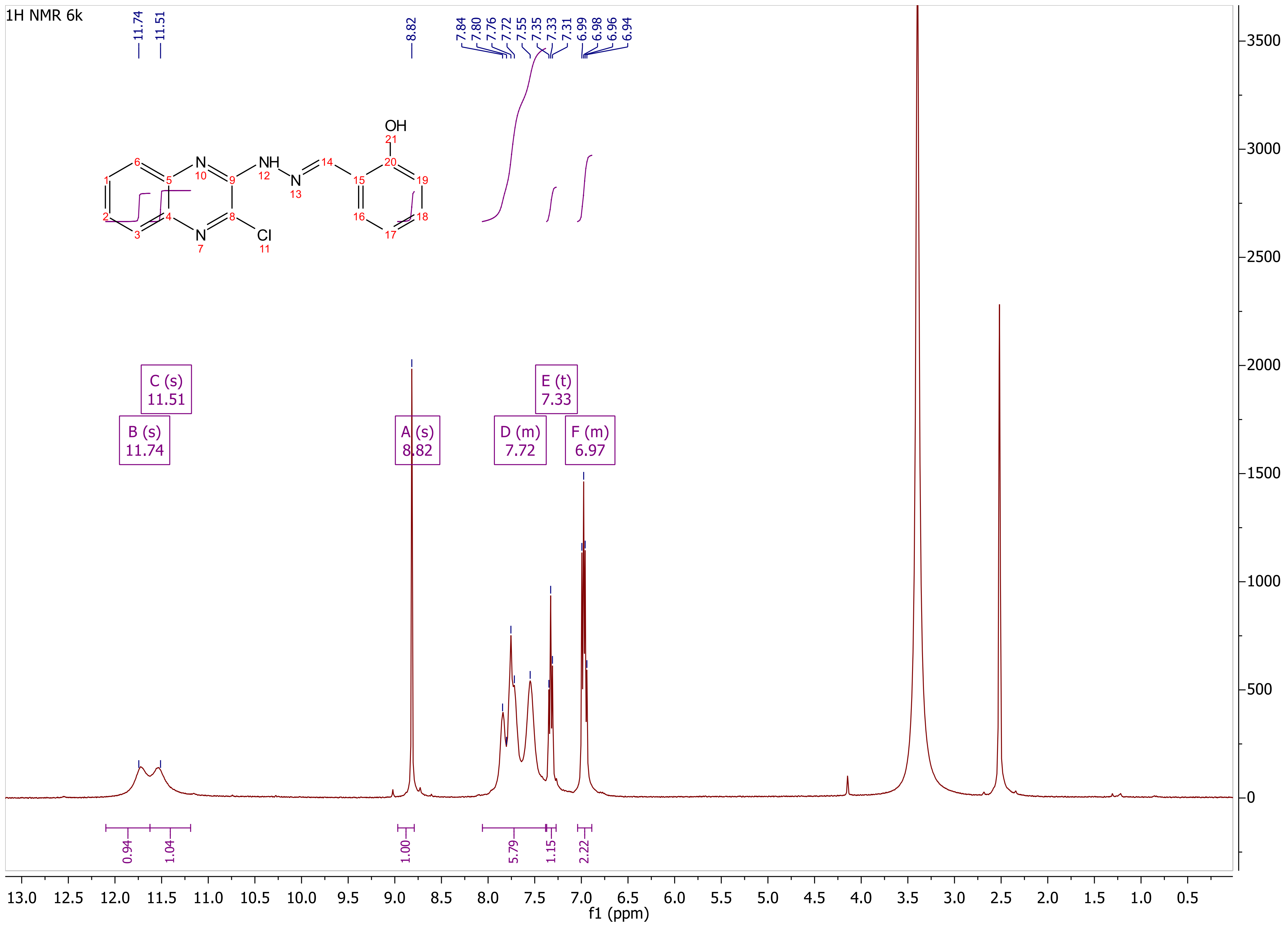
1H NMR 6k
11.74
11.51
8.82
7.84
7.80
7.76
7.72
7.55
7.35
7.33
7.31
6.99
6.98
6.96
6.94
OH
NH
N
Cl
C (s)
11.51
B (s)
11.74
A (s)
8.82
D (m)
7.72
E (t)
7.33
F (m)
6.97
0.94
1.04
1.00
5.79
1.15
2.22
f1 (ppm)

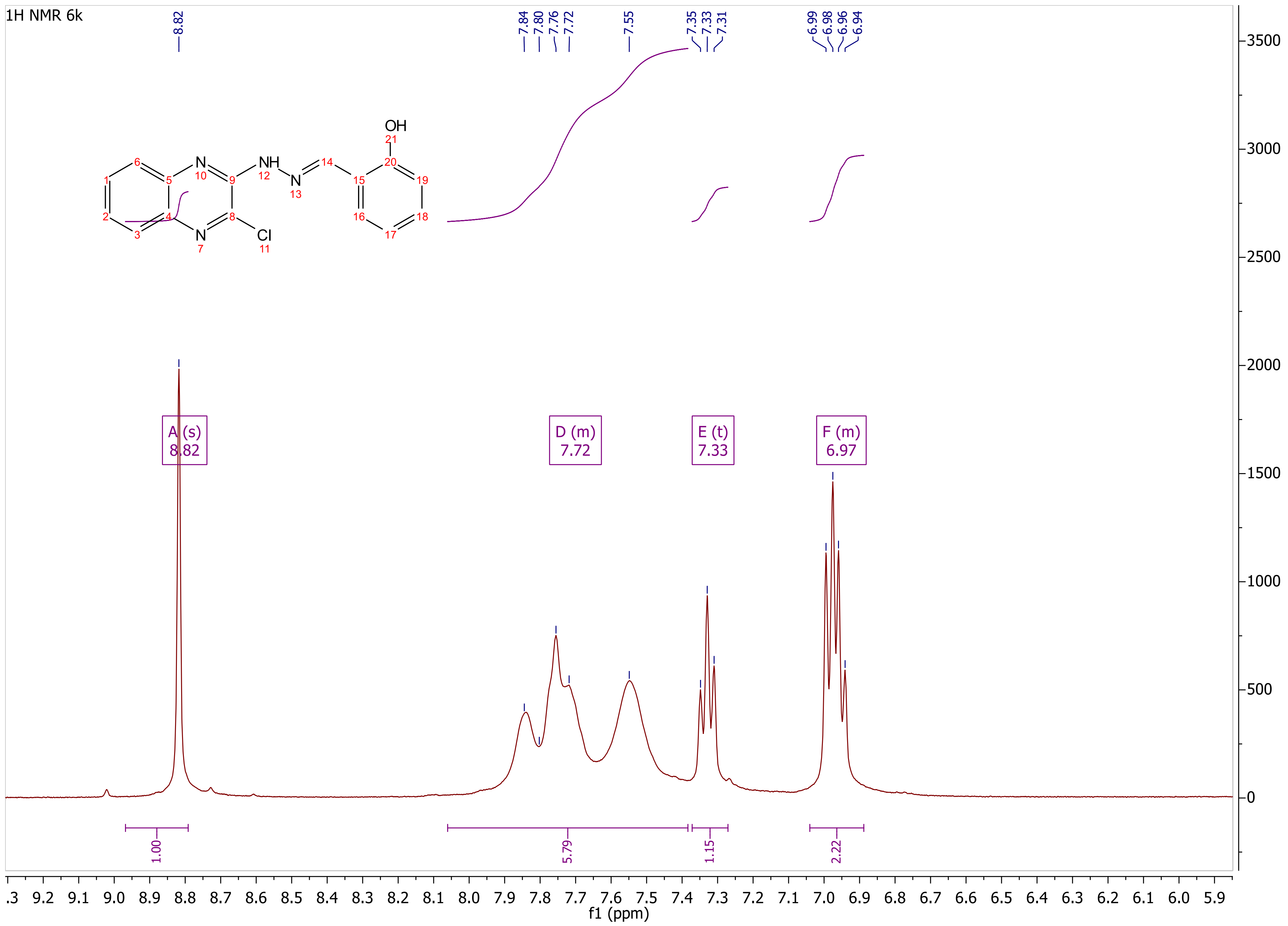
1H NMR 6k
8.82
7.84
7.80
7.76
7.72
7.55
7.35
7.33
7.31
6.99
6.98
6.96
6.94
OH
N
NH
N
N
Cl
A (s) 8.82
D (m) 7.72
E (t) 7.33
F (m) 6.97
1.00
5.79
1.15
2.22
f1 (ppm)

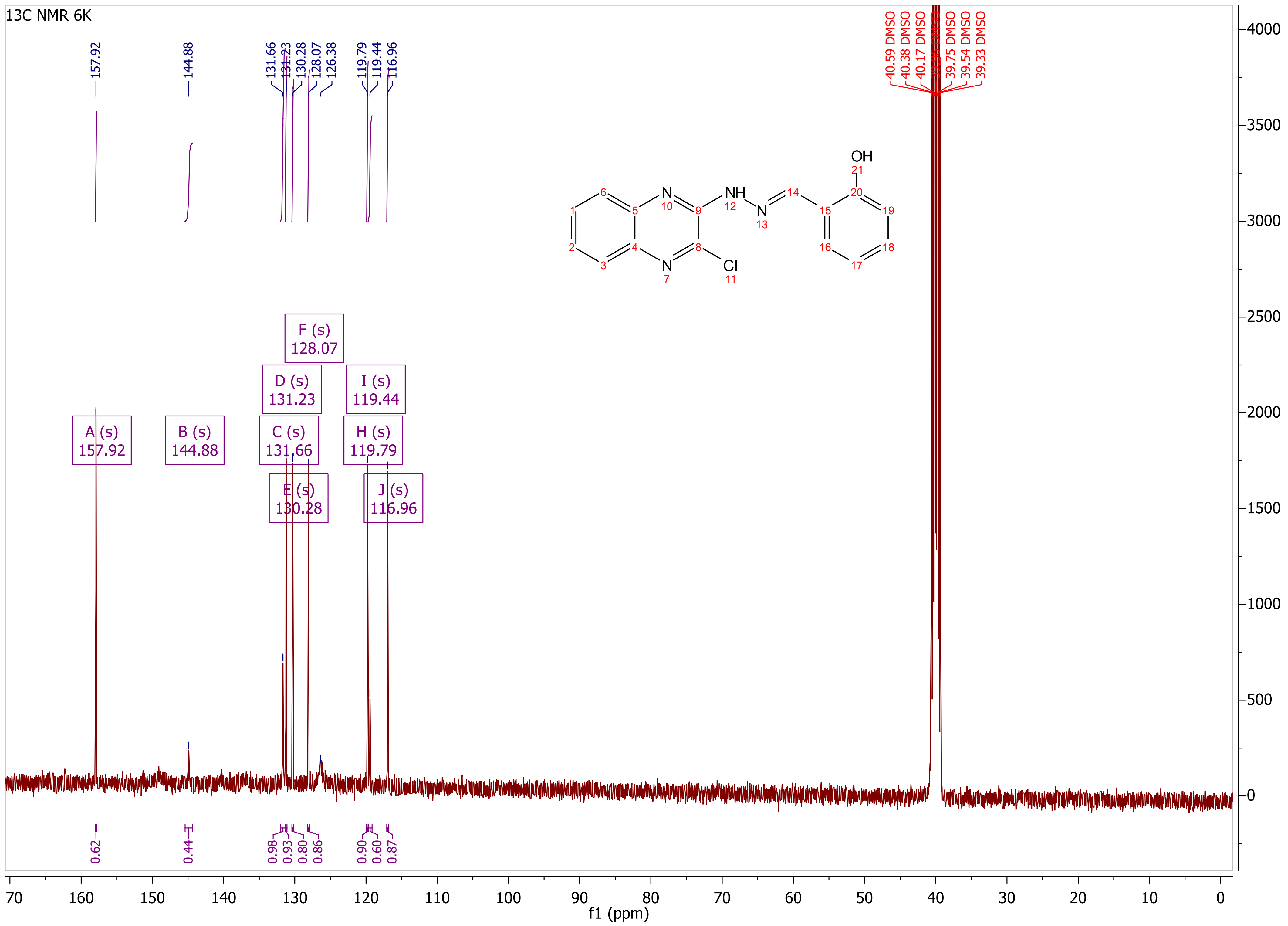
13C NMR 6K
157.92
144.88
131.66
131.23
130.28
128.07
126.38
119.79
119.44
116.96
40.59 DMSO
40.38 DMSO
40.17 DMSO
39.75 DMSO
39.54 DMSO
39.33 DMSO
A (s) 157.92
B (s) 144.88
C (s) 131.66
D (s) 131.23
E (s) 130.28
F (s) 128.07
H (s) 119.79
I (s) 119.44
J (s) 116.96
0.62
0.44
0.98
0.93
0.80
0.86
0.90
0.60
0.87
OH
NH
N
Cl
f1 (ppm)

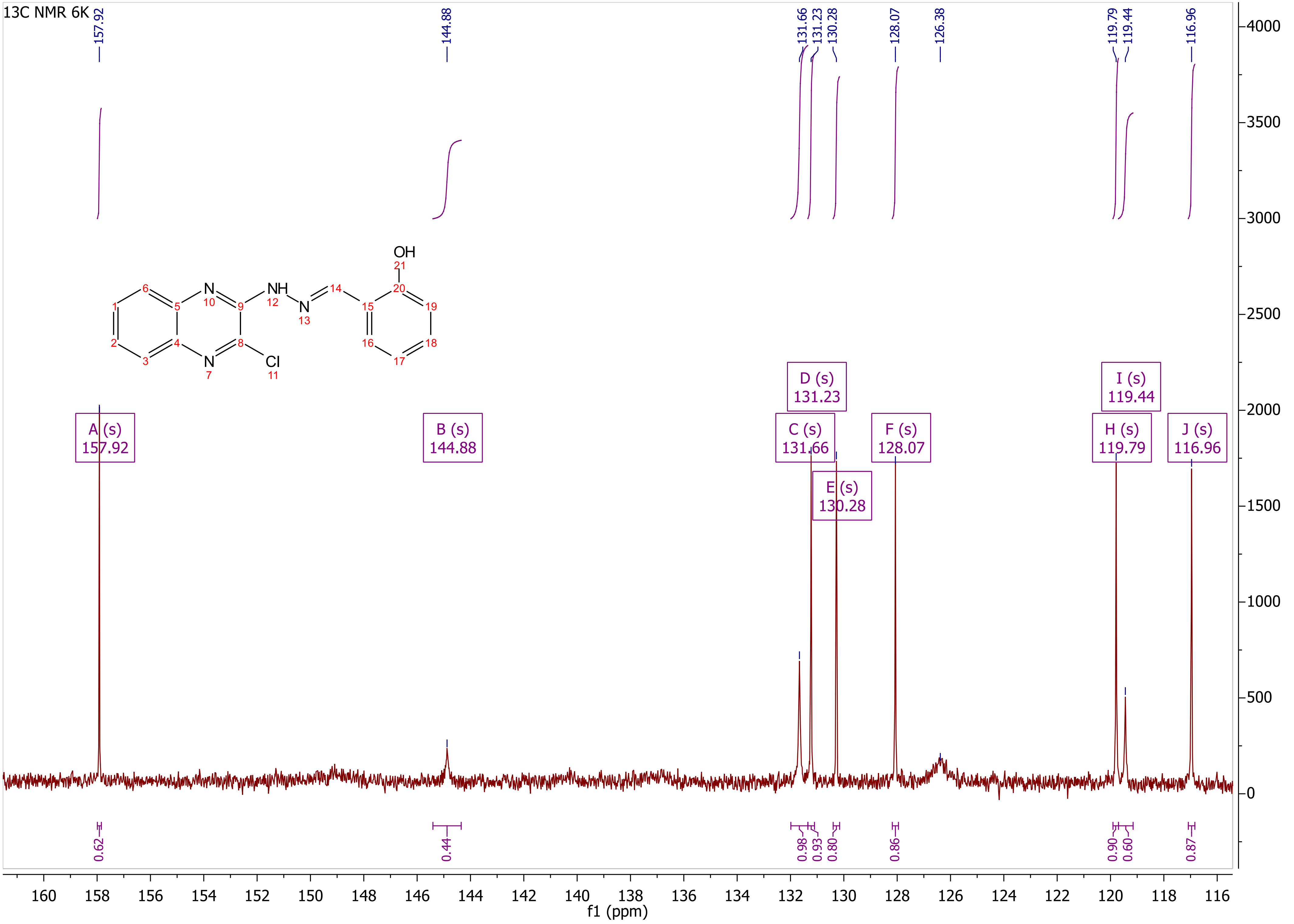
13C NMR 6K
157.92
144.88
131.66
131.23
130.28
128.07
126.38
119.79
119.44
116.96
OH
NH
N
N
N
Cl
A (s) 157.92
B (s) 144.88
C (s) 131.66
D (s) 131.23
E (s) 130.28
F (s) 128.07
H (s) 119.79
I (s) 119.44
J (s) 116.96
0.62
0.44
0.98
0.93
0.80
0.86
0.90
0.60
0.87
160 158 156 154 152 150 148 146 144 142 140 138 136 134 132 130 128 126 124 122 120 118 116
f1 (ppm)
4000
3500
3000
2500
2000
1500
1000
500
0

IR of compound 6l

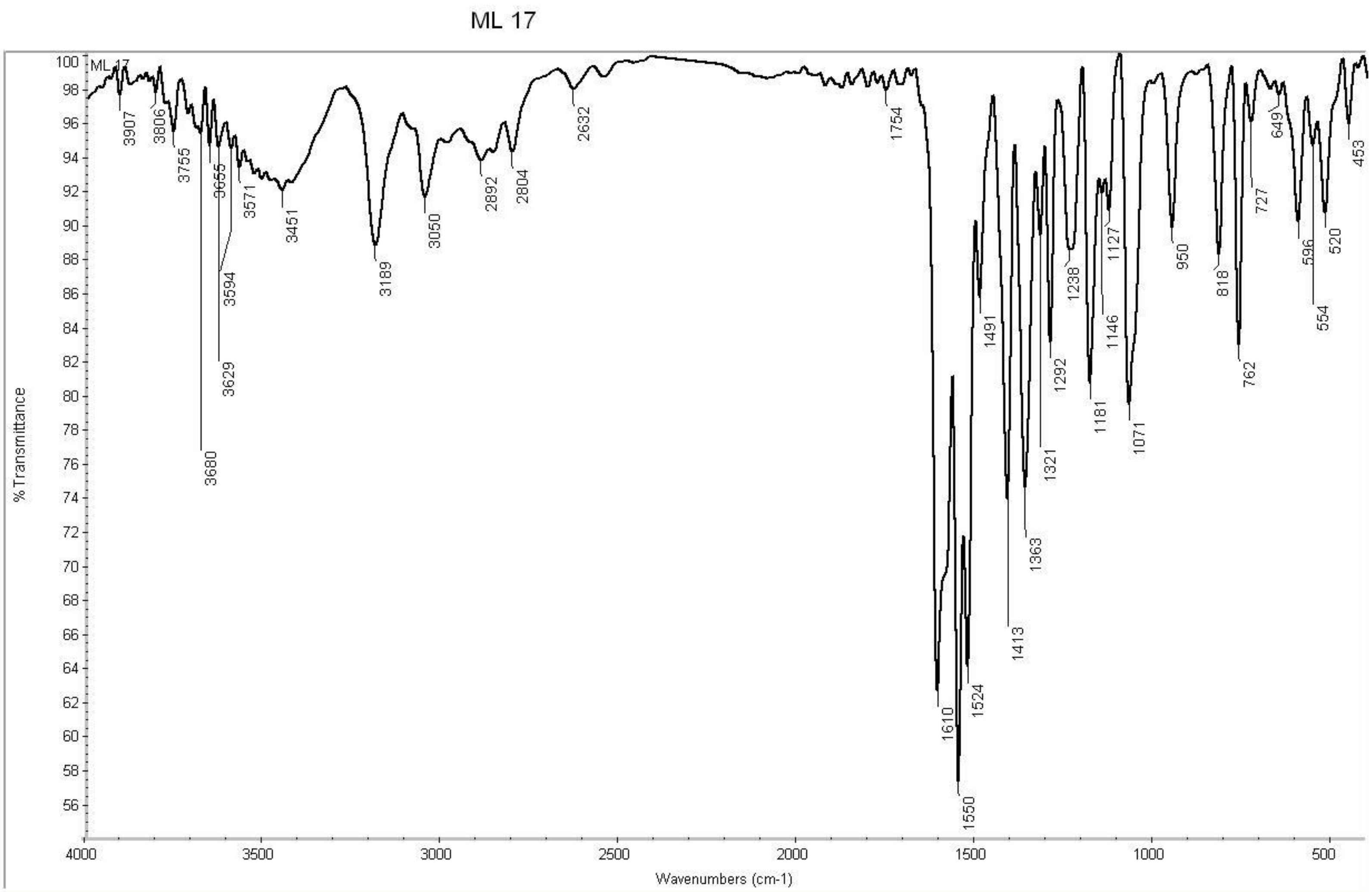

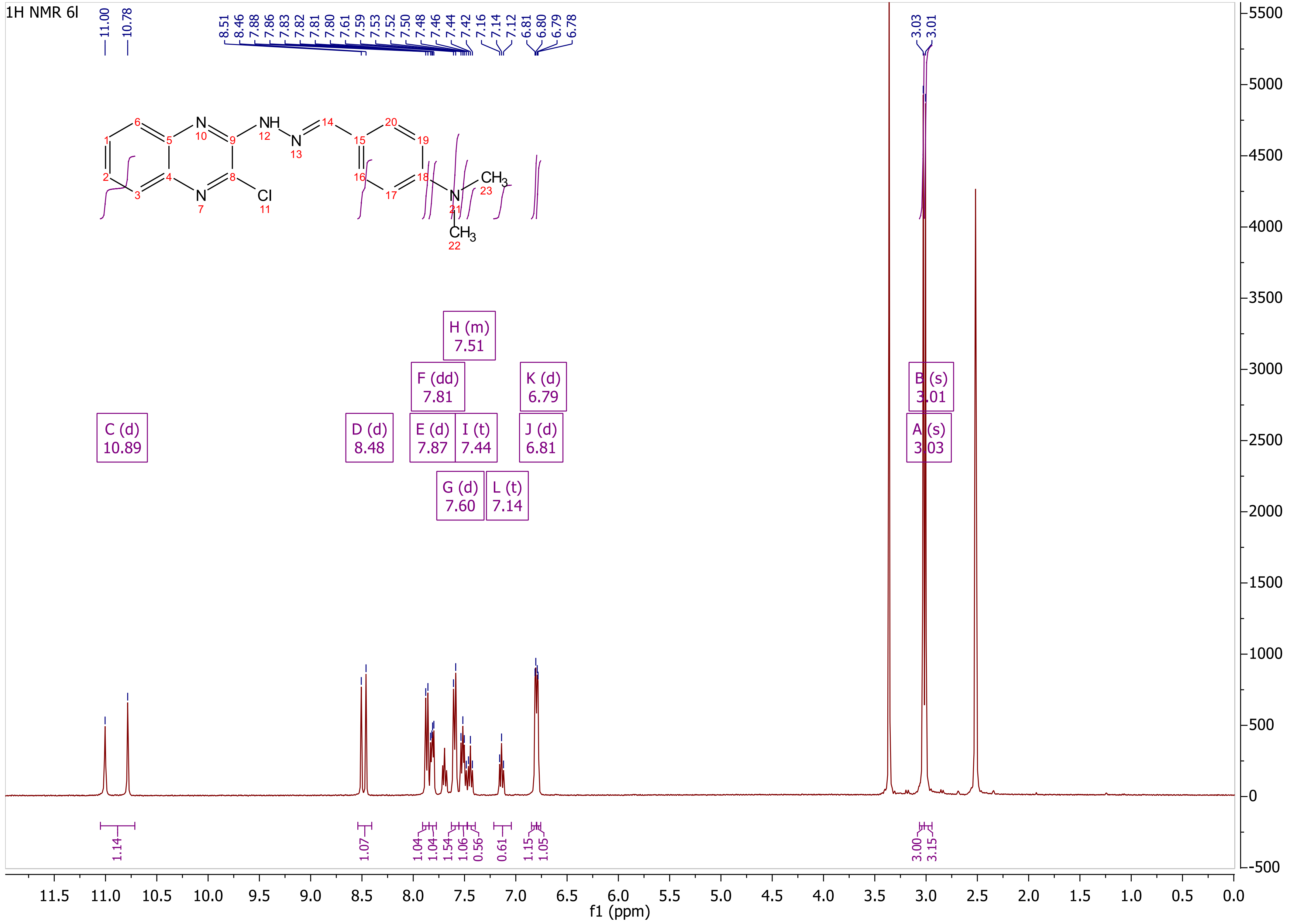
1H NMR 6l
11.00
10.78
8.51
8.46
7.88
7.86
7.83
7.82
7.81
7.80
7.61
7.59
7.53
7.52
7.50
7.48
7.46
7.44
7.42
7.16
7.14
7.12
6.81
6.80
6.79
6.78
3.03
3.01
NH
N
Cl
CH3
H (m) 7.51
F (dd) 7.81
K (d) 6.79
B (s) 3.01
C (d) 10.89
D (d) 8.48
E (d) 7.87
I (t) 7.44
J (d) 6.81
A (s) 3.03
G (d) 7.60
L (t) 7.14
1.14
1.07
1.04
1.04
1.54
1.06
0.56
0.61
1.15
1.05
3.00
3.15
f1 (ppm)

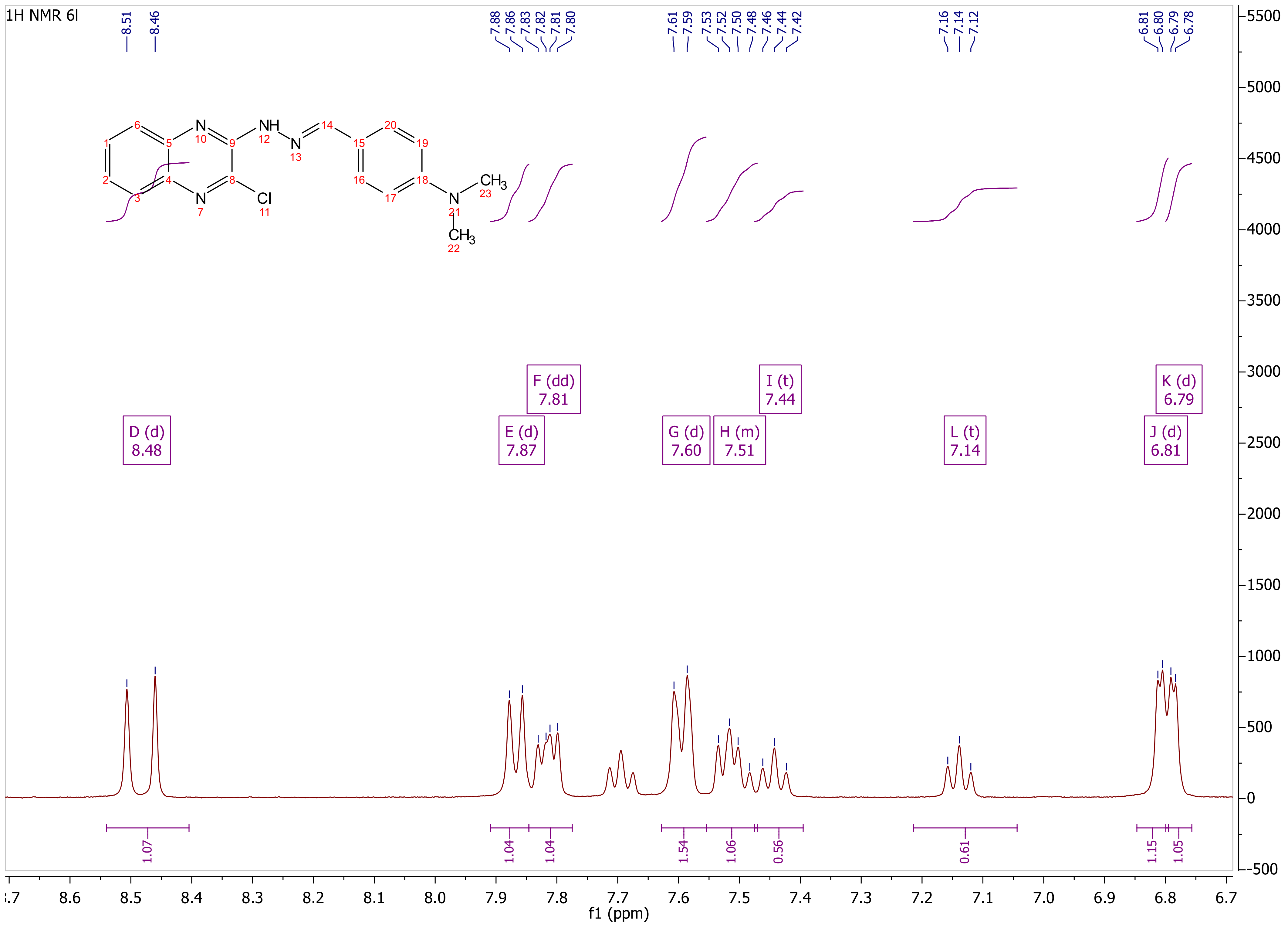
1H NMR 6l
8.51
8.46
7.88
7.86
7.83
7.82
7.81
7.80
7.61
7.59
7.53
7.52
7.50
7.48
7.46
7.44
7.42
7.16
7.14
7.12
6.81
6.80
6.79
6.78
N
NH
N
N
Cl
CH3
N
CH3
D (d)
8.48
E (d)
7.87
F (dd)
7.81
G (d)
7.60
H (m)
7.51
I (t)
7.44
L (t)
7.14
J (d)
6.81
K (d)
6.79
1.07
1.04
1.04
1.54
1.06
0.56
0.61
1.15
1.05
f1 (ppm)

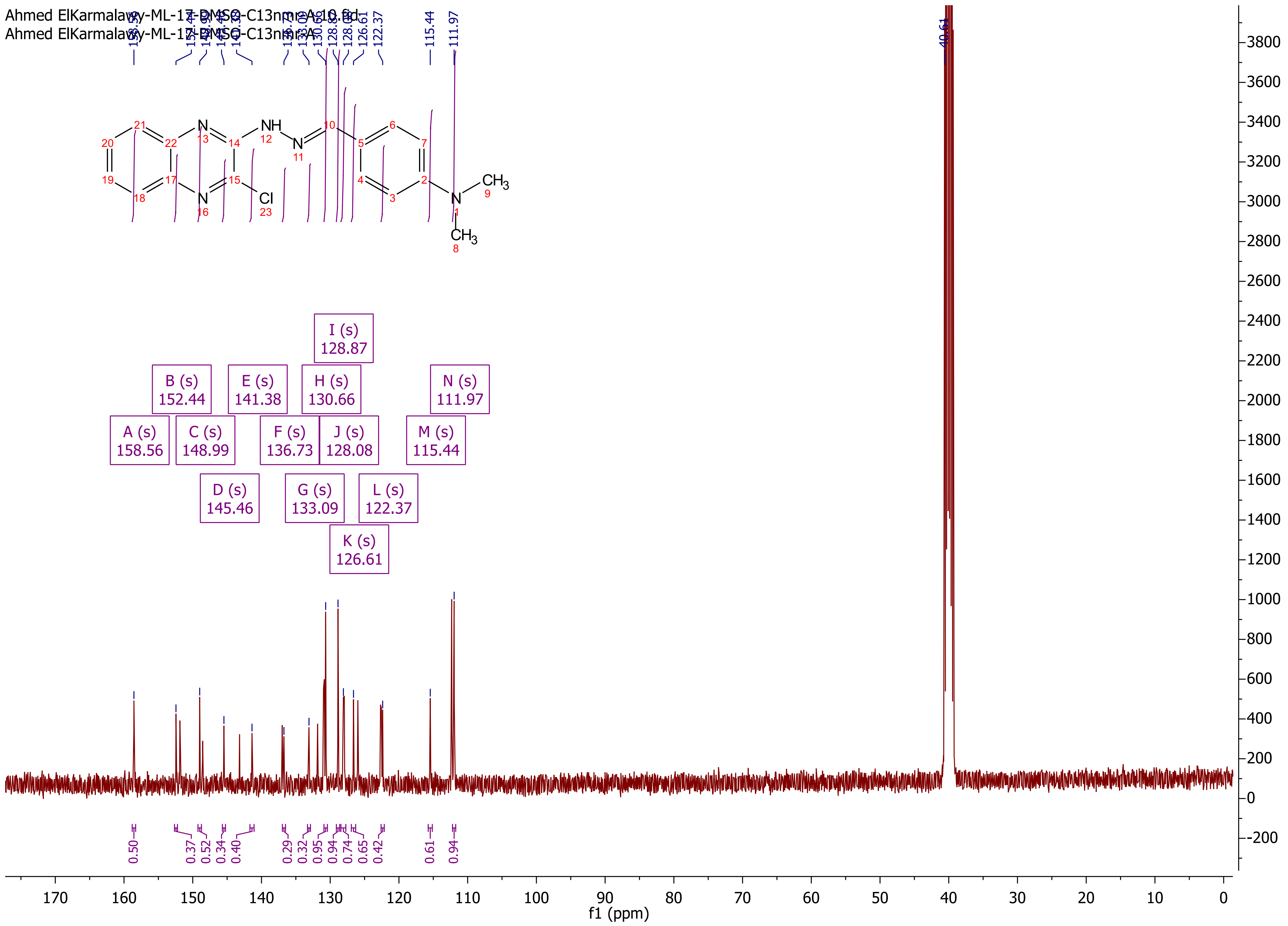
Ahmed ElKarmalawy-ML-17-DMSO-C13nmr-A.10.fid
Ahmed ElKarmalawy-ML-17-DMSO-C13nmr-A
158.56
152.44
148.99
145.46
141.38
136.73
133.09
130.66
128.87
128.08
126.61
122.37
115.44
111.97
40.61
NH
N
Cl
CH_3
A (s) 158.56
B (s) 152.44
C (s) 148.99
D (s) 145.46
E (s) 141.38
F (s) 136.73
G (s) 133.09
H (s) 130.66
I (s) 128.87
J (s) 128.08
K (s) 126.61
L (s) 122.37
M (s) 115.44
N (s) 111.97
0.50
0.37
0.52
0.34
0.40
0.29
0.32
0.95
0.94
0.74
0.65
0.42
0.61
0.94
f1 (ppm)

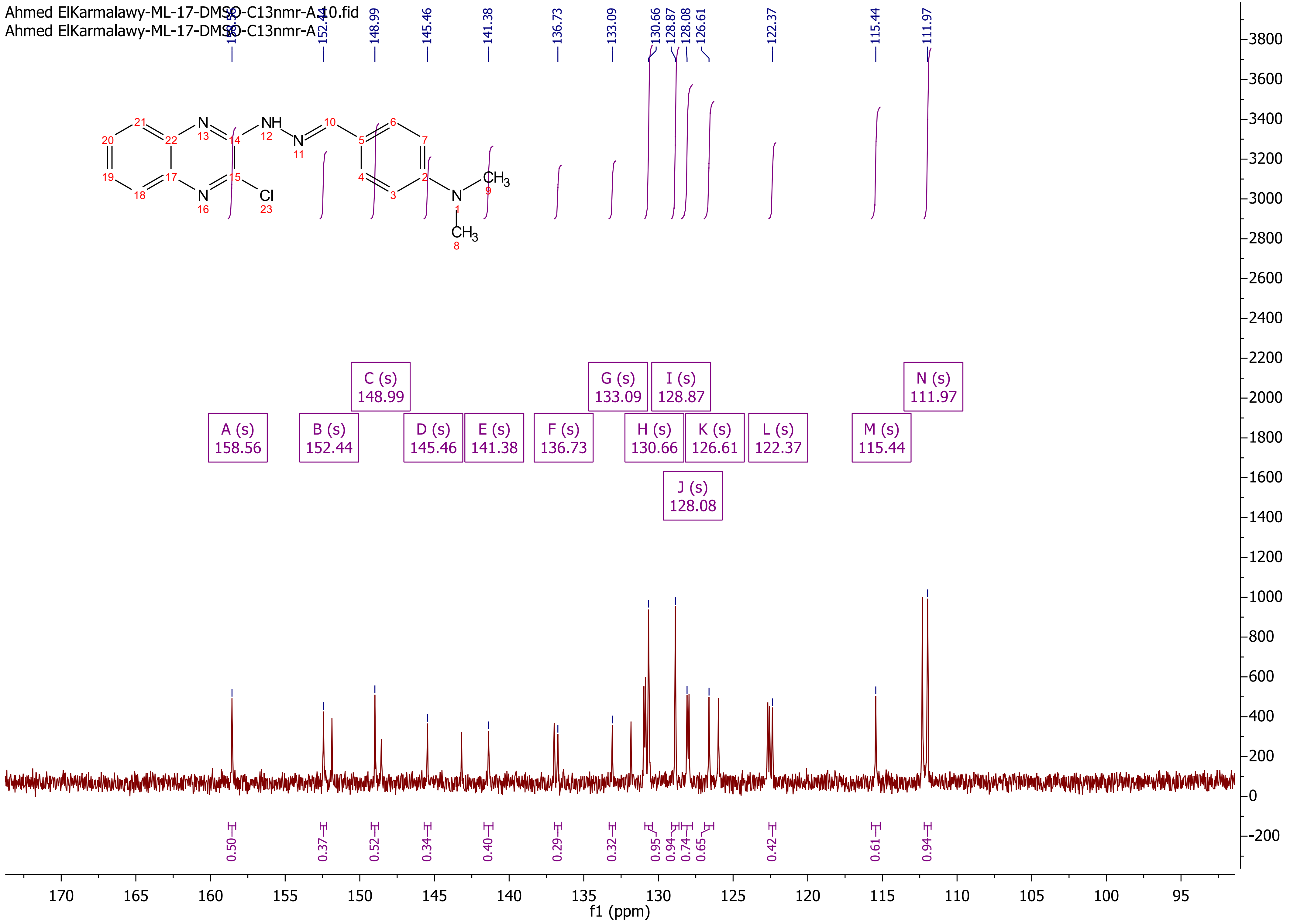
Ahmed ElKarmalawy-ML-17-DMSO-C13nmr-A.10.fid
Ahmed ElKarmalawy-ML-17-DMSO-C13nmr-A
158.56
152.44
148.99
145.46
141.38
136.73
133.09
130.66
128.87
128.08
126.61
122.37
115.44
111.97
A (s) 158.56
B (s) 152.44
C (s) 148.99
D (s) 145.46
E (s) 141.38
F (s) 136.73
G (s) 133.09
H (s) 130.66
I (s) 128.87
J (s) 128.08
K (s) 126.61
L (s) 122.37
M (s) 115.44
N (s) 111.97
0.50
0.37
0.52
0.34
0.40
0.29
0.32
0.95
0.94
0.74
0.65
0.42
0.61
0.94
f1 (ppm)

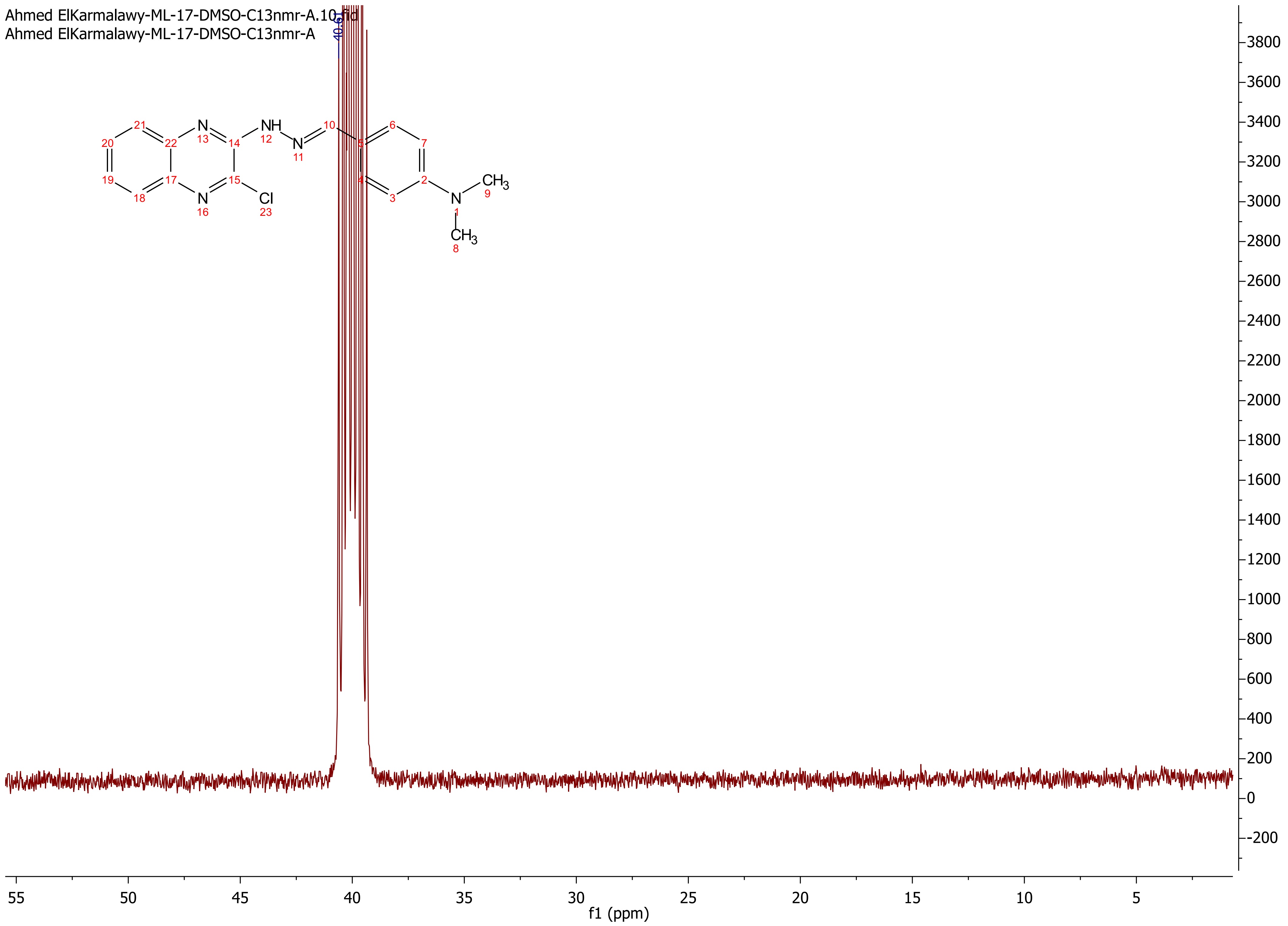
Ahmed ElKarmalawy-ML-17-DMSO-C13nmr-A.10.fid
Ahmed ElKarmalawy-ML-17-DMSO-C13nmr-A
40.61
f1 (ppm)

IR of compound 7a

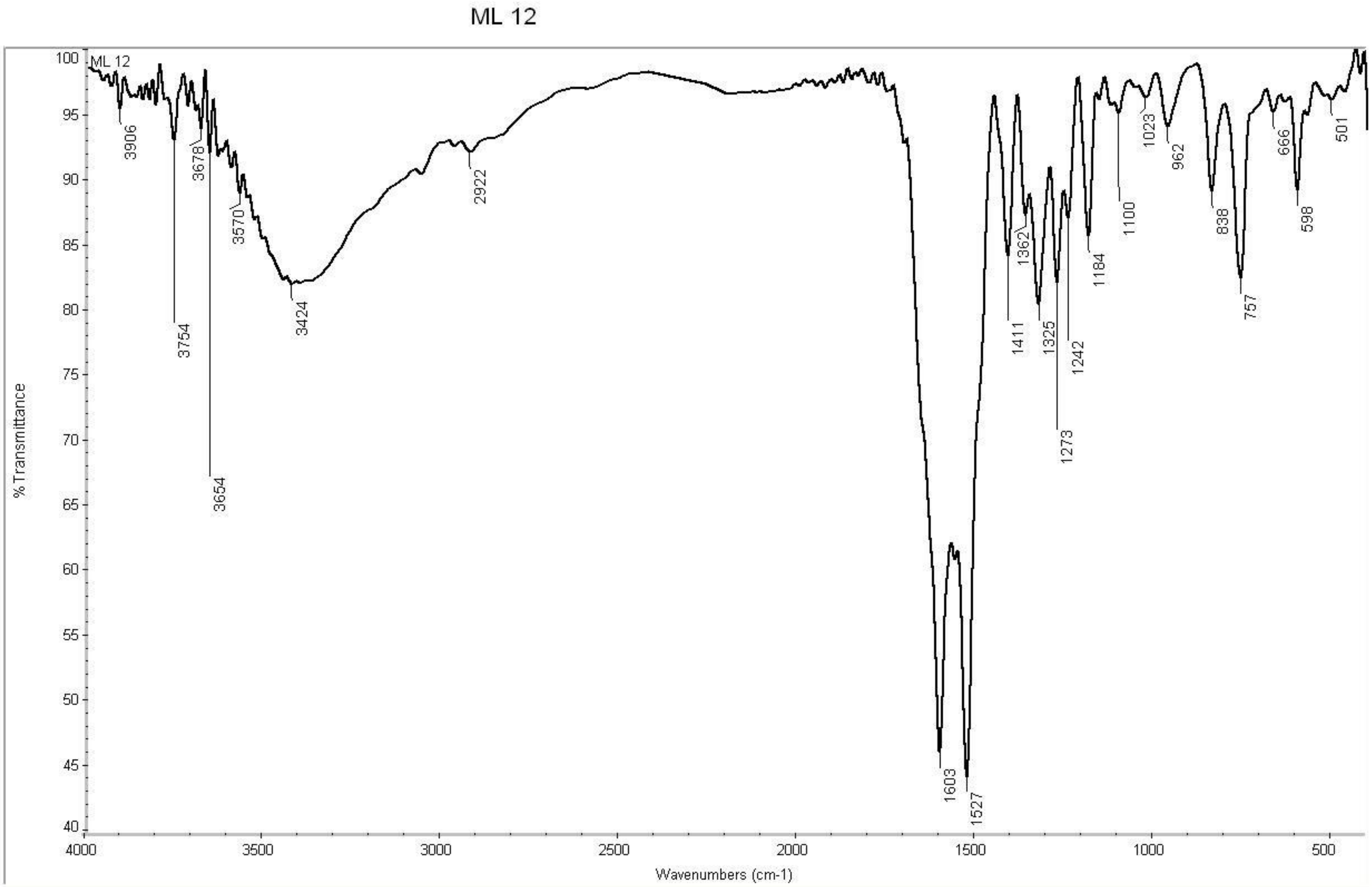

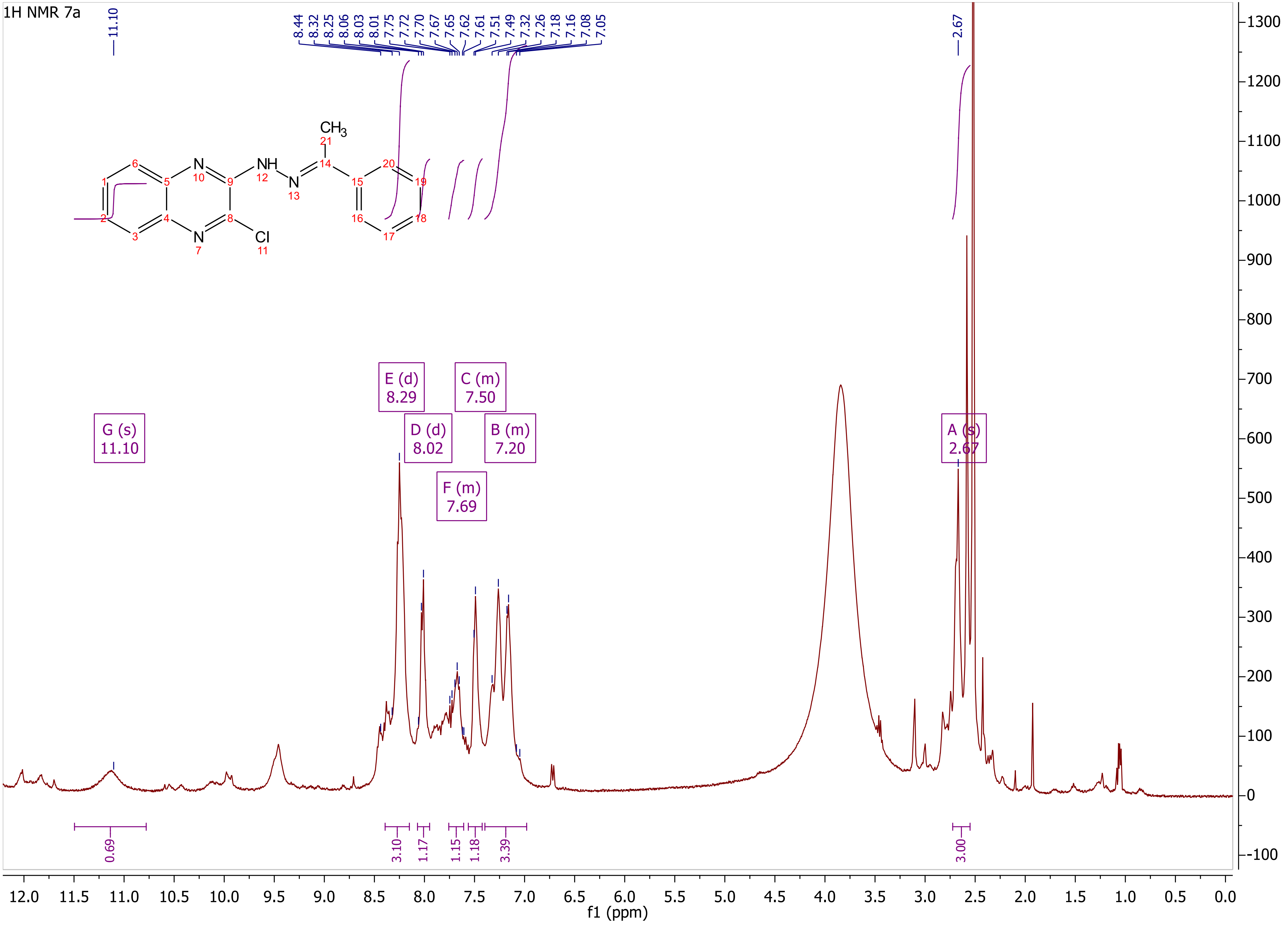

1H NMR 7a
11.10
8.44
8.32
8.25
8.06
8.03
8.01
7.75
7.72
7.70
7.67
7.65
7.62
7.61
7.51
7.49
7.32
7.26
7.18
7.16
7.08
7.05
2.67
CH3
NH
N
N
N
Cl
G (s)
11.10
E (d)
8.29
D (d)
8.02
F (m)
7.69
C (m)
7.50
B (m)
7.20
A (s)
2.67
0.69
3.10
1.17
1.15
1.18
3.39
3.00
f1 (ppm)

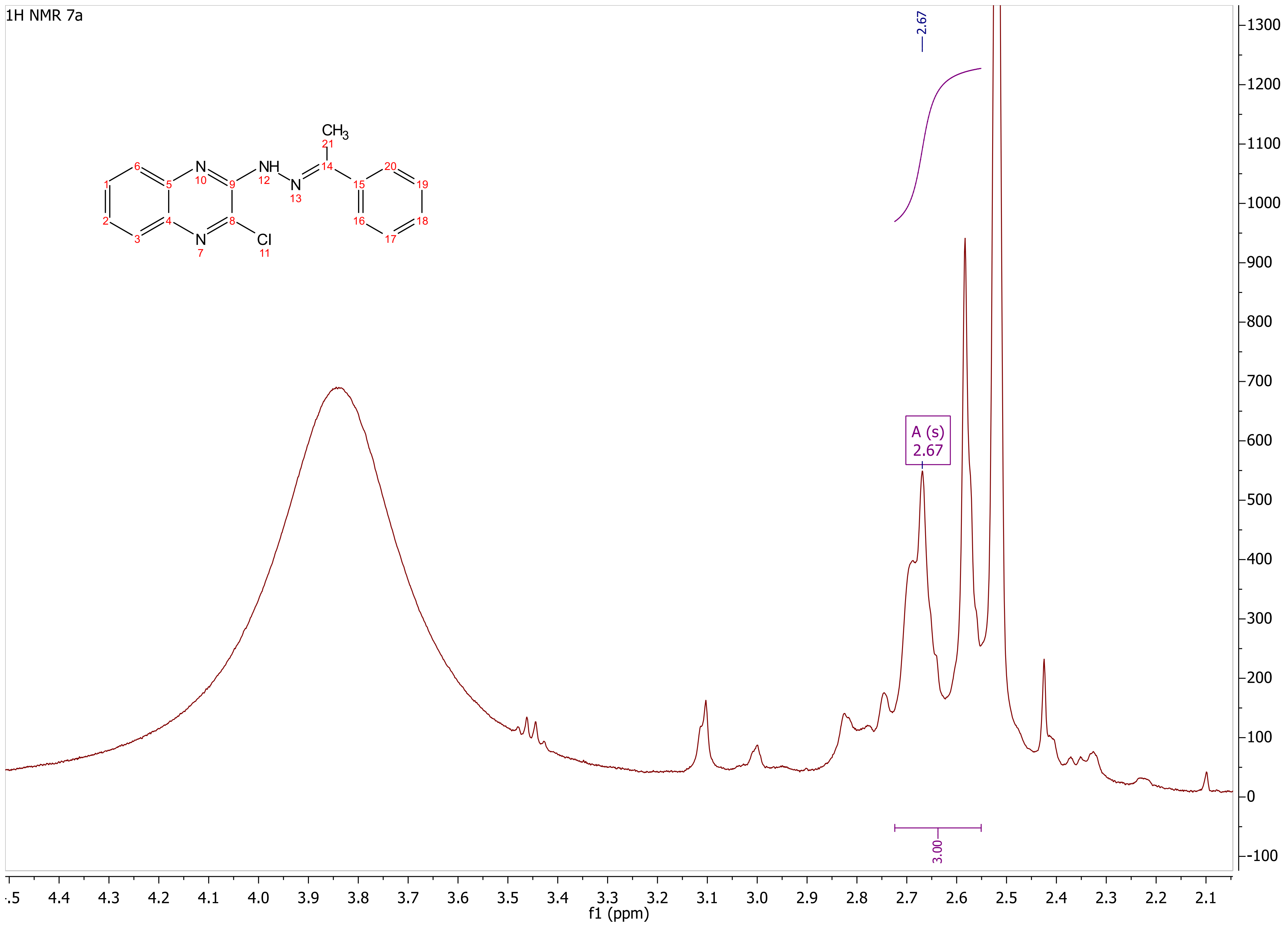

1H NMR 7a
CH3
Cl
NH
N
A (s)
2.67
3.00
f1 (ppm)

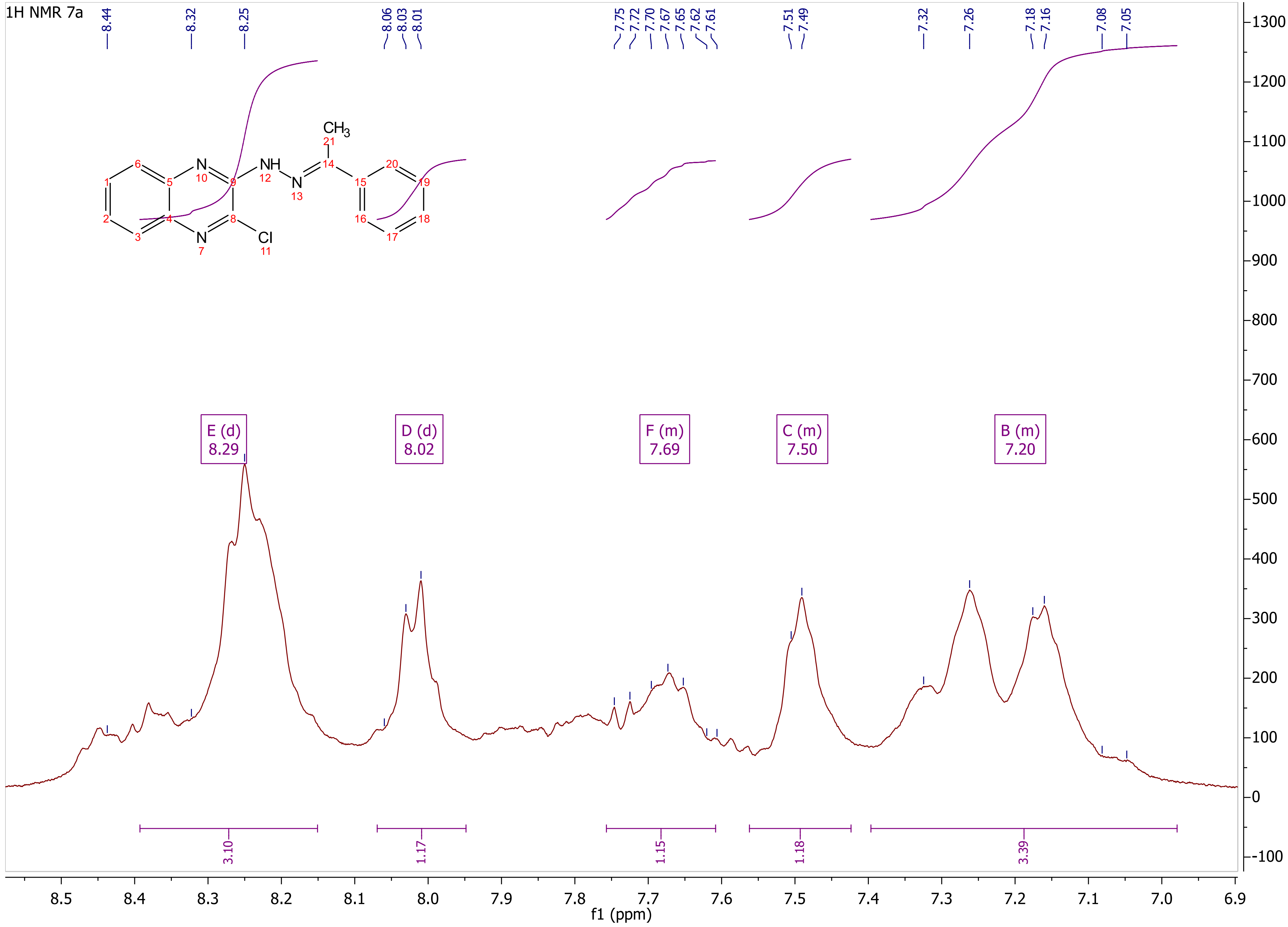

1H NMR 7a
8.44
8.32
8.25
8.06
8.03
8.01
7.75
7.72
7.70
7.67
7.65
7.62
7.61
7.51
7.49
7.32
7.26
7.18
7.16
7.08
7.05
CH3
NH
N
Cl
E (d) 8.29
D (d) 8.02
F (m) 7.69
C (m) 7.50
B (m) 7.20
3.10
1.17
1.15
1.18
3.39
f1 (ppm)

IBRAHIM-HASSAN-7 #176 RT: 2.96 AV: 1 SB: 2 4.45 , 4.45 NL: 2.36E2
T: {0,0} + c EI Full ms [40.00-1000.00]

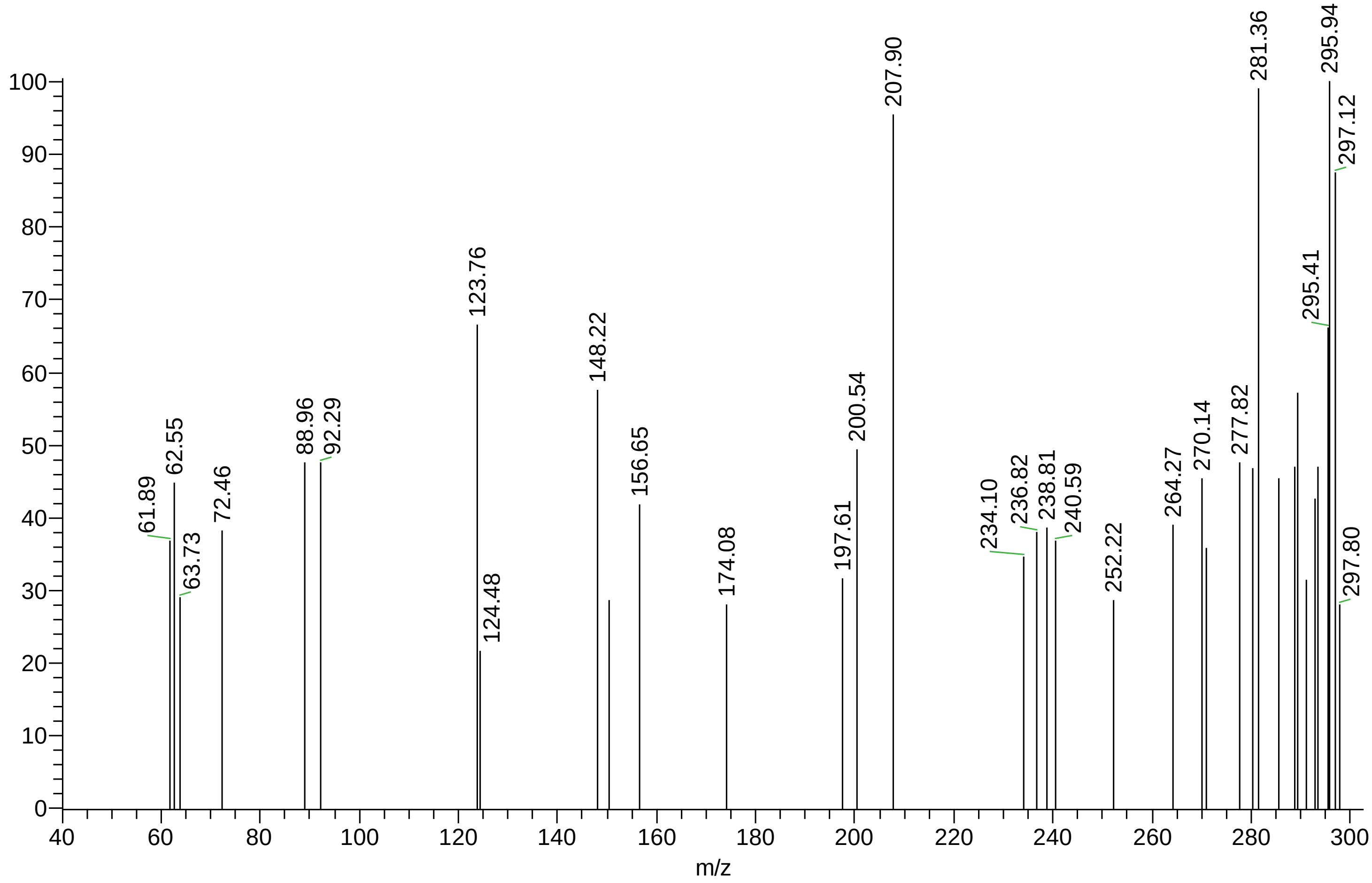

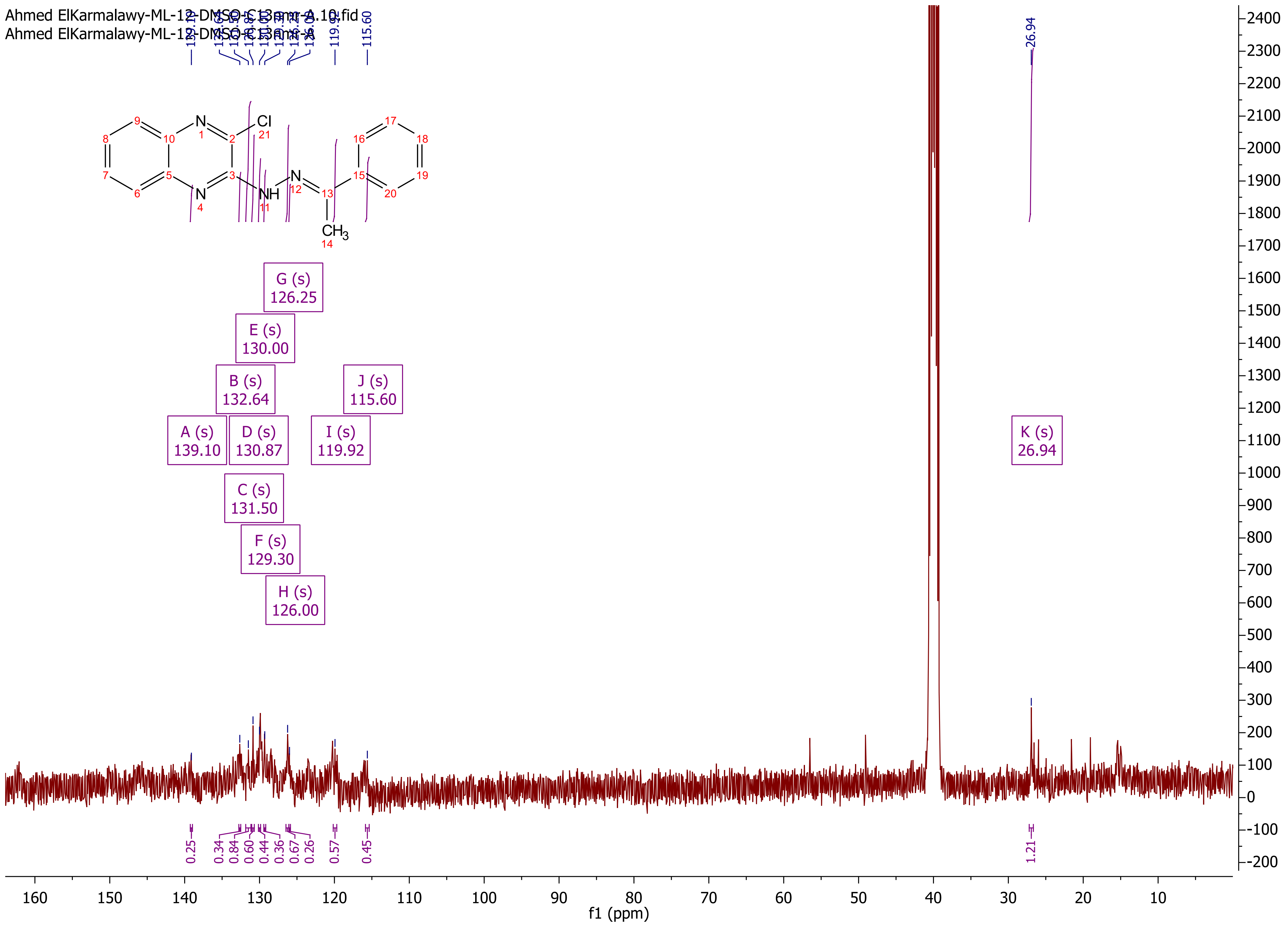
Ahmed ElKarmalawy-ML-12-DMSO-C13nmr-A.10.fid
Ahmed ElKarmalawy-ML-12-DMSO-C13nmr-A
115.60
119.92
26.94
Cl
NH
CH3
A (s) 139.10
B (s) 132.64
C (s) 131.50
D (s) 130.87
E (s) 130.00
F (s) 129.30
G (s) 126.25
H (s) 126.00
I (s) 119.92
J (s) 115.60
K (s) 26.94
0.25
0.34
0.84
0.60
0.44
0.36
0.67
0.26
0.57
0.45
1.21
f1 (ppm)

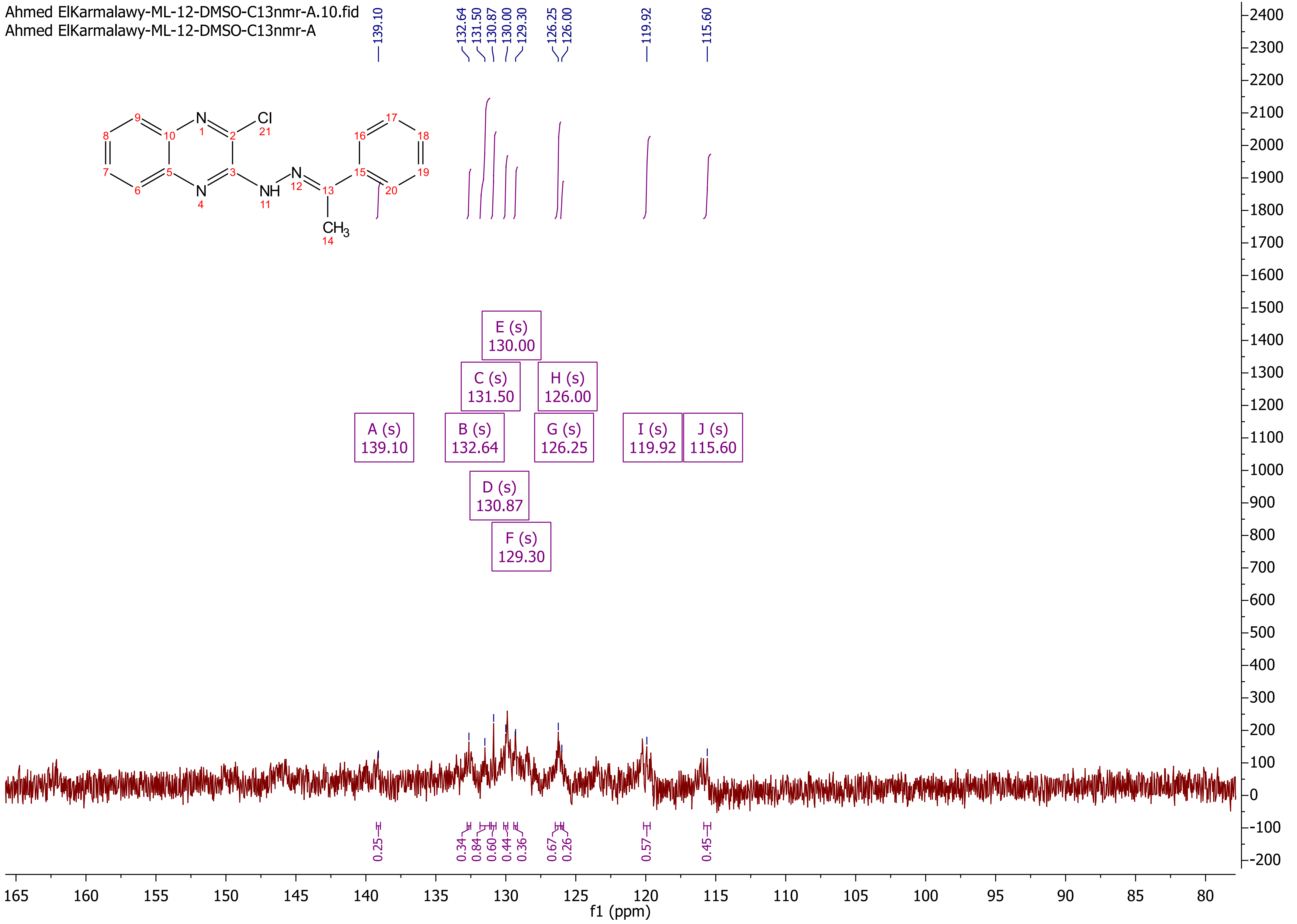
Ahmed ElKarmalawy-ML-12-DMSO-C13nmr-A.10.fid
Ahmed ElKarmalawy-ML-12-DMSO-C13nmr-A
139.10
132.64
131.50
130.87
130.00
129.30
126.25
126.00
119.92
115.60
N
Cl
NH
N
CH3
E (s) 130.00
C (s) 131.50
H (s) 126.00
A (s) 139.10
B (s) 132.64
G (s) 126.25
I (s) 119.92
J (s) 115.60
D (s) 130.87
F (s) 129.30
0.25
0.34
0.84
0.60
0.44
0.36
0.67
0.26
0.57
0.45
f1 (ppm)

Ahmed ElKarmalawy-ML-12-DMSO-C13nmr-A.10.fid
Ahmed ElKarmalawy-ML-12-DMSO-C13nmr-A

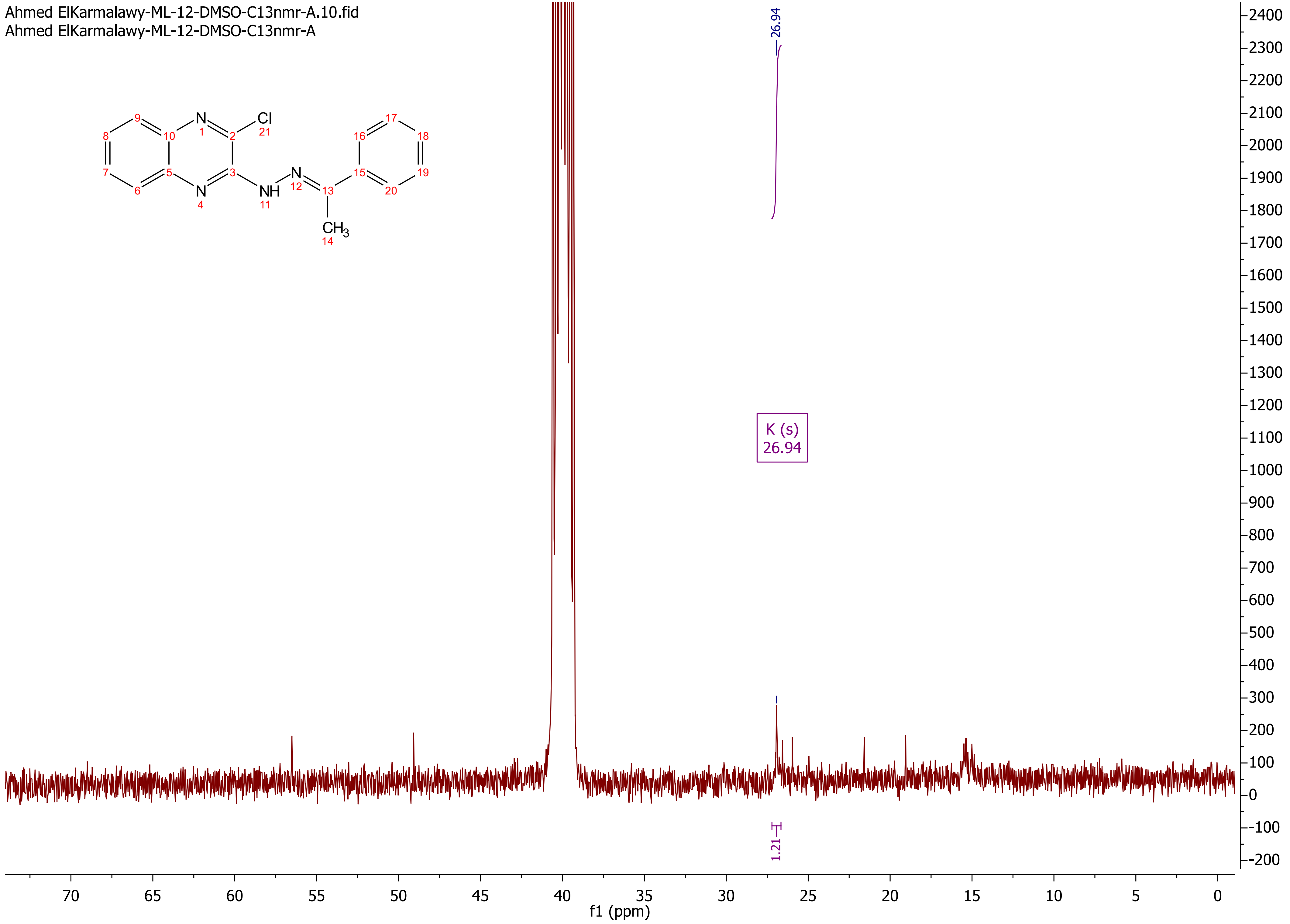

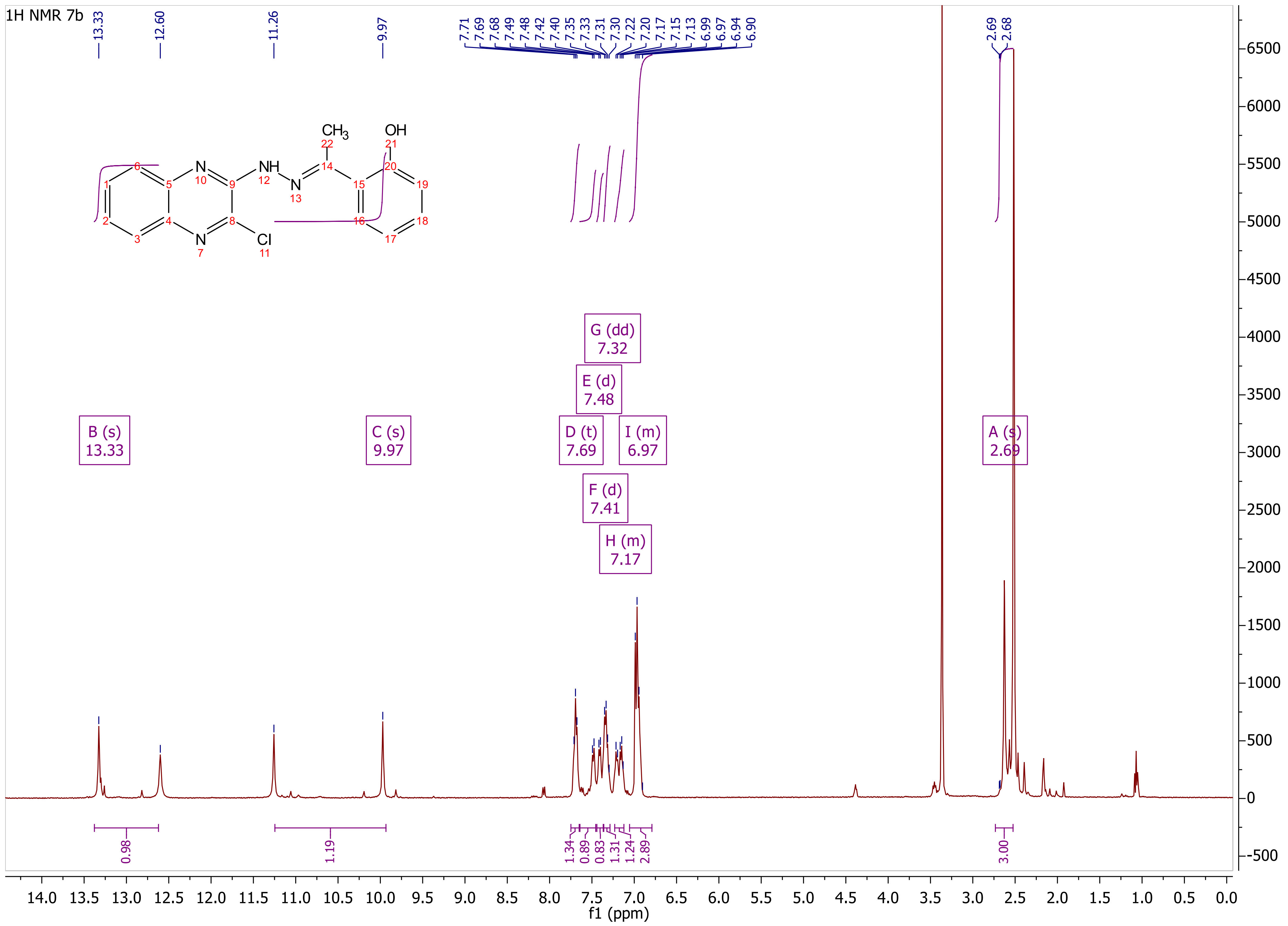

1H NMR 7b
13.33
12.60
11.26
9.97
7.71 7.69 7.68 7.49 7.48 7.42 7.40 7.35 7.33 7.31 7.30 7.22 7.20 7.17 7.15 7.13 6.99 6.97 6.94 6.90
2.69 2.68
CH3
OH
NH
N
N
N
Cl
G (dd) 7.32
E (d) 7.48
B (s) 13.33
C (s) 9.97
D (t) 7.69
I (m) 6.97
A (s) 2.69
F (d) 7.41
H (m) 7.17
0.98
1.19
1.34 0.89 0.83 1.31 1.24 2.89
3.00
f1 (ppm)

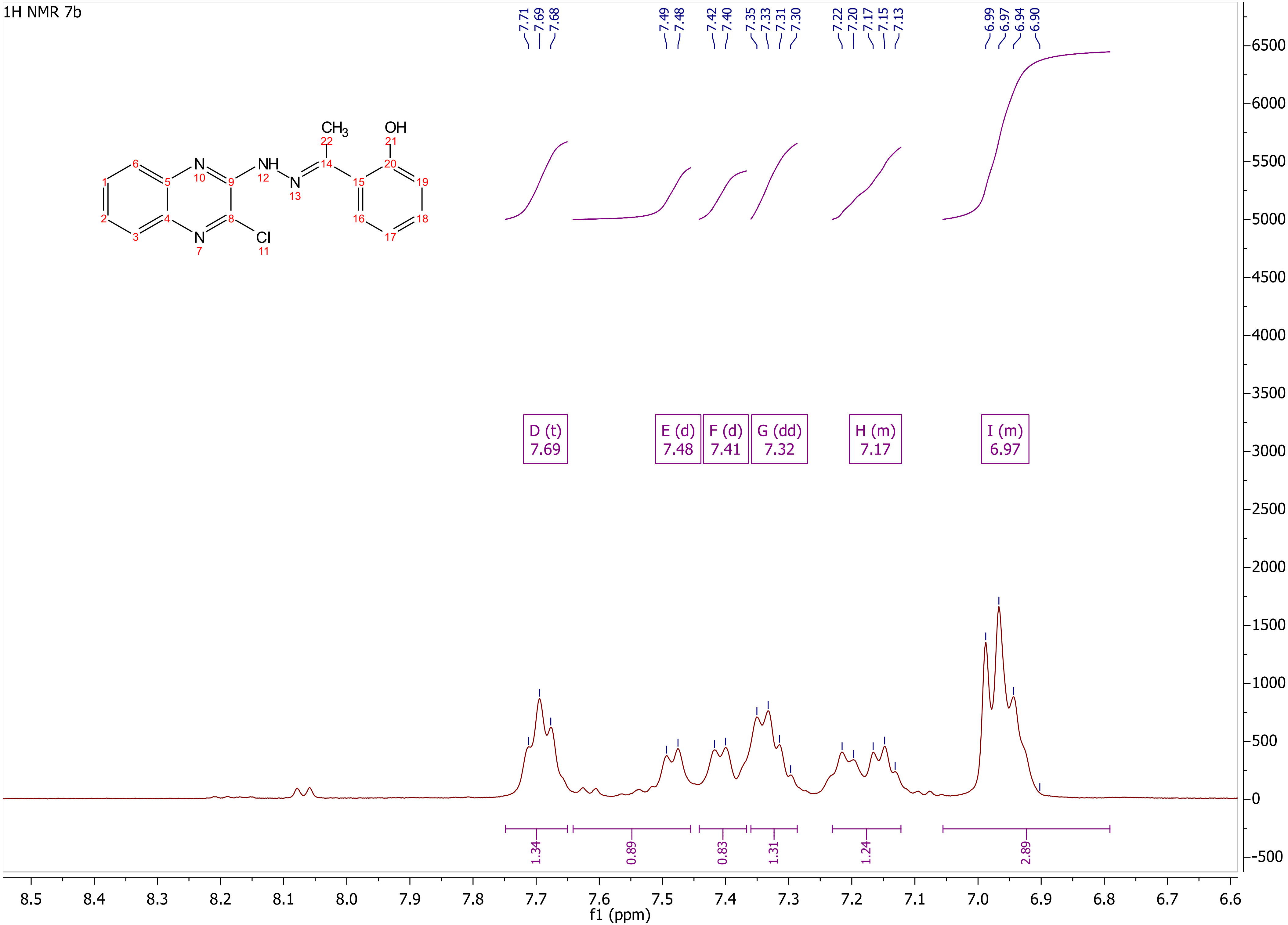
1H NMR 7b
7.71
7.69
7.68
7.49
7.48
7.42
7.40
7.35
7.33
7.31
7.30
7.22
7.20
7.17
7.15
7.13
6.99
6.97
6.94
6.90
CH3
OH
NH
N
Cl
D (t) 7.69
E (d) 7.48
F (d) 7.41
G (dd) 7.32
H (m) 7.17
I (m) 6.97
1.34
0.89
0.83
1.31
1.24
2.89
f1 (ppm)

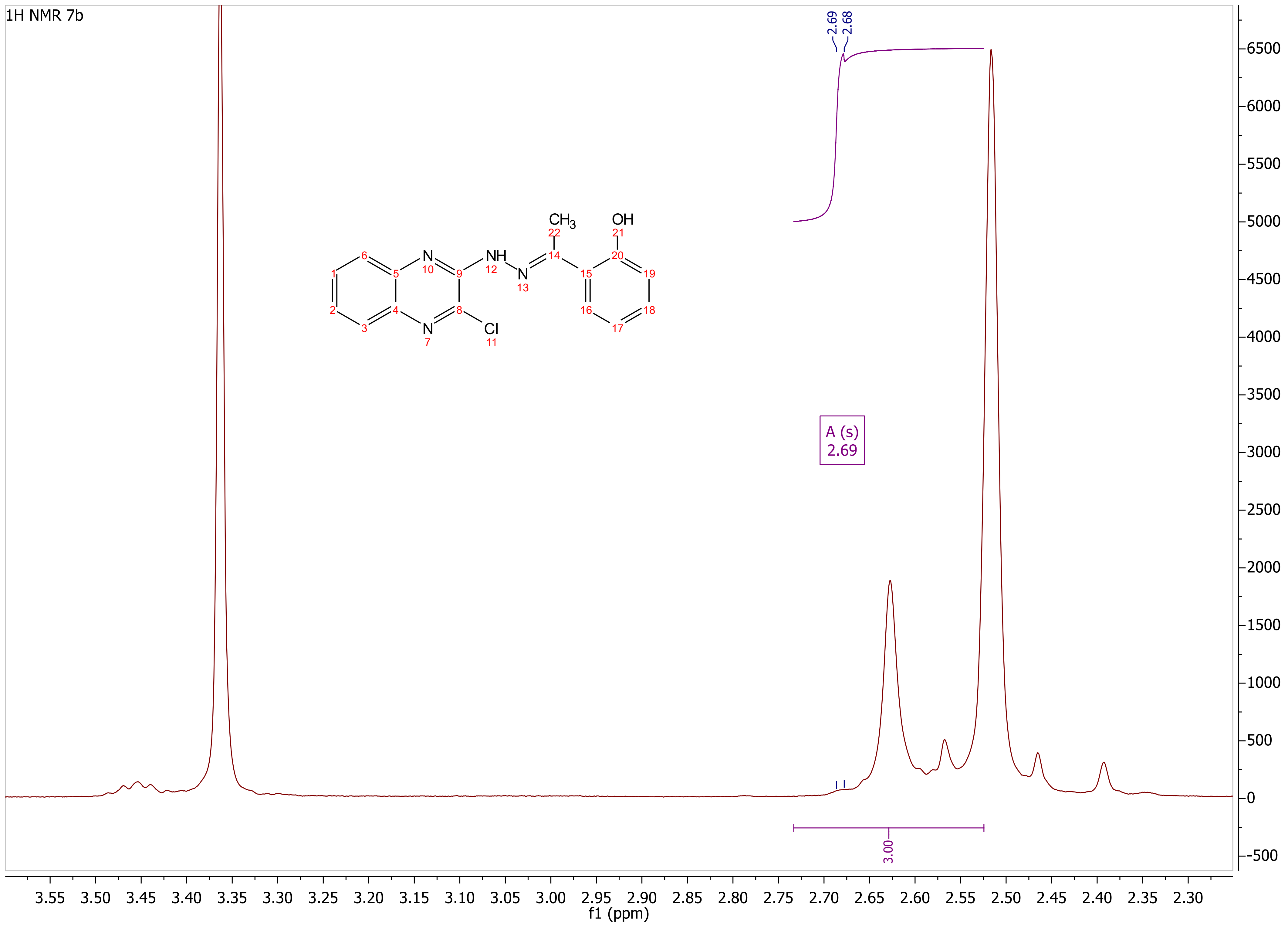
1H NMR 7b
2.69
2.68
A (s)
2.69
3.00
CH3
OH
NH
N
N
N
Cl
f1 (ppm)

IBRAHIM-HASSAN-7B #108 RT: 1.82 AV: 1 SB: 2 4.45 , 4.45 NL: 6.51E2
T: {0,0} + c EI Full ms [40.00-1000.00]

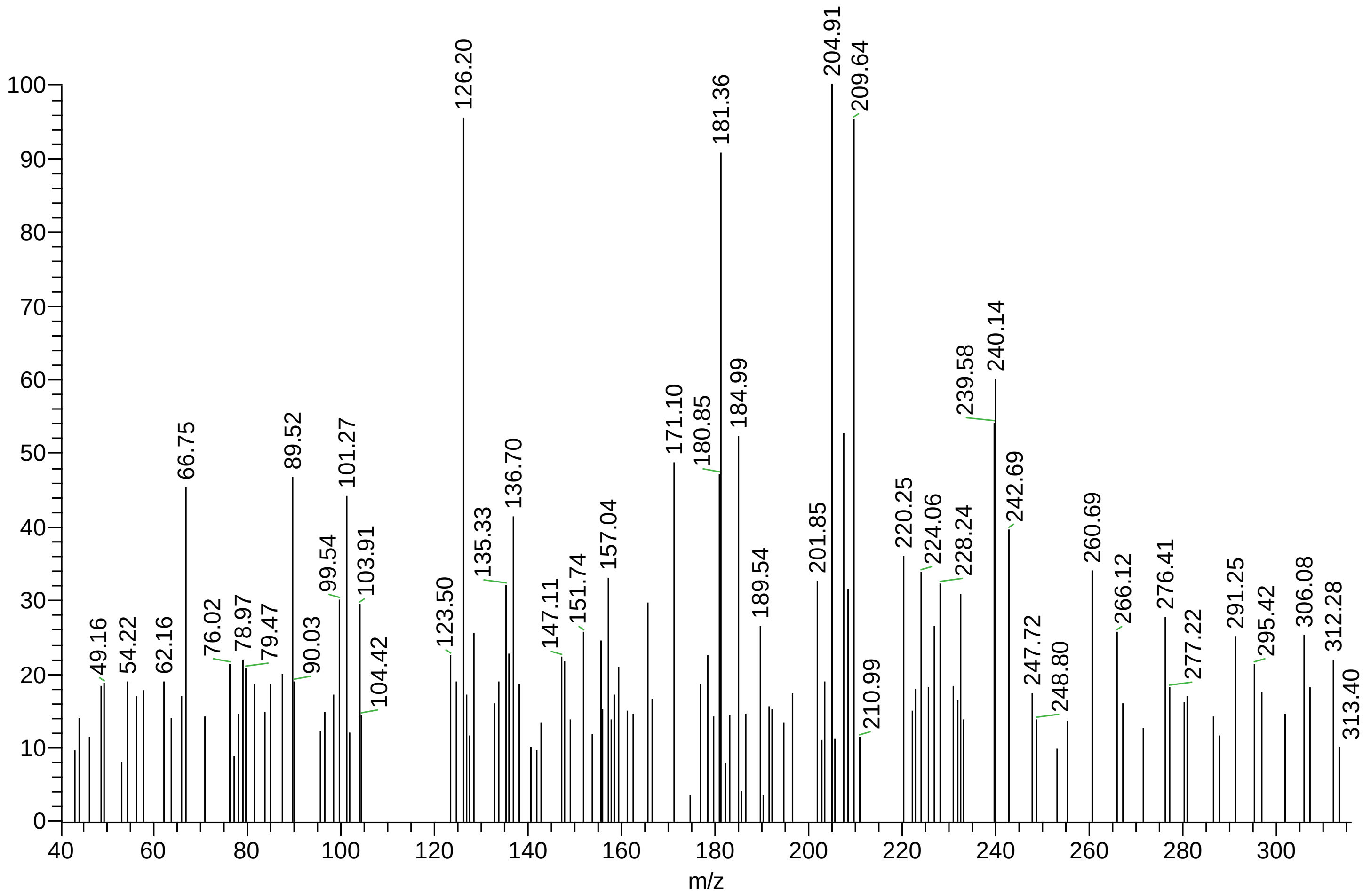

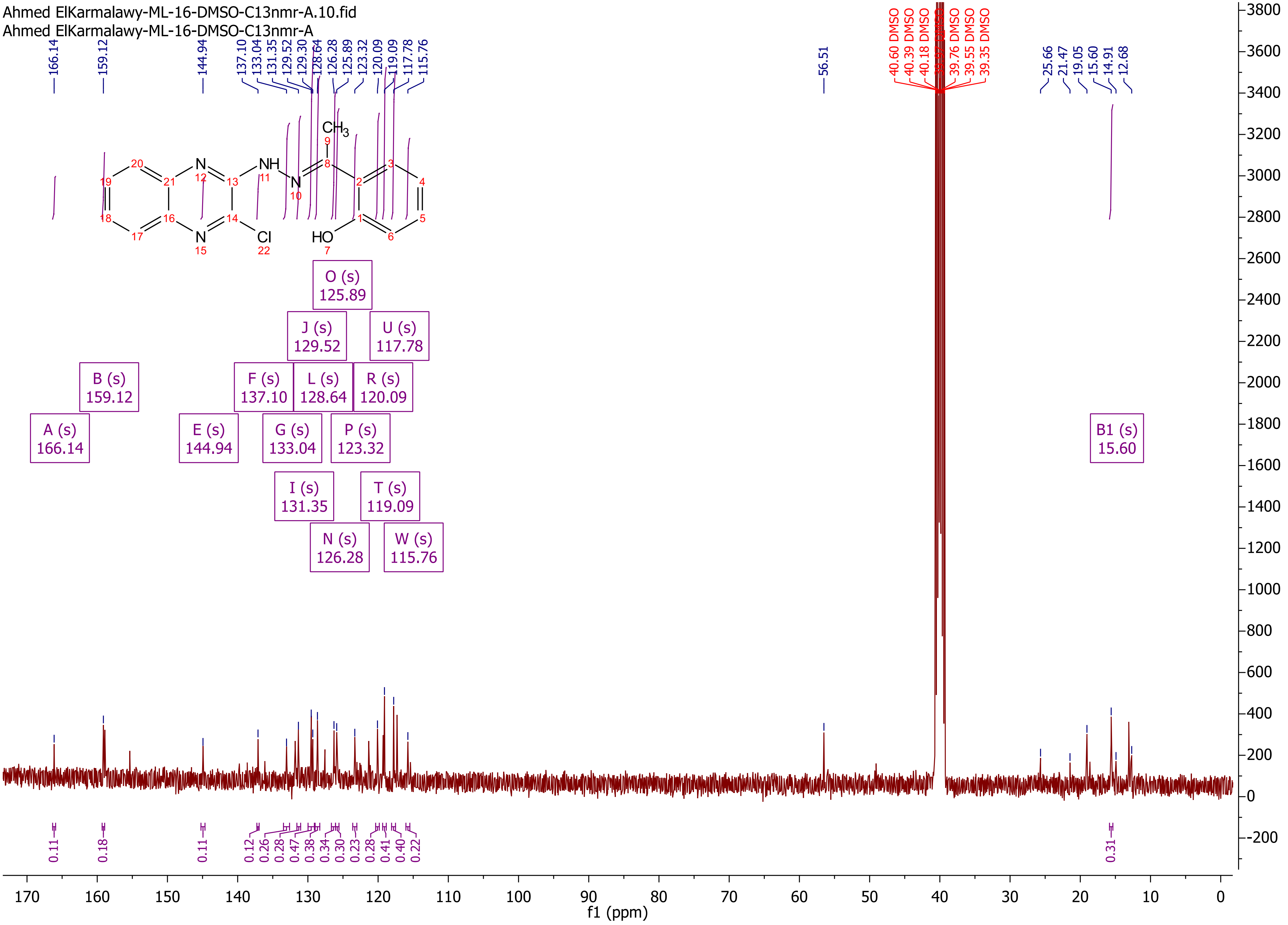
Ahmed ElKarmalawy-ML-16-DMSO-C13nmr-A.10.fid
Ahmed ElKarmalawy-ML-16-DMSO-C13nmr-A
166.14
159.12
144.94
137.10
133.04
131.35
129.52
129.30
128.64
126.28
125.89
123.32
120.09
119.09
117.78
115.76
56.51
40.60 DMSO
40.39 DMSO
40.18 DMSO
39.76 DMSO
39.55 DMSO
39.35 DMSO
25.66
21.47
19.05
15.60
14.91
12.68
CH3
NH
N
HO
Cl
A (s) 166.14
B (s) 159.12
E (s) 144.94
F (s) 137.10
G (s) 133.04
I (s) 131.35
J (s) 129.52
L (s) 128.64
N (s) 126.28
O (s) 125.89
P (s) 123.32
R (s) 120.09
T (s) 119.09
U (s) 117.78
W (s) 115.76
B1 (s) 15.60
0.11
0.18
0.11
0.12
0.26
0.28
0.47
0.38
0.34
0.30
0.23
0.28
0.41
0.40
0.22
0.31
f1 (ppm)

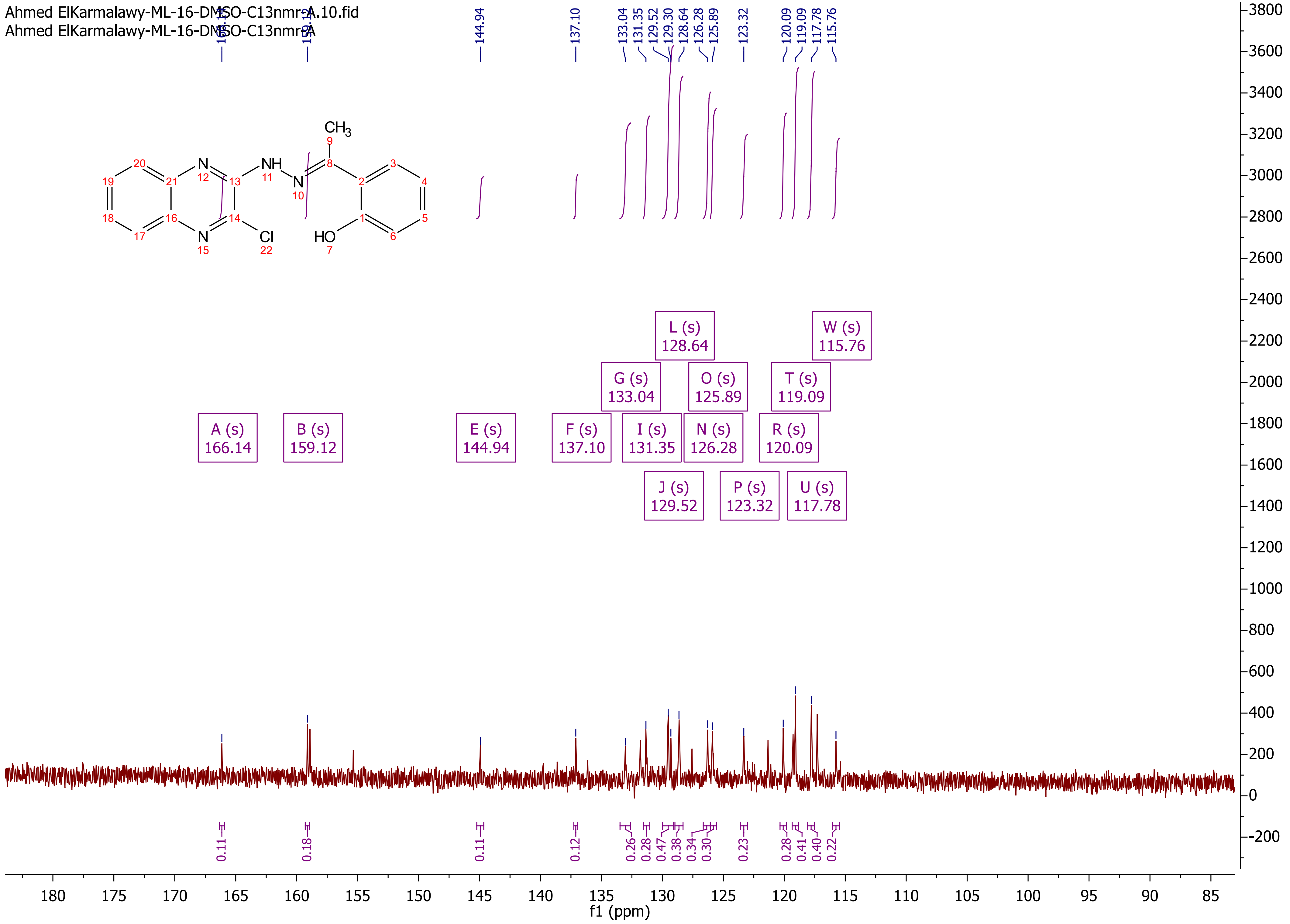
Ahmed ElKarmalawy-ML-16-DMSO-C13nmr-A.10.fid
Ahmed ElKarmalawy-ML-16-DMSO-C13nmr-A
144.94
137.10
133.04
131.35
129.52
129.30
128.64
126.28
125.89
123.32
120.09
119.09
117.78
115.76
CH3
NH
N
N
N
Cl
HO
A (s) 166.14
B (s) 159.12
E (s) 144.94
F (s) 137.10
G (s) 133.04
I (s) 131.35
J (s) 129.52
L (s) 128.64
N (s) 126.28
O (s) 125.89
P (s) 123.32
R (s) 120.09
T (s) 119.09
U (s) 117.78
W (s) 115.76
0.11
0.18
0.11
0.12
0.26
0.28
0.47
0.38
0.34
0.30
0.23
0.28
0.41
0.40
0.22
f1 (ppm)

Ahmed ElKarmalawy-ML-16-DMSO-C13nmr-A.10.fid
Ahmed ElKarmalawy-ML-16-DMSO-C13nmr-A

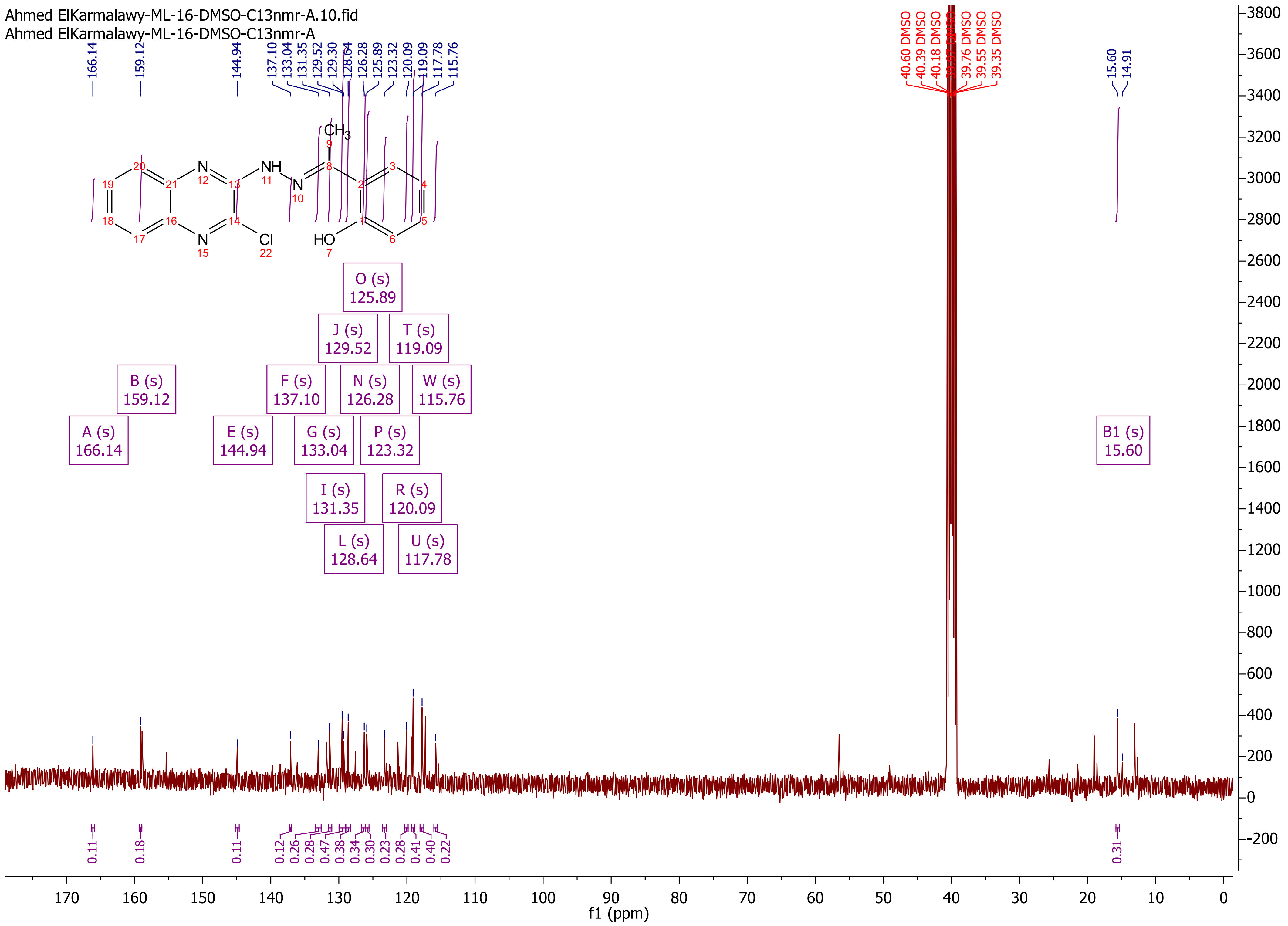

IR of compound 8

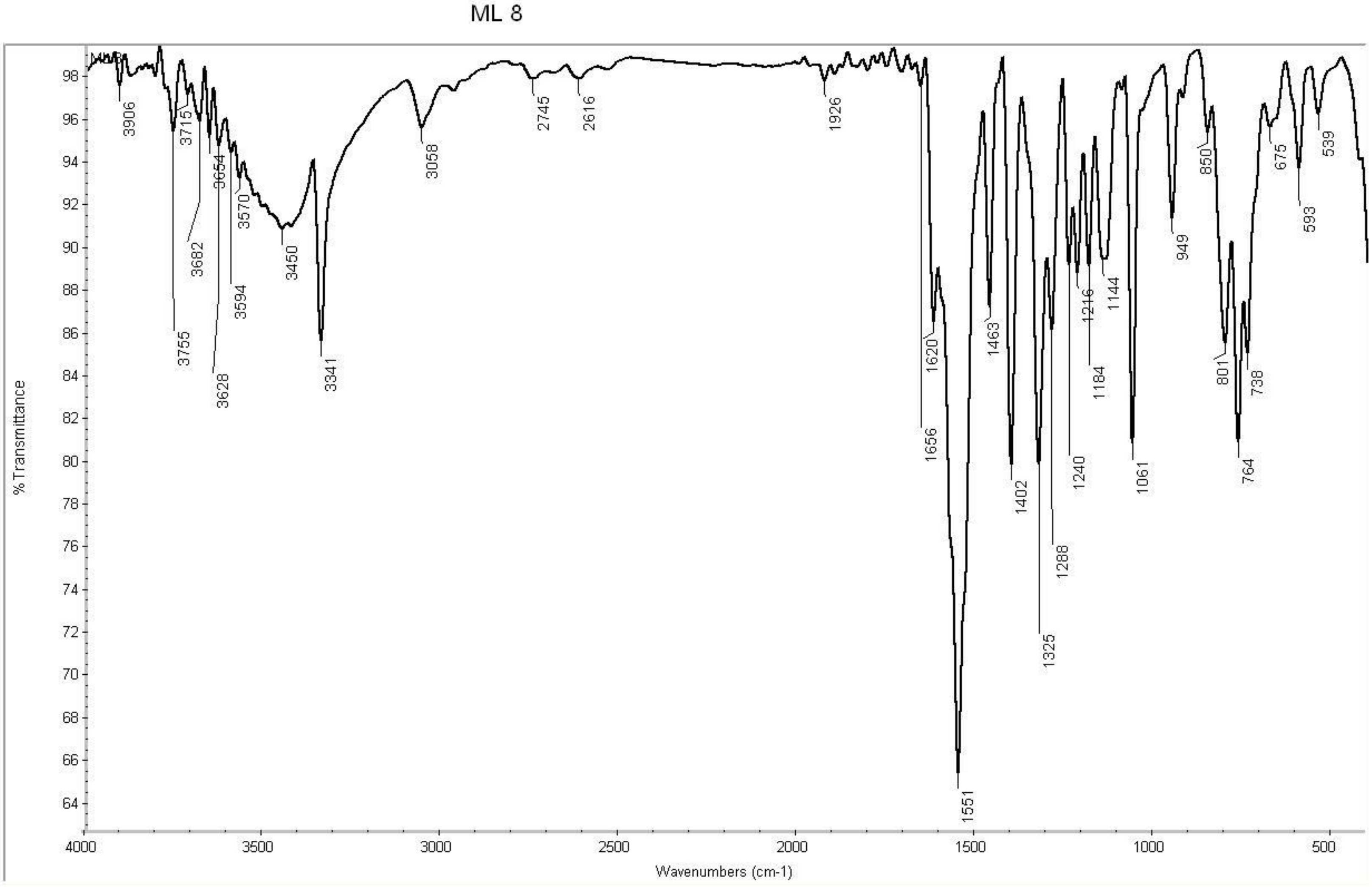

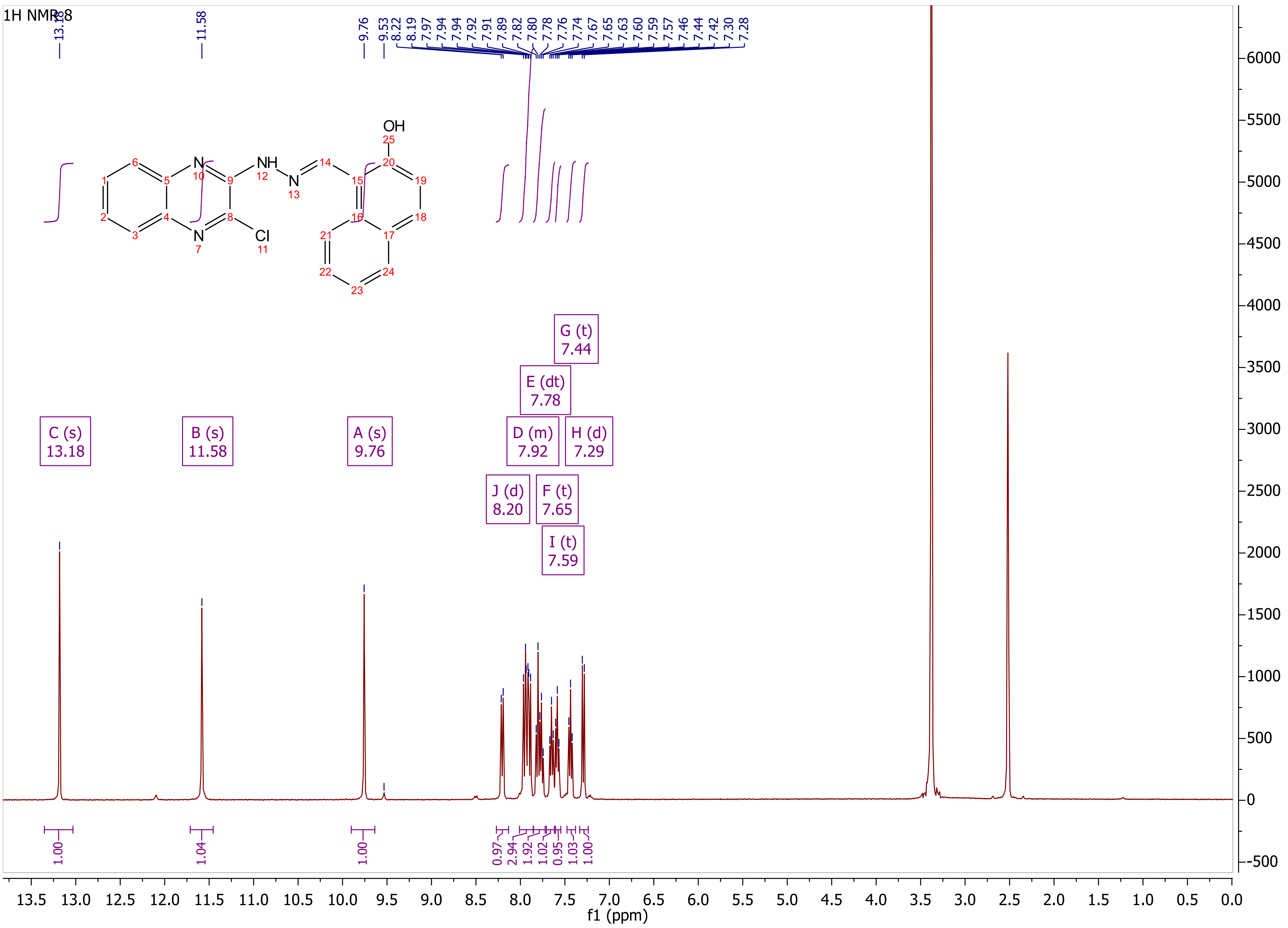
1H NMR 8
13.18
11.58
9.76
9.53
8.22
8.19
7.97
7.94
7.94
7.92
7.91
7.89
7.82
7.80
7.78
7.76
7.74
7.67
7.65
7.63
7.60
7.59
7.57
7.46
7.44
7.42
7.30
7.28
OH
NH
N
N
N
Cl
C (s) 13.18
B (s) 11.58
A (s) 9.76
J (d) 8.20
D (m) 7.92
E (dt) 7.78
F (t) 7.65
I (t) 7.59
G (t) 7.44
H (d) 7.29
1.00
1.04
1.00
0.97
2.94
1.92
1.02
0.95
1.03
1.00
f1 (ppm)

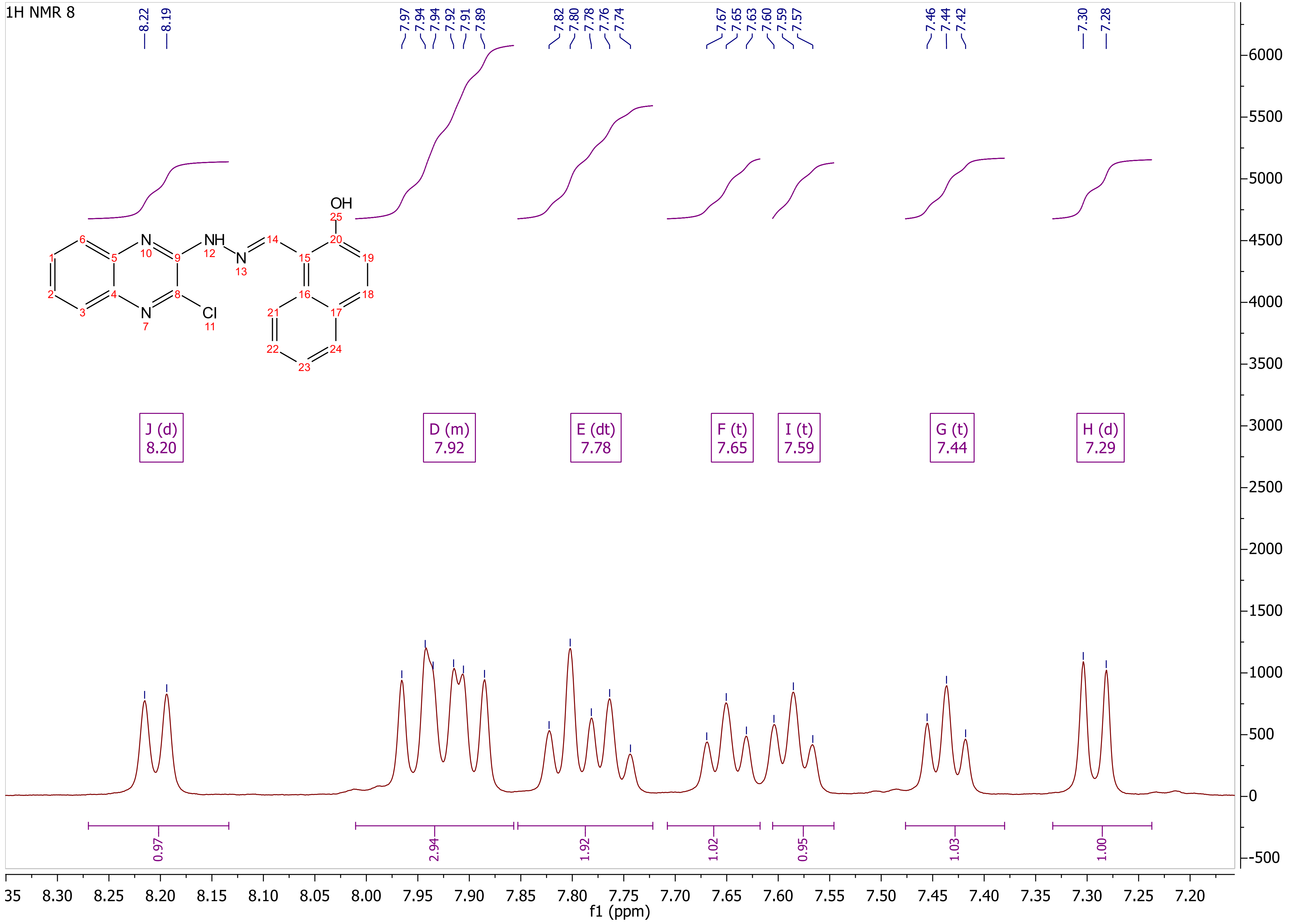
1H NMR 8
8.22
8.19
7.97
7.94
7.94
7.92
7.91
7.89
7.82
7.80
7.78
7.76
7.74
7.67
7.65
7.63
7.60
7.59
7.57
7.46
7.44
7.42
7.30
7.28
OH
NH
N
N
N
Cl
J (d) 8.20
D (m) 7.92
E (dt) 7.78
F (t) 7.65
I (t) 7.59
G (t) 7.44
H (d) 7.29
0.97
2.94
1.92
1.02
0.95
1.03
1.00
f1 (ppm)

13 CNMR of comp. 8

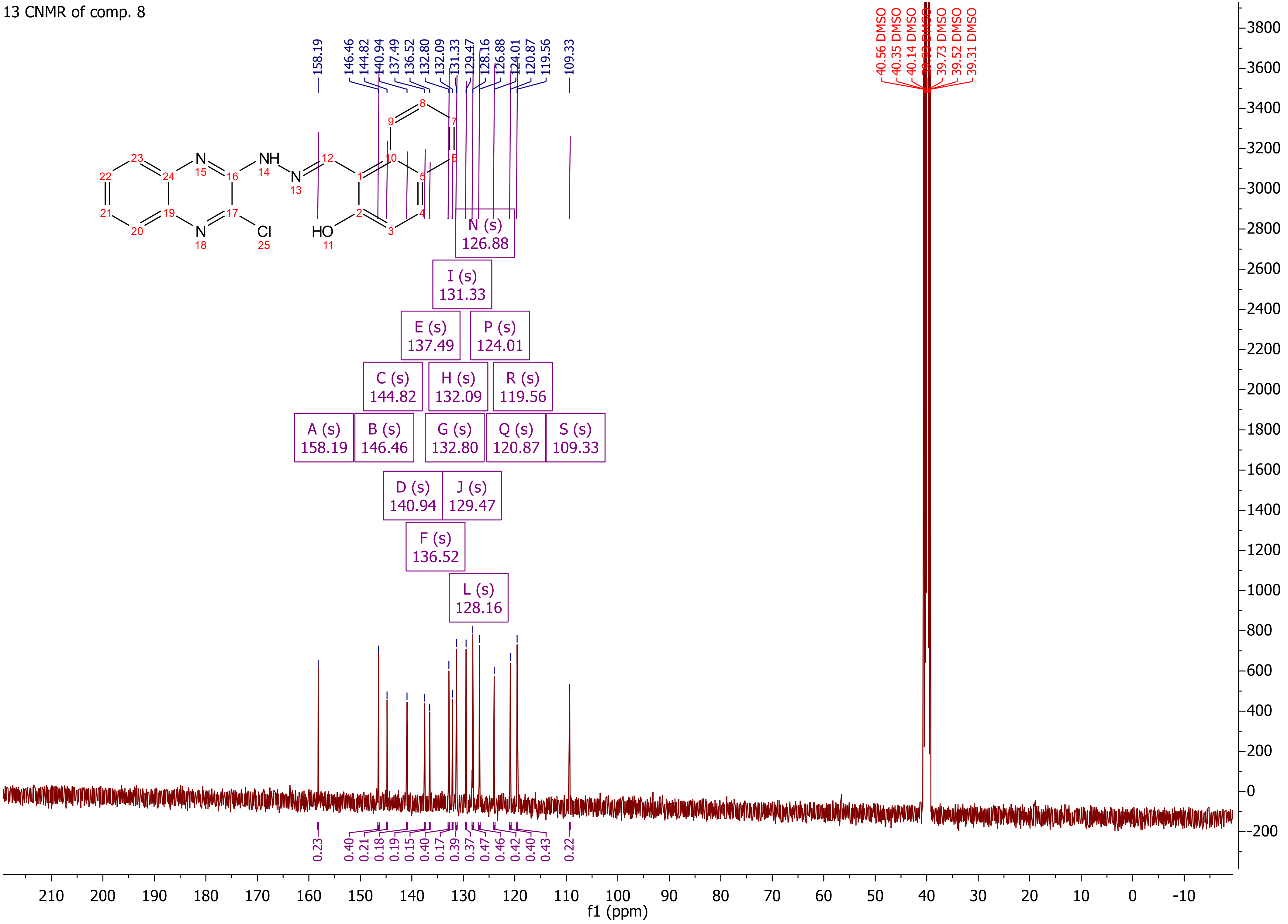

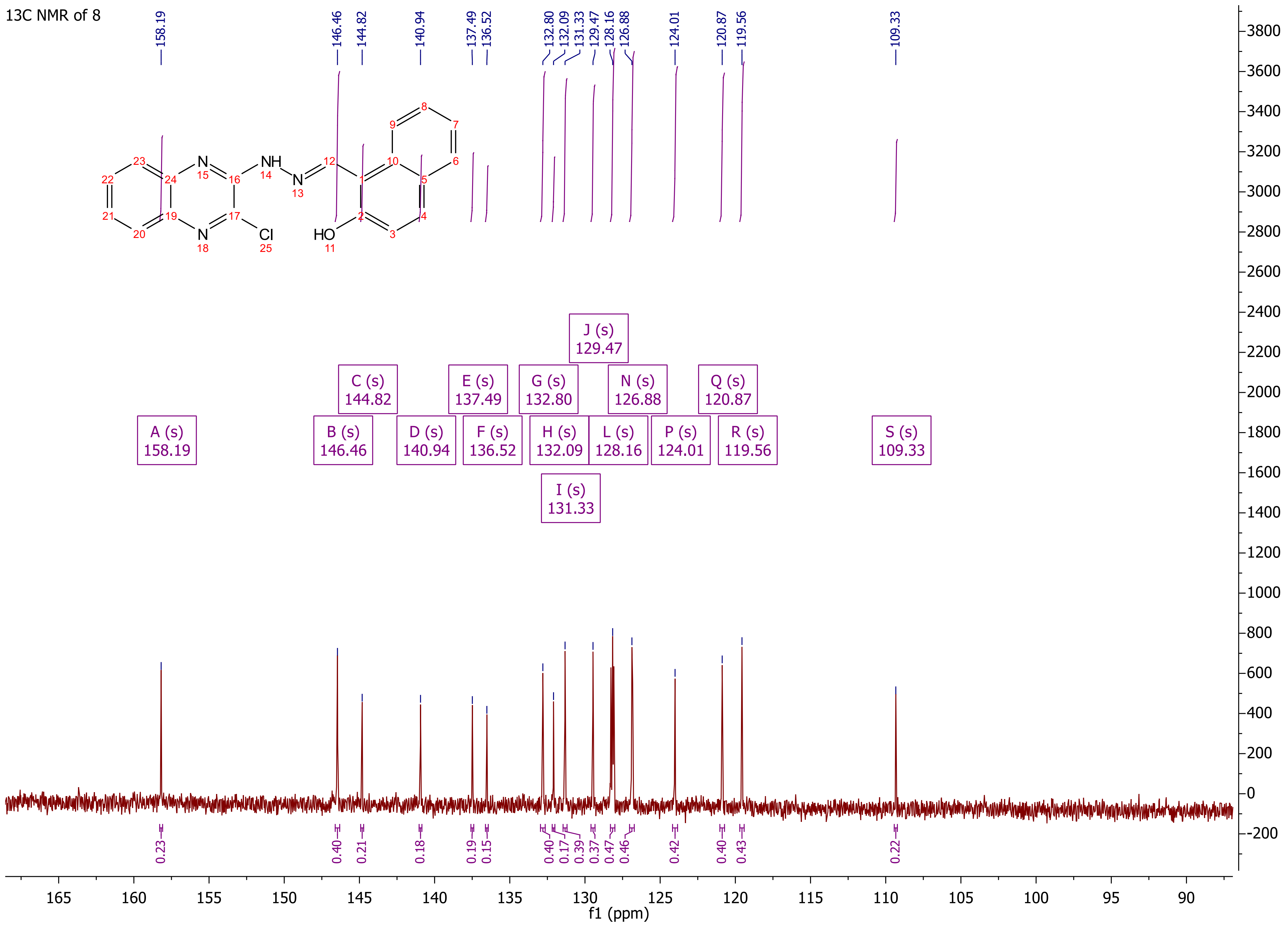
13C NMR of 8
158.19
146.46
144.82
140.94
137.49
136.52
132.80
132.09
131.33
129.47
128.16
126.88
124.01
120.87
119.56
109.33
A (s) 158.19
B (s) 146.46
C (s) 144.82
D (s) 140.94
E (s) 137.49
F (s) 136.52
G (s) 132.80
H (s) 132.09
I (s) 131.33
J (s) 129.47
L (s) 128.16
N (s) 126.88
P (s) 124.01
Q (s) 120.87
R (s) 119.56
S (s) 109.33
0.23
0.40
0.21
0.18
0.19
0.15
0.40
0.17
0.39
0.37
0.47
0.46
0.42
0.40
0.43
0.22
f1 (ppm)

13C NMR of 8

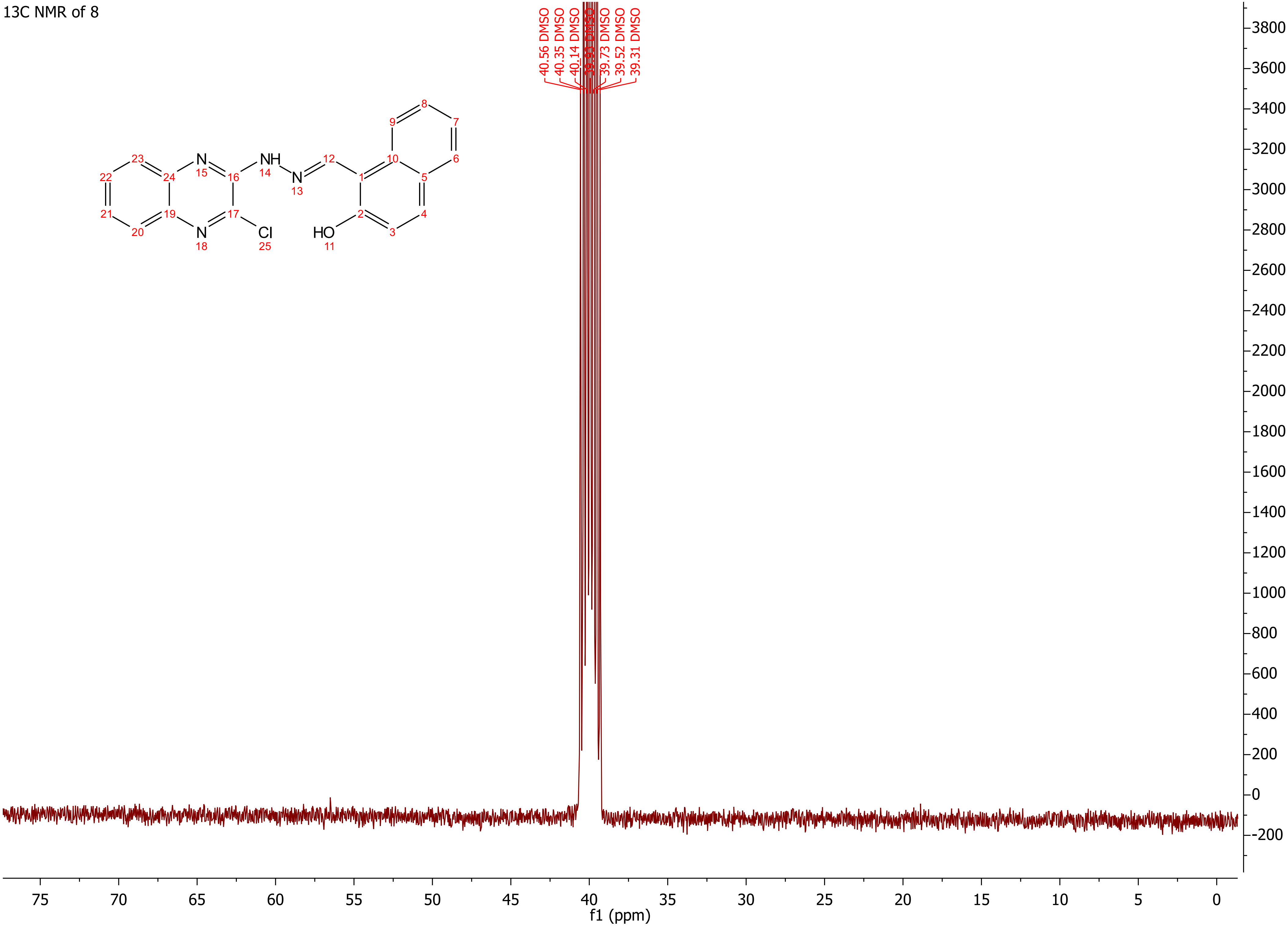

40.56 DMSO
40.35 DMSO
40.14 DMSO
39.93 DMSO
39.73 DMSO
39.52 DMSO
39.31 DMSO
NH
N
Cl
HO
f1 (ppm)

IR of compound 9

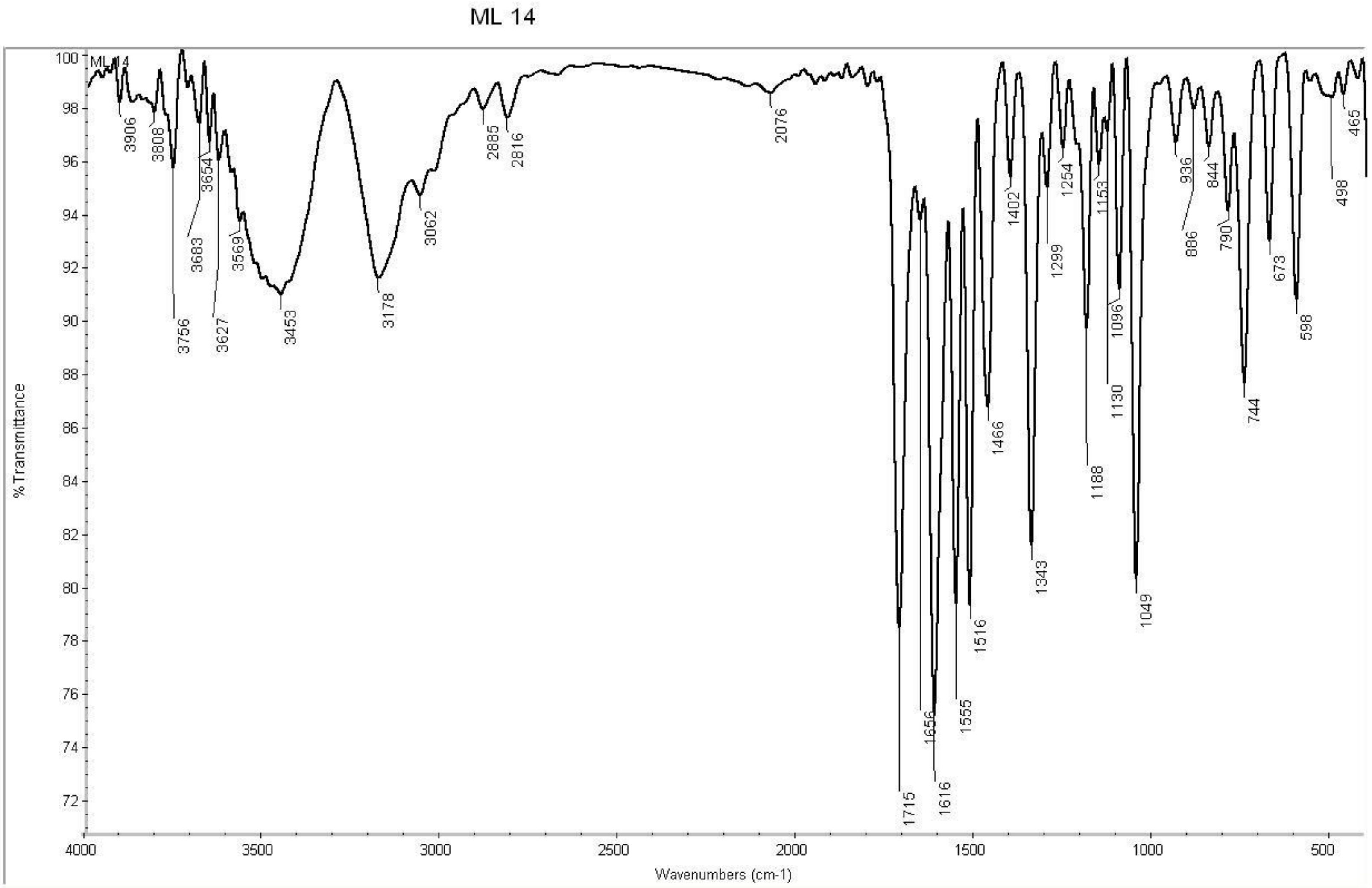

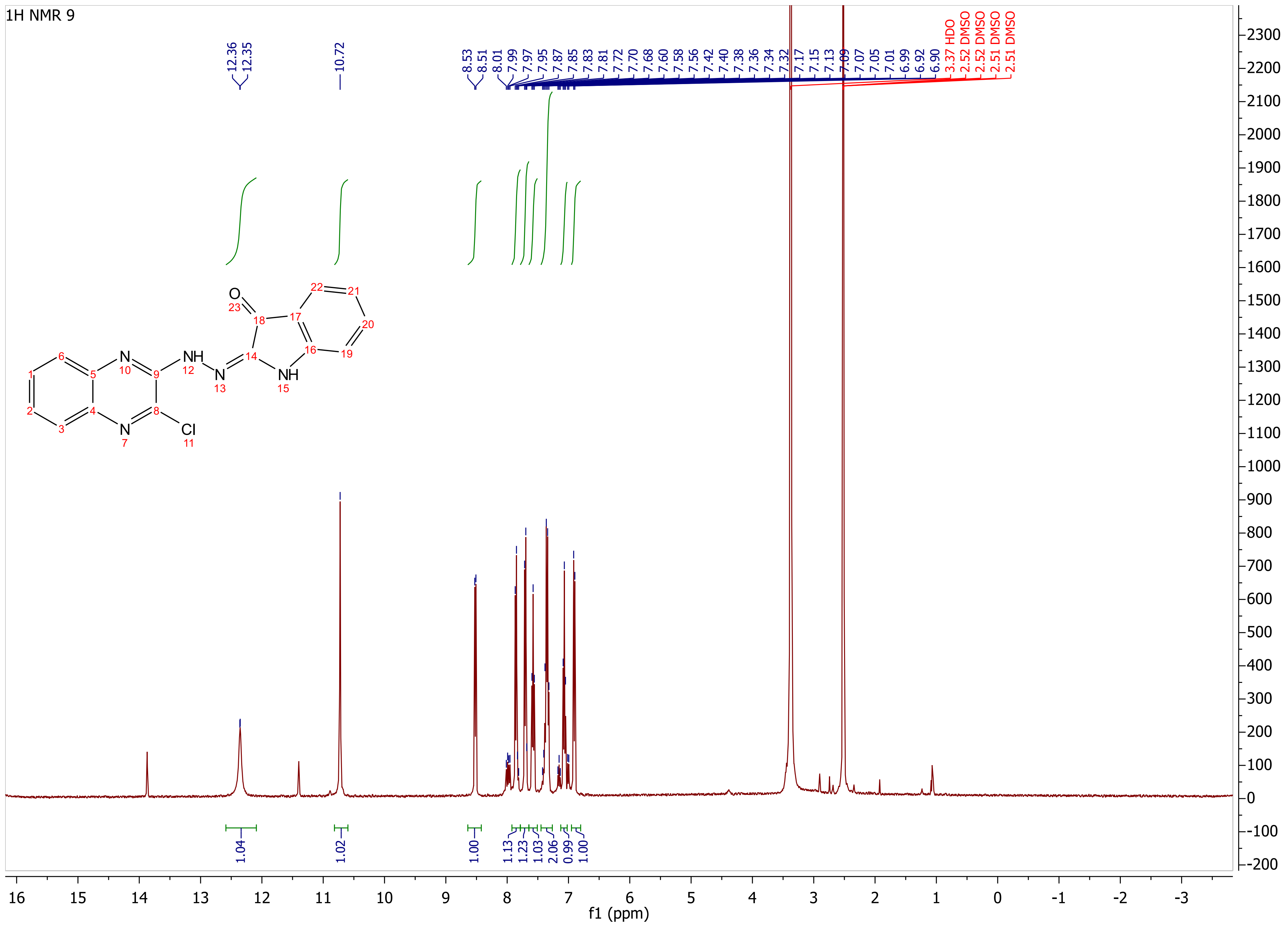
1H NMR 9
12.36
12.35
10.72
8.53
8.51
8.01
7.99
7.97
7.95
7.87
7.85
7.83
7.81
7.72
7.70
7.68
7.60
7.58
7.56
7.42
7.40
7.38
7.36
7.34
7.32
7.17
7.15
7.13
7.09
7.07
7.05
7.01
6.99
6.92
6.90
3.37 HDO
2.52 DMSO
2.52 DMSO
2.51 DMSO
2.51 DMSO
1.04
1.02
1.00
1.13
1.23
1.03
2.06
0.99
1.00
f1 (ppm)

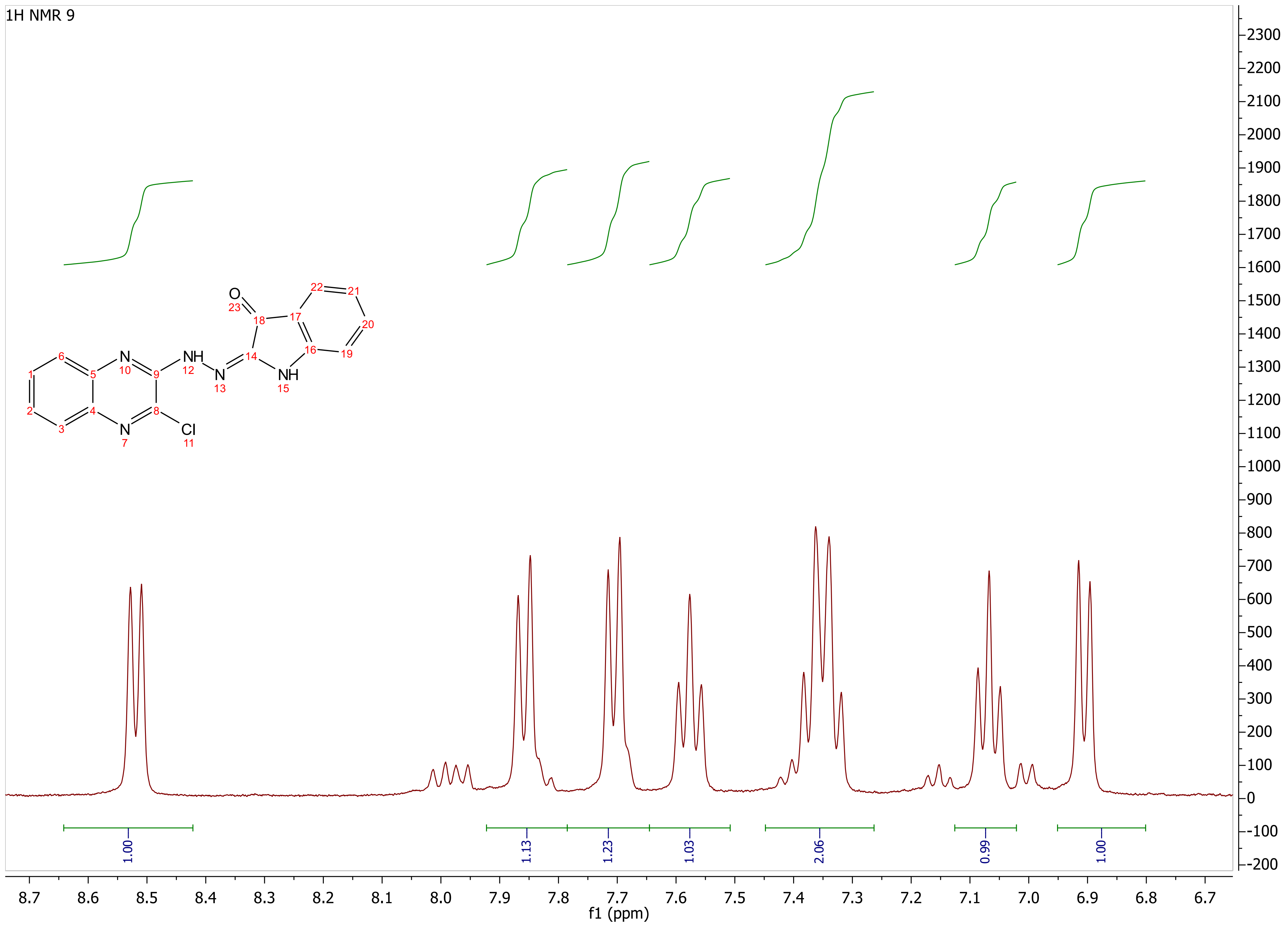

1H NMR 9
O
23
18
17
22
21
20
19
16
14
NH
15
N
13
NH
12
9
N
10
5
6
1
2
3
4
N
7
8
Cl
11
1.00
1.13
1.23
1.03
2.06
0.99
1.00
8.7
8.6
8.5
8.4
8.3
8.2
8.1
8.0
7.9
7.8
7.7
7.6
7.5
7.4
7.3
7.2
7.1
7.0
6.9
6.8
6.7
f1 (ppm)
2300
2200
2100
2000
1900
1800
1700
1600
1500
1400
1300
1200
1100
1000
900
800
700
600
500
400
300
200
100
0
-100
-200

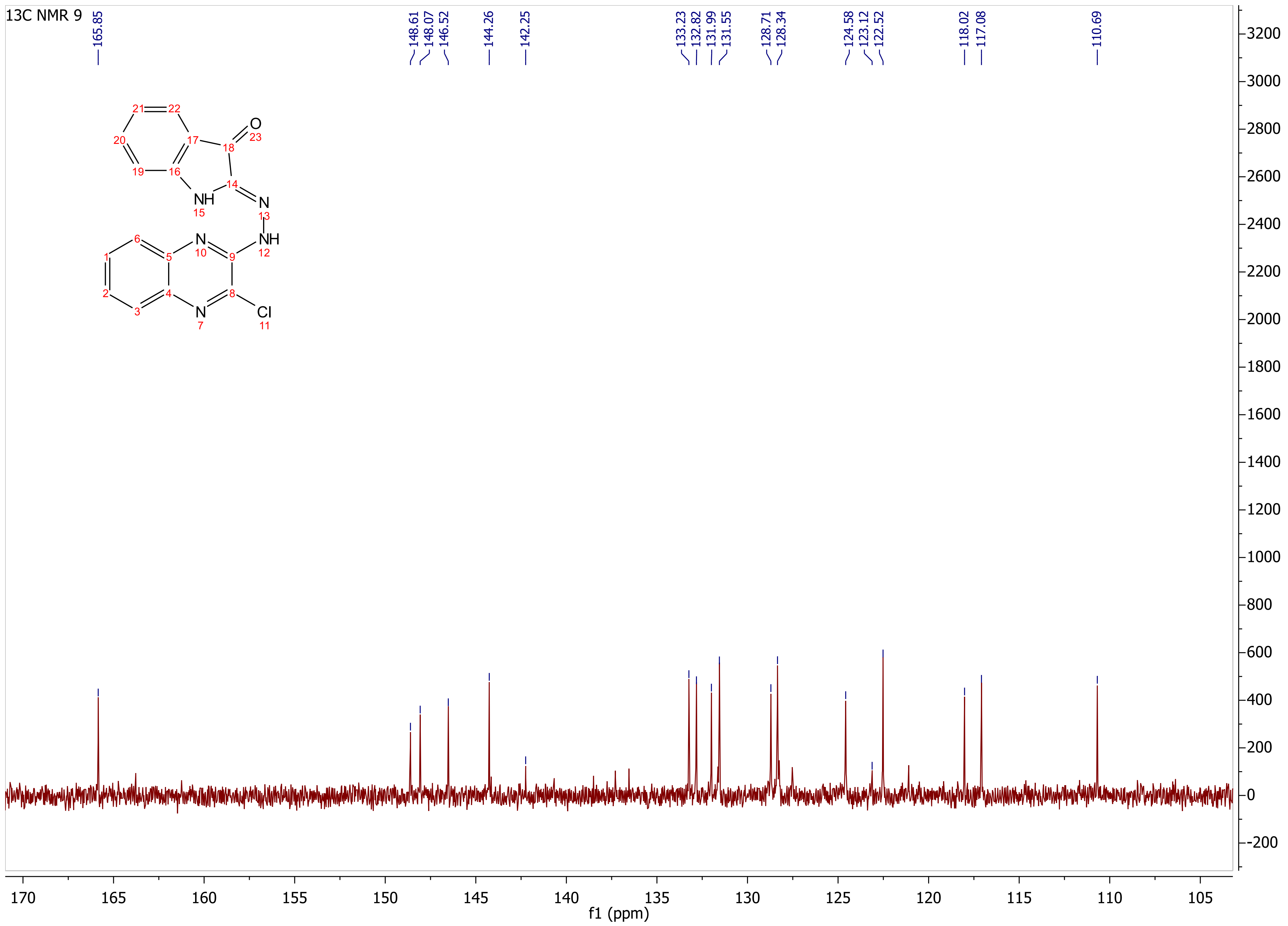

13C NMR 9
165.85
148.61
148.07
146.52
144.26
142.25
133.23
132.82
131.99
131.55
128.71
128.34
124.58
123.12
122.52
118.02
117.08
110.69
f1 (ppm)

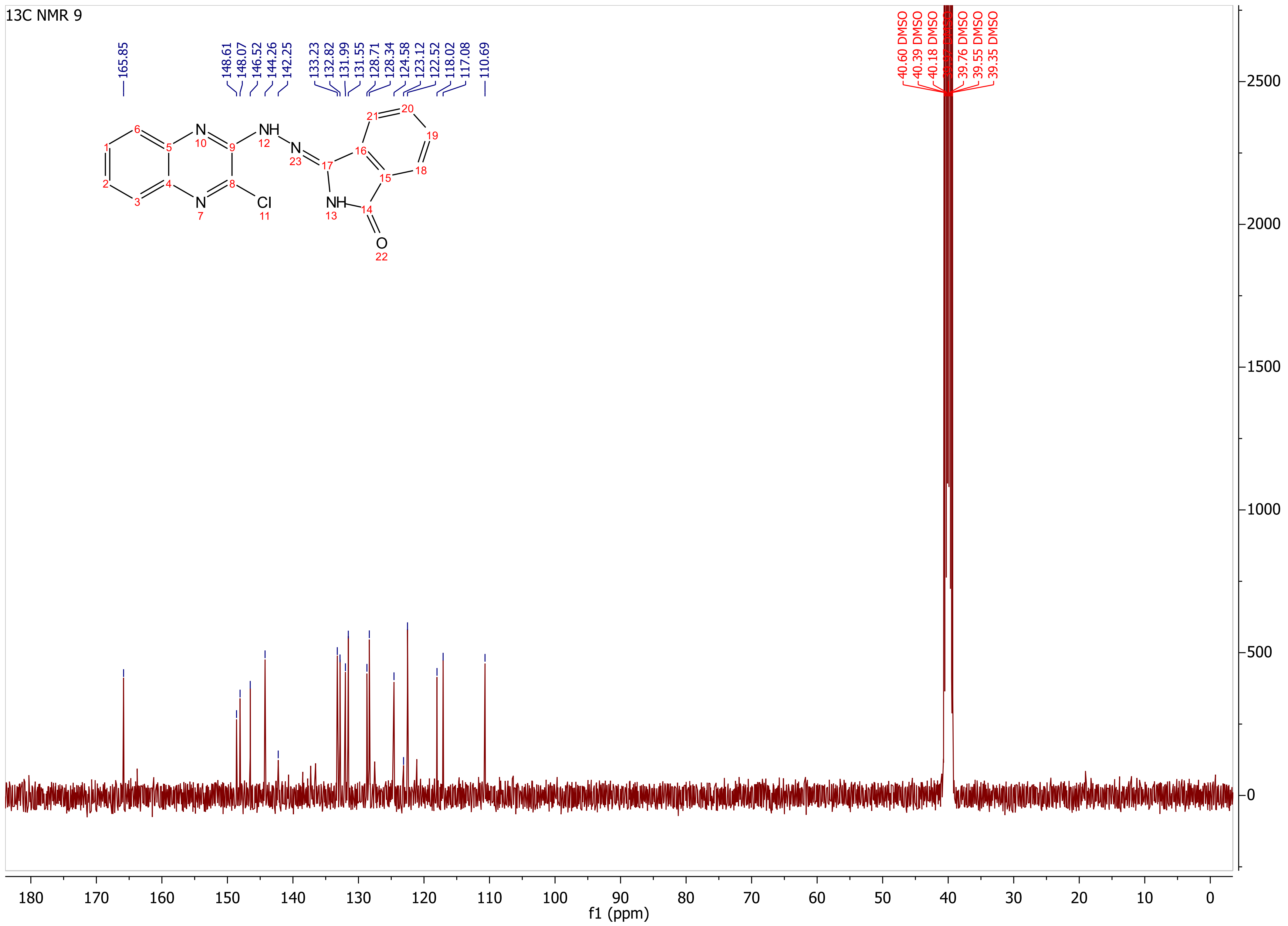
13C NMR 9
165.85
148.61
148.07
146.52
144.26
142.25
133.23
132.82
131.99
131.55
128.71
128.34
124.58
123.12
122.52
118.02
117.08
110.69
40.60 DMSO
40.39 DMSO
40.18 DMSO
39.76 DMSO
39.55 DMSO
39.35 DMSO
NH
N
Cl
O
f1 (ppm)

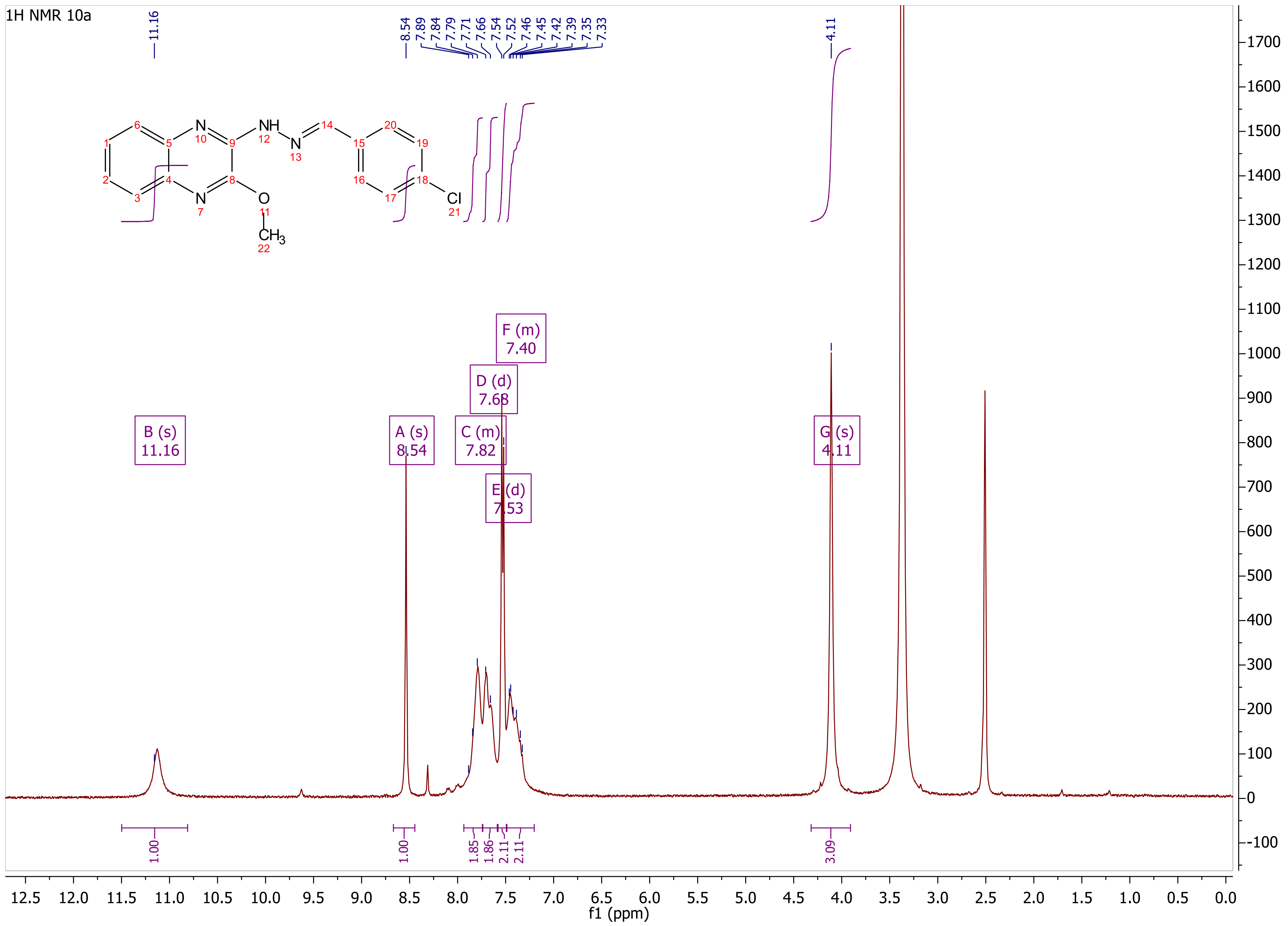

1H NMR 10a
11.16
8.54
7.89
7.84
7.79
7.71
7.66
7.54
7.52
7.46
7.45
7.42
7.39
7.35
7.33
4.11
N
NH
N
Cl
O
CH3
B (s) 11.16
A (s) 8.54
C (m) 7.82
D (d) 7.68
E (d) 7.53
F (m) 7.40
G (s) 4.11
1.00
1.00
1.85
1.86
2.11
2.11
3.09
f1 (ppm)

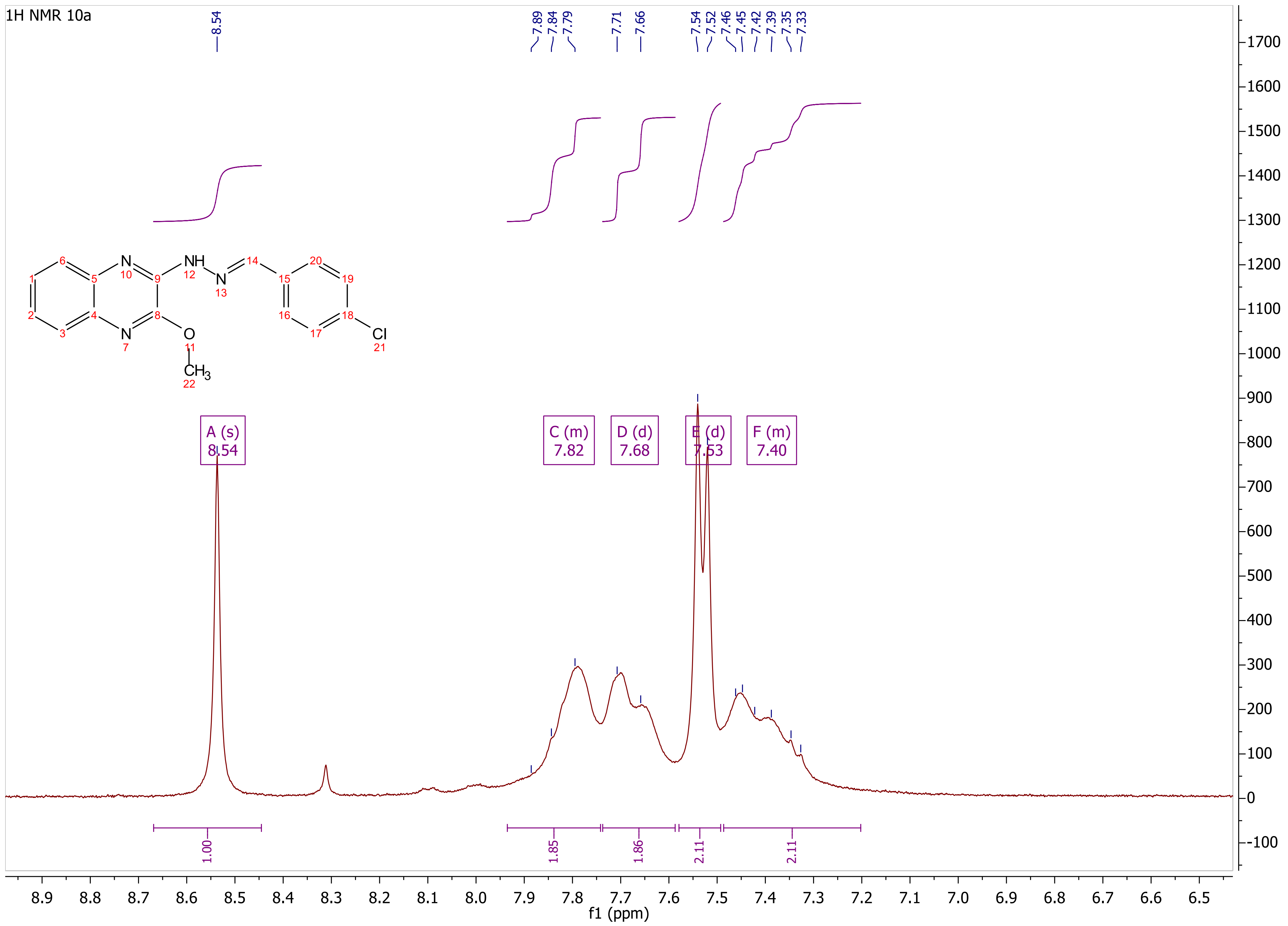
1H NMR 10a
8.54
7.89
7.84
7.79
7.71
7.66
7.54
7.52
7.46
7.45
7.42
7.39
7.35
7.33
A (s) 8.54
C (m) 7.82
D (d) 7.68
E (d) 7.53
F (m) 7.40
1.00
1.85
1.86
2.11
2.11
NH
N
O
CH3
Cl
f1 (ppm)

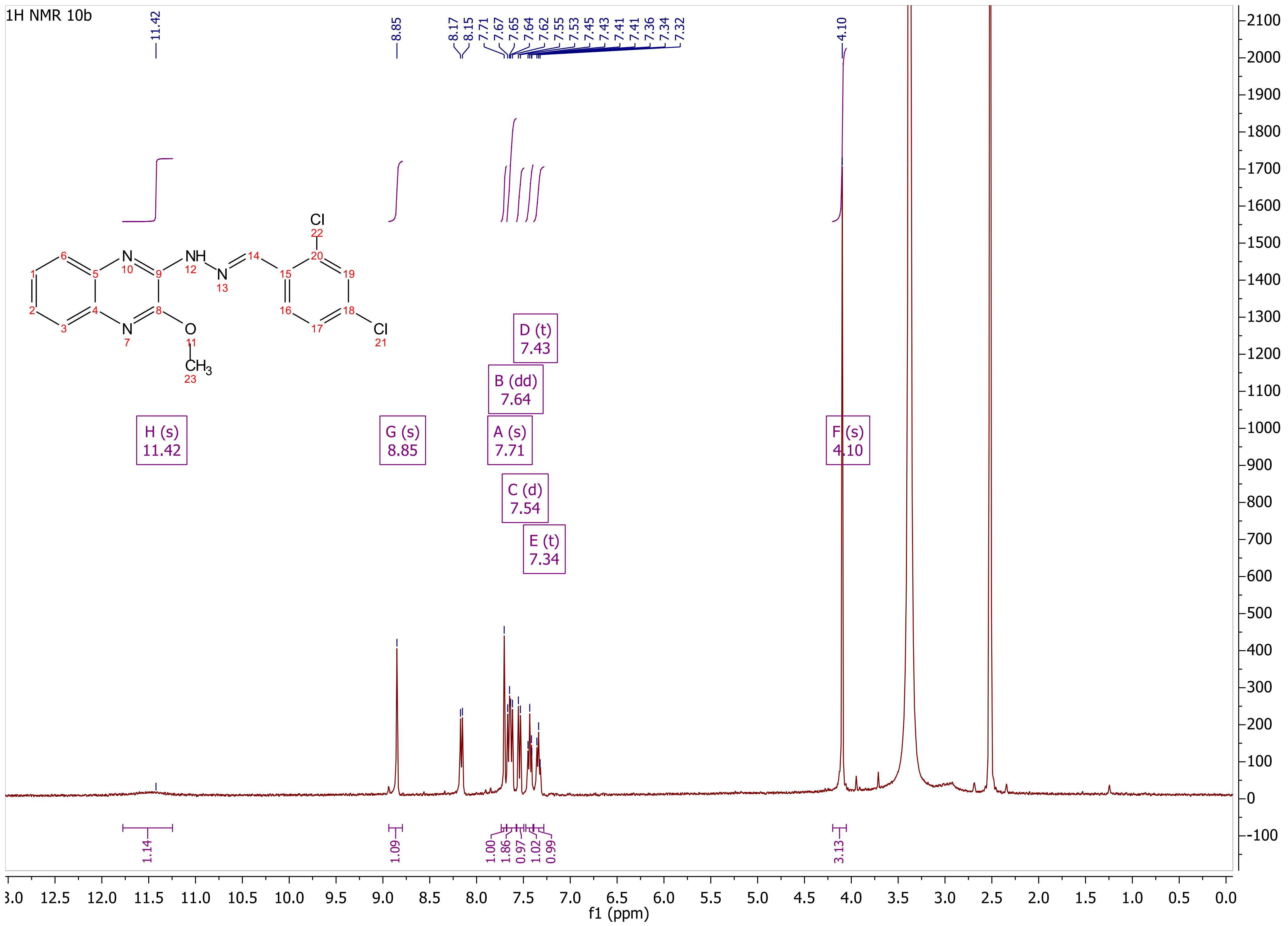
1H NMR 10b
11.42
8.85
8.17
8.15
7.71
7.67
7.65
7.64
7.62
7.55
7.53
7.45
7.43
7.41
7.41
7.36
7.34
7.32
4.10
H (s)
11.42
G (s)
8.85
A (s)
7.71
B (dd)
7.64
C (d)
7.54
D (t)
7.43
E (t)
7.34
F (s)
4.10
1.14
1.09
1.00
1.86
0.97
1.02
0.99
3.13
f1 (ppm)

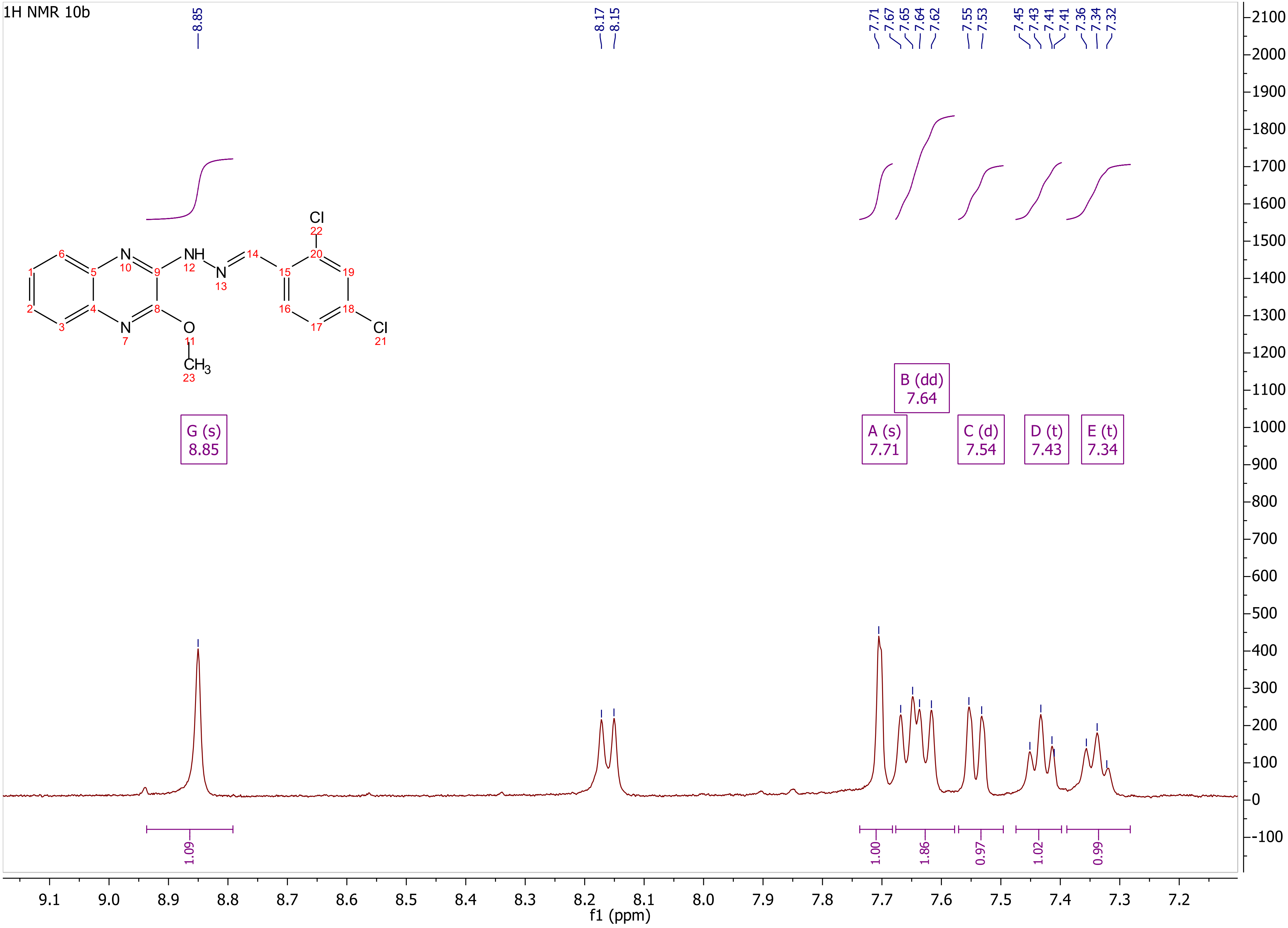
1H NMR 10b
8.85
8.17
8.15
7.71
7.67
7.65
7.64
7.62
7.55
7.53
7.45
7.43
7.41
7.41
7.36
7.34
7.32
G (s) 8.85
A (s) 7.71
B (dd) 7.64
C (d) 7.54
D (t) 7.43
E (t) 7.34
1.09
1.00
1.86
0.97
1.02
0.99
f1 (ppm)

IBRAHIM-HASSAN-10B #111 RT: 1.87 AV: 1 SB: 2 4.45 , 4.45 NL: 8.20E3
T: {0,0} + c EI Full ms [40.00-1000.00]

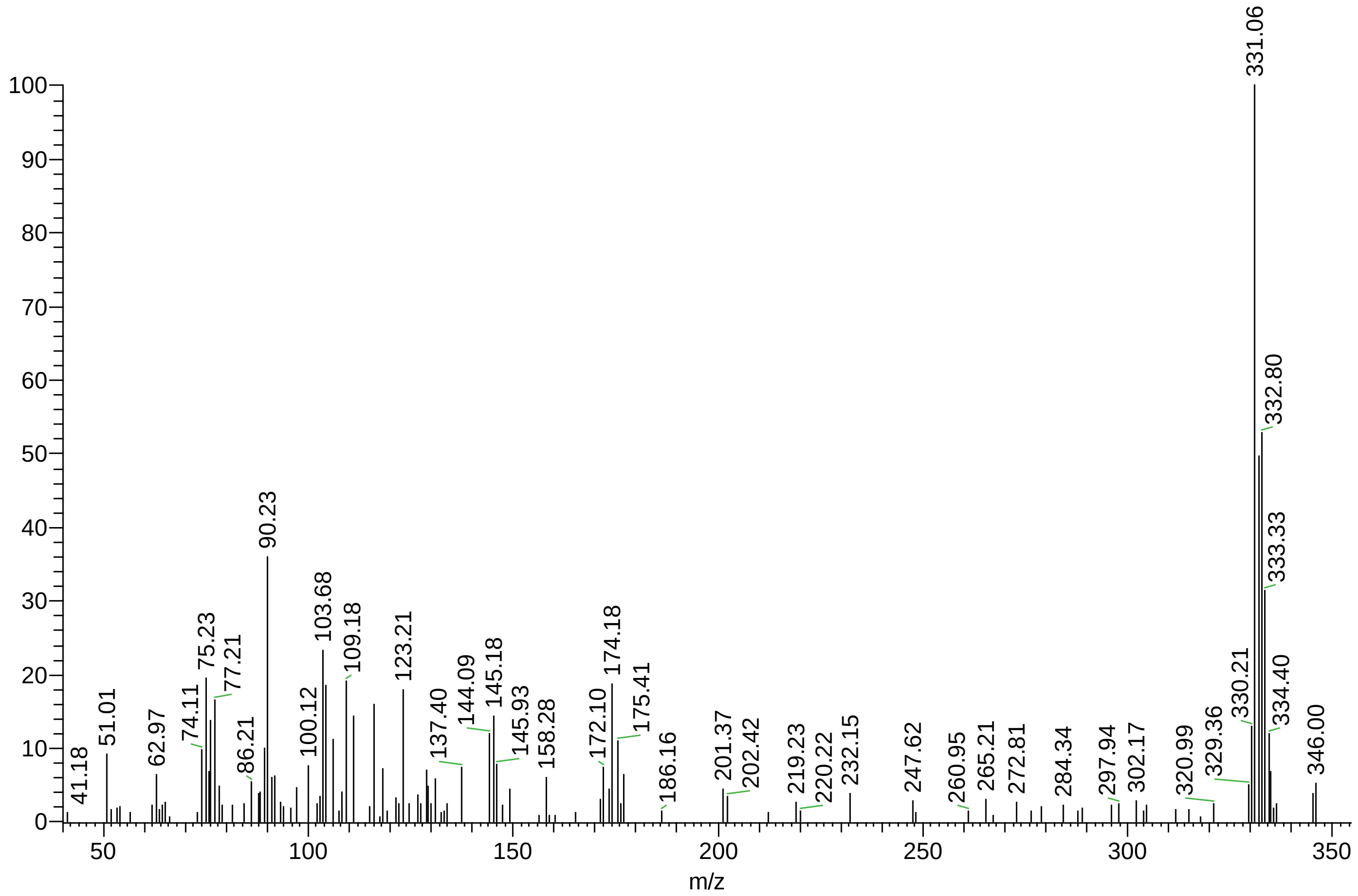

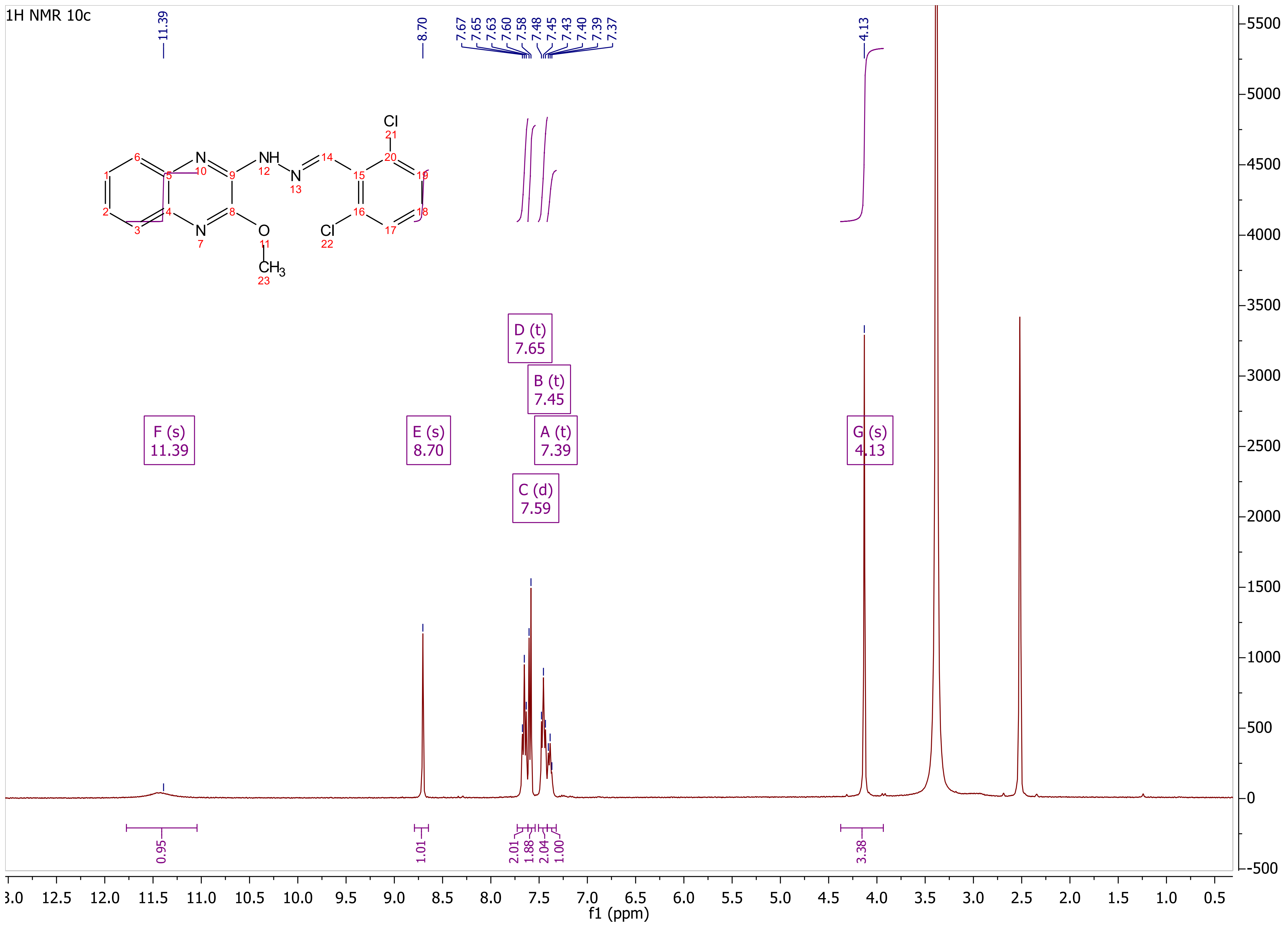
1H NMR 10c
11.39
8.70
7.67 7.65 7.63 7.60 7.58 7.48 7.45 7.43 7.40 7.39 7.37
4.13
Cl
21
20
19
18
17
16
15
14
13
12
NH
N
N
N
O
11
CH3
23
Cl
22
1 2 3 4 5 6 7 8 9 10
D (t) 7.65
B (t) 7.45
A (t) 7.39
C (d) 7.59
F (s) 11.39
E (s) 8.70
G (s) 4.13
0.95
1.01
2.01 1.88 2.04 1.00
3.38
f1 (ppm)

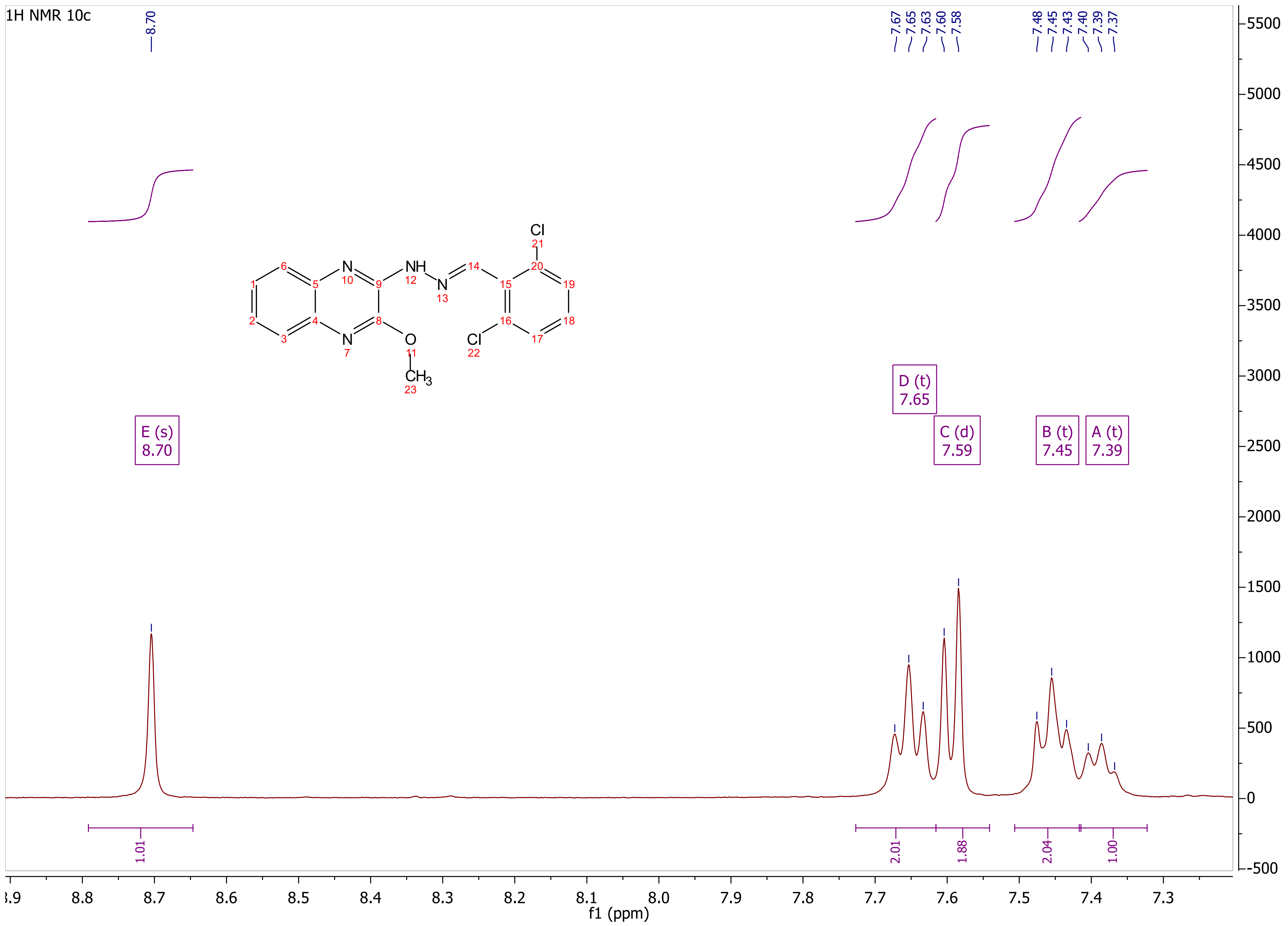

1H NMR 10c
8.70
7.67
7.65
7.63
7.60
7.58
7.48
7.45
7.43
7.40
7.39
7.37
E (s)
8.70
D (t)
7.65
C (d)
7.59
B (t)
7.45
A (t)
7.39
1.01
2.01
1.88
2.04
1.00
f1 (ppm)

IR of compound 10d

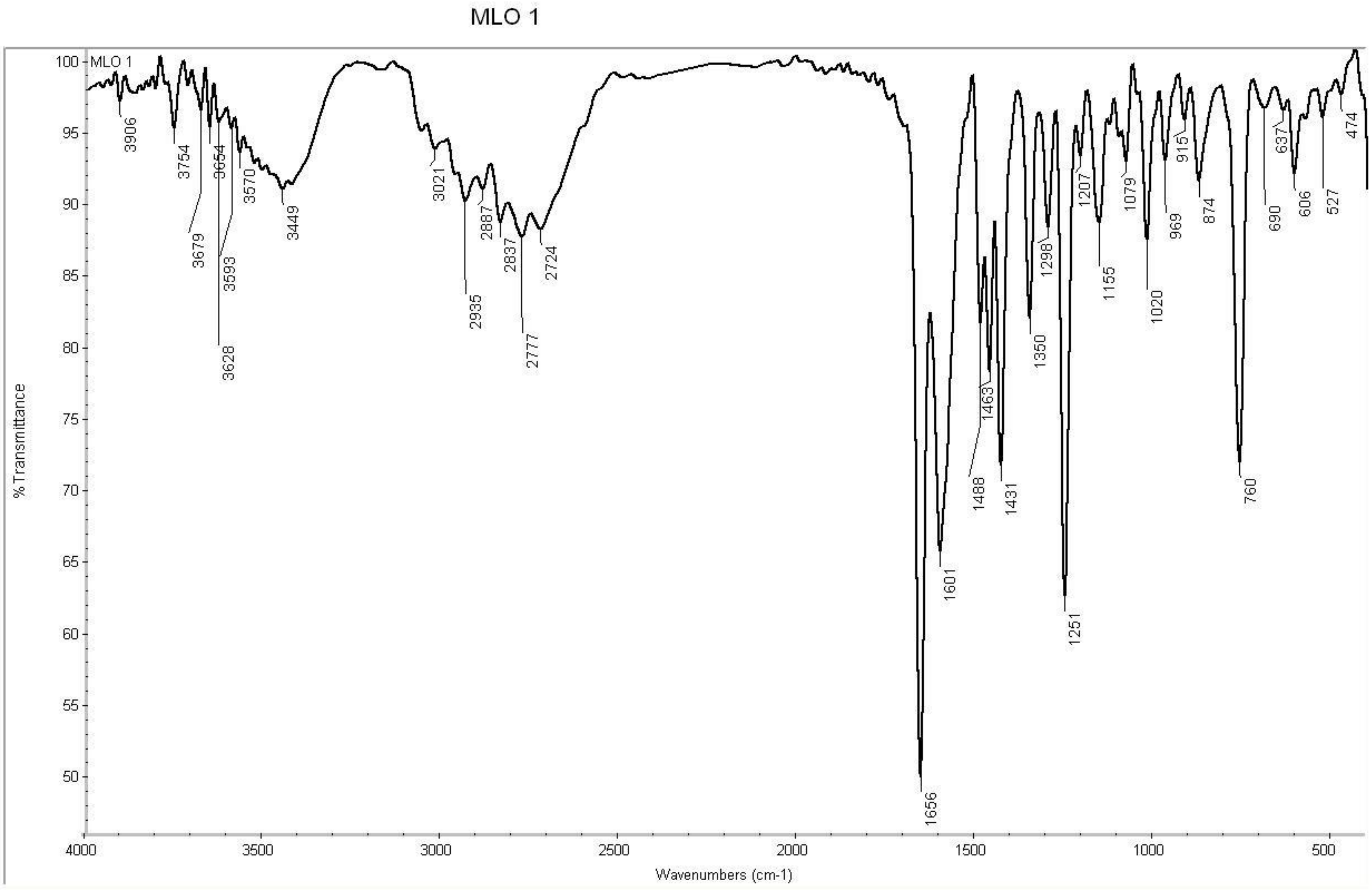

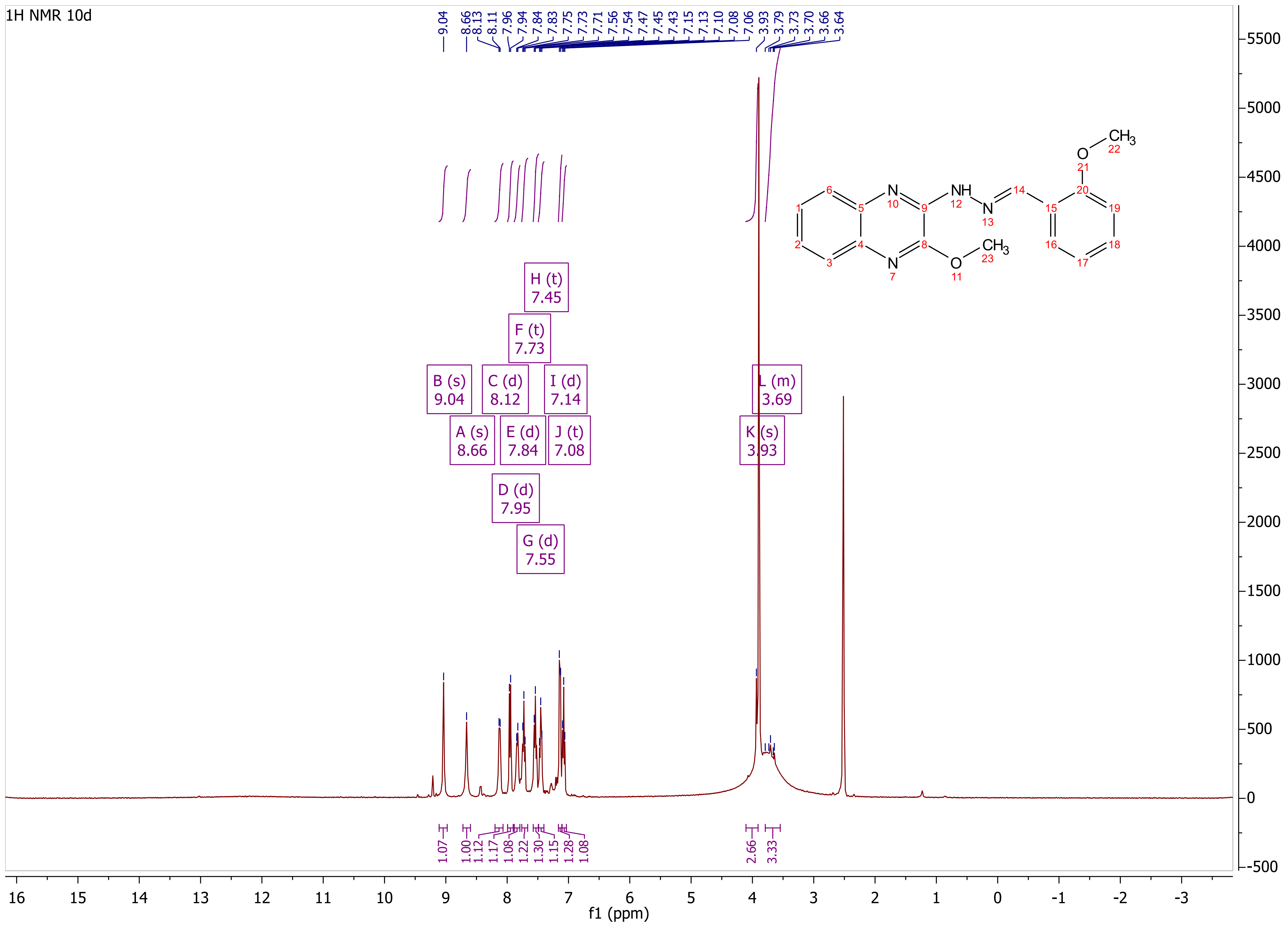
1H NMR 10d
9.04
8.66
8.13
8.11
7.96
7.94
7.84
7.83
7.75
7.73
7.71
7.56
7.54
7.47
7.45
7.43
7.15
7.13
7.10
7.08
7.06
3.93
3.79
3.73
3.70
3.66
3.64
H (t) 7.45
F (t) 7.73
B (s) 9.04
C (d) 8.12
I (d) 7.14
A (s) 8.66
E (d) 7.84
J (t) 7.08
D (d) 7.95
G (d) 7.55
L (m) 3.69
K (s) 3.93
1.07
1.00
1.12
1.17
1.08
1.22
1.30
1.15
1.28
1.08
2.66
3.33
f1 (ppm)

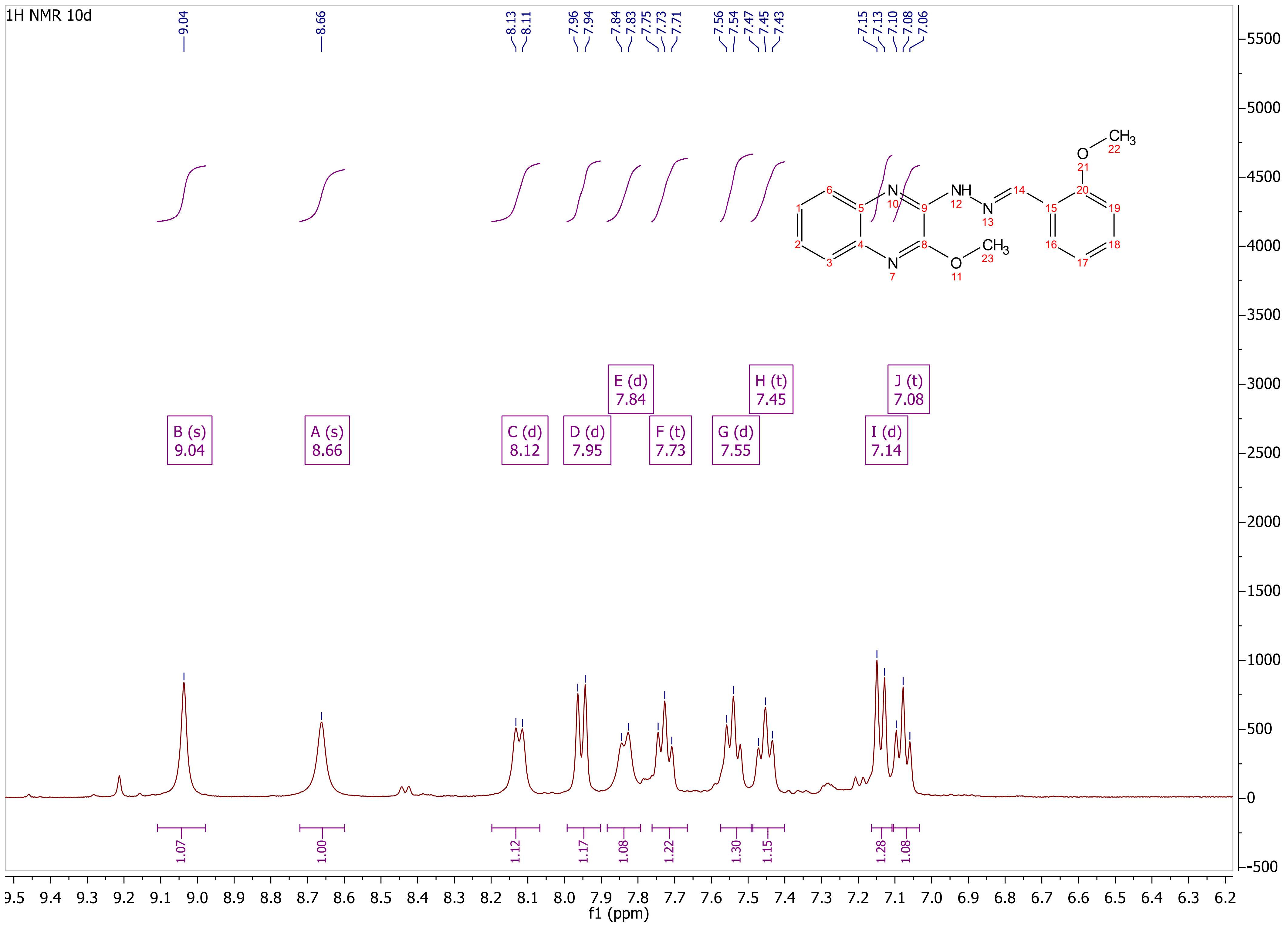
1H NMR 10d
9.04
8.66
8.13
8.11
7.96
7.94
7.84
7.83
7.75
7.73
7.71
7.56
7.54
7.47
7.45
7.43
7.15
7.13
7.10
7.08
7.06
B (s) 9.04
A (s) 8.66
C (d) 8.12
D (d) 7.95
E (d) 7.84
F (t) 7.73
G (d) 7.55
H (t) 7.45
I (d) 7.14
J (t) 7.08
1.07
1.00
1.12
1.17
1.08
1.22
1.30
1.15
1.28
1.08
f1 (ppm)

Ibrahim-hassan-10d #129-133 RT: 2.18-2.24 AV: 5 SB: 2 4.45 , 4.45 NL: 1.03E2
T: {0,0} + c EI Full ms [40.00-1000.00]

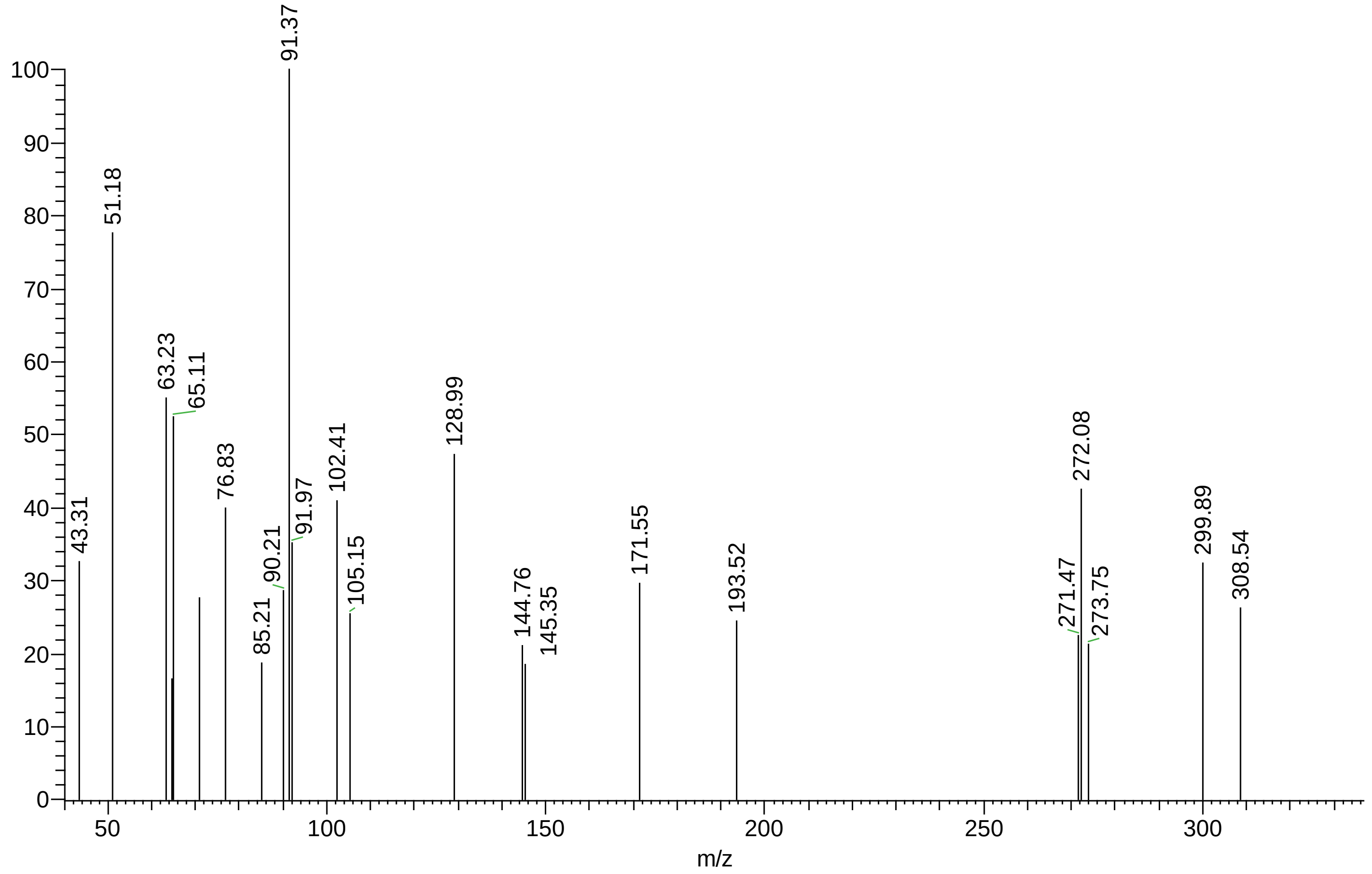

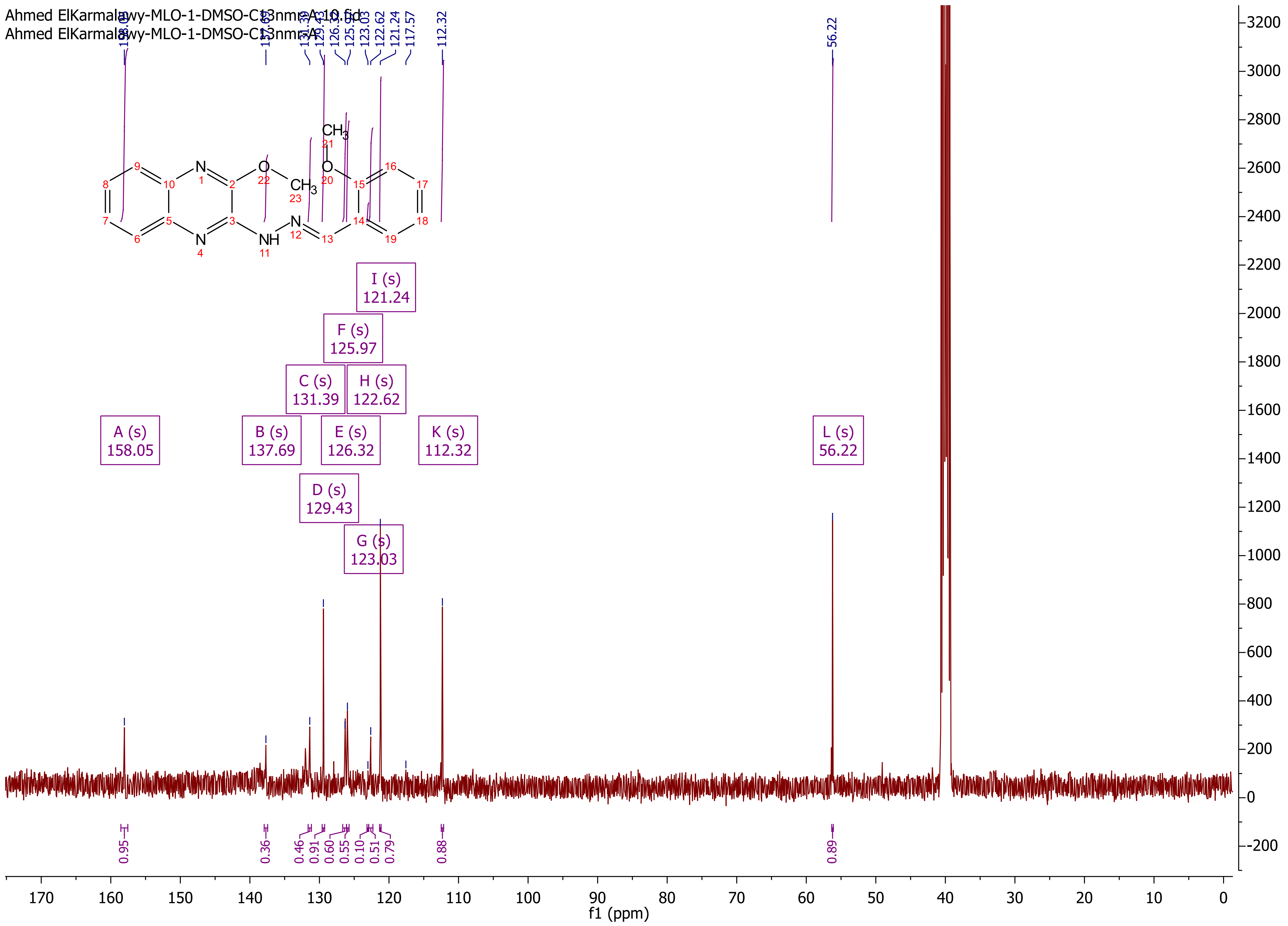
Ahmed ElKarmalawy-MLO-1-DMSO-C13nmr-A10.fid
Ahmed ElKarmalawy-MLO-1-DMSO-C13nmr-A
56.22
122.62
121.24
117.57
112.32
A (s) 158.05
B (s) 137.69
C (s) 131.39
D (s) 129.43
E (s) 126.32
F (s) 125.97
G (s) 123.03
H (s) 122.62
I (s) 121.24
K (s) 112.32
L (s) 56.22
0.95
0.36
0.46
0.91
0.60
0.55
0.10
0.51
0.79
0.88
0.89
f1 (ppm)

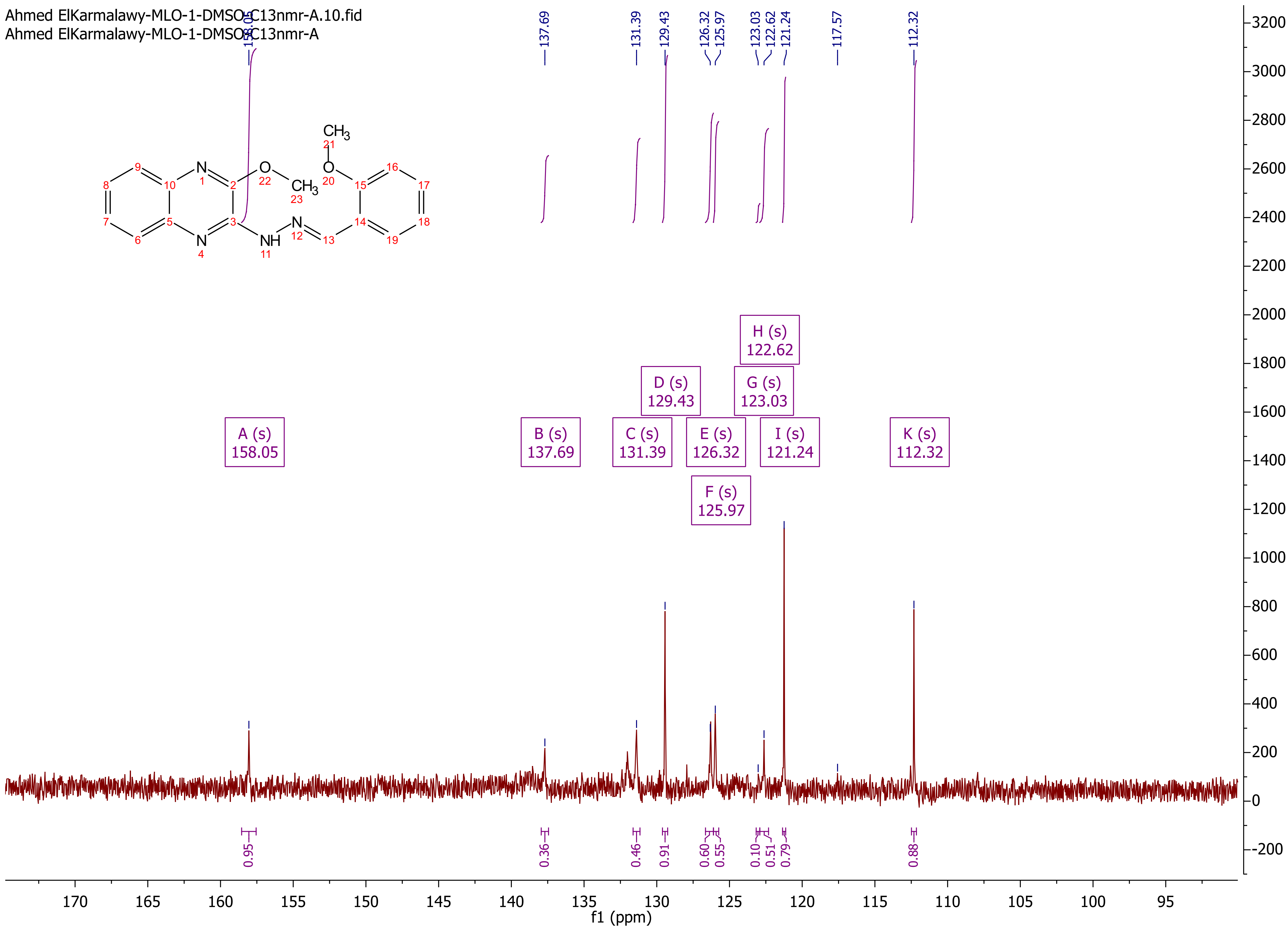

Ahmed ElKarmalawy-MLO-1-DMSO-C13nmr-A.10.fid
Ahmed ElKarmalawy-MLO-1-DMSO-C13nmr-A
158.05
137.69
131.39
129.43
126.32
125.97
123.03
122.62
121.24
117.57
112.32
A (s) 158.05
B (s) 137.69
C (s) 131.39
D (s) 129.43
E (s) 126.32
F (s) 125.97
G (s) 123.03
H (s) 122.62
I (s) 121.24
K (s) 112.32
0.95
0.36
0.46
0.91
0.60
0.55
0.10
0.51
0.79
0.88
f1 (ppm)

Ahmed ElKarmalawy-MLO-1-DMSO-C13nmr-A.10.fid
Ahmed ElKarmalawy-MLO-1-DMSO-C13nmr-A

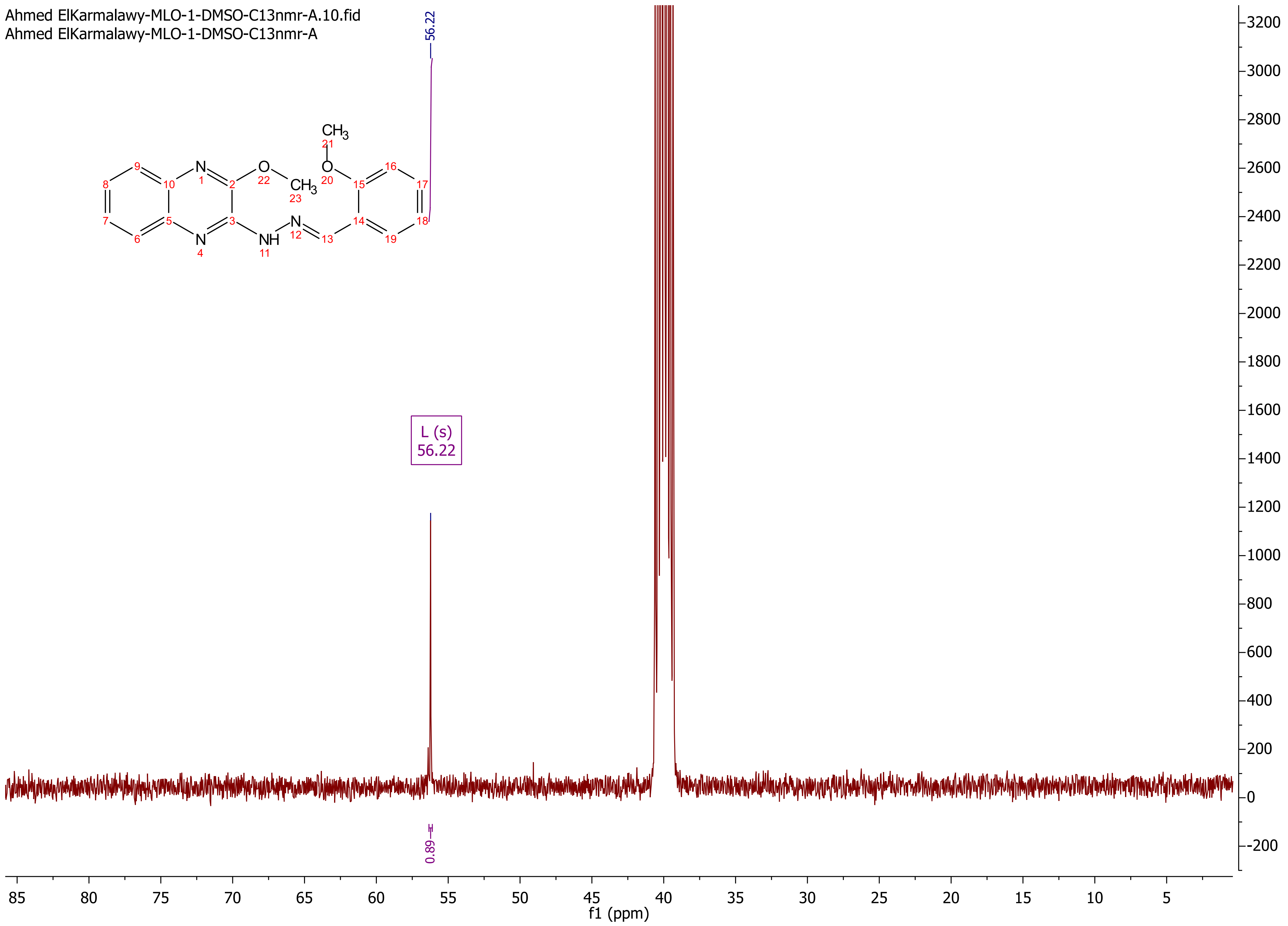

IR of compound 10e

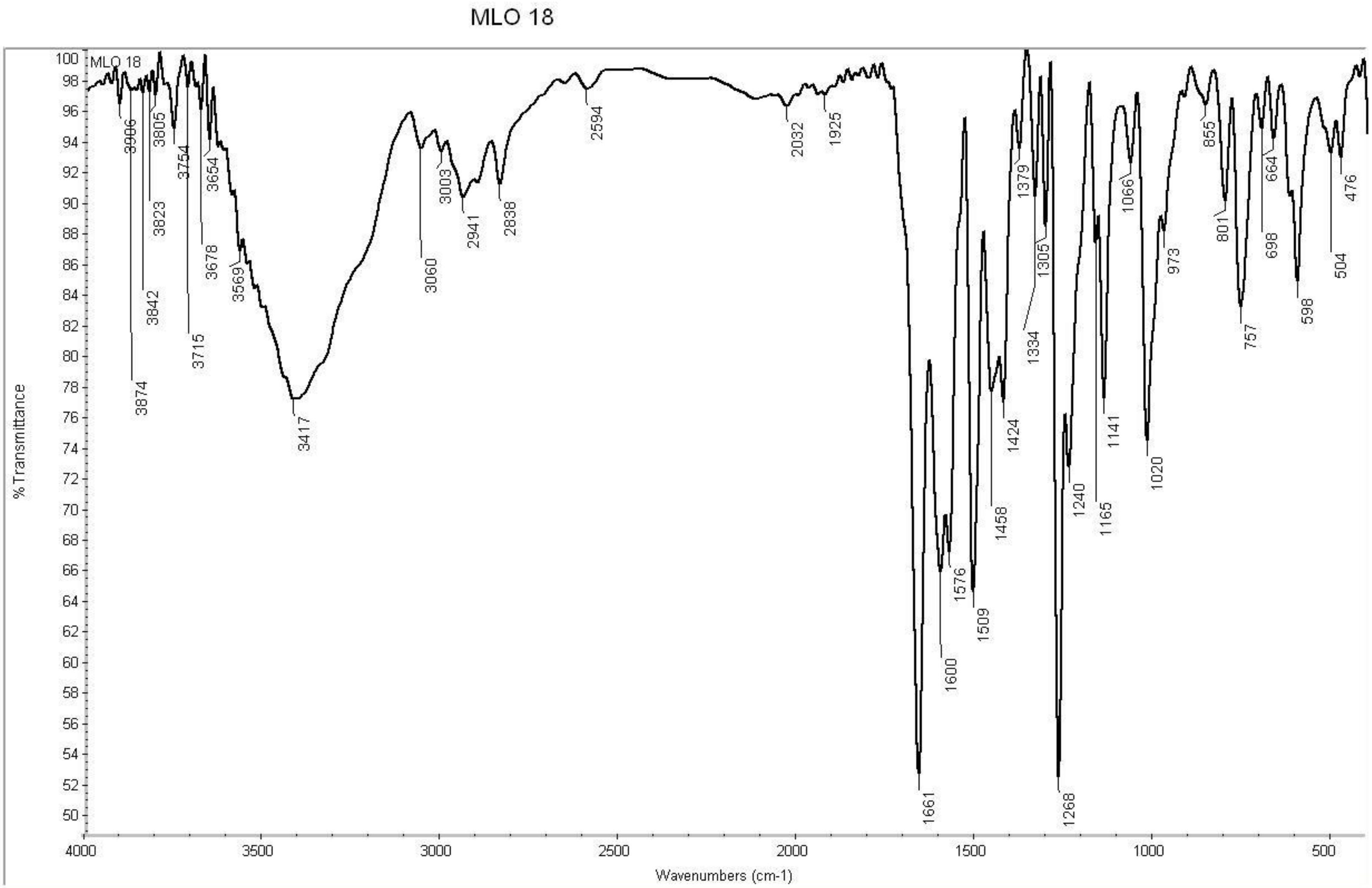

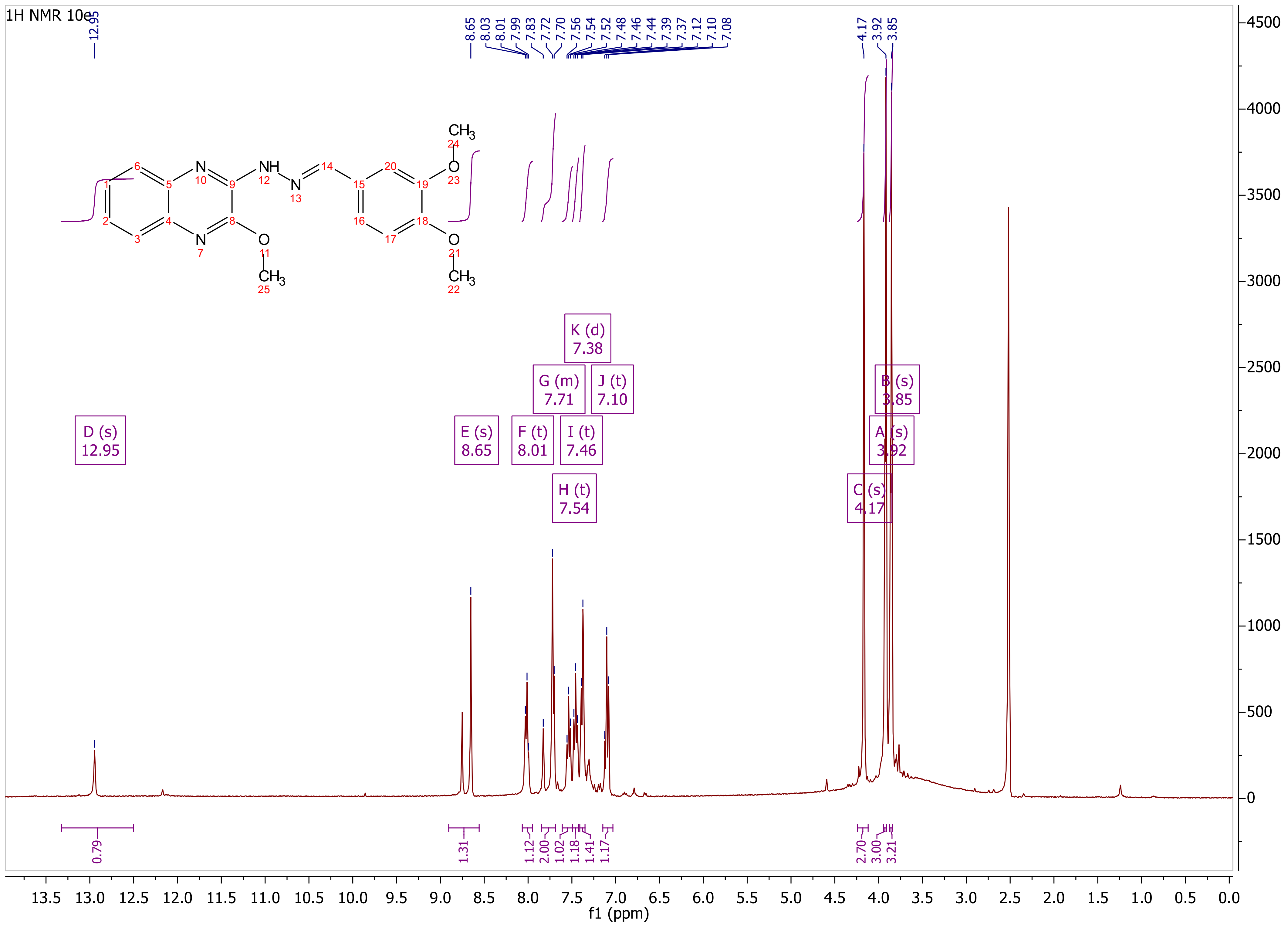
1H NMR 10e
D (s) 12.95
E (s) 8.65
F (t) 8.01
G (m) 7.71
H (t) 7.54
I (t) 7.46
K (d) 7.38
J (t) 7.10
C (s) 4.17
A (s) 3.92
B (s) 3.85
f1 (ppm)

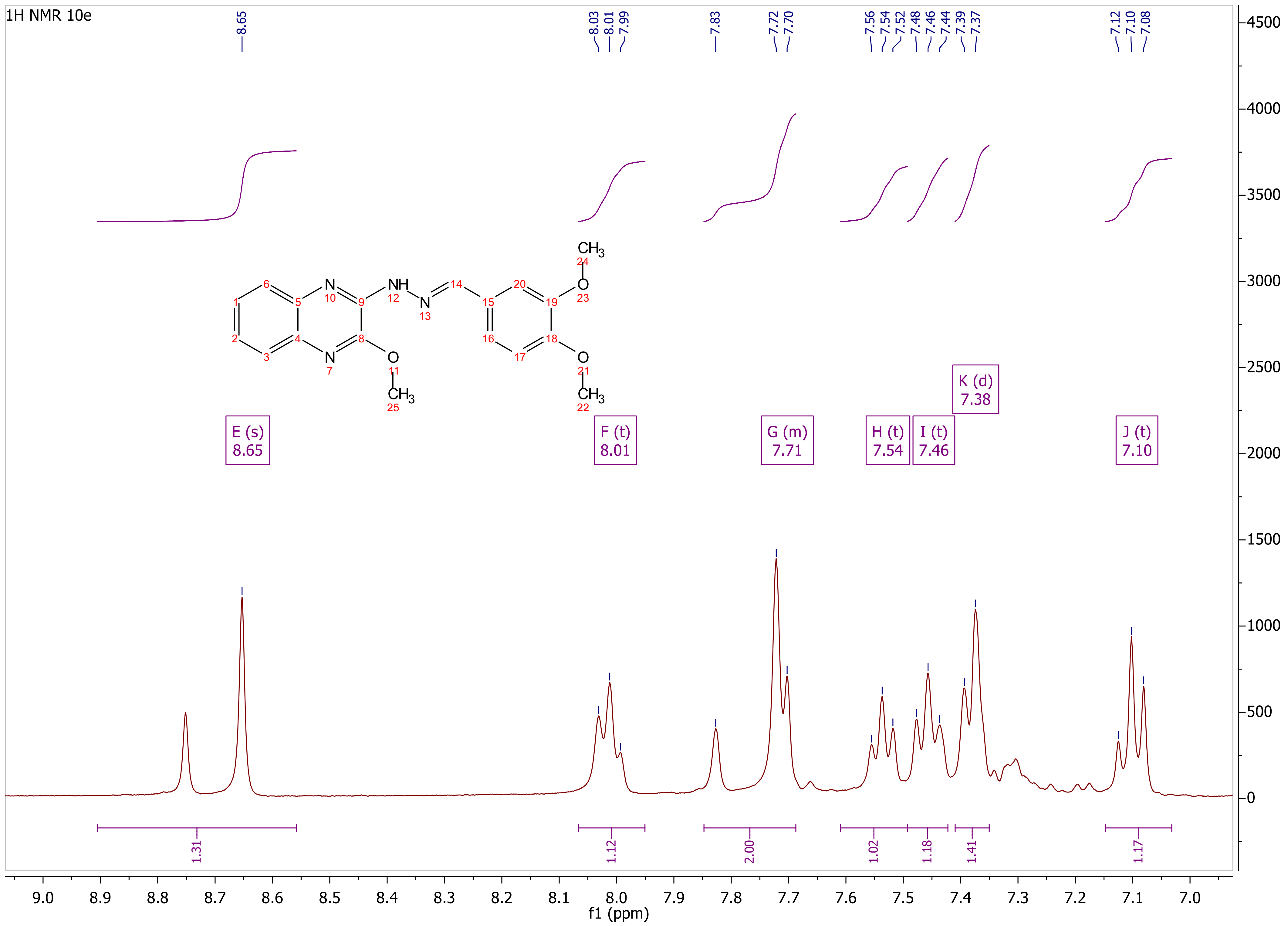
1H NMR 10e
8.65
8.03
8.01
7.99
7.83
7.72
7.70
7.56
7.54
7.52
7.48
7.46
7.44
7.39
7.37
7.12
7.10
7.08
E (s) 8.65
F (t) 8.01
G (m) 7.71
H (t) 7.54
I (t) 7.46
K (d) 7.38
J (t) 7.10
1.31
1.12
2.00
1.02
1.18
1.41
1.17
f1 (ppm)

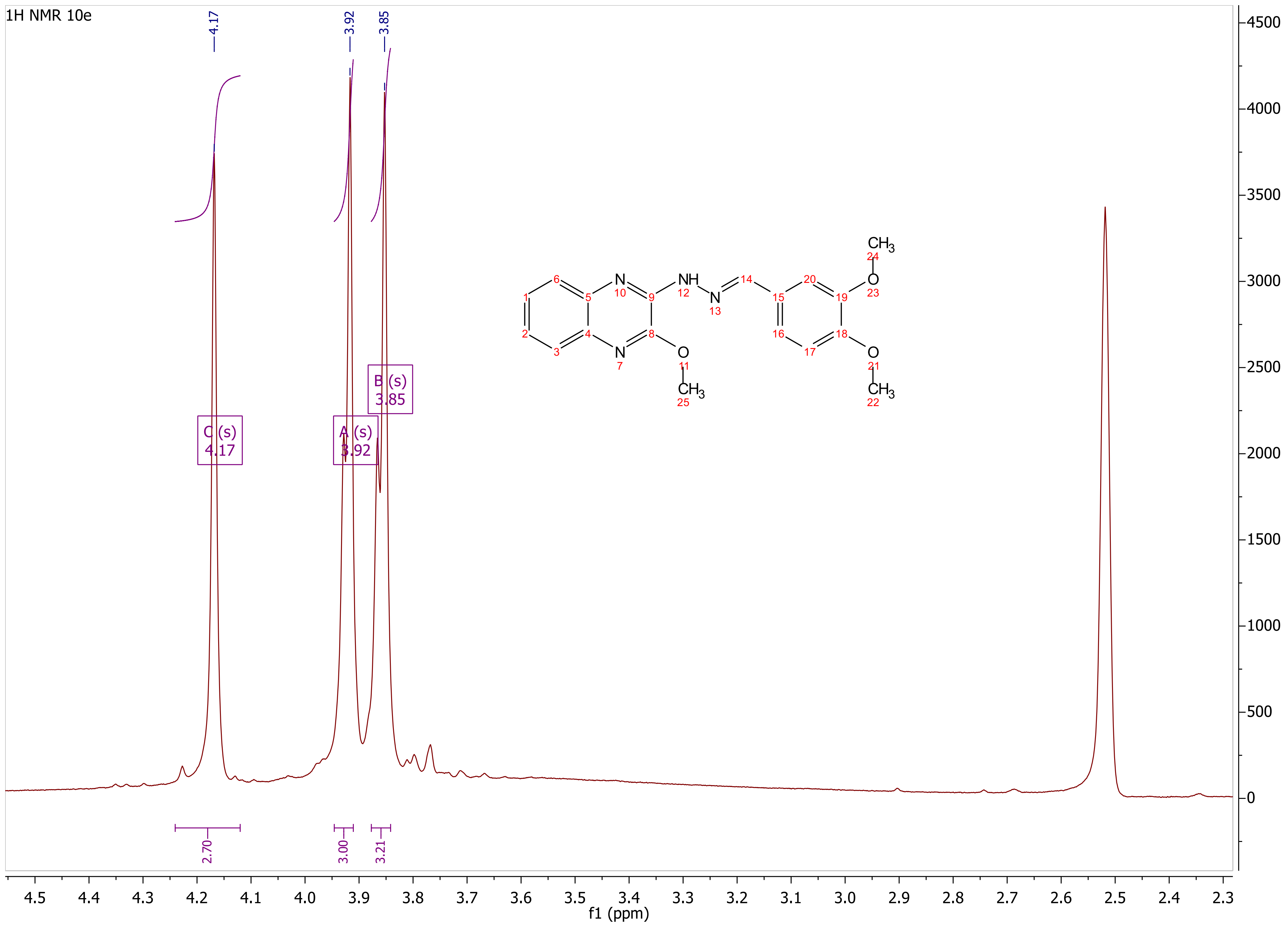

1H NMR 10e
4.17
3.92
3.85
C (s) 4.17
A (s) 3.92
B (s) 3.85
2.70
3.00
3.21
f1 (ppm)

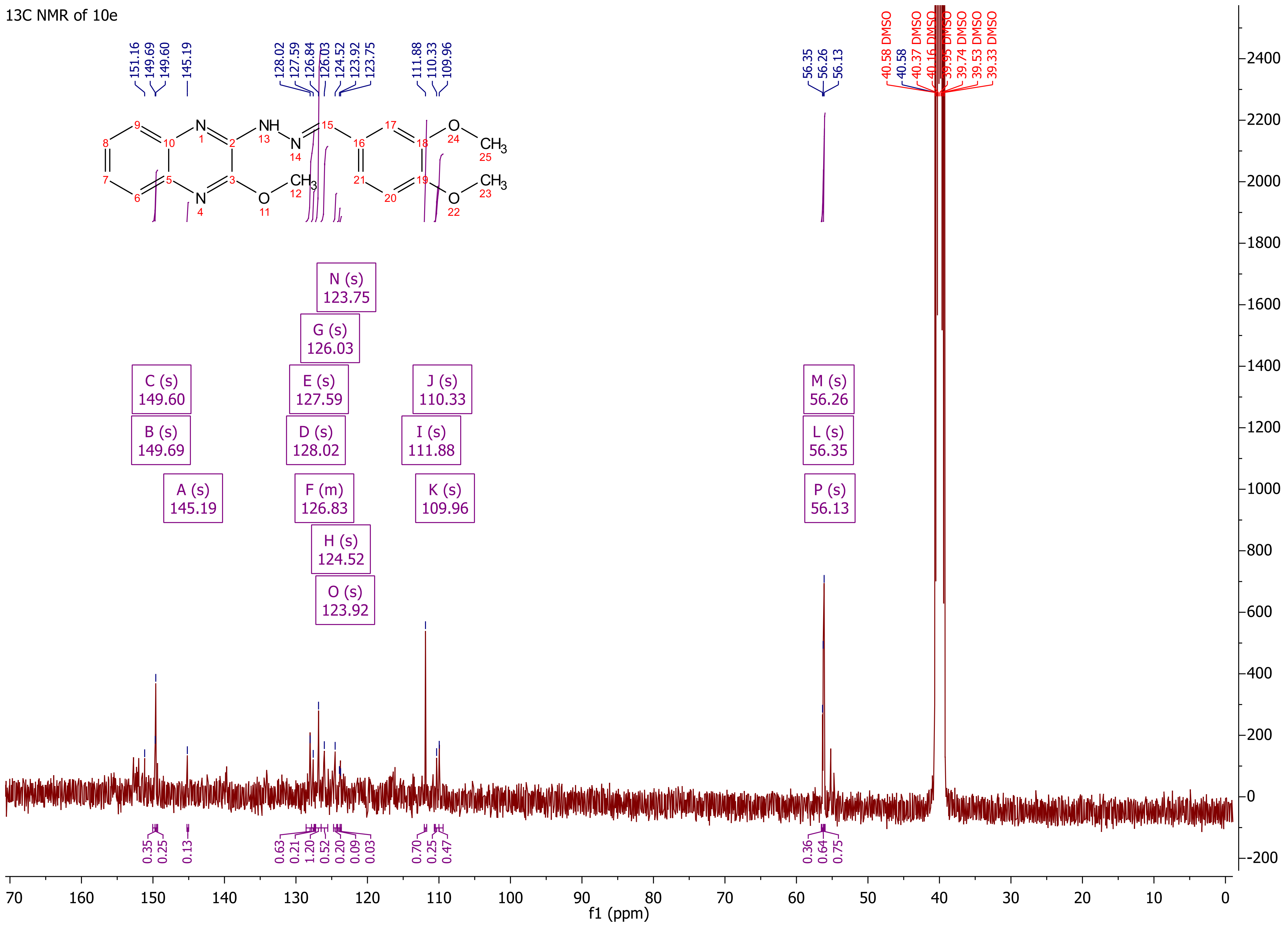
13C NMR of 10e
151.16
149.69
149.60
145.19
128.02
127.59
126.84
126.03
124.52
123.92
123.75
111.88
110.33
109.96
56.35
56.26
56.13
40.58 DMSO
40.58
40.37 DMSO
40.16 DMSO
39.95 DMSO
39.74 DMSO
39.53 DMSO
39.33 DMSO
N (s) 123.75
G (s) 126.03
C (s) 149.60
B (s) 149.69
A (s) 145.19
E (s) 127.59
D (s) 128.02
F (m) 126.83
H (s) 124.52
O (s) 123.92
J (s) 110.33
I (s) 111.88
K (s) 109.96
M (s) 56.26
L (s) 56.35
P (s) 56.13
f1 (ppm)

13C NMR of 10e

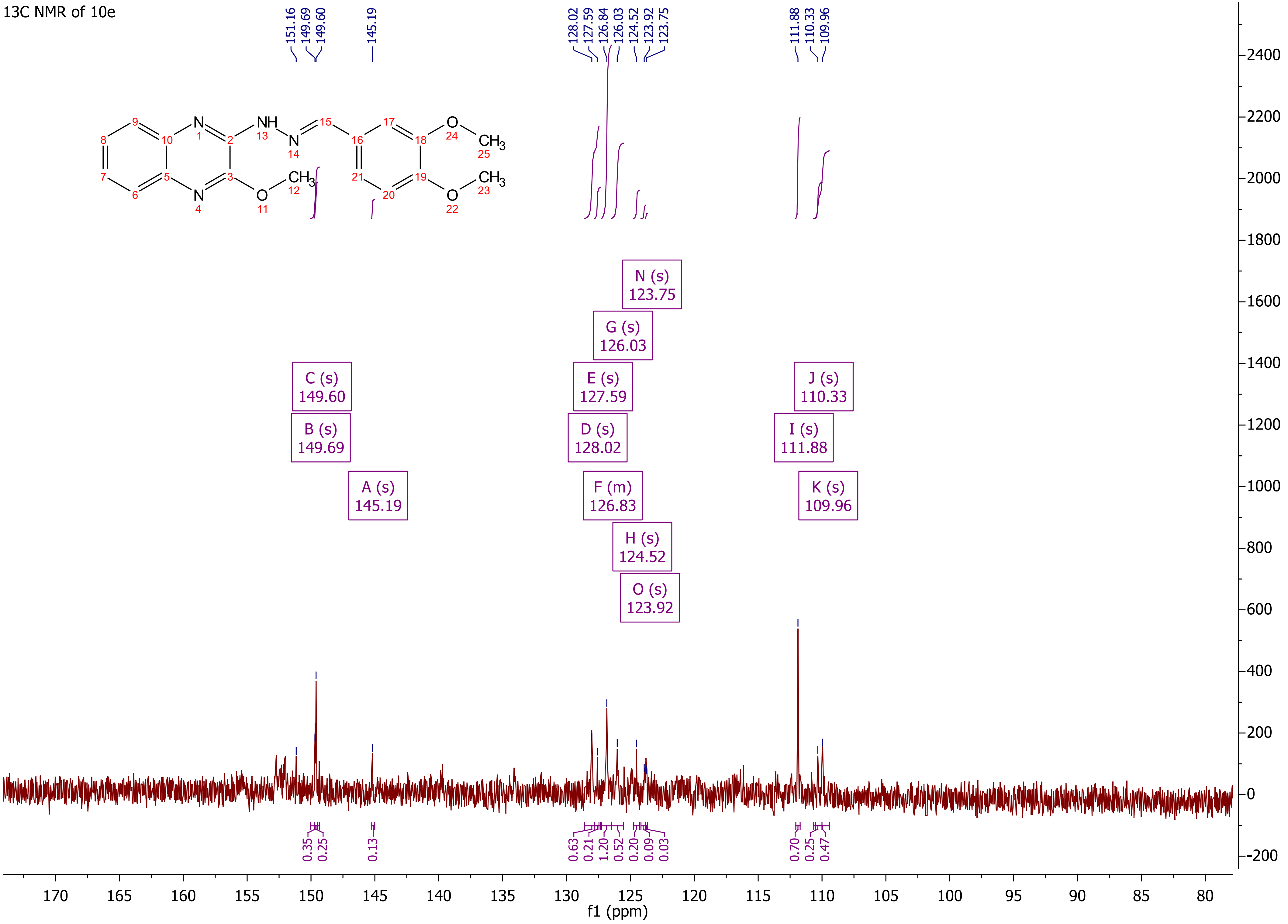

13C NMR of 10e

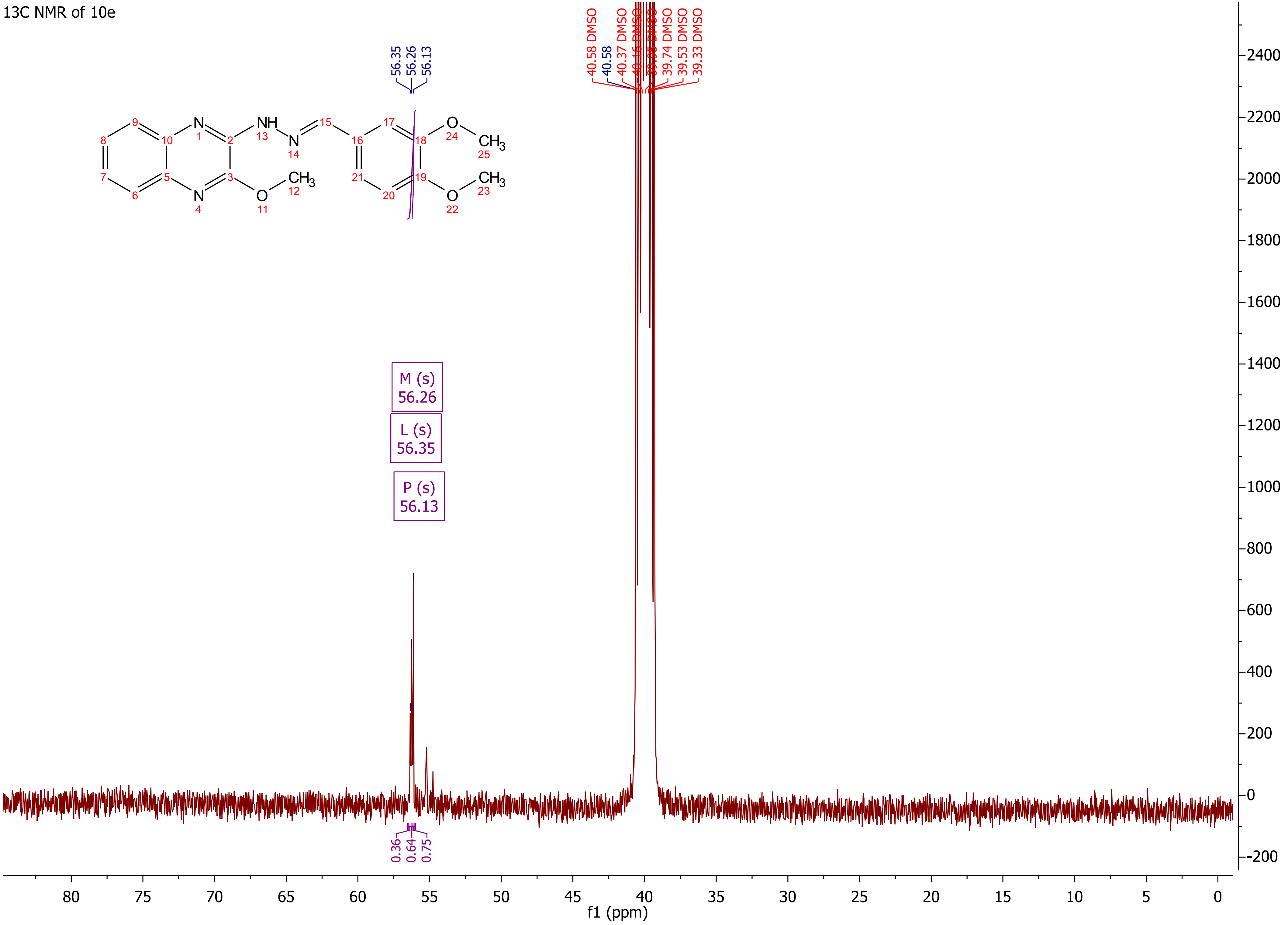

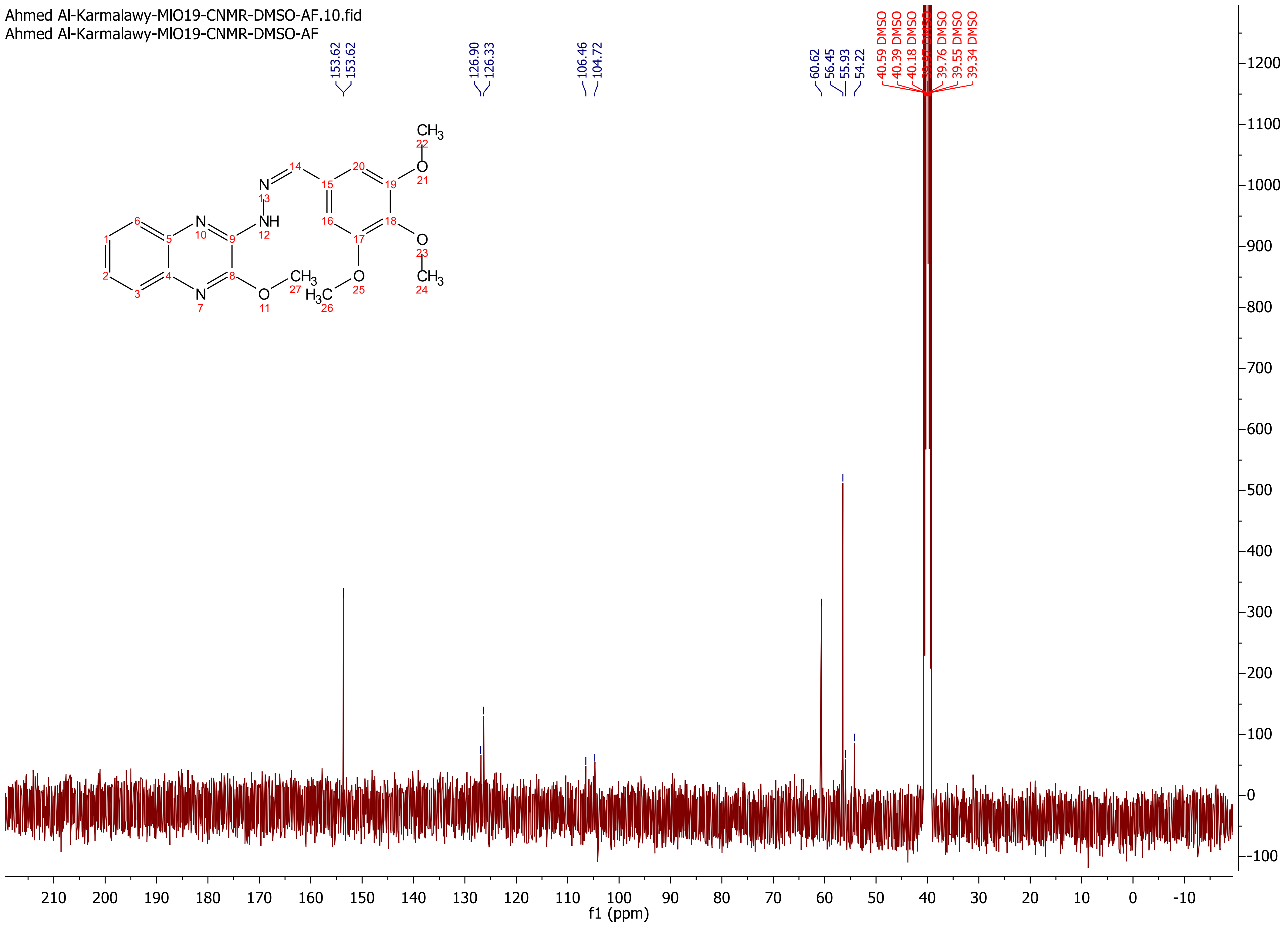

Ahmed Al-Karmalawy-MIO19-CNMR-DMSO-AF.10.fid
Ahmed Al-Karmalawy-MIO19-CNMR-DMSO-AF
153.62
153.62
126.90
126.33
106.46
104.72
60.62
56.45
55.93
54.22
40.59 DMSO
40.39 DMSO
40.18 DMSO
39.76 DMSO
39.55 DMSO
39.34 DMSO
CH3
O
N
NH
N
N
O
CH3
H3C
O
O
CH3
f1 (ppm)

IR of compound 10f

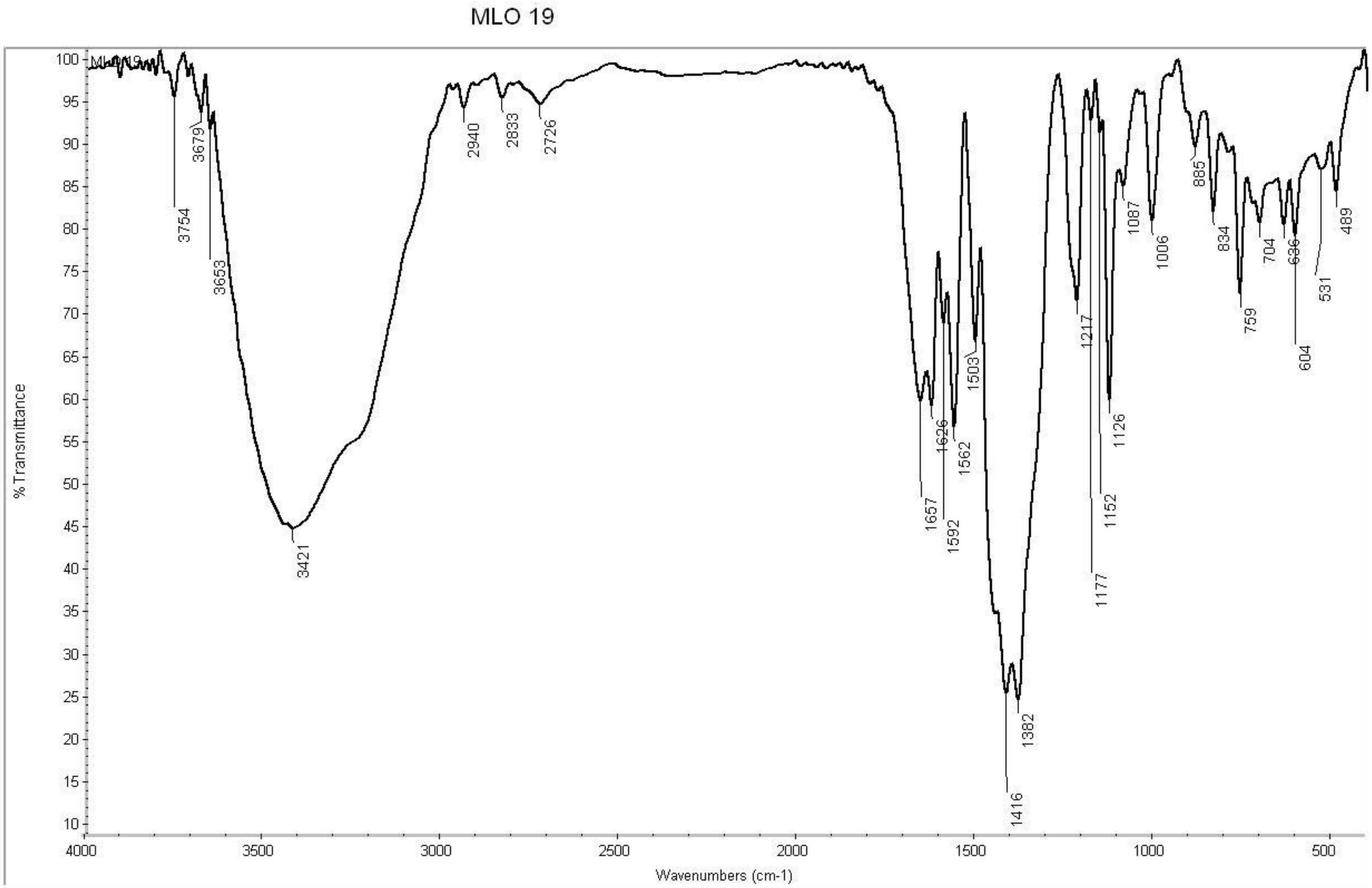

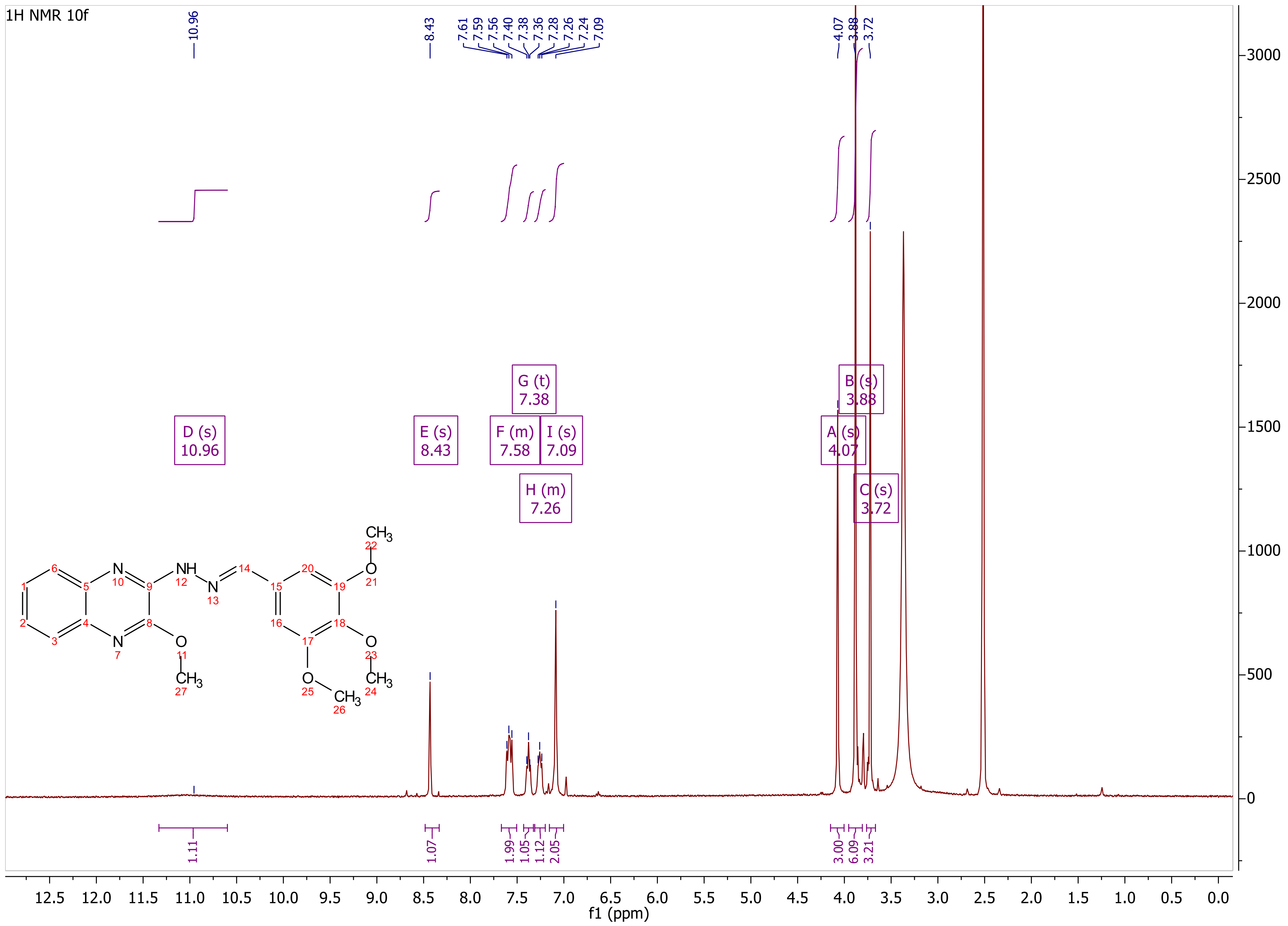
1H NMR 10f
10.96
8.43
7.61 7.59 7.56 7.40 7.38 7.36 7.28 7.26 7.24 7.09
4.07 3.88 3.72
D (s) 10.96
E (s) 8.43
F (m) 7.58
G (t) 7.38
H (m) 7.26
I (s) 7.09
A (s) 4.07
B (s) 3.88
C (s) 3.72
1.11
1.07
1.99 1.05 1.12 2.05
3.00 6.09 3.21
f1 (ppm)

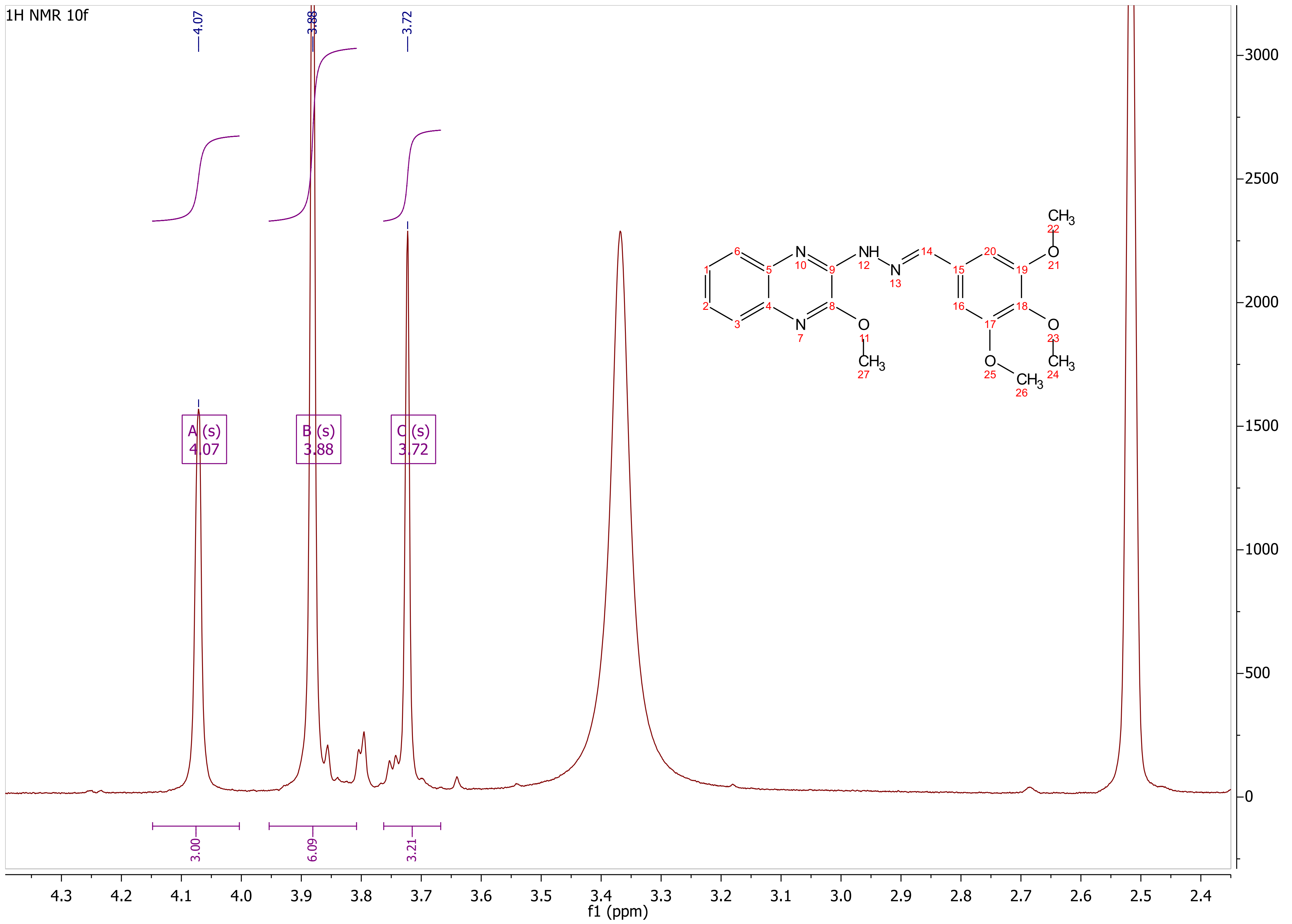
1H NMR 10f
4.07
3.88
3.72
A (s) 4.07
B (s) 3.88
C (s) 3.72
3.00
6.09
3.21
f1 (ppm)

1H NMR 10f

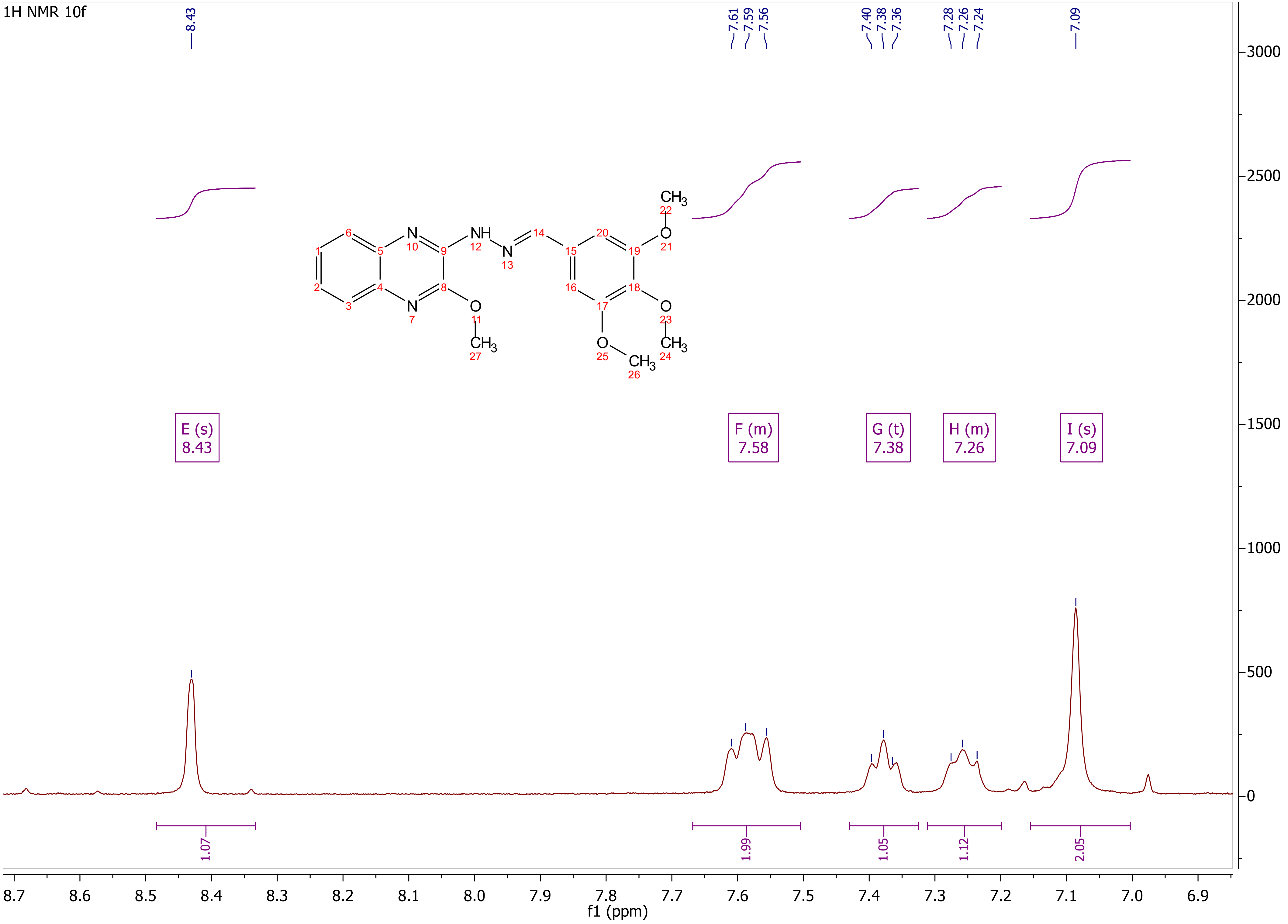
8.43
7.61
7.59
7.56
7.40
7.38
7.36
7.28
7.26
7.24
7.09
E (s) 8.43
F (m) 7.58
G (t) 7.38
H (m) 7.26
I (s) 7.09
1.07
1.99
1.05
1.12
2.05
f1 (ppm)

IR of compound 10g

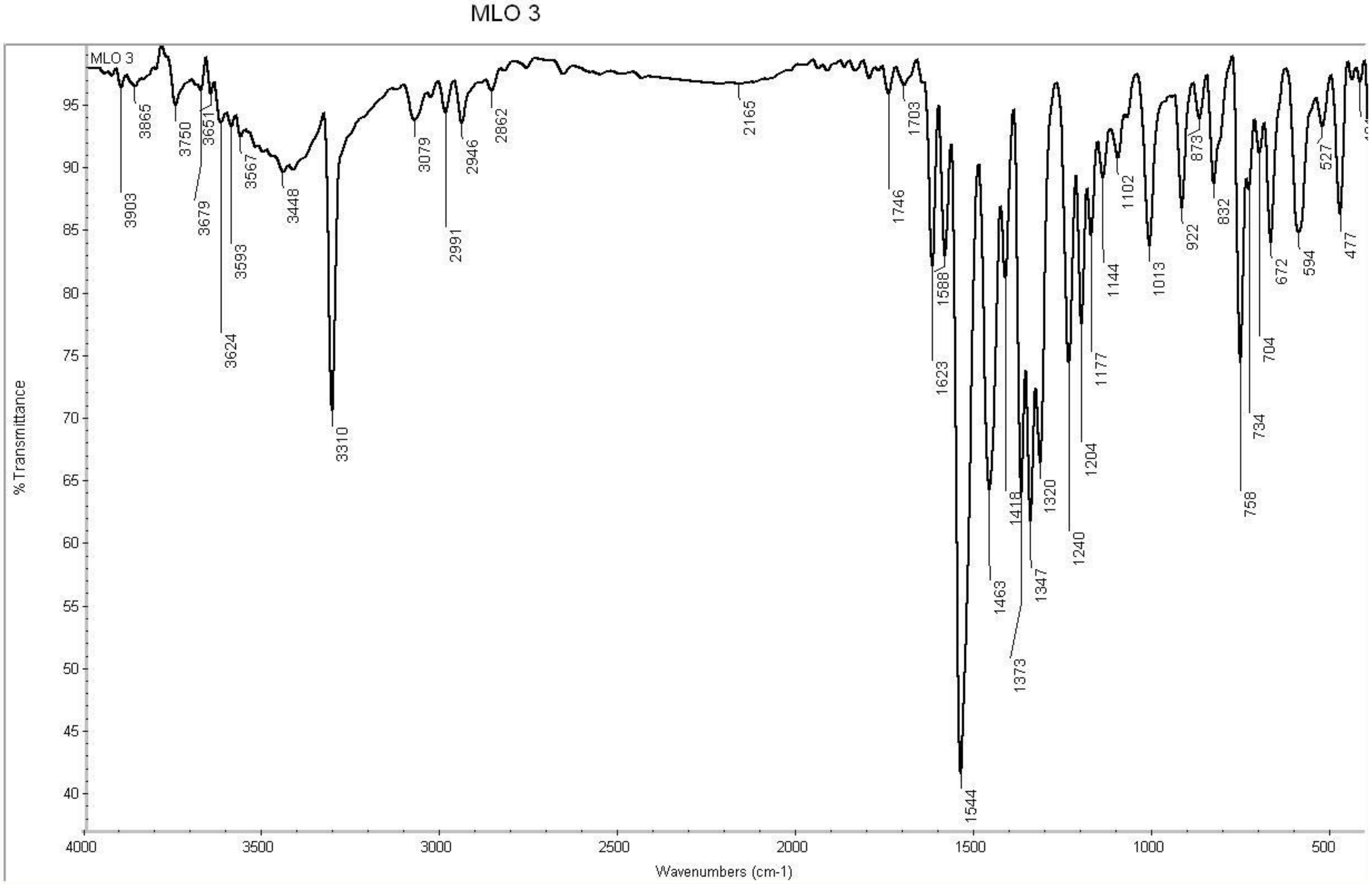

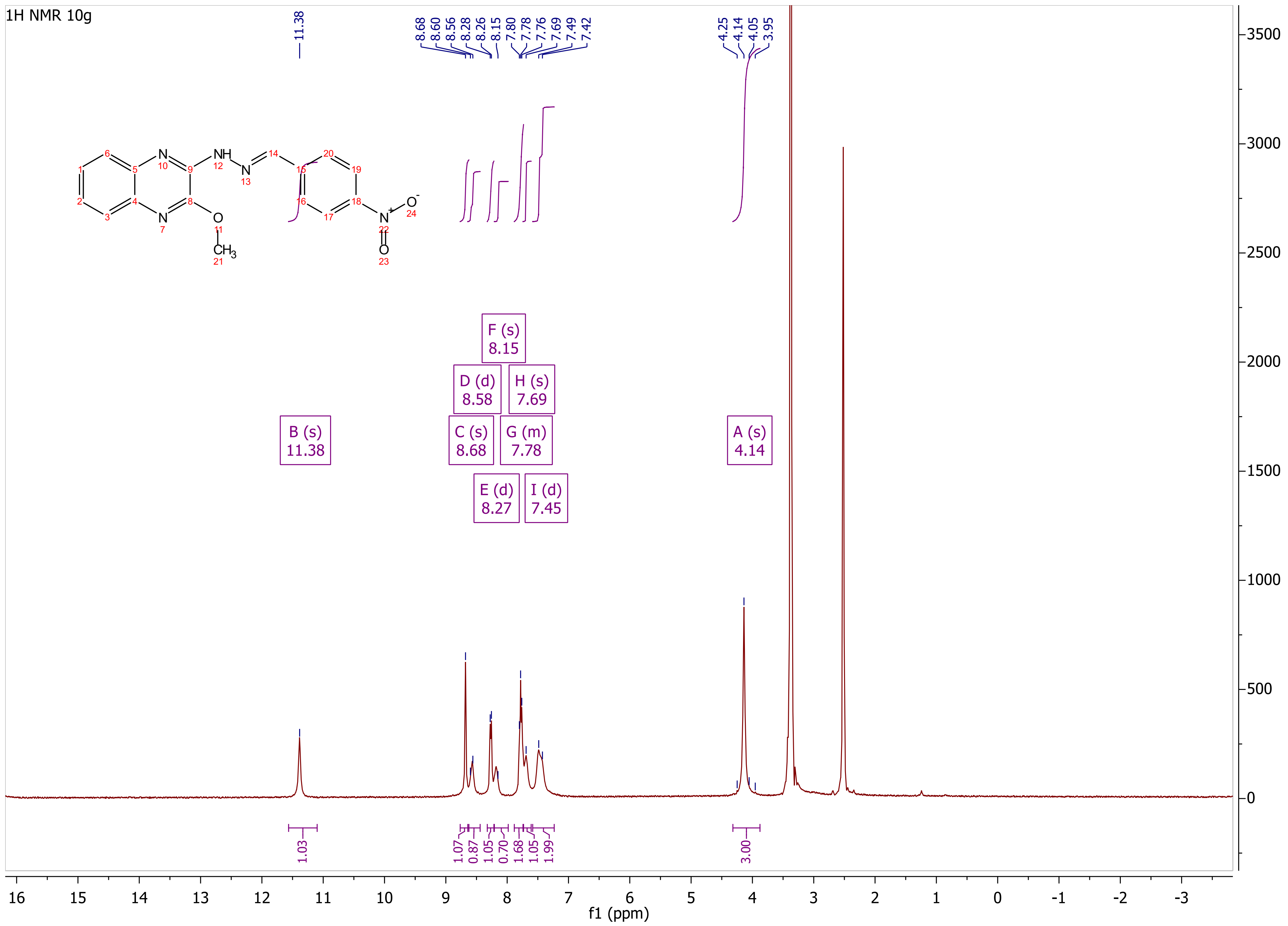
1H NMR 10g
11.38
8.68
8.60
8.56
8.28
8.26
8.15
7.80
7.78
7.76
7.69
7.49
7.42
4.25
4.14
4.05
3.95
F (s)
8.15
D (d)
8.58
H (s)
7.69
B (s)
11.38
C (s)
8.68
G (m)
7.78
A (s)
4.14
E (d)
8.27
I (d)
7.45
1.03
1.07
0.87
1.05
0.70
1.68
1.05
1.99
3.00
f1 (ppm)

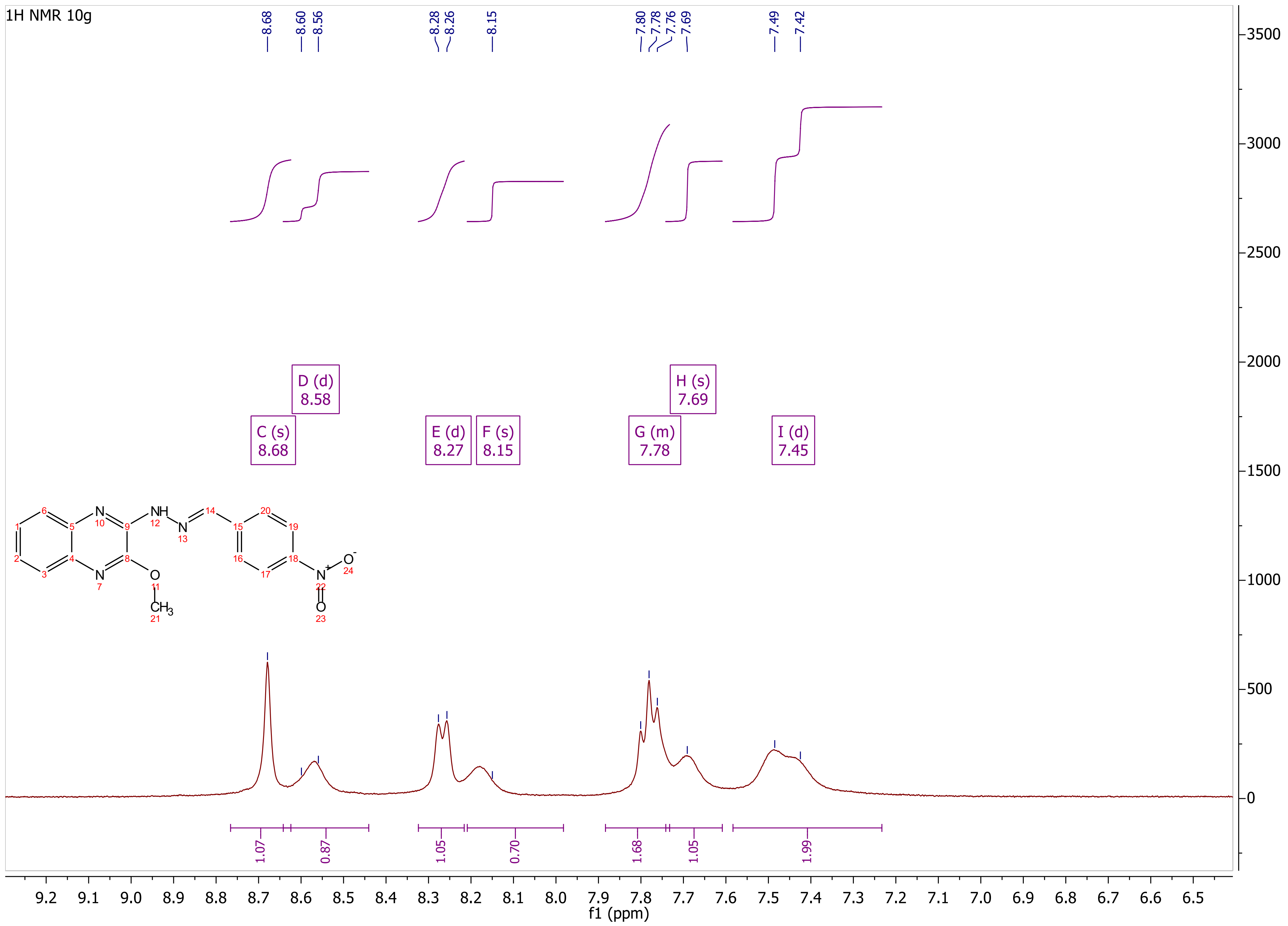
1H NMR 10g
8.68
8.60
8.56
8.28
8.26
8.15
7.80
7.78
7.76
7.69
7.49
7.42
D (d) 8.58
H (s) 7.69
C (s) 8.68
E (d) 8.27
F (s) 8.15
G (m) 7.78
I (d) 7.45
1.07
0.87
1.05
0.70
1.68
1.05
1.99
f1 (ppm)

13C NMR of 10g

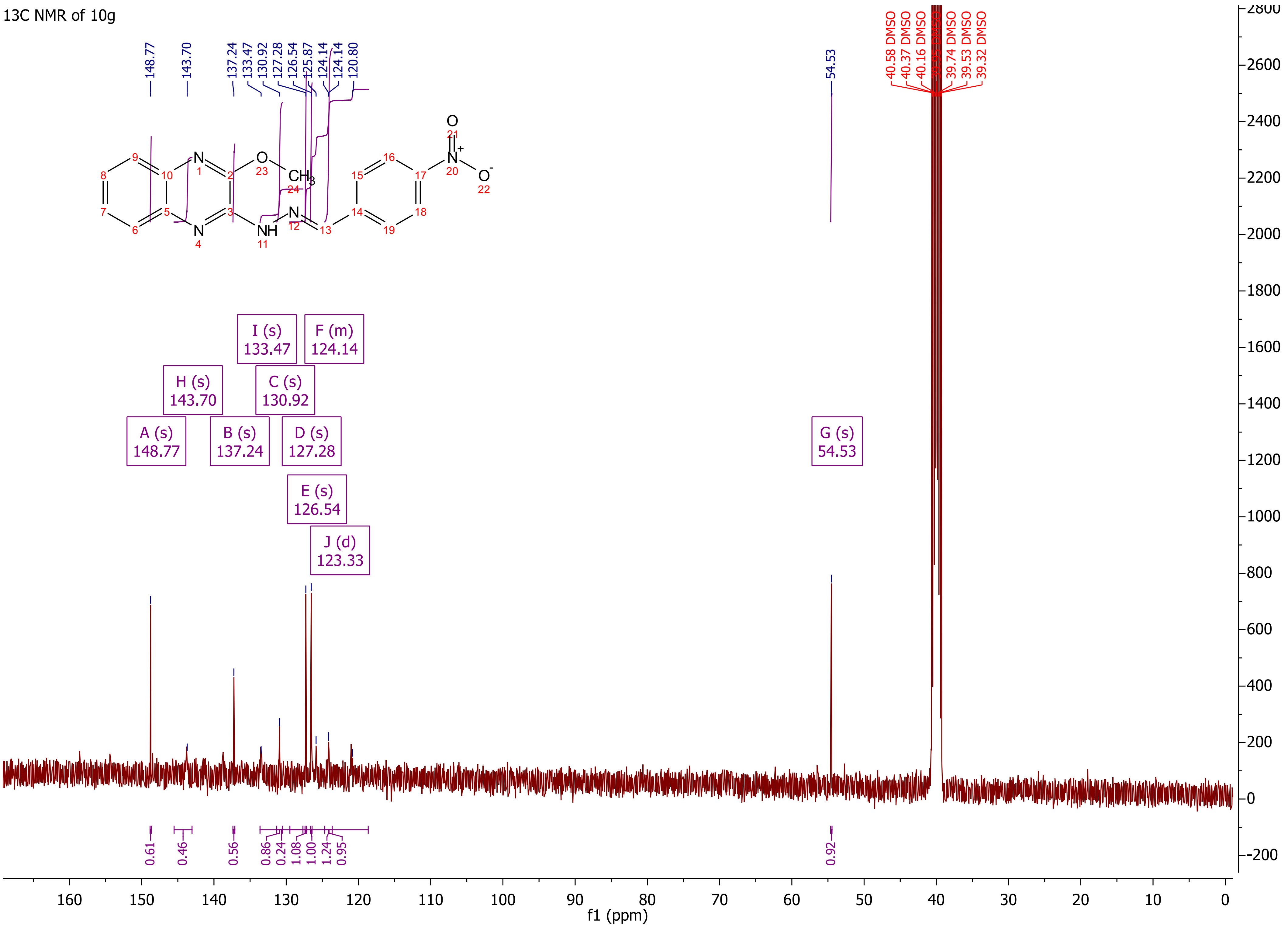

13C NMR of 10g

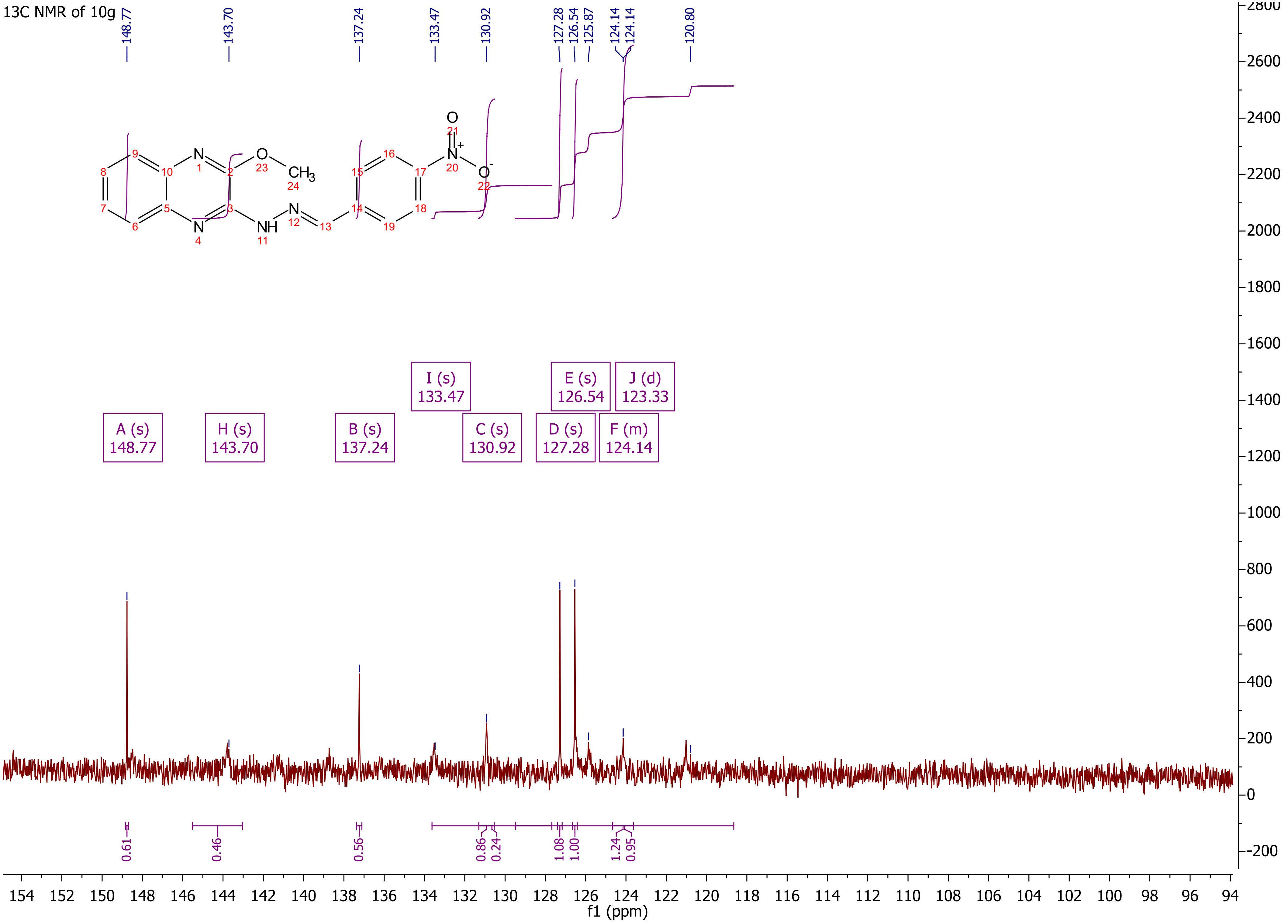

13C NMR of 10g

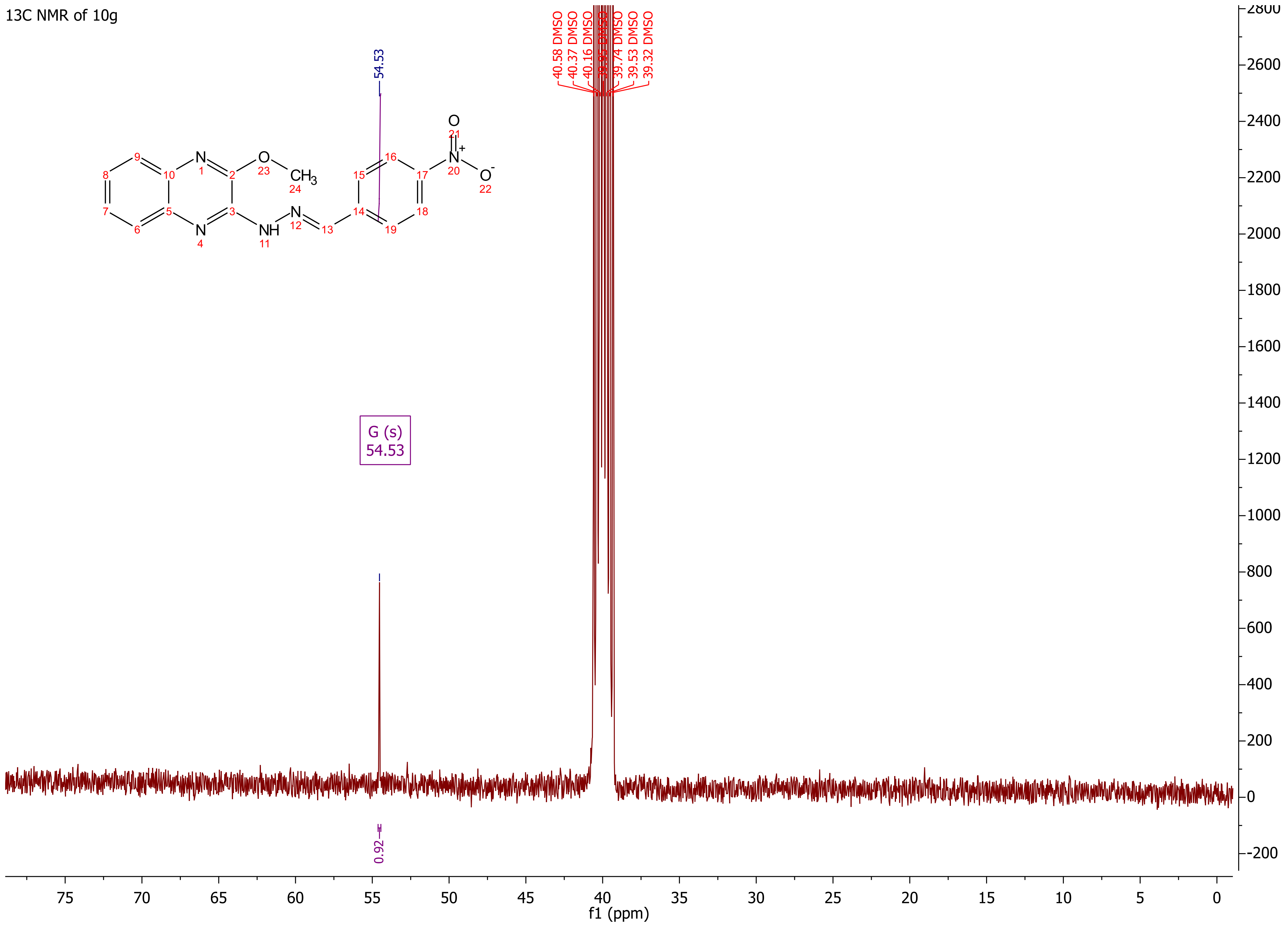

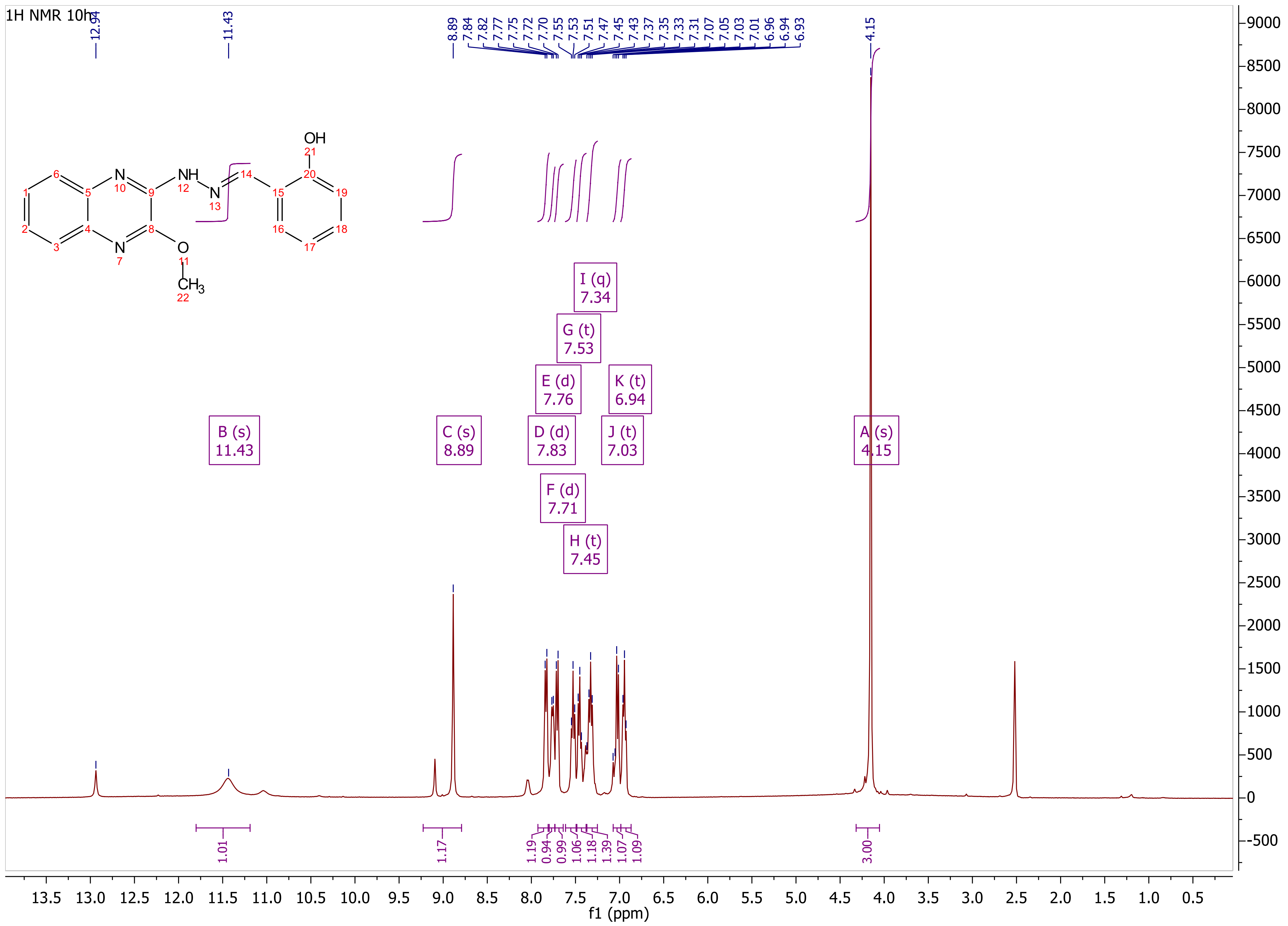

1H NMR 10h
12.94
11.43
8.89
7.84
7.82
7.77
7.75
7.72
7.70
7.55
7.53
7.51
7.47
7.45
7.43
7.37
7.35
7.33
7.31
7.07
7.05
7.03
7.01
6.96
6.94
6.93
4.15
OH
NH
N
O
CH3
I (q) 7.34
G (t) 7.53
E (d) 7.76
K (t) 6.94
B (s) 11.43
C (s) 8.89
D (d) 7.83
J (t) 7.03
A (s) 4.15
F (d) 7.71
H (t) 7.45
1.01
1.17
1.19
0.94
0.99
1.06
1.18
1.39
1.07
1.09
3.00
f1 (ppm)

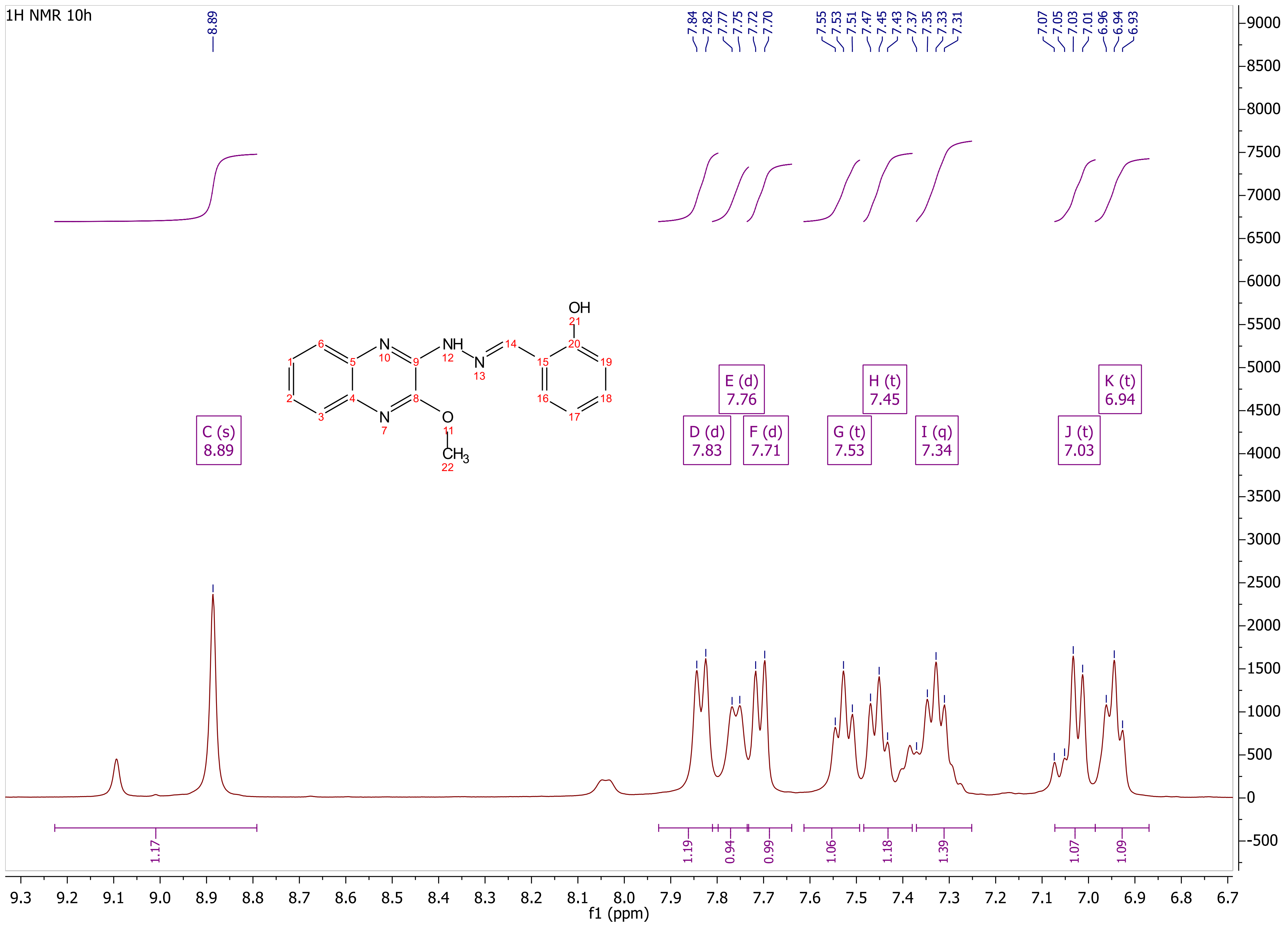

1H NMR 10h
C (s) 8.89
D (d) 7.83
E (d) 7.76
F (d) 7.71
G (t) 7.53
H (t) 7.45
I (q) 7.34
J (t) 7.03
K (t) 6.94
f1 (ppm)

IR of compound 10i

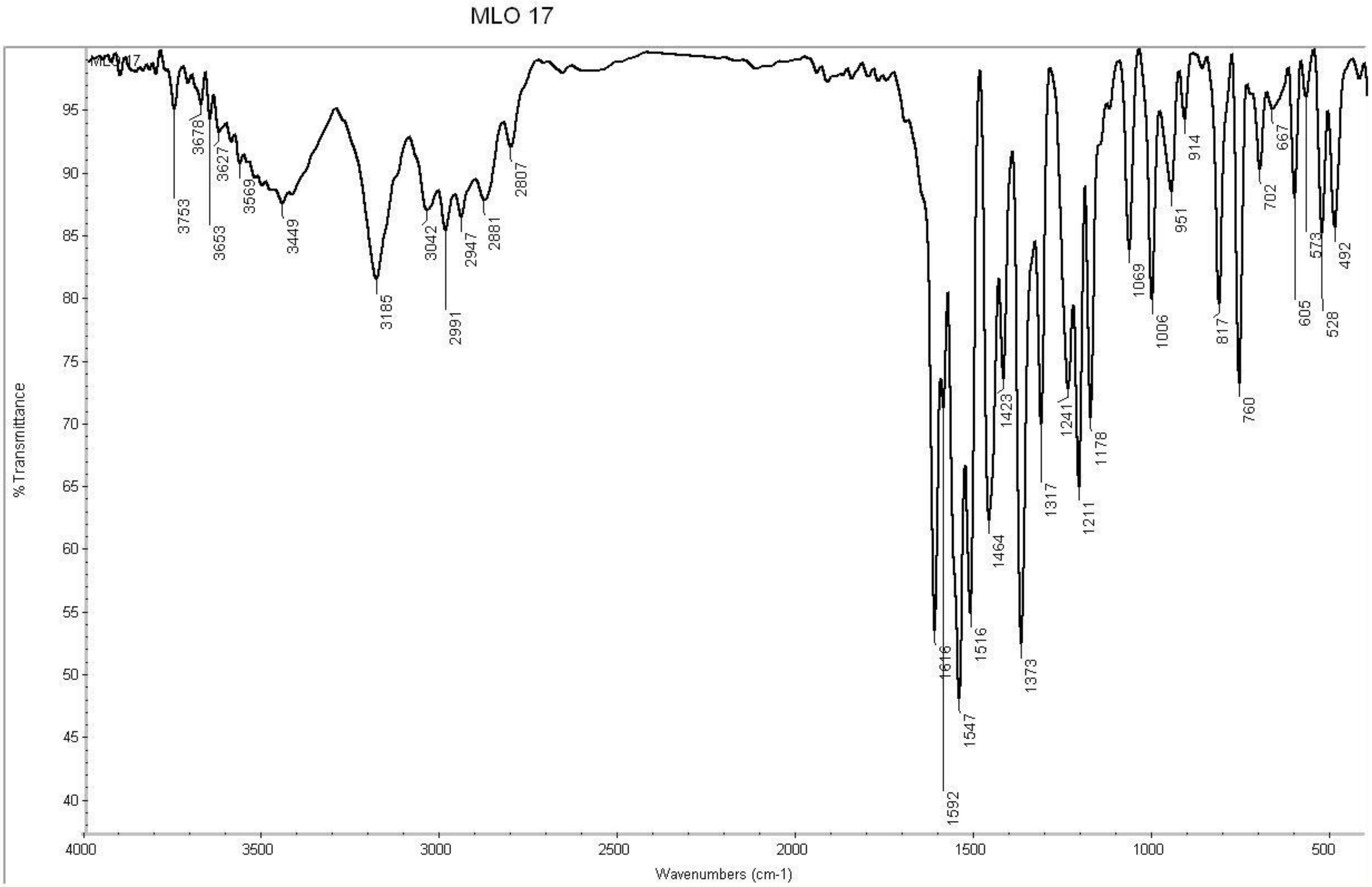

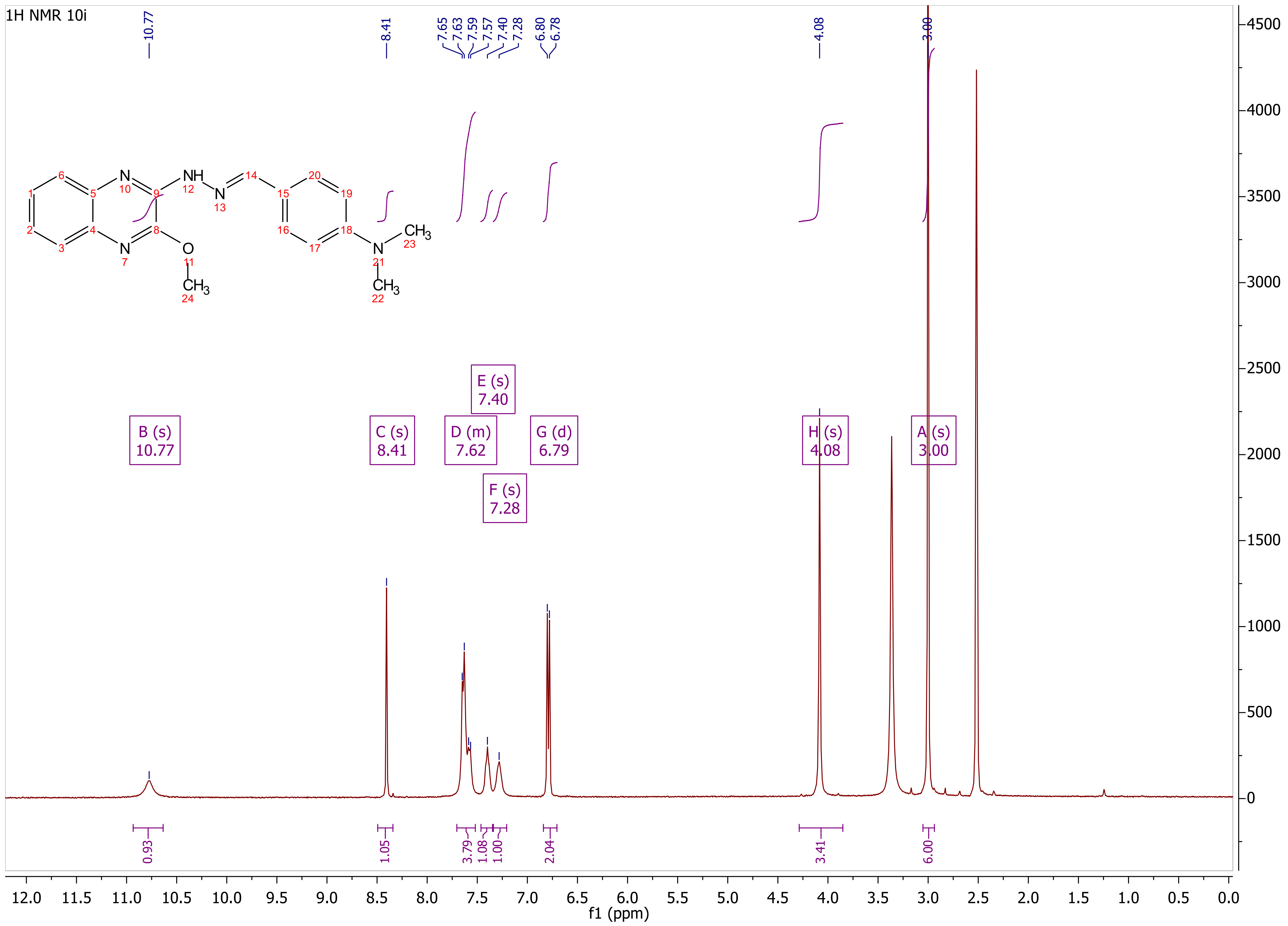
1H NMR 10i
10.77
8.41
7.65
7.63
7.59
7.57
7.40
7.28
6.80
6.78
4.08
3.00
B (s) 10.77
C (s) 8.41
D (m) 7.62
E (s) 7.40
F (s) 7.28
G (d) 6.79
H (s) 4.08
A (s) 3.00
0.93
1.05
3.79
1.08
1.00
2.04
3.41
6.00
f1 (ppm)

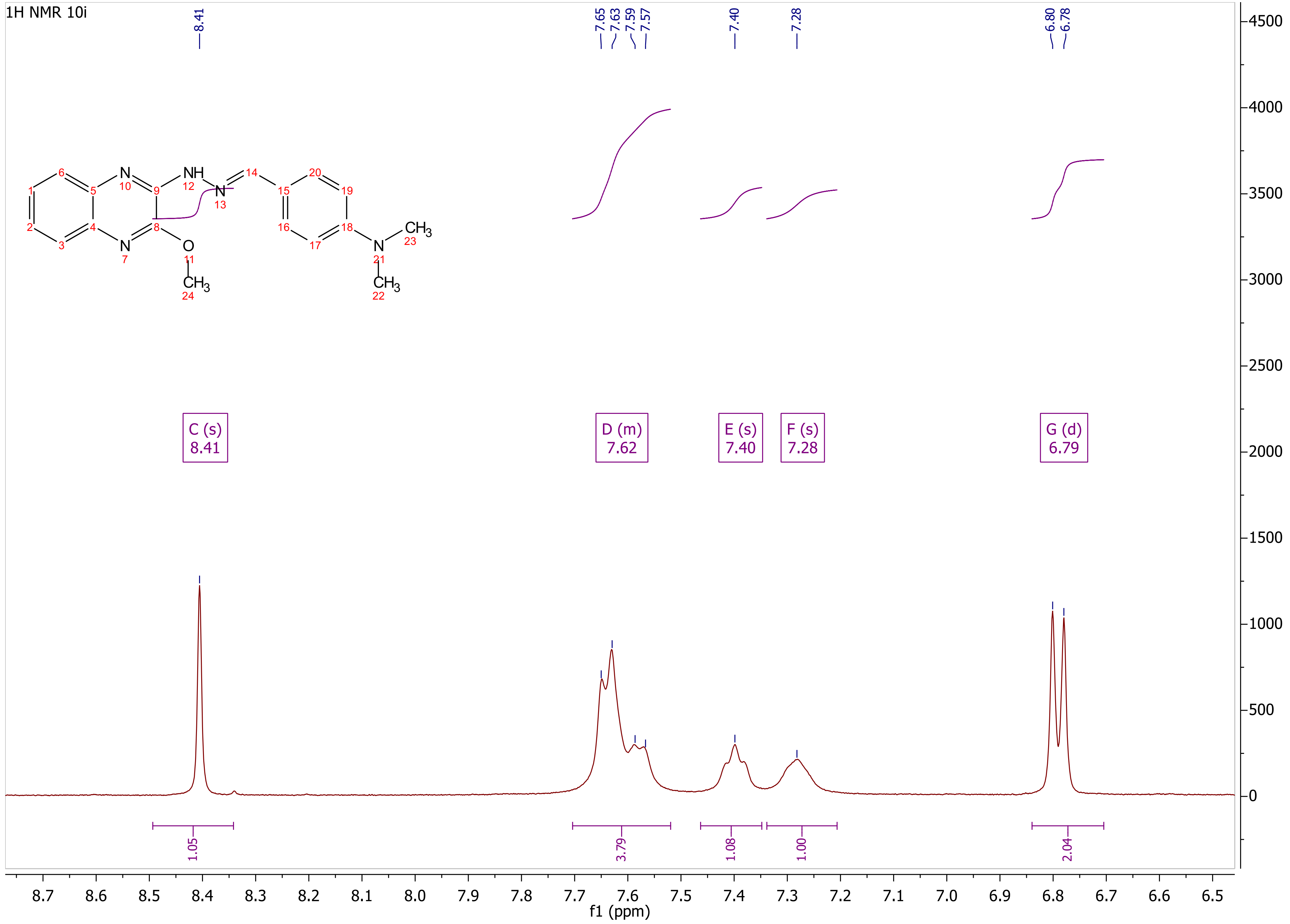

1H NMR 10i
8.41
7.65
7.63
7.59
7.57
7.40
7.28
6.80
6.78
C (s) 8.41
D (m) 7.62
E (s) 7.40
F (s) 7.28
G (d) 6.79
1.05
3.79
1.08
1.00
2.04
f1 (ppm)

IR of compond 11

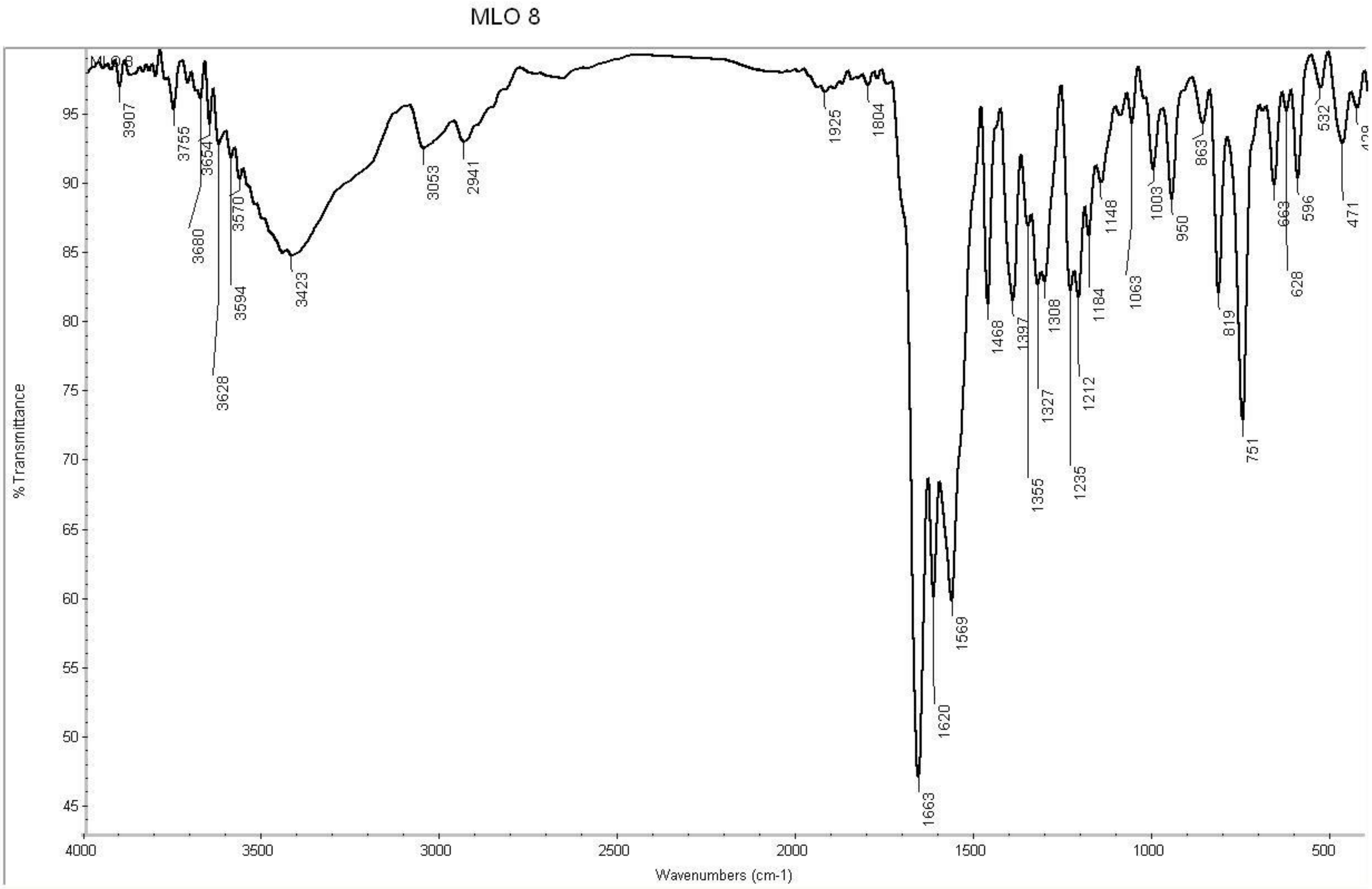

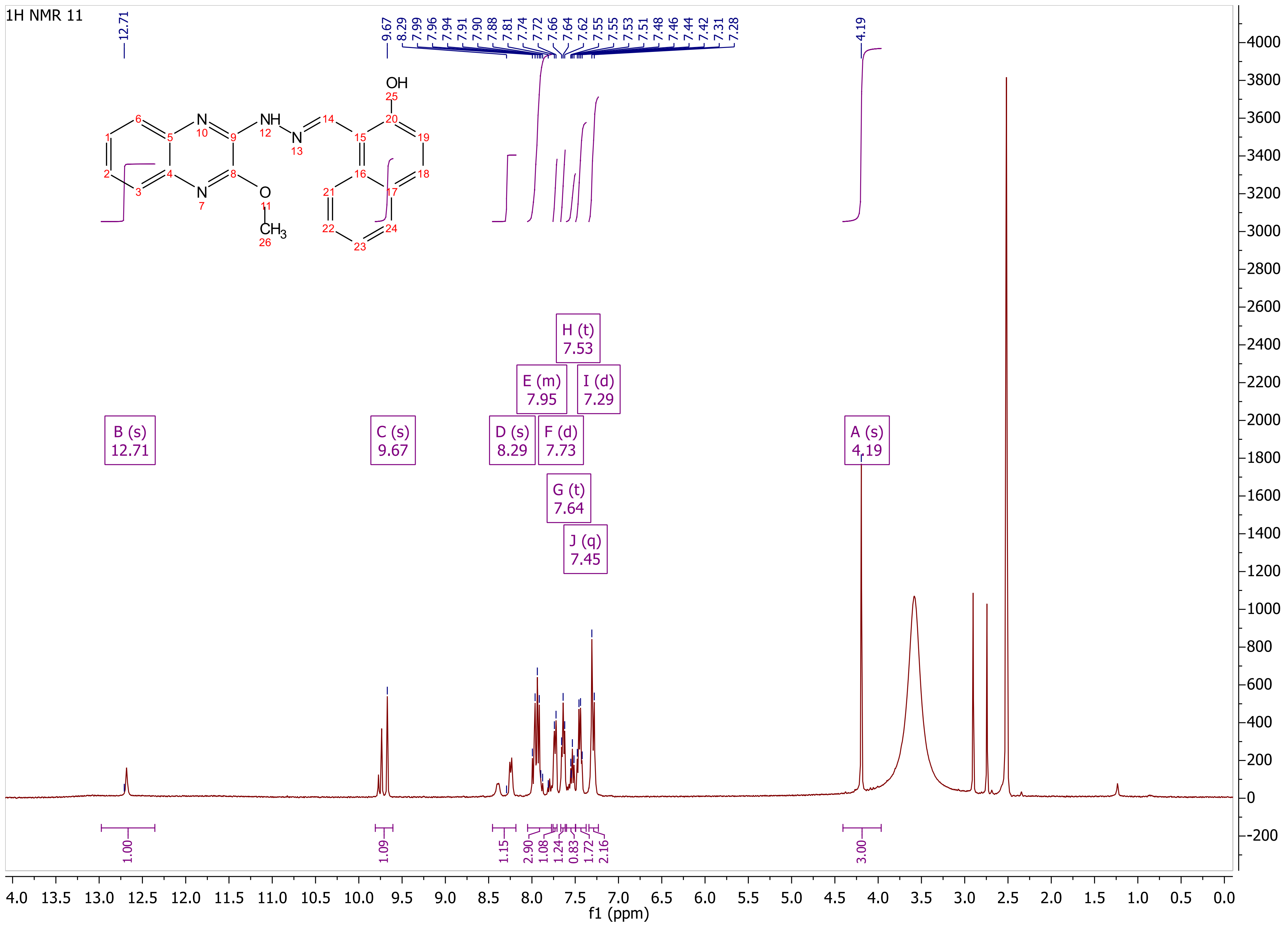

1H NMR 11
B (s) 12.71
C (s) 9.67
D (s) 8.29
E (m) 7.95
F (d) 7.73
G (t) 7.64
H (t) 7.53
J (q) 7.45
I (d) 7.29
A (s) 4.19
f1 (ppm)

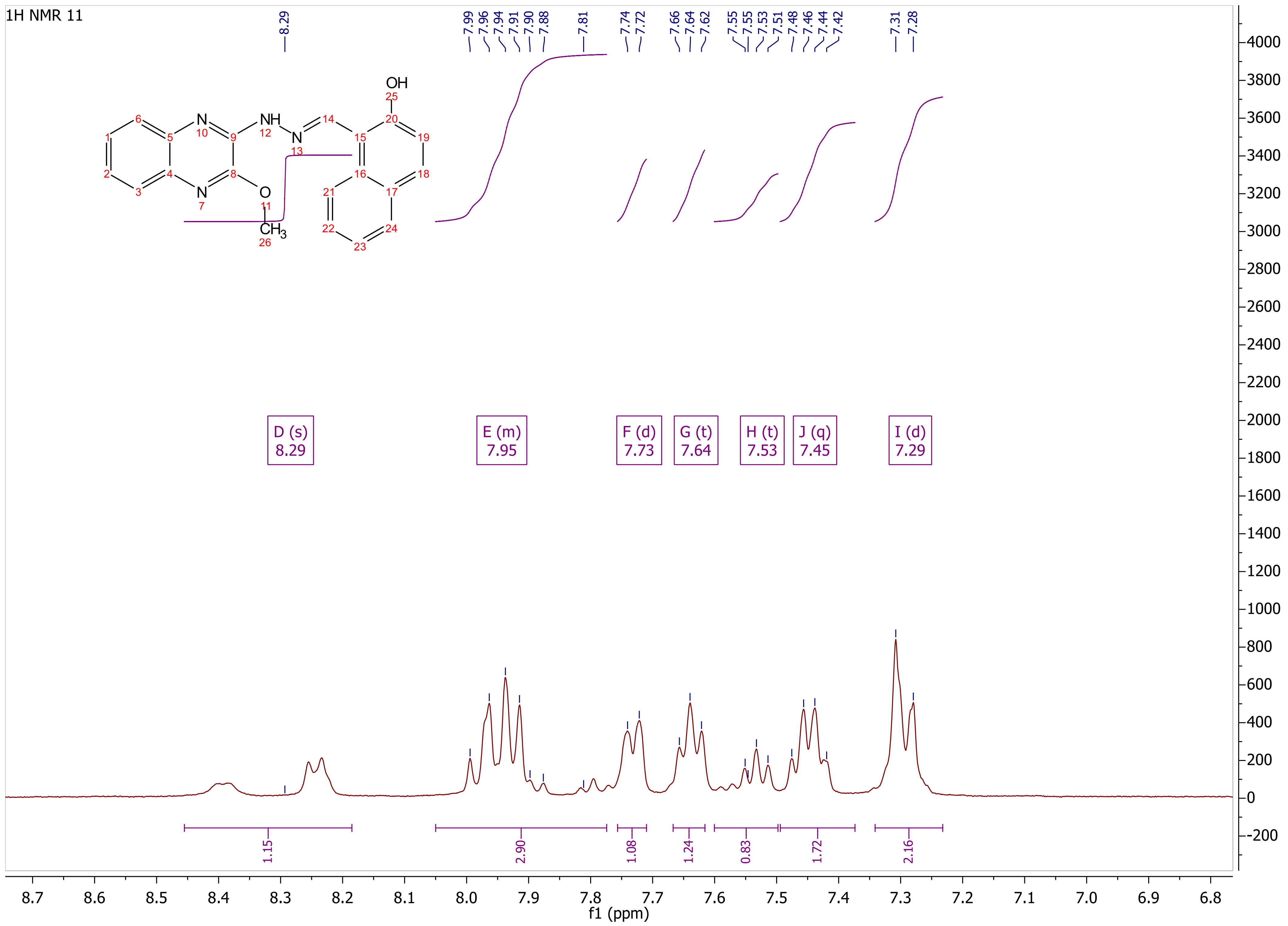

1H NMR 11
8.29
7.99 7.96 7.94 7.91 7.90 7.88 7.81 7.74 7.72 7.66 7.64 7.62 7.55 7.55 7.53 7.51 7.48 7.46 7.44 7.42 7.31 7.28
OH
NH
N
CH3
D (s) 8.29
E (m) 7.95
F (d) 7.73
G (t) 7.64
H (t) 7.53
J (q) 7.45
I (d) 7.29
1.15
2.90
1.08
1.24
0.83
1.72
2.16
f1 (ppm)

IBRAHIM-HASSAN-11 #116 RT: 1.96 AV: 1 SB: 2 4.45 , 4.45 NL: 4.96E2
T: {0,0} + c EI Full ms [40.00-1000.00]

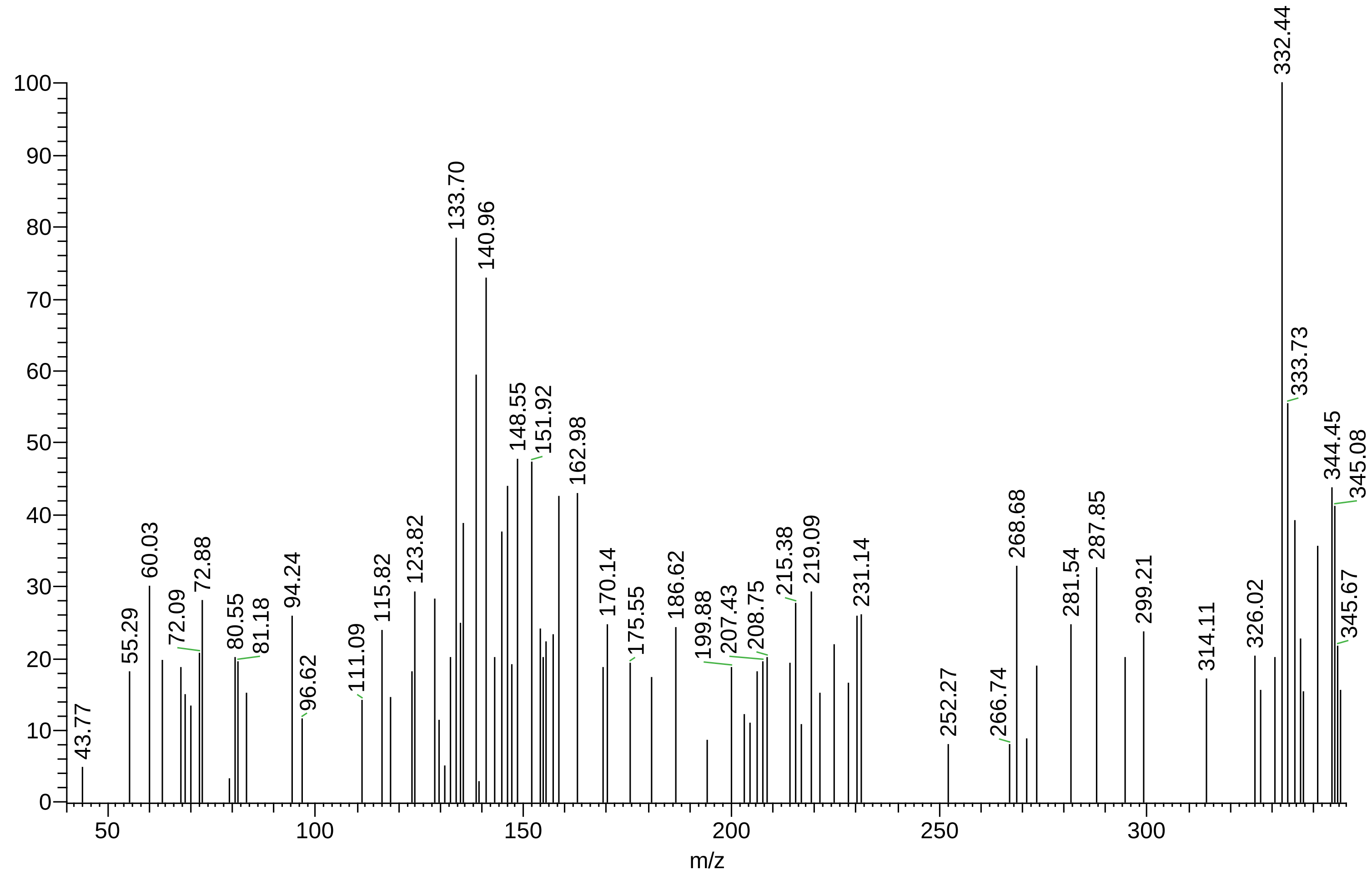

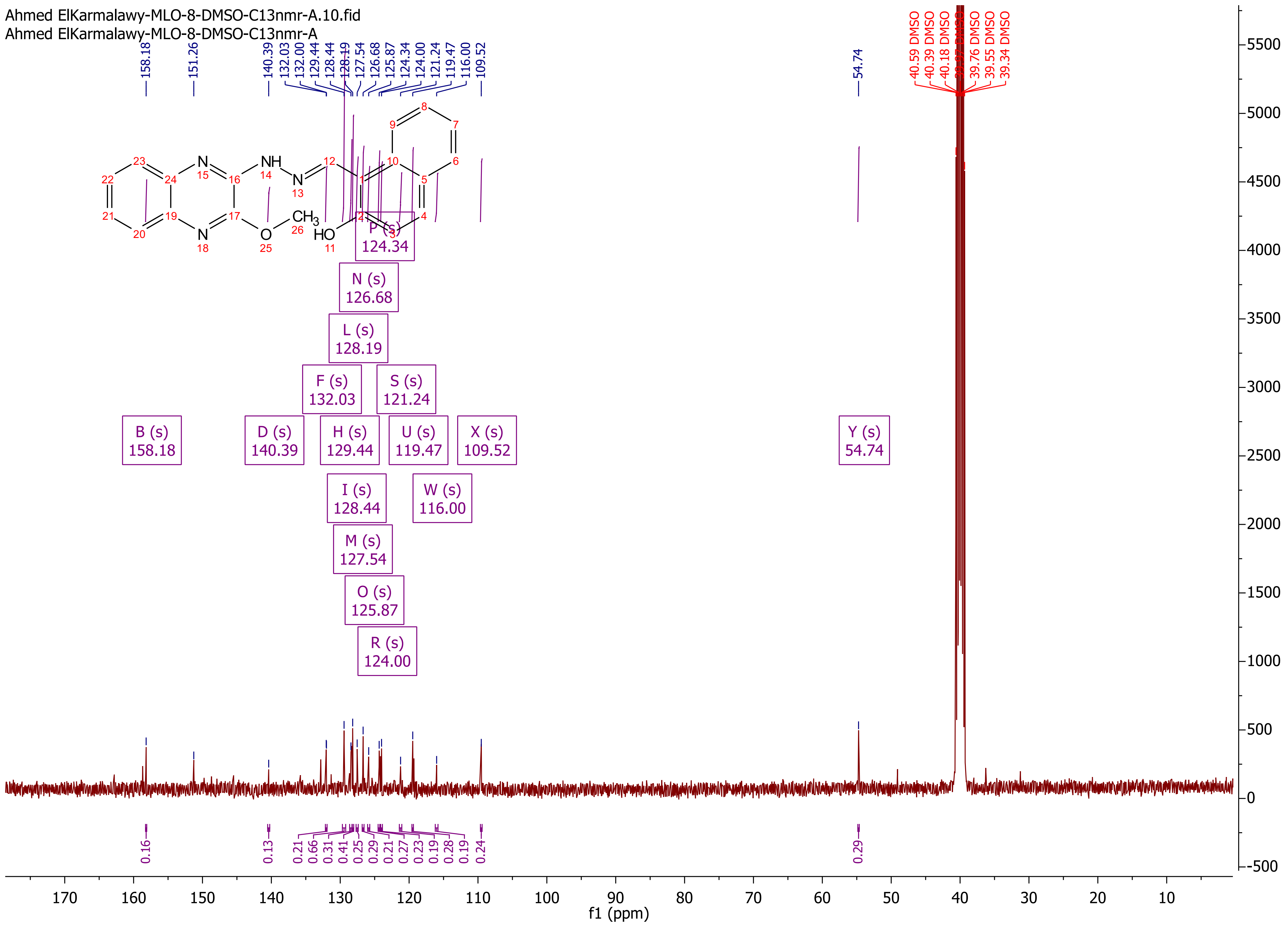

Ahmed ElKarmalawy-MLO-8-DMSO-C13nmr-A.10.fid
Ahmed ElKarmalawy-MLO-8-DMSO-C13nmr-A
158.18
151.26
140.39
132.03
132.00
129.44
128.44
128.19
127.54
126.68
125.87
124.34
124.00
121.24
119.47
116.00
109.52
54.74
40.59 DMSO
40.39 DMSO
40.18 DMSO
39.76 DMSO
39.55 DMSO
39.34 DMSO
NH
N
N
N
O
CH3
HO
P (s) 124.34
N (s) 126.68
L (s) 128.19
F (s) 132.03
S (s) 121.24
B (s) 158.18
D (s) 140.39
H (s) 129.44
U (s) 119.47
X (s) 109.52
Y (s) 54.74
I (s) 128.44
W (s) 116.00
M (s) 127.54
O (s) 125.87
R (s) 124.00
0.16
0.13
0.21
0.66
0.31
0.41
0.25
0.29
0.21
0.27
0.23
0.19
0.28
0.19
0.24
0.29
f1 (ppm)

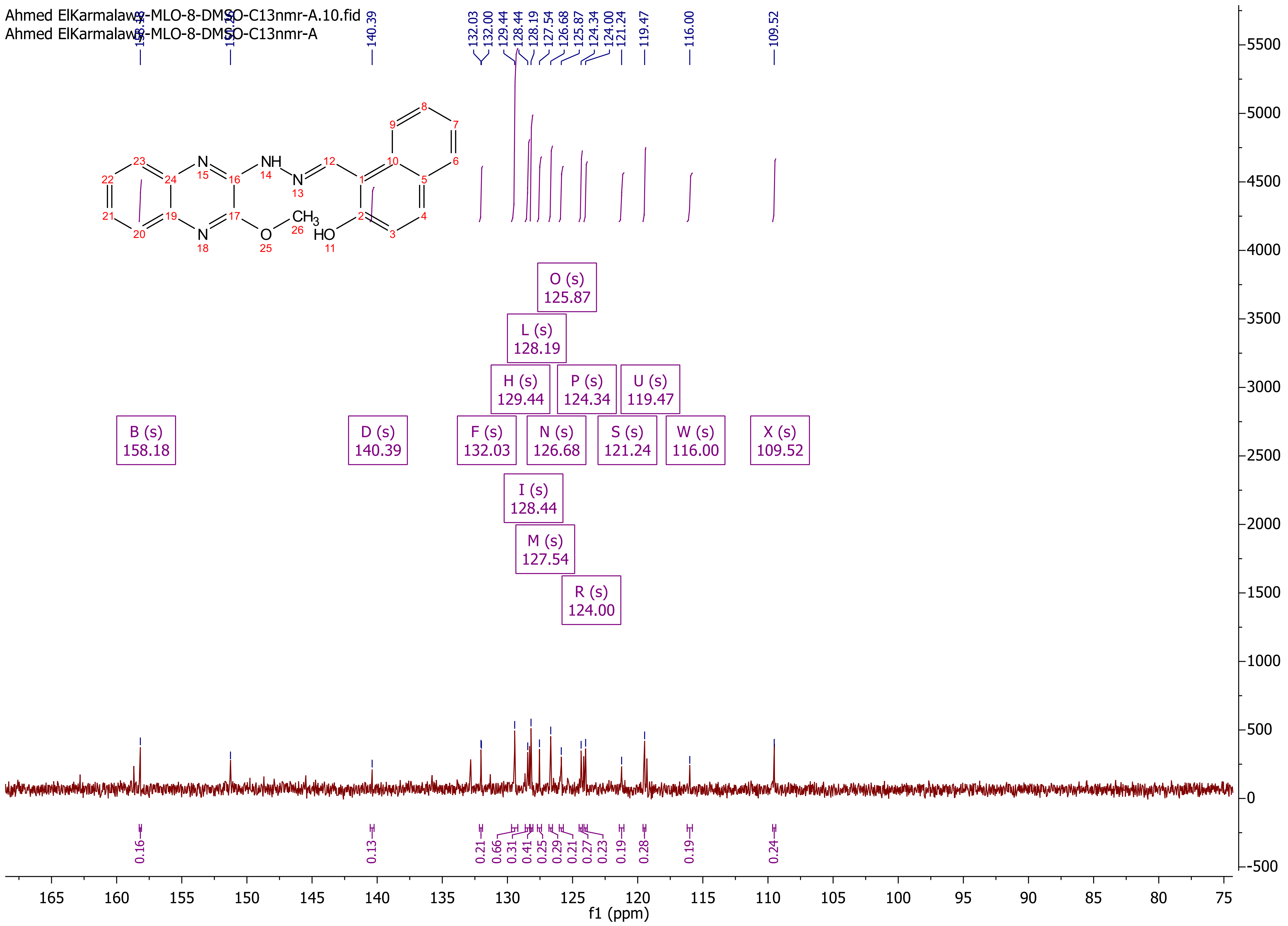

Ahmed ElKarmalawy-MLO-8-DMSO-C13nmr-A.10.fid
Ahmed ElKarmalawy-MLO-8-DMSO-C13nmr-A
140.39
132.03
132.00
129.44
128.44
128.19
127.54
126.68
125.87
124.34
124.00
121.24
119.47
116.00
109.52
B (s) 158.18
D (s) 140.39
F (s) 132.03
H (s) 129.44
I (s) 128.44
L (s) 128.19
M (s) 127.54
N (s) 126.68
O (s) 125.87
P (s) 124.34
R (s) 124.00
S (s) 121.24
U (s) 119.47
W (s) 116.00
X (s) 109.52
0.16
0.13
0.21
0.66
0.31
0.41
0.25
0.29
0.21
0.27
0.23
0.19
0.28
0.19
0.24
f1 (ppm)

Ahmed ElKarmalawy-MLO-8-DMSO-C13nmr-A.10.fid
Ahmed ElKarmalawy-MLO-8-DMSO-C13nmr-A

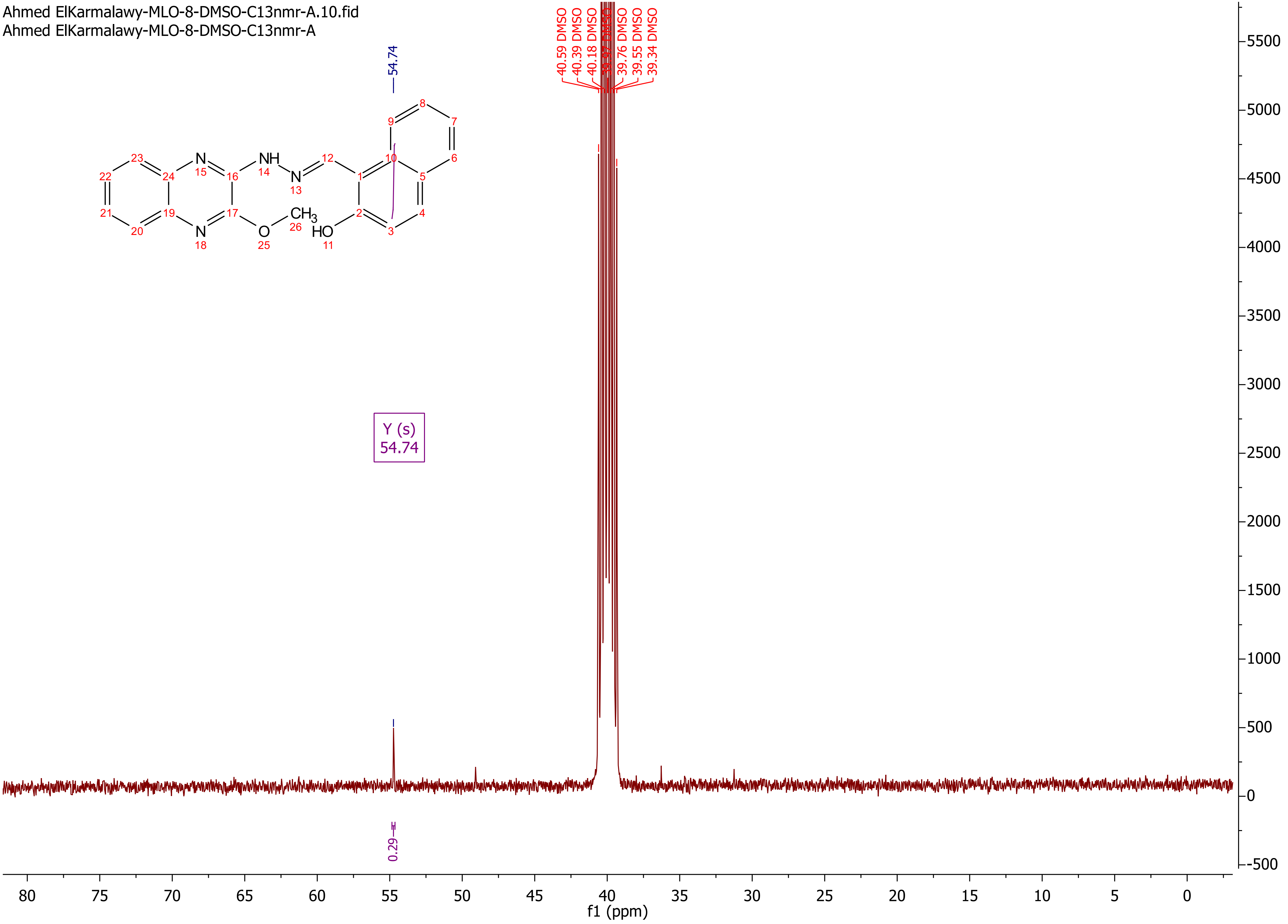

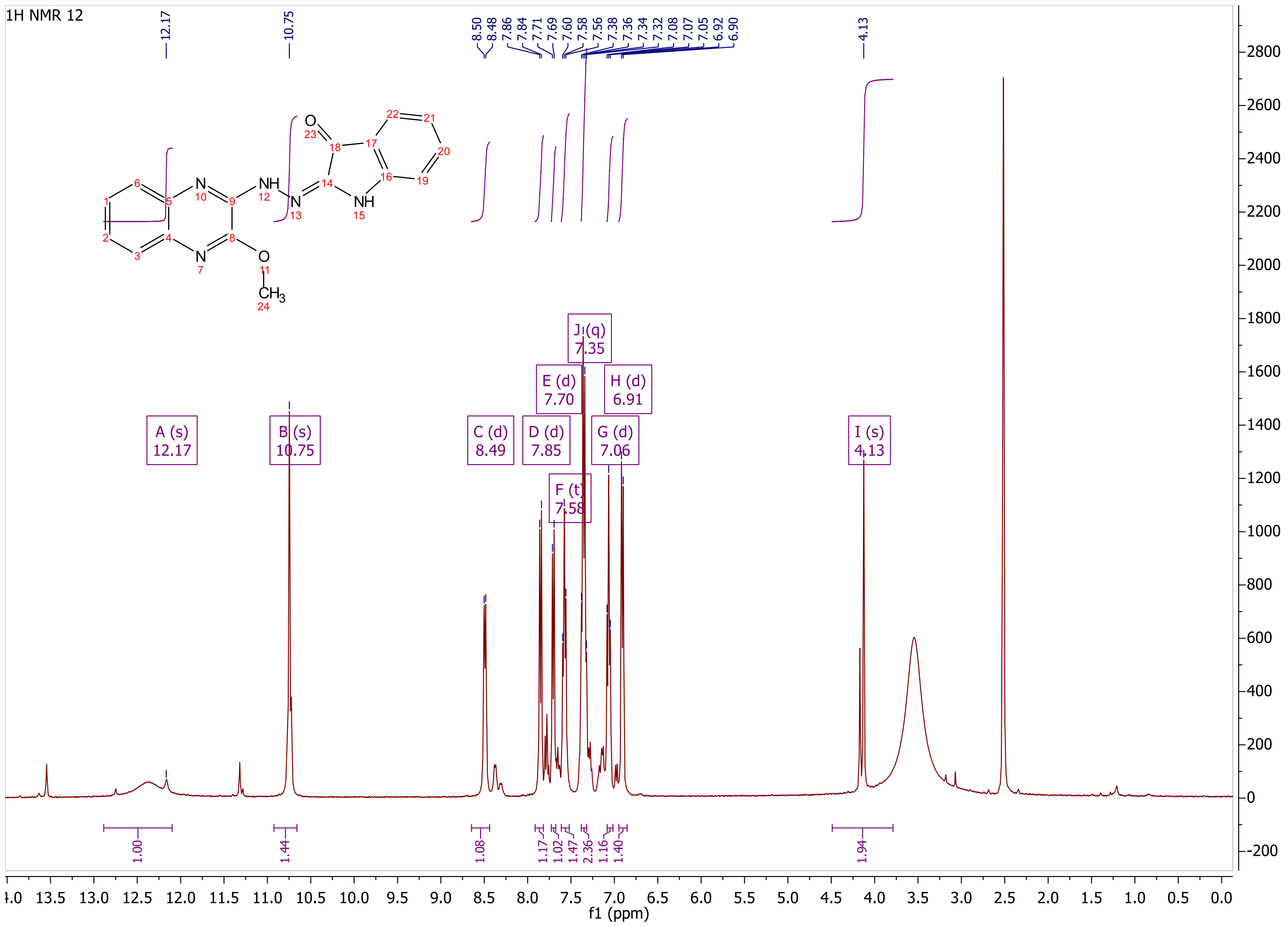

1H NMR 12
12.17
10.75
8.50
8.48
7.86
7.84
7.71
7.69
7.60
7.58
7.56
7.38
7.36
7.34
7.32
7.08
7.07
7.05
6.92
6.90
4.13
A (s) 12.17
B (s) 10.75
C (d) 8.49
D (d) 7.85
E (d) 7.70
F (t) 7.58
J (q) 7.35
G (d) 7.06
H (d) 6.91
I (s) 4.13
1.00
1.44
1.08
1.17
1.02
1.47
2.36
1.16
1.40
1.94
f1 (ppm)

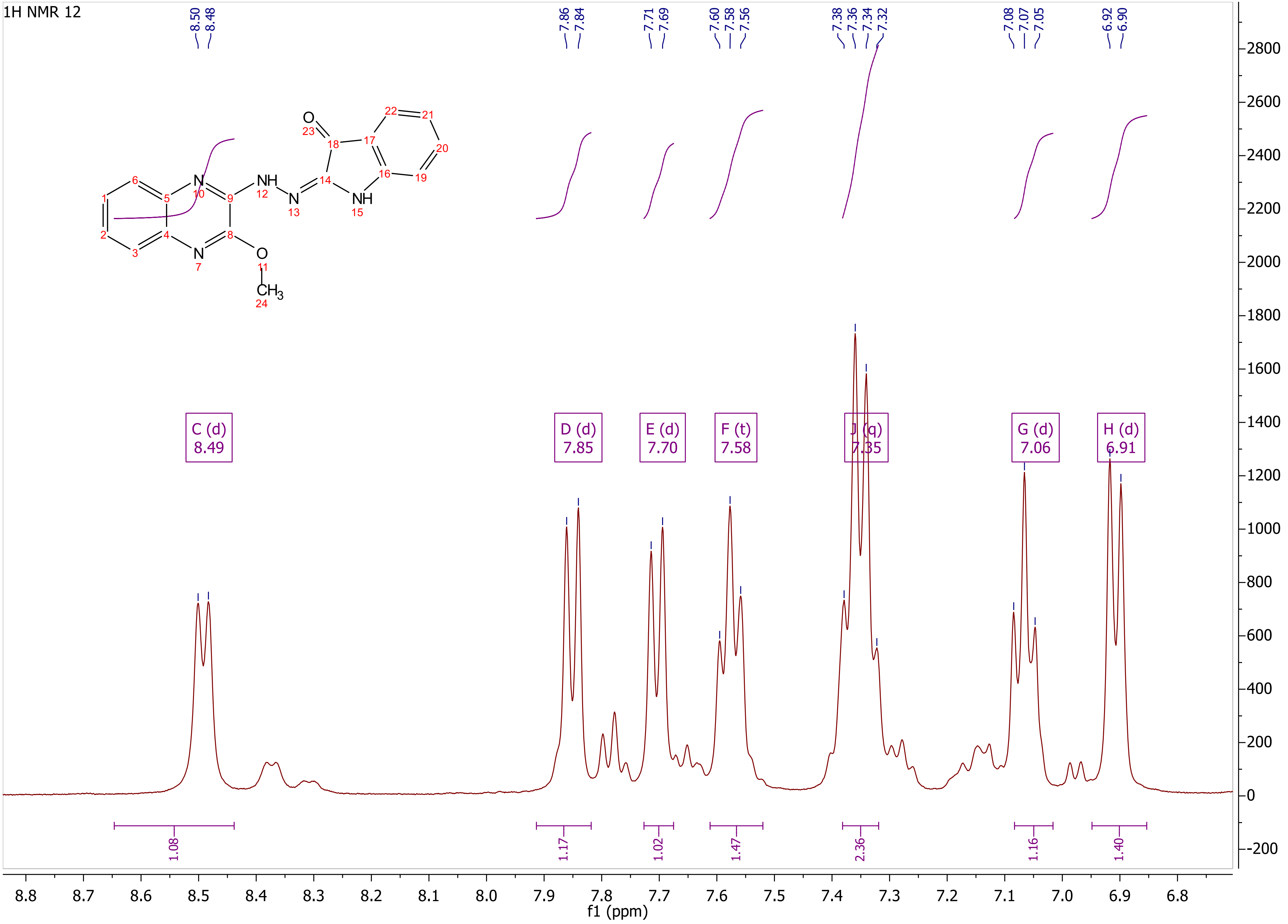

1H NMR 12
8.50
8.48
7.86
7.84
7.71
7.69
7.60
7.58
7.56
7.38
7.36
7.34
7.32
7.08
7.07
7.05
6.92
6.90
C (d) 8.49
D (d) 7.85
E (d) 7.70
F (t) 7.58
J (q) 7.35
G (d) 7.06
H (d) 6.91
1.08
1.17
1.02
1.47
2.36
1.16
1.40
f1 (ppm)

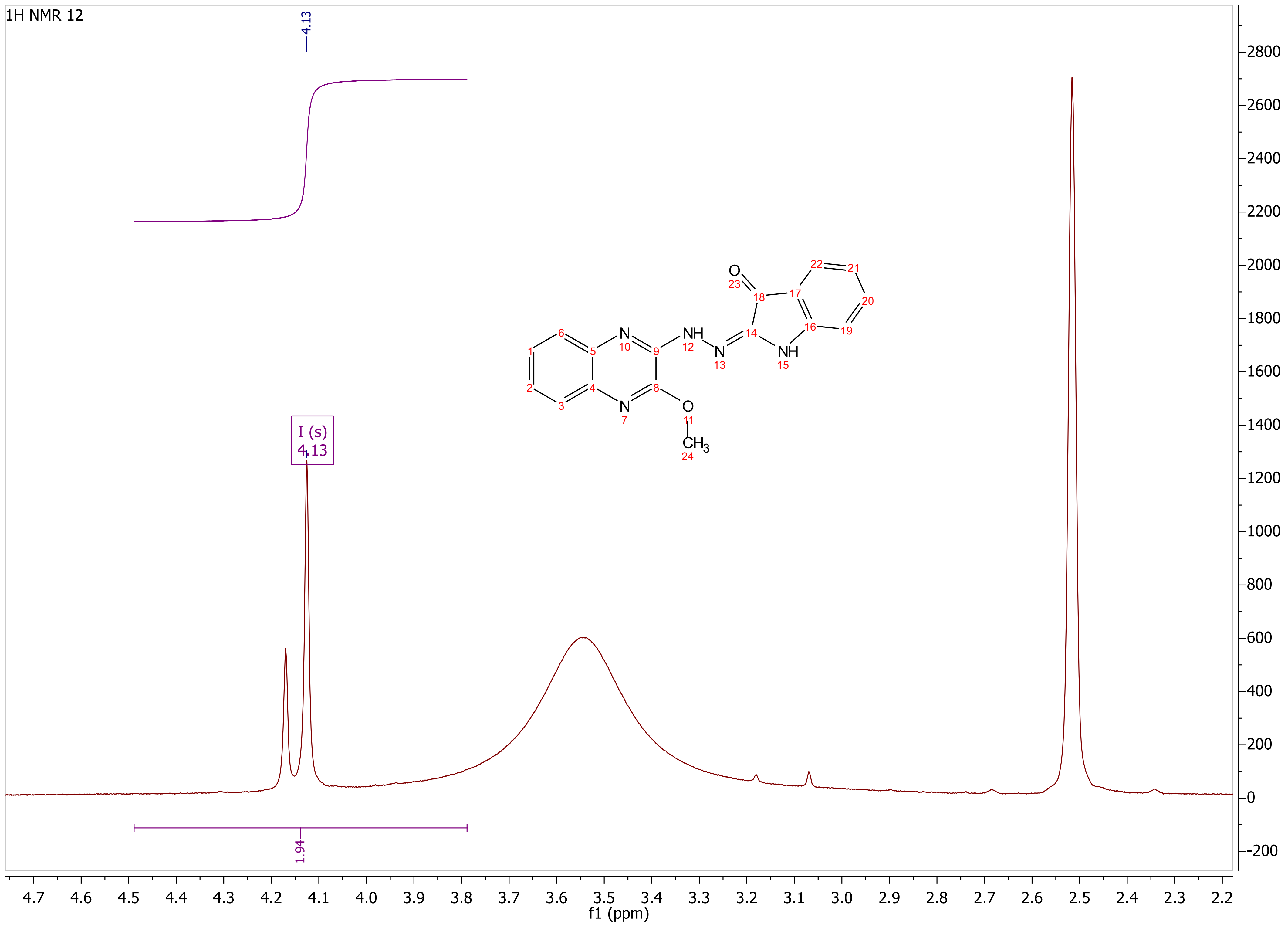
1H NMR 12
4.13
I (s)
4.13
1.94
f1 (ppm)
O
NH
N
NH
CH3
4.7 4.6 4.5 4.4 4.3 4.2 4.1 4.0 3.9 3.8 3.7 3.6 3.5 3.4 3.3 3.2 3.1 3.0 2.9 2.8 2.7 2.6 2.5 2.4 2.3 2.2
2800 2600 2400 2200 2000 1800 1600 1400 1200 1000 800 600 400 200 0 -200
